# The HOXD9-mediated PAXIP1-AS1 regulates gastric cancer progression through PABPC1/PAK1 modulation

**DOI:** 10.1038/s41419-023-05862-5

**Published:** 2023-05-24

**Authors:** Jiaying Li, Miaomiao Pei, Wushuang Xiao, Xuehua Liu, Linjie Hong, Zhen Yu, Ying Peng, Jieming Zhang, Ping Yang, Jianjiao Lin, Xiaosheng Wu, Zhizhao Lin, Weimei Tang, Fachao Zhi, Guoxin Li, Li Xiang, Aimin Li, Side Liu, Ye Chen, Jide Wang

**Affiliations:** 1 Guangdong Provincial Key Laboratory of Gastroenterology, Department of Gastroenterology, Nanfang Hospital, Southern Medical University, Guangzhou, 510515 China; 2 Department of Gastroenterology, Shunde Hospital, Southern Medical University, Foshan, 528300 China; 3 Department of Gastroenterology, The Second Affiliated Hospital, School of Medicine, The Chinese University of Hong Kong, Shenzhen & Longgang District People’s Hospital of Shenzhen, Shenzhen, 518172 China; 4 Department of General Surgery, Nanfang Hospital, Southern Medical University, Guangzhou, 510515 China; 5 Department of Gastroenterology, Integrative Clinical Microecology Center, Shenzhen Hospital, Southern Medical University, Shenzhen, 518000 China

**Keywords:** Metastasis, Long non-coding RNAs, Transcriptional regulatory elements, Ubiquitylation, RNA decay

## Abstract

Long non-coding RNAs (lncRNAs) have been functionally characterised in various diseases. LncRNA PAX-interacting protein 1-antisense RNA 1 (PAXIP1-AS1) has reportedly been associated with cancer development. However, its role in gastric cancer (GC) remains poorly understood. Here, we showed that PAXIP1-AS1 was transcriptionally repressed by homeobox D9 (HOXD9) and was significantly downregulated in GC tissues and cells. Decreased expression of PAXIP1-AS1 was positively correlated with tumour progression, while PAXIP1-AS1 overexpression inhibited cell growth and metastasis both in vitro and in vivo. PAXIP1-AS1 overexpression significantly attenuated HOXD9-enhanced epithelial-to-mesenchymal transition (EMT), invasion and metastasis in GC cells. Poly(A)-binding protein cytoplasmic 1 (PABPC1), an RNA-binding protein, was found to enhance the stability of PAK1 mRNA, leading to EMT progress and GC metastasis. PAXIP1-AS1 was found to directly bind to and destabilise PABPC1, thereby regulating EMT and metastasis of GC cells. In summary, PAXIP1-AS1 suppressed metastasis, and the HOXD9/PAXIP1-AS1/PABPC1/PAK1 signalling axis may be involved in the progression of GC.

## Introduction

Gastric cancer (GC) is the fourth most diagnosed cancer and the third most common cause of cancer-related death worldwide [1]. The prognosis of patients with GC remains dismal despite improvements in surgical and adjuvant treatment approaches, with a five-year overall survival rate of less than 25%. The high mortality rate is attributed to the specific biological features of this disease, together with un-timely diagnosis, delayed clinical manifestation, and high rates of invasion and metastasis [2]. Therefore, a better understanding of the molecular mechanisms underlying the progression and metastasis of GC is required.

Homeobox D9 (HOXD9) belongs to the homeobox (HOX) gene family and is known to be a potential transcription factor that regulates coding genes as well as non-coding RNAs [3, 4]. Previous studies indicate that HOXD9 participates in the development and progression of certain cancers. For example, HOXD9 contributes to both cell proliferation and survival in gliomas [5]. HOXD9 also induced epithelial-to-mesenchymal transition (EMT) in tumour cells, thereby maintaining the invasive potential of cancer cells [6].

Long non-coding RNAs (lncRNAs) are endogenous RNAs longer than 200 nucleotides, and have been shown to play important roles in the development and progression of various types of cancers [7, 8]. For instance, the eukaryotic translation initiation factor 3 subunit J-divergent transcript (EIF3J-DT) can trigger chemoresistance in gastric cancer by activating autophagy [8], and the poly (RC)- binding protein 1-antisense RNA 1 (PCBP1-AS1) inhibits lung adenocarcinoma metastasis by suppressing EMT progression [9]. PAX-interacting protein 1-antisense RNA 1 (PAXIP1-AS1), also known as PAXIP1 divergent transcript (PAXIP1-DT), is identified as an lncRNA [10]. It has been reported to be highly expressed in gliomas and is associated with poor prognosis [11]. PAXIP1-AS1 has been found to be activated by H3K27ac via the miR-6744-5p/PCBP2 axis in ovarian cancer [12]. However, whether PAXIP1-AS1 participates in EMT, invasion, and metastasis and whether its regulation is influenced by HOXD9 status in GC cells remain unknown.

LncRNAs function as platforms for complicated interactions between miRNAs, mRNA, proteins, and their combinations [13, 14]. Poly(A)-binding protein cytoplasmic 1 (PABPC1) is a member of the PABP family of proteins, which can regulate the stability and translation of mRNAs [15, 16]. Several lncRNAs have been identified to interact with certain polyadenylate-binding proteins and alter the stability or activity of their partners [17, 18]. For example, lncRNA RP11-286H15.1 binds to PABP cytoplasmic 4 (PABPC4) and promotes its ubiquitination, thus reducing the stability of tripartite motif-containing protein 37 (TRIM37) and cell division cycle protein 27 (CDC27) mRNAs in hepatocellular carcinoma (HCC) cells [19]. The lncRNA PCIR directly interacts with PABPC4 and thus increases the mRNA stability of transforming growth factor-beta activated kinase 1 binding protein 3 (TAB3) in breast cancer cells [20]. However, the relationships among PAXIP1-AS1, PABP cytoplasmic 1 (PABPC1) and their partners have not been determined yet.

In this study, we found that PAXIP1-AS1 was transcriptionally repressed by HOXD9 in human GC cells. PAXIP1-AS1 was found to inhibit the malignant properties of GC cells, both in vitro and in vivo. Meanwhile, PAXIP1-AS1 inhibited EMT, migration, and invasion of GC cells by binding to and destabilising the PABPC1 protein. Besides, PABPC1 contributed to GC progression by enhancing the stability of PAK1 mRNA. Thus, the HOXD9/PAXIP1-AS1/PABPC1/PAK1 signalling axis may play a role in GC progression.

## Results

### PAXIP1-AS1 is transcriptionally repressed by HOXD9

**Fig. 1:**
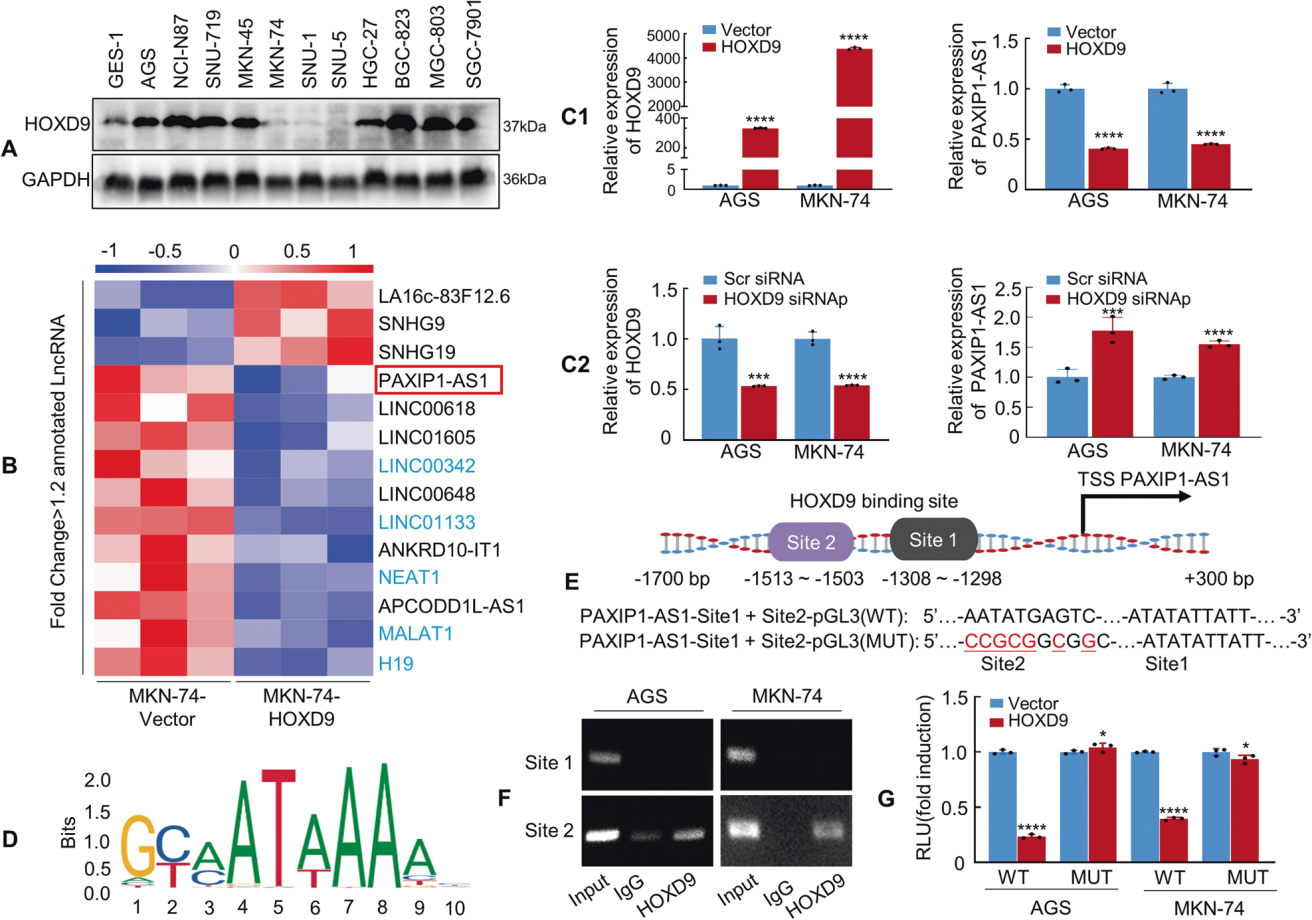
Homeobox D9 (HOXD9) represses transcription of PAX-interacting protein 1-antisense RNA 1 (PAXIP-AS1). **A** Whole lysates of human gastric cancer (GC) and normal mucous cell lines were collected, and HOXD9 expression was detected by western blotting. Glyceraldehyde 3-phosphate dehydrogenase (GAPDH) was used as the internal control. **B** Total RNA was extracted from LV-HOXD9 and LV-Vector MKN-74 cells and subjected to RNA sequencing analysis. The colour scale illustrates the relative expression of lncRNAs. **C1**, **C2** Vector and HOXD9 plasmid (**C1**) or Scr siRNA and HOXD9 siRNAp (**C2**) were transfected into GC cells. The expression of HOXD9 and PAXIP1-AS1 was detected in GC cells using qPCR. ****P < 0.001, Vector vs. HOXD9; ***P < 0.01 and ****P < 0.001, Scr siRNA vs. HOXD9 siRNAp. Scr siRNA, scrambled siRNA; siRNAp, siRNApool; siRNAp: siRNA1~ siRNA3, siRNAs: final concentration = 33.3%. **D** Transcriptional factor HOXD9 binding motif was predicted by Jaspar software. **E** Schematic diagram of PAXIP1**-**AS1 promoter with two potential HOXD9-binding sites and corresponding mutant binding sites. **F** ChIP assay demonstrated the direct binding of HOXD9 to the PAXIP1-AS1 promoter in AGS and MKN-74 cells. **G** Luciferase reporter assay showing the transcriptional regulation ability of HOXD9 to the PAXIP1-AS1 promoter. Luciferase constructs containing PAXIP1-AS1 full-length or mutant promoter were co-transfected with the HOXD9 or Vector plasmid into AGS and MKN-74 cells, and the luciferase activity was measured. Selective mutagenesis of −1503 to −1513 bp from the transcription start site resulted in a loss of response to HOXD9, indicating that this region was HOXD9-responsive (*n* = 3). *P > 0.05 and ****P < 0.001, Vector vs. HOXD9. WT, wild type and MUT, mutated.

We previously reported that the transcription factor HOXD9 promotes EMT-induced metastasis in gastrointestinal cancer [3, 21]. Here, we investigated the involvement of HOXD9 in lncRNA-mediated metastasis. We first confirmed the expression of HOXD9 protein in GC cell lines and showed that the expression levels in AGS, NCI-N87, SNU-719, MKN-45, HGC-27, BGC-823, MGC-803, and SGC-7901 cell lines were significantly higher than those in the GES-1 cell line, whereas MKN-74, SNU-1, and SNU-5 showed significantly lower expression (Fig. 1A). Secondly, comprehensive RNA sequencing analyses were conducted to obtain lncRNA and mRNA expression profiles. HOXD9 overexpression resulted in the downregulation of 54 lncRNAs and 495 protein-coding genes, whereas 11 lncRNAs and 229 protein-coding genes were upregulated above the 1.2-fold threshold level (Supplementary Table 1). Fourteen lncRNAs were differentially expressed and annotated according to the HUGO Gene Nomenclature Committee (HGNC) database (Fig. 1B). Eleven lncRNAs were downregulated and PAXIP1-AS1, LINC00618, LINC01605, LINC00648, ANKRD10-IT1, and APCODD1L-AS1 have not been reported in association with GC to date among those genes. Third, we investigated whether the expression of the six downregulated lncRNAs mentioned above was related to overall survival (OS) using Kaplan–Meier curves in GC. Expression profiles describing the lncRNAs were downloaded from UCSC-Xena (https://xenabrowser.net/datapages/). Patients with GC from The Cancer Genome Atlas (TCGA) cohort were divided into two groups with high and low lncRNA expression using the optimal cutoff determined by the X-tile. The results showed that patients with low LINC01605, LINC00648, or APCODD1L-AS1 (Supplementary Fig. 1A–C) expression had better survival than those with high expression; no significant difference was observed in the OS of patients with neither high nor low LINC00618 and ANKRD10-IT1 expression (Supplementary Fig. 1D, E). However, patients with low PAXIP1-AS1 expression had a shorter OS (Supplementary Fig. 1F). Fourth, we assessed the prognostic value of disease-specific survival (DSS). Patients with low PAXIP1-AS1 expression (Supplementary Fig. 1G) had unfavourable DSS outcomes. Therefore, PAXIP1-AS1 was selected as the study object.

Fifth, the forced expression (Fig. 1C1) or knockdown of HOXD9 (Fig. 1C2) verified that HOXD9 expression was negatively correlated with PAXIP1-AS1 expression in AGS and MKN-74 cells. Sixth, we investigated whether HOXD9 directly regulated PAXIP1-AS1 transcription. The PAXIP1-AS1 putative promoter region (http://genome.ucsc.edu/, −1700 ~ 300 bp: 2000 bp) was assessed using the ALGGEN - PROMO program, with the two most probable binding motifs for HOXD9 lying in the −1298 to −1308 (Site 1) and −1503 to −1513 (Site 2) regions (Fig. 1D, E). We then performed chromatin immunoprecipitation (ChIP) analysis on the GC cells using an anti-HOXD9 antibody. PCR amplification showed a band corresponding to 190 bp that included the second possible binding site (−1503 to −1513). Normal rabbit IgG was used as the negative control. No bands were evident in the immunoprecipitate for the first possible binding site (−1298 to −1308) or the control IgG (Fig. 1F). The luciferase reporter assay showed that HOXD9 transrepressed PAXIP1-AS1 promoter activity. Site-directed mutagenesis showed that the second HOXD9-binding site was critical for HOXD9-induced PAXIP1-AS1 transrepression (Fig. 1G).

The collective data suggest that HOXD9 binds to a specific PAXIP1-AS1 promoter to repress its transcription.

### PAXIP1-AS1 functions as a tumour suppressor during GC progression

**Fig. 2:**
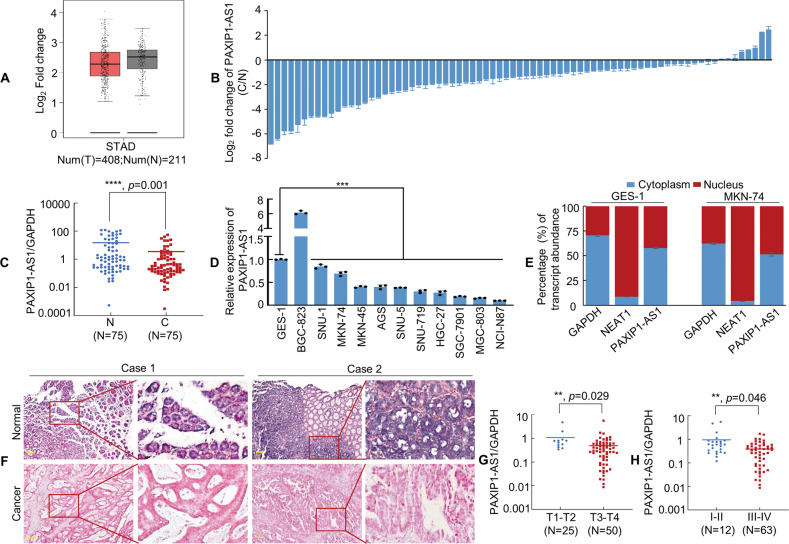
PAXIP1-AS1 expression is downregulated in GC cell lines and tissues. **A** The expression level of PAXIP1-AS1 in gastric tissue was determined from the GEPIA database, using the TCGA tumour matching TCGA and GTEx normal data. **B**, **C** PAXIP1-AS1 expression was detected by qPCR in 75 pairs of human GC and adjacent normal mucosal tissues. Error bars represent the standard deviation (SD) in three independent experiments. ****P < 0.001. N, Normal; C, Cancer. **D** The expression level of PAXIP1-AS1 in immortalised gastric mucosal cell line GES-1 and GC cell lines was obtained using qPCR. One-way analysis of variance (ANOVA) and Dunnett’s T3 multiple comparison test were used to compare the expression difference among GES-1 and all GC cell lines. ***P < 0.01. **E** Subcellular localisation of PAXIP1-AS1, GAPDH, and NEAT1 in GES-1 and MKN-74 cells. GAPDH mRNA and NEAT1 mRNA were used as controls for the cytoplasmic and nuclear fractions, respectively. **F** Representative In situ hybridization (ISH) images of PAXIP1-AS1 expression in normal gastric mucosa and GC tissues. Scale bar, 100 μm. **G** PAXIP1-AS1 expression level was lower in T3–4 than in T1–2 GC samples. **P < 0.05. **H** Expression of PAXIP1-AS1 in different clinical GC stages. **P < 0.05. stages I–II vs. stages III–IV.

PAXIP1-AS1 is located on the chromosomal locus 7q36.2. Online coding ability analysis using the CPAT and CPC databases demonstrated low PAXIP1-AS1 coding potential (Supplementary Fig. 2A, B). We then used the GEPIA database to show that PAXIP1-AS1 expression was lower in tissues from the GC group (*n* = 408) as compared to that from the normal group (*n* = 211), as shown in Fig. 2A. Next, we used qPCR to detect PAXIP1-AS1 expression in 75 pairs of matched normal and cancerous gastric tissues, the results of which indicated that PAXIP1-AS1 was significantly downregulated in GC (Fig. 2B, C). Moreover, PAXIP1-AS1 expression was downregulated in most GC cell lines except BGC-823, compared to that in the normal GES-1 cell line control (Fig. 2D). Importantly, PAXIP1-AS1 expression was found to be negatively correlated with HOXD9 expression (Fig. 1A) in most GC cells.

To determine the subcellular localisation of PAXIP1-AS1 in GC cells, cytoplasmic/nuclear fractionation of GES-1 and MKN-74 cells was performed. The expression of PAXIP1-AS1 was higher in the cytoplasm than in the nucleus (Fig. 2E). We further examined this result by using ISH to investigate 10 pairs of normal and GC tissues and obtained consistent results, as shown in Fig. 2F.

To assess the putative correlations between PAXIP1-AS1 and clinicopathological features, the relative expression of PAXIP1-AS1 in GC tissue samples was measured. PAXIP1-AS1 expression was found to be significantly correlated with tumour invasion (T1–2 vs. T3–4; Fig. 2G, *P* < 0.05), lymph node stage (*P* < 0.05), and the TNM stage (AJCC) (I–II vs. III–IV; Fig. 2H, *P* < 0.05) (Supplementary Table 2). However, no significant association was observed between PAXIP1-AS1 expression and sex, age, tumour size, or tumour differentiation (all *P* > 0.05).

These findings indicate that PAXIP1-AS1 acts as a tumour suppressor in GC progression.

### PAXIP1-AS1 expression suppresses aggressive phenotypes of GC cells in vitro and in vivo

Given the notable decrease in PAXIP1-AS1 expression in GC tissues and cells, the functional effect of PAXIP1-AS1 on cell behaviour was assessed in vitro. First, stable transfectants of PAXIP1-AS1 and control plasmids in AGS and MKN-45 cells, or small interfering RNA (siRNA) knockdown and Scr (scrambled) siRNA in MKN-74 cells were established. To verify the expression levels, PAXIP1-AS1 overexpression or knockdown was confirmed by qPCR in the GC cell lines (Supplementary Fig. 3A).

**Fig. 3:**
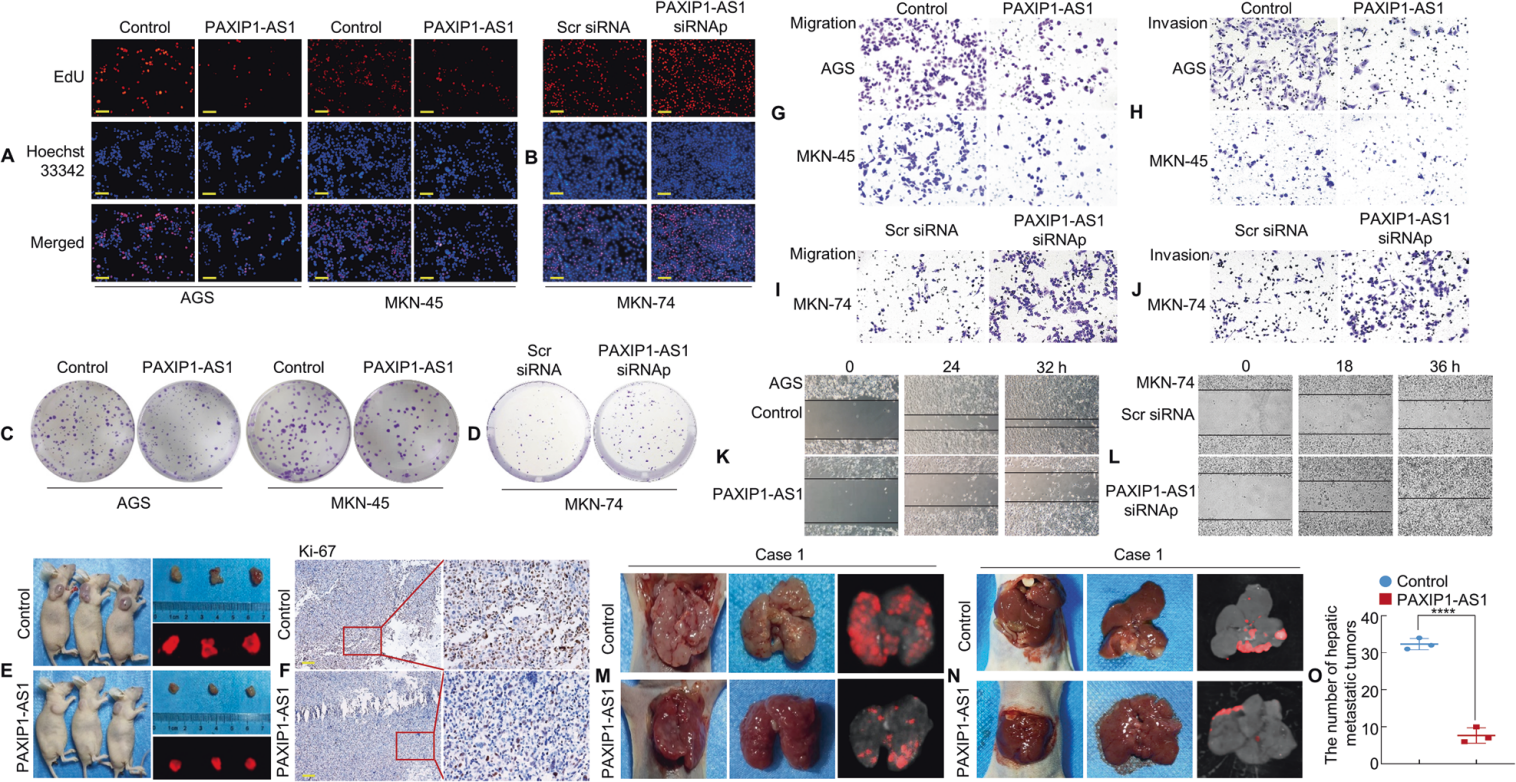
Forced expression of PAXIP1-AS1 inhibits GC cell proliferation and metastasis. **A**, **B** Representative micrographs of EdU positivity in cells transfected with Control and PAXIP1-AS1 (**A**) or Scr siRNA and PAXIP1-AS1 siRNAp (**B**). Red fluorescence represents EdU-positive cells and blue fluorescence from the Hoechst 33342 stain represents total cells. **C**, **D** Representative images of crystal violet stained cell colonies formed by the indicated GC cell, 14 d after inoculation. **E** Gross appearance and external whole-body fluorescence images of subcutaneous tumours from mice administered subcutaneous injection of MKN-74/RFP-Control and MKN-74/RFP-PAXIP1-AS1 cells. **F** The cell proliferation rate of PAXIP1-AS1 overexpressing tumours was significantly lower than that of Control, as observed by immunohistochemistry (IHC) assay with Ki-67. **G**–**J** Migration (**G** and **I**) and invasion (**H** and **J**) assays were conducted using GC cells transfected with PAXIP1-AS1 and Control (**G** and **H**) or PAXIP1-AS1 siRNAp and Scr siRNA (**I** and **J**). **K**, **L** The wound healing assay was used to detect GC cell motility following transfection with PAXIP1-AS1 and Control (**K**) or PAXIP1-AS1 siRNAp and Scr siRNA (**L**). **M** Lung samples were obtained from mice that received intravenous tail injections of MKN-74/PAXIP1-AS1 and Control cells, respectively (*n* = 3). **N** Representative images of livers from intrasplenic liver metastasis model constructed with MKN-74/PAXIP1-AS1 and control cells (*n* = 3). **O** The number of hepatic metastatic tumours of the control and PAXIP1-AS1 groups. Scale bars, 50 μm in A & B and 100 μm in F. For quantification, see Supplementary Figs. 3 & 4.

Subsequently, we tested the rates of cell proliferation by EdU fluorescence staining, which is used to directly measure active DNA synthesis or S-phase synthesis during the cell cycle. The EdU incorporation assay indicated that the average percentage of positive cells was significantly lower in the PAXIP1-AS1-overexpressing groups (Fig. 3A and Supplementary Fig. 3B), and significantly higher in the PAXIP1-AS1 knockdown groups as compared to that in the Control groups (Fig. 3B and Supplementary Fig. 3C). Colony formation assays further demonstrated that PAXIP1-AS1 overexpression resulted in the decreased proliferation of AGS and MKN-45 cells (Fig. 3C and Supplementary Fig. 3D), whereas PAXIP1-AS1 knockdown resulted in increased proliferation of MKN-74 cells (Fig. 3D and Supplementary Fig. 3E).

To verify the growth in vivo, MKN-74 cells containing lenti-RFP-Control or lenti-RFP-PAXIP1-AS1 were injected into BALB/c-nu/nu mice to form subcutaneous xenografts, as shown in Fig. 3E. The mice were euthanised 28 d after injection and the tumours were dissected. The presence of cancerous tissue sections was confirmed by histological analysis (Supplementary Fig. 3F). The tumour volumes associated with PAXIP1-AS1-overexpressing cells were markedly smaller than those associated with the Vector-expressing cells (Supplementary Fig. 3G). Next, we examined the expression of the cell proliferation marker Ki-67 in xenograft tumours. IHC showed significantly decreased proliferation rates in the PAXIP1-AS1-overexpressing group compared to those in the Control group (Fig. 3F and Supplementary Fig. 3H). These findings indicate that the forced expression of PAXIP1-AS1 resulted in decreased growth of the GC cells, both in vitro and in vivo.

To investigate the effects of PAXIP1-AS1 on the invasion and migration of cancer cells, Transwell migration and wound healing assays were performed in vitro. Transwell assays with or without Matrigel demonstrated lower migratory and invasive activity under the forced expression of PAXIP1-AS1 in AGS and MKN-45 cells than in the control cells. However, MKN-74 cells subjected to PAXIP1-AS1 knockdown showed higher migratory and invasive activity than control cells (Fig. 3G–J and Supplementary Fig. 4A–D). The wound healing assay showed that PAXIP1-AS1 overexpression in GC cells was associated with significantly slower wound closure, and vice versa (Fig. 3K, L and Supplementary Fig. 4E–G).

Next, we constructed tail vein-lung and intrasplenic liver metastatic models to test the effect of PAXIP1-AS1 on GC metastasis in vivo. MKN-74/PAXIP1-AS1 and Control cells were injected into the tail vein of nude mice (Fig. 3M and Supplementary Fig. 4H). The results indicated that MKN-74/PAXIP1-AS1 cells formed fewer metastatic nodules in the lungs than MKN-74/Control cells 28 d after implantation (Supplementary Fig. 4I). Histological analysis of HE-stained lung sections verified the presence of lung metastases (Supplementary Fig. 4J). Expression of the EMT markers E-cadherin and MMP2 were confirmed by IHC. E-cadherin expression was lower, whereas MMP2 expression was higher in the cancer tissues than in the normal tissues (Supplementary Fig. 4K, L). Besides, MKN-74/PAXIP1-AS1 and Control cells were injected into the spleen of mice and their livers were dissected three weeks later. Fewer metastatic tumours were observed in the PAXIP1-AS1 group compared with the Control group (Fig. 3N, O and Supplementary Fig. 4M). HE staining was used to visualise the tumours in the mouse livers (Supplementary Fig. 4N).

Collectively, our data demonstrate that PAXIP1-AS1 can inhibit tumour proliferation and metastasis.

### Overexpression of PAXIP1-AS1 significantly attenuates HOXD9-enhanced EMT and invasion-metastasis in GC cells

**Fig. 4:**
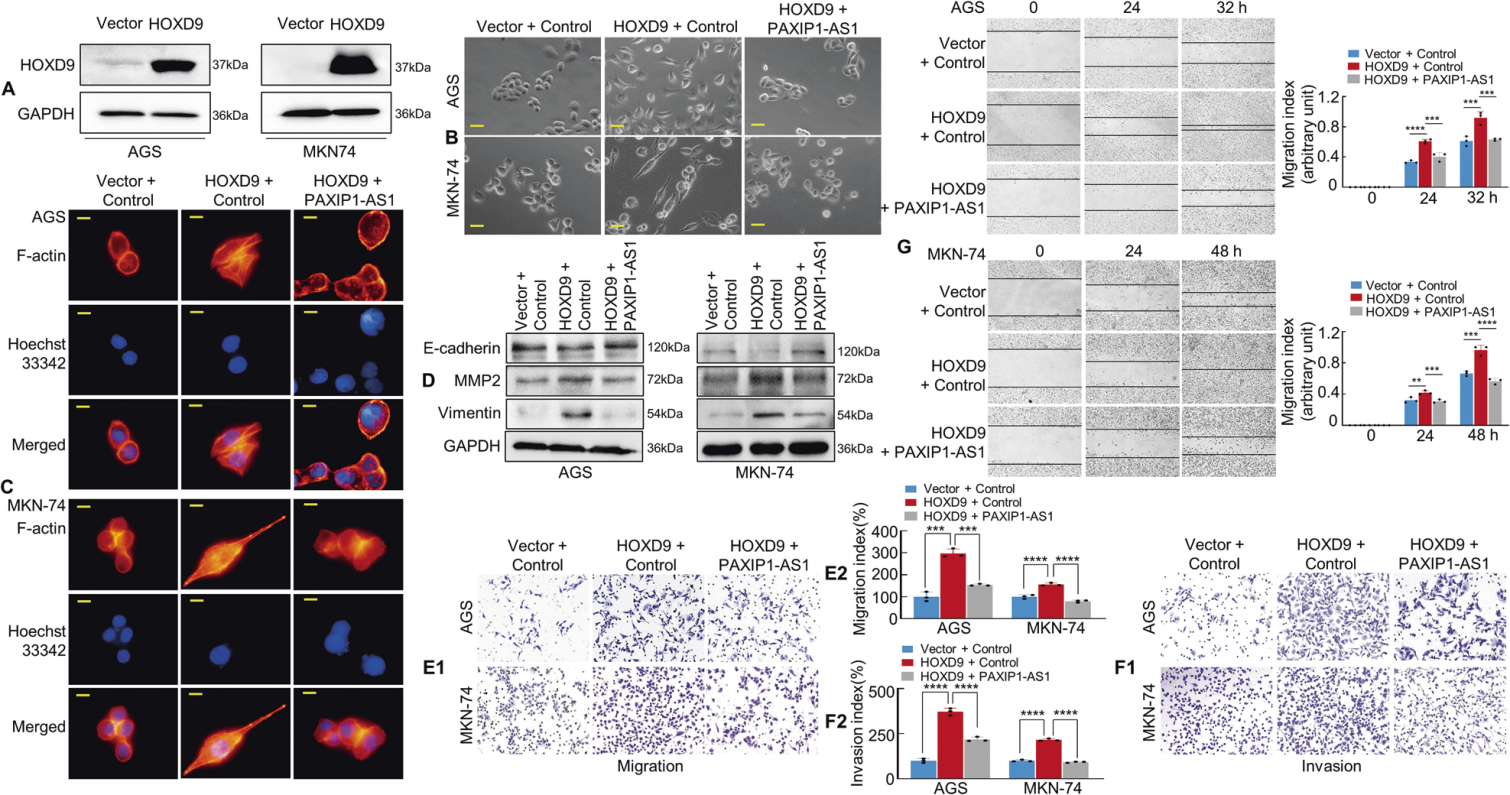
HOXD9-PAXIP1-AS1 axis regulates the migration and invasion of GC cells in vitro. **A** The efficiency of HOXD9 plasmid transfection was detected by western blotting assay. **B** The morphology of GC cells was observed under phase-contrast microscopy. **C** Transfected AGS and MKN-74 cells were stained with rhodamine-phallotoxin to identify F-actin filaments under the fluorescent microscope. All experiments were repeated three times. **D** EMT biomarkers, including E-cadherin, MMP2, and Vimentin were detected by western blotting 48 h after transfection. **E**, **F** Transwell migration (**E1**) and invasion (**F1**) assay were performed on GC cells. Quantification of migration and invasion capabilities of GC cells are shown (**E2**, **F2**). ***P < 0.01, ****P < 0.001. **G** The wound healing assay was used to detect cell motility of AGS and MKN-74 cells following transfection. The migration index is shown in the right panel. **P < 0.01, ***P < 0.01, ****P < 0.001. Scale bars, 25 μm in (**B**) and 10 μm in (**C**).

Studies by various researchers, including our group, have reported that HOXD9 promotes tumour metastasis via EMT alterations [6, 21]. After confirming the efficiency of HOXD9 overexpression by western blotting (Fig. 4A), we determined whether PAXIP1-AS1 was involved in HOXD9-mediated tumour cell migration, invasion, and EMT. We observed that forced HOXD9 transfectants exhibited spindle-like fibroblastic morphology, which is one of the main characteristics of EMT, whereas Vector transfectants displayed round or flat morphology with a short cytoplasmic process. Moreover, co-transfection of HOXD9 and PAXIP1-AS1 in GC cells led to the reversal of HOXD9-induced EMT enhancement under a phase-contrast microscope (Fig. 4B).

Previous reports have indicated that EMT enhances cell motility and actin reorganisation [22–24]. We, therefore, investigated this by staining F-actin using phalloidin. In contrast to the Vector+Control group, the HOXD9+Control group showed a highly structured array of thick F-actin filaments in the bulk cytoplasm. However, the forced expression of HOXD9 and PAXIP1-AS1 in GC cells led to partial depolymerisation of the thick F-actin filaments compared to those in HOXD9+Control group (Fig. 4C), indicating an opposite phenotype to that induced by the EMT.

We further found that exogenous HOXD9 expression upregulated MMP2 and Vimentin and downregulated E-cadherin compared to the Vector cell group. However, western blotting indicated that the cell groups with HOXD9 and PAXIP1-AS1 overexpression showed decreased MMP2 and Vimentin expression and increased E-cadherin expression compared with the HOXD9+Control group (Fig. 4D).

To further explore the role of PAXIP1-AS1 and HOXD9 in GC cell progression, Transwell assays with or without Matrigel and wound healing assays were performed in vitro. Forced HOXD9 expression was found to increase the invasion and migration potential of AGS and MKN-74 cells compared to that of the Vector cells; however, plasmids comprising HOXD9 and PAXIP1-AS1 decreased the invasion and migration potential of GC cells compared to those in HOXD9+Control group (Fig. 4E–G).

These findings indicate that the HOXD9-PAXIP1-AS1 axis modulates EMT, migration, and invasion of GC cells.

### PAXIP1-AS1 is associated with PABPC1 protein in GC cells

**Fig. 5:**
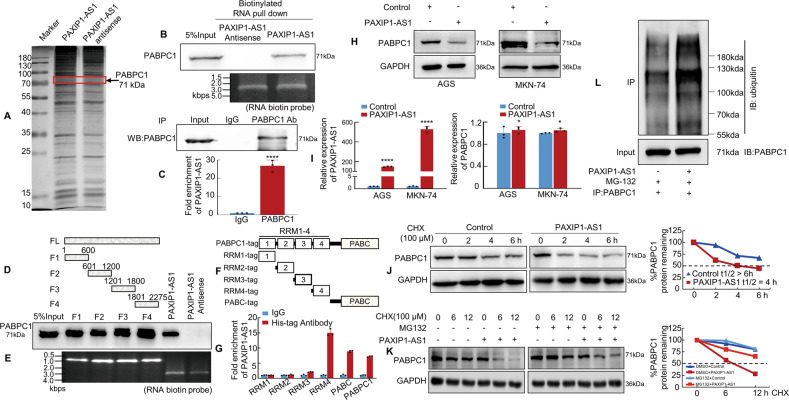
PAXIP1-AS1 binds to Polyadenylate-binding protein cytoplasmic 1 (PABPC1) in human GC cells. **A** Silver staining of biotinylated PAXIP1-AS1-associated proteins. **B** Western blotting of proteins from PAXIP1-AS1 and its antisense pull-down assays; *n* = 3. **C** The association of endogenous PABPC1 and PAXIP1-AS1 in MKN-74 cells was detected by RNA immunoprecipitation (RIP) assay using anti-PABPC1 antibodies. Relative enrichment (mean ± SD) of PAXIP1-AS1 with anti-PABPC1 or anti-IgG was measured by qPCR. **D**, **E** Western blotting of PABPC1 in samples pulled down by full-length or truncated PAXIP1-AS1 (F1: 1–600, F3: 601–1200, F3: 1201–1800, F4: 1801–2275); *n* = 3. **F**, **G** Full-length and truncated PABPC1 plasmids with 6× His-tag were transfected into MKN-74 cells for 48 h and extracted for RIP assays with anti-His antibody. Results showed that PAXIP1-AS1 mainly bound to the RRM4 region and PABC domain in full-length PABPC1. **H** Western blotting analysis of PABPC1 in Control and PAXIP1-AS1-overexpressed GC cells. **I** Total RNA from Control or PAXIP1-AS1-overexpressing AGS and MKN-74 cells was analysed by qPCR to validate PAXIP1-AS1 expression (the left panel) and determine PABPC1 mRNA levels (the right panel). ****P < 0.001 and *P > 0.05, Control vs. PAXIP1-AS1. *n* = 3. **J** Control or PAXIP1-AS1-overexpressed MKN-74 cells were treated with 100 μM CHX for indicated periods. Subsequently, cell lysates were analysed by western blotting with anti-PABPC1 and anti-GAPDH antibodies. **K** PAXIP1-AS1 or control plasmid was transfected into MKN-74 cells for 48 h and cells were treated with DMSO, CHX (100 μM), or CHX plus MG132 (10 nM) for 0, 6, or 12 h before protein extraction. Subsequently, cell lysates were analysed by western blotting with anti-PABPC1 and anti-GAPDH antibodies. **L** PAXIP1-AS1-overexpressed or control plasmid was transfected into MKN-74 cells for 48 h and cells were treated with 10 nM MG132 for 6 h. Extracts were immunoprecipitated with anti-PABPC1 antibody, and PABPC1 polyubiquitination was examined by western blotting using anti-ubiquitin antibody.

Recent studies showed that lncRNA could interact with multiple protein partners to promote tumour development and progression [25, 26]. We performed RNA pull-down assays and subsequent mass spectrometry (MS) analysis to explore the proteins potentially associated with PAXIP1-AS1. Proteins were identified using SDS-PAGE and silver staining (Fig. 5A) and were concurrently subjected to MS; through this analysis, 312 potential binding proteins were identified (Supplementary Table 3). These binding proteins included PABPC1, a PABP family member that interacts with the 3′-UTR of various genes [27]. We then searched the RBPDB database (http://rbpdb.ccbr.utoronto.ca/) and found that the PAXIP1-AS1 might be associated with PABPC1 (Supplementary Fig. 5A). Further validation indicated that PABPC1 was readily detected in the PAXIP1-AS1 pull-down complex, but not in the control samples that were pulled down using the PAXIP1-AS1 antisense transcript (Fig. 5B). We also performed RIP to verify the candidate proteins pulled down by PAXIP1-AS1 using antibodies against PABPC1, with a non-specific antibody (IgG) as control. The results showed that PABPC1 bound directly to PAXIP1-AS1 in MKN-74 cells (Fig. 5C).

To identify the binding sites, we took advantage of a series of deletion PAXIP1-AS1 mutants to map the PABPC1-binding region; F1 corresponds to the region between nucleotides 1 and 600 of PAXIP1-AS1, which is the truncated 5′-end; F2 corresponds to the region between nucleotides 601 and 1200; F3 corresponds to the region between nucleotides 1201 and 1800; and F4 corresponds to the region between nucleotides 1801 and 2275, which is at the truncated 3′-end (Fig. 5D). The results of the in vitro binding assays indicated that PABPC1 interacted with either the F1–F4 region or the full-length PAXIP1-AS1 (Fig. 5E).

We next sought to determine the PAXIP1-AS1-binding region or sites within the PABPC1 protein. The PABPC1 protein consists of five main domains, four RNA recognition motif (RRM) domains and a PABC domain. To further identify the PABPC1 sites that interact with PAXIP1-AS1, full-length PABPC1 with His-tag was constructed along with five deletion mutants. (Fig. 5F and Supplementary Fig. 5B). The results showed that in addition to full-length (FL) PABPC1, mutants that included the RRM4 and PABC domain could pull down PAXIP1-AS1 (Fig. 5G), indicating that these domains might serve as important PAXIP1-AS1 interaction sites.

Since many lncRNAs have been shown to regulate the stability of binding proteins [28–30], we further explored whether the binding of PAXIP1-AS1 to PABPC1 affected protein stability. We showed that exogenous PAXIP1-AS1 expression led to a decrease in the expression of PABPC1 at the protein level, but its mRNA level remained unaffected (Fig. 5H, I). Next, PAXIP1-AS1-overexpressed and Control cells were treated with cycloheximide (CHX) to block protein synthesis and PABPC1 expression at the protein level was measured. The results showed that PAXIP1-AS1 promoted degradation of the PABPC1 protein (Fig. 5J). To clarify the mechanism underlying the PAXIP1-AS1-mediated degradation of PABPC1, MKN-74 cells were treated with MG132 to block proteasome-based protein degradation and western blotting was performed to determine the difference in the PAXIP1-AS1 and control group expression cells. The results suggested that the proteasome inhibitor MG132 significantly attenuated the degradation of the PABPC1 protein resulting from PAXIP1-AS1 overexpression (Fig. 5K). In addition, ubiquitination assays were conducted to confirm that PAXIP1-AS1 promoted the ubiquitination of PABPC1 (Fig. 5L).

The results of these experiments indicate that PAXIP1-AS1 may bind directly to the PABPC1 protein and promote its degradation via ubiquitination, thereby regulating its expression.

### PAXIP1-AS1 interacts with PABPC1 to regulate metastases in GC cells

**Fig. 6:**
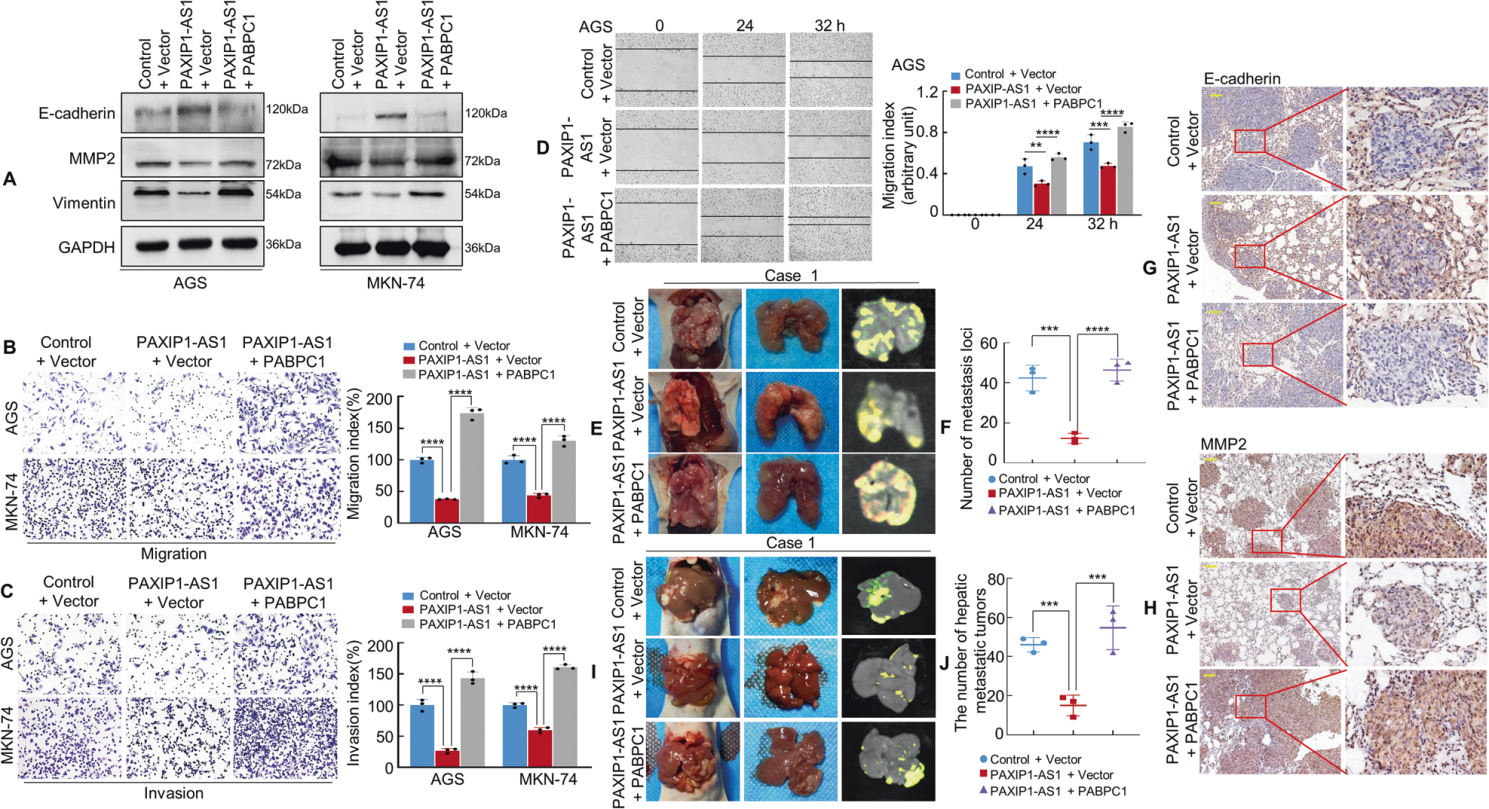
PAXIP1-AS1 with PABPC1 regulates migration and invasion of GC cells. **A** The expression of EMT biomarkers, including E-cadherin, MMP2, and Vimentin was detected by western blotting 48 h following transfection. **B**, **C** GC cells were transfected and subjected to Transwell migration and invasion assay. Quantification of migration and invasion capabilities of GC cells are shown in the right panel. ****P < 0.001. **D** The mobility of transfected AGS cells was detected by the wound healing assay and the migration index is shown in the right panel. **P < 0.05, ***P < 0.01, ****P < 0.001. **E** Representative images of metastatic tumours in the lungs from mice in the Control + Vector, PAXIP1-AS1 + Vector, and PAXIP1-AS1 + PABPC1 groups are shown, respectively (*n* = 3 in each group). **F** The number of metastatic tumours in the lung was counted. ***P < 0.001, ****P < 0.001. **G**, **H** IHC staining of E-cadherin and MMP2 expressions. **I**, **J** Representative images (**I**) and qualification (**J**) of hepatic metastatic tumours in the indicated groups (*n* = 3). ***P < 0.01. Scale bar, 100 μm in (**G**) and (**H**).

To determine whether the interaction between PAXIP1-AS1 and PABPC1 was functional, a PABPC1 plasmid was used to induce PABPC1 expression in GC cells (Supplementary Fig. 6A). We then transiently co-transfected the GC cells with empty Control + Vector, PAXIP1-AS1 + Vector, or PAXIP1-AS1 + PABPC1 plasmids. The results showed that the overexpression of PAXIP1-AS1 in GC cells increased the expression of epithelial markers (E-cadherin) while decreasing the expression of mesenchymal markers (Vimentin and MMP2) relative to the Control cells; moreover, forced expression of PAXIP1-AS1 and PABPC1 reversed the PAXIP1-AS1-induced suppression of EMT in GC cells, as analysed by western blotting (Fig. 6A).

PAXIP1-AS1 was also found to significantly decrease the number of and rate at which invading cells migrated compared to those of the Control cells, whereas co-transfection with PAXIP1-AS1 and PABPC1 was found to reverse the inhibitory effect on invasion and migration (Fig. 6B–D and Supplementary Fig. 6B) in AGS and MKN-74 cells.

To determine whether PAXIP1-AS1 regulated PABPC1 expression in vivo, MKN-74 cells expressing the Lentivirus (LV)-Control + Vector, LV-PAXIP1-AS1 + Vector, or LV-PAXIP1-AS1 + PABPC1 were implanted into the tail vein of nude mice (Fig. 6E and Supplementary Fig. 6C). The presence of GC metastasis in the lungs was confirmed by histological analysis (Supplementary Fig. 6D). Notably, compared to the Control + Vector group, intravenous inoculation with LV-PAXIP1-AS1 + Vector cells led to a significant reduction in the number of visible tumours in the lungs, which correlated with a lower number of metastatic loci. In contrast, an increase in the number of metastatic loci was observed in the PAXIP1-AS1 + PABPC1 group (Fig. 6F). IHC staining with anti-E-cadherin and anti-MMP2 antibodies further verified the presence of GC metastases in the lungs of nude mice (Fig. 6G, H). Besides, the mice that underwent injection of the corresponding groups of cells mentioned above were sacrificed three weeks later. We found that fewer metastatic tumours in livers were observed in the PAXIP1-AS1 + Vector group compared with the Control + Vector one. In contrast, PAXIP1-AS1 + PABPC1 reversed the effects observed in PAXIP1-AS1 + Vector group (Fig. 6I, J and Supplementary Fig. 6E). Pathological analysis was performed using HE staining (Supplementary Fig. 6F).

Moreover, we found that interference with PAXIP1-AS1 led to the promotion of EMT induction as well as the invasive and migratory ability of GC cells, which could be restored after interfering with the expression of both PAXIP1-AS1 and PABPC1(Supplementary Fig. 7A–E).

These data indicate that PAXIP1-AS1 cooperates with PABPC1 to regulate GC cell migration and EMT.

### PABPC1 promotes GC metastasis by enhancing PAK1 mRNA stability

**Fig. 7:**
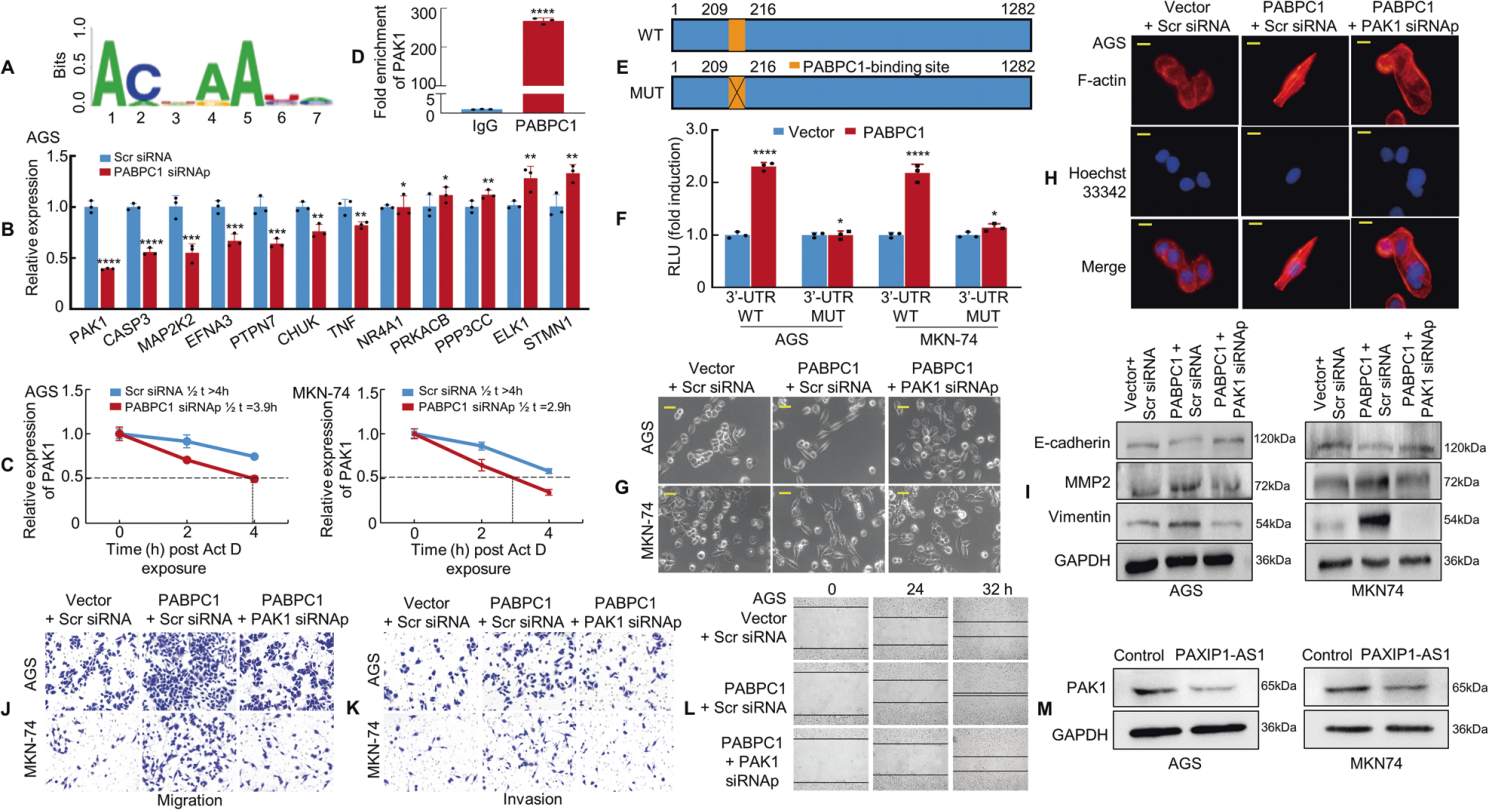
PABPC1 promotes GC metastasis by enhancing PAK1 mRNA stability. **A** PABPC1-binding motif predicted by bioinformatics analysis. **B** The relative expression of 12 genes in PABPC1 knockdown AGS cells was detected by qPCR. The experiment was performed in triplicate. *P > 0.05; **P < 0.05; ***P < 0.01; ****P < 0.001. Scr siRNA vs. PABPC1 siRNAp. **C** Actinomycin D (10 μg/mL) was applied to GC cells transfected with PABPC1 siRNAp or Scr siRNA. RNA was extracted at indicated time points to detect the mRNA level of PAK1. **D** RIP assay in MKN-74 cells showed that PABPC1 bound directly to PAK1 mRNA; anti-IgG was used as a negative control. ****P < 0.001. IgG vs. PABPC1. **E** The PAK1 3′UTR region is shown in the blue box. The orange box represents a PABPC1 potential binding site(WT, upper panel) and the orange box with a cross represents the corresponding mutation site(MUT, lower panel). **F** PABPC1 overexpression enhanced luciferase activity in GC cells transfected with the PAK1 3′UTR wild-type reporter construct as compared to those transfected with the mutant reporter construct. *P > 0.05; ****P < 0.001. Vector vs. PABPC1. **G** The morphology of transfected AGS and MKN-74 cells was observed under phase-contrast microscopy. **H** Transfected AGS cells were stained with rhodamine-phallotoxin to visualise F-actin filaments under the fluorescence microscope. **I** PAK1 knockdown decreased the expression of mesenchymal markers and restored the expression of epithelial markers in PABPC1 overexpressed GC cells. **J**, **K** GC cells were transfected and subjected to Transwell migration and invasion assays. **L** The wound healing assay was used to detect GC cell motility. **M** Control and PAXIP1-AS1 were transfected into GC cells. PAK1 expression and GAPDH were detected in GC cells using western blotting. Scale bars, 25 μm in (**G**) and 10 μm in (**H**). For quantification, see Supplementary Fig. 8.

PABPC1 is important for protein translation initiation and decay and regulates mRNAs by binding to their poly(A) tails or 5′‐ACUAAUC‐3′ motifs. Thus, we used the Atlas of UTR Regulatory Activity (AURA) database (http://aura.science.unitn.it/) to predict the binding motif of PABPC1 (Fig. 7A) and to determine the potential mRNAs to which PABPC1 might bind. Two hundred and ninety-two 3′-UTR mRNAs (Supplementary Table 4) were predicted and input into the STRING database for the Kyoto Encyclopedia of Genes and Genomes (KEGG) pathway analysis. Thirty-six significantly enriched KEGG pathways were identified, including the MAPK signalling pathway (Supplementary Table 5). Twelve components were included in the MAPK signalling pathway, and of these, EFNA3, STMN1, PAK1, and TNF have previously been found to regulate EMT in cancer progression [31–33].

Subsequently, we used siRNAs to knock down PABPC1 and verified the efficiency in GC cells (Supplementary Fig. 8A). The expression of the 12 related genes was measured by qPCR in PABPC1 knockdown and control GC cells. The results showed that PAK1 mRNA displayed the most significant change in expression in the GC cells subjected to PABPC1 knockdown (Fig. 7B and Supplementary Fig. 8B). The impact of PABPC1 on the stability of PAK1 mRNA was measured using Actinomycin D chase analysis. A shorter half-life for PAK1 mRNA was observed in GC cells with PABPC1 knockdown, indicating that PABPC1 enhances the stability of PAK1 mRNA in a post-transcriptional manner (Fig. 7C).

We then performed the RIP assay in MKN-74 cells and found high enrichment of PAK1 mRNA in the PABPC1-RNA binding complex with anti-IgG as a negative control (Fig. 7D). We next showed that PABPC1 might bind to the PAK1 mRNA 3′-UTR at the 5′-ACUAAUC-3′ sequence using the RBPDB database (Supplementary Fig. 8C). A dual-luciferase assay using reporters that carried the entire region of the wild-type PAK1 3′-UTR or PABPC1-binding site (210–216 nucleotides) mutant PAK1 3′-UTR was also performed (Fig. 7E). Overexpression of PABPC1 boosted luciferase activity in GC cells transfected with wild-type PAK1 3′-UTR, however, mutated PAK1 3′-UTR had no significant effect (Fig. 7F). The results show that PABPC1 could bind to PAK1 3′-UTR, thereby increasing its stability.

Given that PAK1 is a critical downstream effector of PABPC1, we attempted to characterise the functional role of PAK1 in the context of EMT and metastasis of GC cells. First, the effect of siRNA-mediated PAK1 knockdown was verified in AGS and MKN-74 cells (Supplementary Fig. 8D). The forced expression of PABPC1 was found to promote EMT and enhance cell invasion and migration; however, depleted PAK1 in the background of PABPC1 transfection in cells was found to reverse PABPC1-induced GC invasion and metastasis through EMT (Fig. 7G–L, Supplementary Fig. 8E–I).

The relationship among PAXIP1-AS1, PABPC1, and PAK1 expression was also examined using western blotting and the result showed that PAXIP1-AS1 upregulation significantly decreased the expression of PAK1 (Fig. 7M), suggesting that PAXIP1-AS1 is involved in regulating PABPC1 and PAK1 expression in GC cells. Besides, Actinomycin D chase analysis indicated that overexpression of PAXIP1-AS1 could reduce the half-life of PAK1 mRNA as well (Supplementary Fig. 9).

These results indicate that PABPC1 promotes metastasis and EMT by enhancing the stability of PAK1 mRNA in GC cells.

### The expression of PAXIP1-AS1 is negatively correlated with HOXD9, PABPC1, and PAK1 expression in human gastric samples

**Fig. 8:**
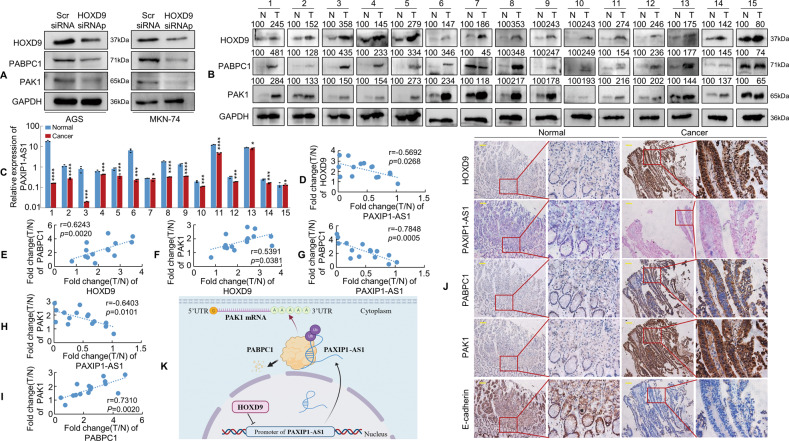
PAXIP1-AS1 expression correlates inversely with HOXD9, PABPC1, and PAK1 expression in GC tissues. **A** Scr siRNA and HOXD9 siRNAs were transfected into GC cells. The expression of HOXD9, PABPC1 and PAK1 was detected in GC cells using western blotting analysis. **B**, **C** Expression levels of HOXD9, PABPC1, PAK1, and PAXIP1-AS1 in 15 paired normal and GC tissue specimens were detected by western blotting (**B**) or qPCR (**C**) and normalised to GAPDH. N, normal; T, cancer. Student’s *t*-test; *P > 0.05; ***P < 0.01 and ****P < 0.001, normal vs. cancer. **D**–**I** Pearson correlation analysis was used to identify expression correlations between HOXD9 and PAXIP1-AS1 (**D**), HOXD9 and PABPC1 (**E**), HOXD9 and PAK1 (**F**), PAXIP1-AS1 and PABPC1 (**G**), PAXIP1-AS1 and PAK1 (**H**), and PABPC1 and PAK1 (**I**) using the expression data above. **J** The expression of indicated genes in normal and GC tissues was evaluated using IHC and ISH. Scale bar, 100 μm. **K** The hypothetical model depicts the roles of HOXD9, PAXIP1-AS1, PABPC1, and PAK1 in GC cells.

We demonstrated a negative correlation between HOXD9 and PAXIP1-AS1 expression in GC cells (Fig. 1C). Further experiments were thus performed to determine whether HOXD9 modulated PABPC1 and PAK1 expression in AGS and MKN-74 cells. The results indicated that HOXD9 knockdown significantly decreased PABPC1 and PAK1 expression (Fig. 8A). We then analysed the expression of HOXD9, PAXIP1-AS1, PABPC1, and PAK1 in 15 pairs of GC and normal mucosal biopsies. Western blotting and qPCR results indicated that HOXD9, PABPC1, and PAK1 were usually upregulated, whereas PAXIP1-AS1 was frequently downregulated in the 15 tumour samples examined compared to that in the paired paracancerous tissues from the same patient (Fig. 8B, C). Pearson correlation analysis showed negative correlations between PAXIP1-AS1 and HOXD9 (Fig. 8D), PAXIP1-AS1 and PABPC1 (Fig. 8G), and PAXIP1-AS1 and PAK1 (Fig. 8H) and positive correlations between PABPC1 and HOXD9 (Fig. 8E), PAK1 and HOXD9 (Fig. 8F), and PAK1 and PABPC1 (Fig. 8I) in GC tissues. IHC and ISH showed that the expression of HOXD9, PABPC1 and PAK1 was significantly higher, whereas that of PAXIP1-AS1 was remarkably low in GC as compared to that in normal tissues (Fig. 8J).

Taken together, these results suggest that the expression of PAXIP1-AS1 is negatively associated with that of HOXD9, PABPC1, and PAK1 in GC samples.

## Discussion

In this study, we found that PAXIP1-AS1, a direct transcriptional target of HOXD9, was markedly downregulated in GC tissues. Moreover, low PAXIP1-AS1 expression was associated with the malignant progression of GC cells. PAXIP1-AS1 overexpression was found to inhibit cell proliferation and migration or invasion both in vitro and in vivo. Furthermore, PAXIP1-AS1 was found to interact with the RNA‐binding protein PABPC1, which modulates EMT and metastasis in GC cells. PABPC1 was found to promote GC metastasis by stabilising PAK1 mRNA. These results suggest that the HOXD9/PAXIP1-AS1/PABPC1/PAK1 signalling axis (Fig. 8K) may regulate the development and progression of GC.

PAXIP1-AS1 is a relatively novel lncRNA that plays a significant role in the development and progression of several cancers [11, 12]. Previous studies have reported that PAXIP1-AS1 overexpression modulates the migration, invasion, and angiogenesis of human umbilical vein endothelial cells in glioma [11] and that PAXIP1-AS1 knockdown regulates cell proliferation, accelerates apoptosis, and modulates cell migration and EMT in ovarian cancer [12]. However, the role of PAXIP1-AS1 in GC remains unclear. Here, we demonstrated that PAXIP1-AS1 was markedly downregulated in GC cell lines. Moreover, analysis of TCGA data indicated that low PAXIP1-AS1 expression was significantly correlated with poor OS and DSS in patients with GC. Functional experiments showed that PAXIP1-AS1 inhibited GC cell growth and metastasis/invasion, both in vitro and in vivo. Thus, PAXIP1-AS1 is a tumour suppressor that inhibits the proliferation, migration, and invasion of GC cells.

Several recent studies have shown that lncRNA transcription may be regulated by transcription factors and epigenetic regulators [25, 26, 34], in a manner similar to that observed in the case of protein-coding genes. For example, the transcription factor upstream stimulatory factor 1 (USF1) promotes glioma cell invasion and migration by activating the lncRNA hyaluronan synthase 2-AS1 (HAS2-AS1) [34]. Signal transducer and activator of transcription (STAT1)-induced upregulation of the lncRNA LINC01123 is associated with poor prognosis and promotes endometrial cancer progression [35]. Studies have demonstrated that HOXD9 is a sequence-specific DNA-binding protein that interacts with the promoter regions of target protein-coding genes and regulates transcription [3, 6]. However, it remains unclear whether HOXD9 regulates lncRNA expression. Using ChIP and luciferase assays, we verified that HOXD9 could bind to the PAXIP1-AS1 promoter region to repress its transcription. Moreover, HOXD9 overexpression resulted in enhanced EMT and migration or invasion abilities, which could be reversed to a certain extent by forced expression of PAXIP1-AS1. These results suggest that the HOXD9-PAXIP1-AS1 axis modulates EMT, migration, and invasion of GC cells.

PABPC1 is an RNA-binding protein located in chromosome region 8q22.3 and is expressed in most eukaryotes. PABPC1 is important for protein translation initiation and decay [16, 17]. Abnormally expressed PABPC1 has been found in many human malignancies, including esophageal squamous cell carcinoma [36], hepatocellular carcinoma [37], and GC [38]. Studies have reported that the lncRNA BDNF-AS interacts with PABPC1 to repress the proliferation, migration, and invasion of glioma cells [39] and the lncRNA PAGBC synergizes with PABPC1 to promote the development and progression of gallbladder cancer [40]. Consistently, we discovered that PAXIP1-AS1 might directly bind to PABPC1 protein using the RNA pull-down, MS and RIP assay. Furthermore, protein stability and degradation assays showed that PAXIP1-AS1 could promote PABPC1 degradation via ubiquitination. More importantly, we used functional assay and found that PAXIP1-AS1 attenuated EMT and the motility of GC cells, whereas PABPC1 reversed PAXIP1-AS1-induced EMT inhibition and the invasive and migratory abilities of GC cells. Therefore, we speculate that PAXIP1-AS1 and PABPC1 mutually modulate GC cell motility.

PABPC1 normally functions in the cytoplasm, where it binds to the 3′-UTR of target mRNAs, regulating their stability by either antagonizing or enhancing the activity of cytoplasmic deadenylases [16, 17, 41, 42]. For example, PABPC1 binds directly to the 3′-UTR of β-globin mRNA, which increases the stability of β-globin mRNA [41]. β-casein mRNA is protected from degradation by virtue of the structural interaction between 3′-UTR and poly(A) tail via a protein complex comprising HuR and PABPC1 [42]. We found that PABPC1 potentially bound to the 3′UTR of PAK1 mRNA using bioinformatic analysis (http://aura.science.unitn.it/). Therefore, PAK1 was selected for further study.

PAK1, a main downstream effector of small Rho GTPases, has been reported to induce the proliferation, motility, and invasion of human cancer cells, including colorectal [43], bladder [44], pancreatic [45], and gastric cancers [46]. Importantly, PAK1 acts as a potential EMT inducer by stimulating Vimentin expression, thereby inducing E-cadherin loss in lung cancer cells [47]. Nevertheless, the exact molecular mechanism by which PABPC1 regulates PAK1 expression to promote GC progression has not been investigated. Here, we identified PABPC1 as a direct target of PAK1. The conclusions are based on the following observations. First, RNA stability analysis demonstrated that PABPC1 positively regulates PAK1 mRNA stability. Second, the RIP assay showed that PABPC1 directly interacted with PAK1 mRNA. Third, a dual-luciferase assay using full-length wild-type or mutant PAK1 3′-UTR plasmids confirmed that PABPC1 upregulated PAK1 by binding to a certain motif in the 3′-UTR of PAK1 mRNA. Fourth, functional experiments revealed that PAK1 knockdown inhibited the EMT ability and metastatic potential induced by PABPC1 overexpression in GC cells. These results suggest that PABPC1 facilitates the stability of PAK1 mRNA post-transcriptionally and plays a role in promoting the EMT-induced metastasis of GC cells.

In summary, this study is the first to characterise the frequent downregulation of PAXIP1-AS1 in GC and demonstrate the antitumour function of PAXIP1-AS1 in the context of malignant phenotypes of GC cells. Moreover, PAXIP1-AS1 is directly and transcriptionally repressed by HOXD9 and attenuates HOXD9-enhanced EMT, invasion, and metastasis in GC cells. PAXIP1-AS1 interacts with PABPC1 and facilitates its degradation to regulate GC cell migration and EMT. In addition, PABPC1 promotes EMT and metastasis in GC by enhancing the stability of PAK1 mRNA. Our study highlights the importance of the HOXD9/PAXIP1-AS1/PABPC1/PAK1 signalling axis in GC metastasis and provides insights into diagnostic markers and therapeutic targets that could aid in GC treatment.

## Materials and methods

### Cell lines, cell culture and antibodies

Human GC cell lines AGS, NCI-N87, SNU-719, MKN-45, MKN-74, SNU-1, SNU-5, HGC-27, BGC-823, MGC-803, and SGC-7901, and normal human gastric mucosal cell line GES-1 were obtained from the American Type Culture Collection (ATCC). Cells were grown in basic Roswell Park memorial institute (RPMI-1640) medium (1×) or Dulbecco’s Modified Eagle Medium (DMEM; Thermo Fisher Scientific, Waltham, MA, USA) supplemented with 10% fetal bovine serum (FBS; Biological Industries, Kibbutz Beit-Haemek, Israel), penicillin (100 µg/mL) and streptomycin (100 µg/mL) (Sigma-Aldrich, St. Louis, MO, USA) at 37 °C with 5% CO_2_. The antibodies used are listed in Supplementary Table 6.

### Tissue samples from patients

A total of 75 human GC samples were obtained with the corresponding paracancerous tissue (at least 5 cm from the margin of the tumour) from patients who underwent gastrectomy between July 2017 and April 2019 at Nanfang Hospital, Southern Medical University, Guangzhou, China. Tissue specimens were collected only from patients who had undergone standard surgical treatment and histopathological examinations and had not been treated with radiotherapy, chemotherapy, or molecular-targeted therapy before gastrectomy. This study was approved by the Ethics Committee of the Nanfang Hospital, Southern Medical University.

### Western blotting

Proteins were resolved by sodium dodecyl sulphate poly acrylamide gel electrophoresis (SDS-PAGE) and transferred onto polyvinylidene difluoride membranes (Merck Millipore, Billerica, MA, USA). Primary antibody incubations were carried out overnight at 4 °C and were followed by incubation with the respective secondary antibodies. GAPDH was used as a loading control. The membranes were washed three times with Tris-buffered saline containing 0.1% Tween 20 detergent (TBST) buffer and visualised by chemiluminescence (Beyotime, Beijing, China).

### RNA sequencing

MKN-74 cells were transfected with lentivirus expressing HOXD9 and Vector from Genechem (Shanghai, China) using the Ubi-MCS-3×FLAG-CBh-gcGFP-IRES-puromycin vector. Stably transfected cell lines were established after being treated with puromycin for three weeks, according to the manufacturer’s instructions and used for RNA sequencing. Sequencing was performed using HiSeq 4000 system (Illumina, San Diego, CA, USA). Solexa pipeline version 1.8 (Off-Line Base Caller software, version 1.8) was used for image processing and base identification. The R software package Ballgown was used to calculate fragments per kilobase million (FPKM) at the gene and transcript levels. Sequencing results were deposited in the Gene Expression Omnibus database (accession code GSE210016).

### Vector construction

Human PAXIP1-AS1 cDNA (2275 bp; Kidan Biosciences, Guangzhou, China) and HOXD9 cDNA (1871 bp) were amplified and subcloned into pcDNA3.1 (+) and pEnter-3×Flag-6×His (tag), respectively. PABPC1 (2861 bp; Vigene Biosciences, Shandong, China) and its truncations (GeneCreate Biotech, Wuhan, China) were cloned into pEnter-3×Flag-6×His (tag) using the same method.

### RNA isolation and quantitative real-time polymerase chain reaction (qPCR)

For qPCR, total RNA was isolated from cells using TRIzol Reagent (Invitrogen, Carlsbad, CA, USA) as per the manufacturer’s protocol. A reverse transcriptase cDNA synthesis kit (TaKaRa, Tokyo, Japan) was used to obtain the first-strand cDNA from 500 ng total RNA. The resulting cDNA was analysed by qPCR using a SYBR Green PCR Kit (Takara) and a Roche-LightCycler-480 system (Roche, Basel, Switzerland). Experiments were repeated at least three times to ensure the reproducibility of the results. Human GAPDH was used as an internal control. Comparative quantification was performed using the 2^−ΔΔCt^ method. Primer sequences are listed in Supplementary Table 7.

### Chromatin immunoprecipitation (ChIP) assays

ChIP assays were performed using a ChIP kit from Cell Signalling Technology (Danvers, MA, USA) according to the manufacturer’s instructions. PCR was performed to detect DNA fragments that co-immunoprecipitated with HOXD9. The primers used are listed in Supplementary Table 7.

### RNA interference (RNAi)

Cells were seeded and cultured in growth media until the cell density reached 30–40%, and siRNA transfection was performed using Lipofectamine 3000 (Invitrogen), in accordance with the manufacturer’s instructions. Cells were cultured for an additional 48 h for the subsequent assays. The siRNA sequences used are listed in Supplementary Table 8.

### Coding potential analysis

Coding Potential Calculator (CPC) and the Coding-Potential Assessment Tool (CPAT) were used to analyse the coding potential of PAXIP1-AS1, GAPDH and nuclear enriched abundant transcript 1 (NEAT1). GAPDH and NEAT1 were used to represent the coding probability of the coding and non-coding gene, respectively.

### In situ hybridisation (ISH)

In situ hybridization was performed to detect PAXIP1-AS1 expression in GC specimens, using three PAXIP1-AS1 ISH nucleotide probes corresponding to different PAXIP1-AS1 regions as follows:

Probe-1: 5′-AGATTAGATCACTTTCCAGGCTTTAAGTTGGCCTTTGTTC-3′; Probe-2: 5′-TTCCTTTACAAACTTAGGTTAAACATTCCTAATCTGAAAT-3′; Probe-3: 5′-CTGAAATACTTTTGTCCCAAGCATTTCAGATAAAGGTTAC-3′.

Briefly, paraffin-embedded tissue sections (4 µm) were de-paraffinised and rehydrated using graded ethanol series (100–75%) and deionised water. Sections were then treated with pepsin prepared in 3% sodium citrate for 30 min at 37 °C and next fixed in 4% paraformaldehyde for 10 min. The slides were then hybridised with PAXIP1-AS1 probes overnight at 37 °C. After hybridisation, a series of gradient saline-sodium citrate solutions were used to wash the sections before digoxigenin blocking and anti-digoxigenin incubation. Finally, hybridisation signals were visualised using a 5-bromo-4-chloro-3-indolyl phosphate/nitro blue tetrazolium (BCIP/NBT) liquid substrate system (Roche). Sections were then washed with deionised water to stop the reaction and counterstained with nuclear fast red for 5 min before mounting.

### Immunohistochemistry (IHC)

Paraffin-embedded tissues were prepared and cut into 4-μm-thick sections, which were then transferred to glass slides, de-paraffinised with xylene, and subjected to hydration with decreasing concentrations of ethanol. Three percent hydrogen peroxide was used to block the endogenous peroxidase activity. Citrate buffer was used for antigen retrieval. Sections were incubated overnight with the primary antibody at 4 °C before washing and then with a horseradish peroxidase (HRP)-conjugated secondary antibody (ZSGB-Bio, Beijing, China). Freshly prepared 3, 3′-diaminobenzidine (DAB) complexes were used for staining. Gene expression was visualised using 1 mg/mL DAB and sections were counterstained with hematoxylin. Histopathological analysis confirmed the presence of malignant tissues.

### Subcellular fractionation analysis

Subcellular isolation of RNAs from GES-1 and MKN-74 cells was conducted using the cytoplasmic and nuclear RNA purification kit (Norgenbiotek Corporation, Thorold, ON, Canada) in accordance with the manufacturer’s instructions. NEAT1 was used as the endogenous control for the nucleus, while GAPDH was used as the endogenous control for the cytoplasm. The primers used for PCR are listed in Supplementary Table 7.

### Colony formation assay

For the colony formation assay, 400 cells were seeded per well in 6-well plates and cultured for 14 d. The resulting colonies were fixed in 4% paraformaldehyde and stained with 0.05% crystal violet. The total number of colonies was counted manually. Stained single clones were observed under a microscope.

### EdU incorporation assay

Cell proliferation was detected using the Cell-Light™ EdU Cell Proliferation Detection Kit (RiboBio, Guangzhou, China) as per the manufacturer’s instructions. Each group of transfected tumour cells was seeded into 96-well plates in triplicate at a density of 10^3^ cells/well and incubated for 48 h. Cells were then incubated for an additional 2 h in the medium containing 50 μM EdU. The cells were incubated with Hoechst 33342 for 30 min to stain the DNA and visualised using an inverted fluorescence microscope (Olympus IX73, Japan). Five random fields were captured in each EdU experiment. The captured images were processed and analyzed using ImageJ 1.53. The EdU incorporation rate was determined as the ratio of EdU-positive cells to total Hoechst 33342-positive cells in each field.

### In vitro Transwell migration and invasion assay

The cell migration and invasion assays were performed in 24-well Transwell plates with 8 μm polyethylene terephthalate membrane filters (Falcon, Corning, NY, USA). GC (3 × 10^4^) cells resuspended in 500 mL serum-free medium were seeded in the upper chambers, which contained either uncoated (migration) or Matrigel-coated (invasion) membranes. Each lower chamber was filled with 500 μL medium supplemented with 10% FBS. After incubation for 48 h, the filters were removed and cells that had not migrated were detached from the upper side of the filters using cotton swabs. The filters were then fixed in 4% paraformaldehyde for 15 min. Cells on the lower side of the filters were stained with 0.1% crystal violet for 20 min. Migrating and invading cells were counted in five fields per well that were randomly selected. The migration index (%) represents the number of cells in each group relative to the empty control.

### Wound-healing assay

Cells transfected with the corresponding plasmids were seeded in 6-well plates and incubated for 48 h to form confluent monolayers. Then a linear wound was made with a sterile 10 μL plastic pipette tip. Cellular debris was removed by washing with phosphate-buffered saline (PBS). The distance migrated by the cells in the monolayer to close the wound area over the indicated time were measured. The migration index indicated the cell migration ability. The index was calculated as [(initial wound width − width of the wound measured at the indicated time point)/initial wound width] × 100%. Each experiment was performed at least three times.

### Biotinylated RNA pull-down assay and mass spectrometry

RNA pull-down assays were performed using an RNA pulldown kit (BersinBio, Guangzhou, China), following the manufacturer’s instructions. Briefly, biotin-labelled RNAs were transcribed in vitro and purified using the RNAmax-T7 kit (RiboBio). Fifty picomolar biotin-labelled RNA was incubated with 50 μL streptavidin magnetic beads for 30 min at 25 °C under rotation conditions and then incubated with cell lysates. RNA-associated proteins were eluted and subjected to silver staining, mass spectrometry (MS), or western blotting. Liquid chromatography-tandem MS (LC-MS/MS) was performed by Fitgene Bio. (Guangzhou, China). Proteins that were identified by MS are listed in Supplementary Table 3.

### RNA immunoprecipitation (RIP)

RNA immunoprecipitation was performed using the Magna RIP Kit (Merck Millipore), as described previously [48]. Cell lysates were prepared using RIP lysis buffer at 4 °C and then incubated with 5 μg of anti-PABPC1 or IgG overnight at 4 °C. RNA-protein immunocomplexes were collected using protein A/G magnetic beads. RNA was extracted, purified, and subjected to qPCR. The truncations of PABPC1 plasmids were transfected into MKN-74 cells. Cell lysates were prepared and used for RIP assays with the anti-6×His antibody as described above. Primers used for qPCR of PAXIP1-AS1 and PAK1 are listed in Supplementary Table 7.

### Protein stability assays

To determine the stability of the endogenous PABPC1 protein, cells were transfected with either an empty plasmid or PAXIP1-AS1 plasmid with 100 μM cycloheximide (CHX) for the time periods indicated in the figure legends. Total protein in the cell lysates was determined using the bicinchoninic acid (BCA) assay. Lysates with equivalent amounts of protein were resolved by SDS-PAGE and analysed by western blotting to determine the abundance of endogenous PABPC1.

### Protein degradation assay

MG132 (carbobenzoxy-Leu-Leu-leucinal), a peptide aldehyde that effectively blocks the proteolytic activity of the 26S proteasome complex, was purchased from Sigma-Aldrich. PABPC1 expression was measured in the background of MG132 (10 nM) treatment (6 h) under different PAXAP1-AS1 conditions to confirm whether PAXAP1-AS1 facilitates PABPC1 degradation.

### RNA stability assays

GC cells under different transfection status were cultured in 12-well plates and treated with 10 μg/mL actinomycin D (ActD) at the indicated time points before cell scraping. Total RNA was isolated and qPCR was used to quantify the relative PAK1 levels as described previously [46].

### F-actin cytoskeleton staining

Transfected cells were seeded onto coverslips in 12-well plates. Then, they were washed with PBS and fixed in 3.7% formaldehyde on ice for 10 min. Following this, cells were permeabilised in 0.5% Triton X-100 for 10 min, washed with PBS and incubated with 5 U/mL rhodamine-conjugated phallotoxin (Molecular Probes, Eugene, OR, USA) prepared in PBS (1:40 dilution) for 30 min before staining the nuclei with 1 μg/mL Hoechst 33342. F-actin cytoskeleton staining was evaluated and images were captured using a fluorescence microscope.

### Ubiquitylation analysis

Ubiquitination assays were conducted to analyse the ubiquitination of PABPC1 as previously described [49]. First, MKN-74 cells were transfected with PAXIP1-AS1 or Control for 48 h. Cell lysates were prepared using the cell lysis buffer. Whole-cell lysates were immunoprecipitated with the indicated antibodies (anti-PABPC1, or IgG) on protein A/G beads (Santa Cruz Biotechnology) overnight at 4 °C. Beads were washed and boiled in SDS-loading buffer and immunoprecipitated proteins were analysed by western blotting.

### Construction and transfection of lentiviral vectors in vivo

A PAXIP1-AS1 lentiviral expression vector (pLENti-EF1-mCherry-P2A-Neo-CMV-MCS-3×flag-WPRE) or PABPC1 lentiviral expression vector (pLENti-EF1-EGFP-F2A-Puro-CMV-MCS-WPRE) was constructed by Obio Technology (Shanghai, China). Empty vectors were used as controls. Lentiviruses were transduced into GC cell lines using 5 μg/mL polybrene. Puromycin or geneticin (G418) was used to treat cell pools to produce puromycin/neomycin-resistant cells. Selected pools of overexpressing cells were used for subsequent experiments.

### Promoter analysis

The 2 kb (−1700 ~ +300 bp) region directly upstream of PAXIP1-AS1 was predicted using UCSC software. The HOXD9 binding sites in the PAXIP1-AS1 promoter were analysed using the transcription factor prediction program PROMO (http://alggen.lsi.upc.es/cgi-bin/promo_v3/promo/promoinit.cgi?dirDB=TF_8.3/). The full-length potential binding region of PAXIP1-AS1 upstream (PAXIP1-AS1p: −1700 ~ +300 bp) containing the sequence 5′-ATATATTATT-3′ (site 1: −1298 ~ −1308) and 5′-AATATGAGTC-3′ (site 2: −1503 ~ −1513) was subcloned into the KpnI-XhoI sites of the pGL3-basic vector from Genechem (Shanghai, China). Site-directed mutagenesis was performed on sequence 5′-AATATGAGTC-3′ to 5′-CCGCGGCGGC-3′ at positions −1513 ~ −1503 (site 2) of PAXIP1-AS1p. The dual-luciferase assay was performed using the Dual-Luciferase Reporter Assay Kit (Promega, Madison, WI, USA). Briefly, 1 × 10^5^ cells were seeded in each well of a 24-well plate and were co-transfected with HOXD9 or Vector plasmids, pGL3-Basic constructs expressing firefly luciferase, and Renilla luciferase plasmids for 48 h. The relative promoter activity of PAXIP1-AS1 was measured and determined as firefly luciferase activity normalised to Renilla luciferase activity. Each experiment was performed at least three times.

### Luciferase activity assay for 3′-UTR study

To construct a 3′-UTR luciferase reporter plasmid, the 3′-UTR of PAK1 (+2178 ~ 3459) was inserted into downstream of the firefly luciferase ORF between NheI and SalI under the control of the PGK promoter. The mutant reporter construct was generated by changing the motif ACUAAUC (ACTAATC) in the 3′-UTR of PAK1 to GCCAGUA (GCCAGTA). The pmirGLO construct containing either the wild-type or mutant 3′-UTR of PAK1 was transfected into PABPC1-overexpressed or control GC cells. Cells were harvested after 48 h to measure luciferase intensity. Dual-luciferase reporter assays were performed as described above.

### Animal studies

BALB/c nude mice (nu/nu, aged 4–6 weeks, and weighing ~20 g) were purchased from the Central Laboratory of Animal Science at the Southern Medical University (Guangzhou, China) and bred in specific pathogen-free facilities. The mice were randomly allocated using a list of random numbers generated by the computer. All experimental procedures were approved by the Laboratory Animal Care and Use Committee of Southern Medical University and Nanfang Hospital.

To evaluate in vivo tumourigenesis, six mice were randomly divided into Control and PAXIP1-AS1 groups, with three mice in each. Cells (5 × 10^6^) were suspended in 0.1 mL PBS and injected subcutaneously into the right flank of the mice. The resulting tumour size was measured weekly. Tumour volumes were calculated as total tumour volume (mm^3^) = π/6 × *L* × *W*^2^, where *L* is the length and *W* is the width. The mice were euthanised 28 d after inoculation, and the tumours were dissected and measured. The fluorescence emitted by the cells was imaged using the In-Vivo Imaging System Fx Pro (Bruker, USA). The proliferation index, based on Ki-67 immunostaining, was determined by the ratio of Ki-67-positive cells to the total number of cells in three randomly selected fields.

For tail vein-lung metastasis model, two batches of mice from various groups were used to evaluate the metastatic ability of cancer cells. The first batch was composed of the Control group and PAXIP1-AS1, whereas the second batch comprised three groups, i.e., Control + Vector, PAXIP1-AS1 + Vector, and PAXIP1-AS1 + PABPC1. A total of 5 × 10^6^ lentivirus-transfected cells were injected into the tail veins of nude mice, which were euthanised after 28 d. Lung tissues were dissected and tested for bioluminescence as previously described [3]. Metastatic tumours were fixed and embedded in paraffin, for hematoxylin and eosin (HE) staining and immunochemistry using anti-E-cadherin and anti-MMP2 antibodies.

For intrasplenic liver metastasis, two groups of lentivirus-transfected cells expressing PAXIP1-AS1 or Control and three groups of cells expressing Control + Vector, PAXIP1-AS1 + Vector, or PAXIP1-AS1 + PABPC1 were prepared respectively before. The mice were first anaesthetised with pentobarbital (60 mg/kg) via intraperitoneal injection. Then a left flank incision was made to expose the spleen and a total of 2 × 10^6^ lentivirus-transfected cells suspended in 50 µL PBS were injected into the spleen. The spleen was restored after careful hemostasis and the abdominal wall was carefully closed with a 5-0 suture (Silaikang, Shanghai, China). Three weeks after the surgery, all mice were sacrificed and their livers were collected and imaged. External whole-body fluorescence images of the liver were captured as above. Hematoxylin and eosin staining was used to visualise the metastatic loci.

### Statistical analysis

Statistical analyses were performed using SPSS (version 23.0; SPSS Inc., Chicago, IL, USA). All experiments were repeated three times to determine statistical significance. Quantitative data are shown as mean ± standard deviation. Survival rates were calculated using Kaplan–Meier curves, and the log-rank test was used to test the differences in the survival rates between the two groups. Correlations were assessed using Pearson’s correlation. Student’s t-test and one-way analysis of variance (ANOVA) were performed to assess the significance of the differences. Statistical significance was set at *P* < 0.05 (two-tailed).

## Supplementary information

Supplementary Figure Legends

Supplementary Figure 1

Supplementary Figure 2

Supplementary Figure 3

Supplementary Figure 4

Supplementary Figure 5

Supplementary Figure 6

Supplementary Figure 7

Supplementary Figure 8

Supplementary Figure 9

Supplementary Tables

Raw data of western blotting

Reproducibility Checklist

## Data Availability

All data are available within the article and supplementary files, or from the authors upon reasonable request.
