# Supplementary material for: The HOXD9-mediated PAXIP1-AS1 regulates gastric cancer progression through PABPC1/PAK1 modulation
Source: Cell Death Dis. 2023 May 24;14(5):341. doi: 10.1038/s41419-023-05862-5 (PMC10209196; doi:10.1038/s41419-023-05862-5)
Supplement: Supplementary file 1 — Supplementary Figure Legends [file 41419_2023_5862_MOESM1_ESM.docx]

**Supplementary figure legends**

**Supplementary Fig. 1. Kaplan-Meier analyses for overall survival (OS) and disease-specific survival (DSS) of indicated lncRNAs using gastric cancer patients' data from TCGA. (A-E)** Kaplan‒Meier analyses for OS by expression of LINC01605(A), LINC00648(B), APCODD1L-AS1(C), LINC00618(D), and ANKRD10-IT1(E). **(F)&(G)**Overall survival(F) and DSS(G) by expression of PAXIP1-AS1. Kaplan-Meier survival curves were analysed by log-rank test.

**Supplementary Fig. 2. Coding potential of PAXIP1-AS1. (A) & (B)**The sequences of PAXIP1-AS1, nuclear enriched abundant transcript 1 (NEAT1), and GAPDH were evaluated by the Coding Potential Assessment Tool (CPAT, A) and Coding Potential Calculator (CPC, B) programs. NEAT1 served as a lncRNA control and GAPDH as a coding RNA control.

**Supplementary Fig. 3. Forced expression of PAXIP1-AS1 inhibits GC cell proliferation. (A)** Detection of overexpression and knockdown efficiency of PAXIP1-AS1 plasmids, siRNAs, and siRNA pool by qPCR. Student’s t-test. ****, *P* < 0.001. Control vs. PAXIP1-AS1; ***, *P* < 0.01 and ****, *P* < 0.001. Scr siRNA vs. PAXIP1-AS1 siRNAp. **(B) & (C)** The average percentage of EdU-positive cells was determined as the number of EdU-positive cells/the number of Hoechst-positive cells in corresponding groups. ***, *P* < 0.01, control vs. PAXIP1-AS1; Scr siRNA vs. PAXIP1-AS1 siRNAp. **(D) & (E)** Quantification of crystal violet-stained cell colonies formed by indicated GC cell lines, 14 d after inoculation. ***, *P* < 0.01, ****, *P* < 0.01, Control vs. PAXIP1-AS1; ***, *P* < 0.01, Scr siRNA vs. PAXIP1-AS1 siRNAp. **(F)** HE staining was performed on subcutaneous tumour samples. **(G)** Tumour volumes were measured on the indicated days to assess the effects of PAXIP1-AS1 on subcutaneous tumour growth. ***, *P* < 0.01. **(H)** Cell proliferation rate in cells with forced expression of PAXIP1-AS1 was significantly lower than that in control cells (Ki-67), as observed by IHC. ***, *P* < 0.01. Scale bar, 100 μm in F.

**Supplementary Fig. 4. Forced PAXIP1-AS1 expression suppresses GC cell invasion and metastasis. (A) - (D)** Transwell migration and invasion assays were performed and quantitative results in GC cells are shown. ***, *P* < 0.01 and ****, *P* < 0.001, Control vs. PAXIP1-AS1; ****, *P* < 0.001, Scr siRNA vs. PAXIP1-AS1 siRNAp. **(E) - (G)** Wound healing assay was used to detect cell motility after transfection and the migration indexes are shown. ***, *P* < 0.01 and ****, *P* < 0.001, control vs. PAXIP1-AS1; ****, *P* < 0.001, Scr siRNA vs. PAXIP1-AS1 siRNAp. Each bar represented mean ± SD. Results were reproduced in three independent experiments. **(H)** Gross appearance and fluorescence images of lungs dissected from mice received intravenous tail injection of MKN-74/PAXIP1-AS1 and control cells, respectively.  **(I)** The number of metastatic pulmonary tumours was counted. ***, *P* < 0.01. **(J)-(L)** HE staining, and IHC of MMP2 and E-cadherin were applied on the pulmonary sections of mice from PAXIP1-AS1 and the control groups. **(M)** Images of liver and metastatic tumours from intrasplenic liver metastasis model in the control and PAXIP1-AS1 groups. **(N)** HE staining of liver sections of mice from the Control and PAXIP1-AS1 groups. Scale bar, 100 μm in J-L and N.

**Supplementary Fig. 5. PAXIP1-AS1 may be related to PABPC1.** **(A)** Predicted interaction between PABPC1 and PAXIP1-AS1 based on the RBPDB database (http://rbpdb.ccbr.utoronto.ca/). The red box highlights the potential interacting sequences of PABPC1 and PAXIP1-AS1. **(B)** Immunoprecipitation detecting His-tagged PABPC1 full-length or truncation mutants via anti-His antibody in MKN-74 cells.

**Supplementary Fig. 6. PABPC1 mediates effects of PAXIP1-AS1 on tumour migration and invasion in GC cells**. **(A)** GC cells were transfected with Vector and PABPC1, after which modulations in PABPC1 expression were confirmed by western blotting. **(B)** The wound healing assay was used to detect MKN-74 cell motility. The migration index is shown in the panel below. **, *P* < 0.05, ****, *P* < 0.001. **(C)** Gross appearance and fluorescence images of MKN-74 cells in corresponding expression status-induced metastatic lung tumours. (n = 3 in each group, the remaining two of three samples were shown. **(D)** HE staining of lung samples from mice. **(E)** Gross appearance and fluorescence images of livers and hepatic metastatic tumours induced by corresponding expression status. **(F)** HE staining of hepatic tissue from mice in corresponding groups. Scale bar, 100 μm in D & F.

**Supplementary Fig. 7. Knockdown of PAXIP1-AS1 promotes gastric cancer migration and invasion which can be reversed by PABPC1 knockdown. (A)** PAXIP1-AS1 knockdown regulated the expression of EMT markers and PABPC1 knockdown could restore their expression. **(B) & (C)** Transwell migration and invasion assays were used to detect the migratory and invasive ability of GC cells in different transfection statuses and their quantitative results were shown in the lower panel. ***, *P* < 0.01; ****, *P* < 0.001. **(D) & (E)** The migratory ability of transfected gastric cells was detected by wound healing assay and the migration index was shown in the right panel, respectively. **, *P* < 0.05; ***, *P* < 0.01; ****, *P* < 0.001.

**Supplementary Fig. 8. PAK1 mediates PABPC1-induced EMT and metastasis in GC cells**. **(A)** The knockdown efficiency of PABPC1 siRNAs and siRNA pool was confirmed by western blotting. **(B)** The relative expression of 12 genes in PABPC1 knockdown MKN-74 cells was detected by qPCR. The experiment was performed in triplicate. *, *P* > 0.05; **, *P* < 0.05; ***, *P* < 0.01; ****, *P* < 0.001. Scr siRNA vs. PABPC1 siRNAp. **(C)** Prediction of the interaction between PAK1 mRNA 3'UTR and PABPC1 based on the RBPDB database. The red box highlights the potential interacting sequences. **(D)** The PAK1 expression of AGS and MKN-74 cells was detected by western blotting analysis after transfecting PAK1 siRNAp and Scr siRNA for 48h. **(E)** MKN-74 cells were stained with rhodamine-phallotoxin to identify F-actin filaments, which were visualised by fluorescence microscopy. **(F)&(G)** Quantification of migration and invasion capabilities of GC cells. ****, *P* < 0.001. **(H)& (I)** The wound healing assay was used to detect GC cell motility. The migration indexes of AGS (H) and MKN-74 (I) are shown, respectively. **, *P* < 0.05, ***, *P* < 0.01, ****, *P* < 0.001. Scale bar, 10 μm in E.

**Supplementary Fig. 9. Forced expression of PAXIP1-AS1 reduces the mRNA stability of PAK1 in AGS (left panel) and MKN-74 (right panel) cells.**
