# Supplementary figures and images for: The HOXD9-mediated PAXIP1-AS1 regulates gastric cancer progression through PABPC1/PAK1 modulation
Source: Cell Death Dis. 2023 May 24;14(5):341. doi: 10.1038/s41419-023-05862-5 (PMC10209196; doi:10.1038/s41419-023-05862-5)

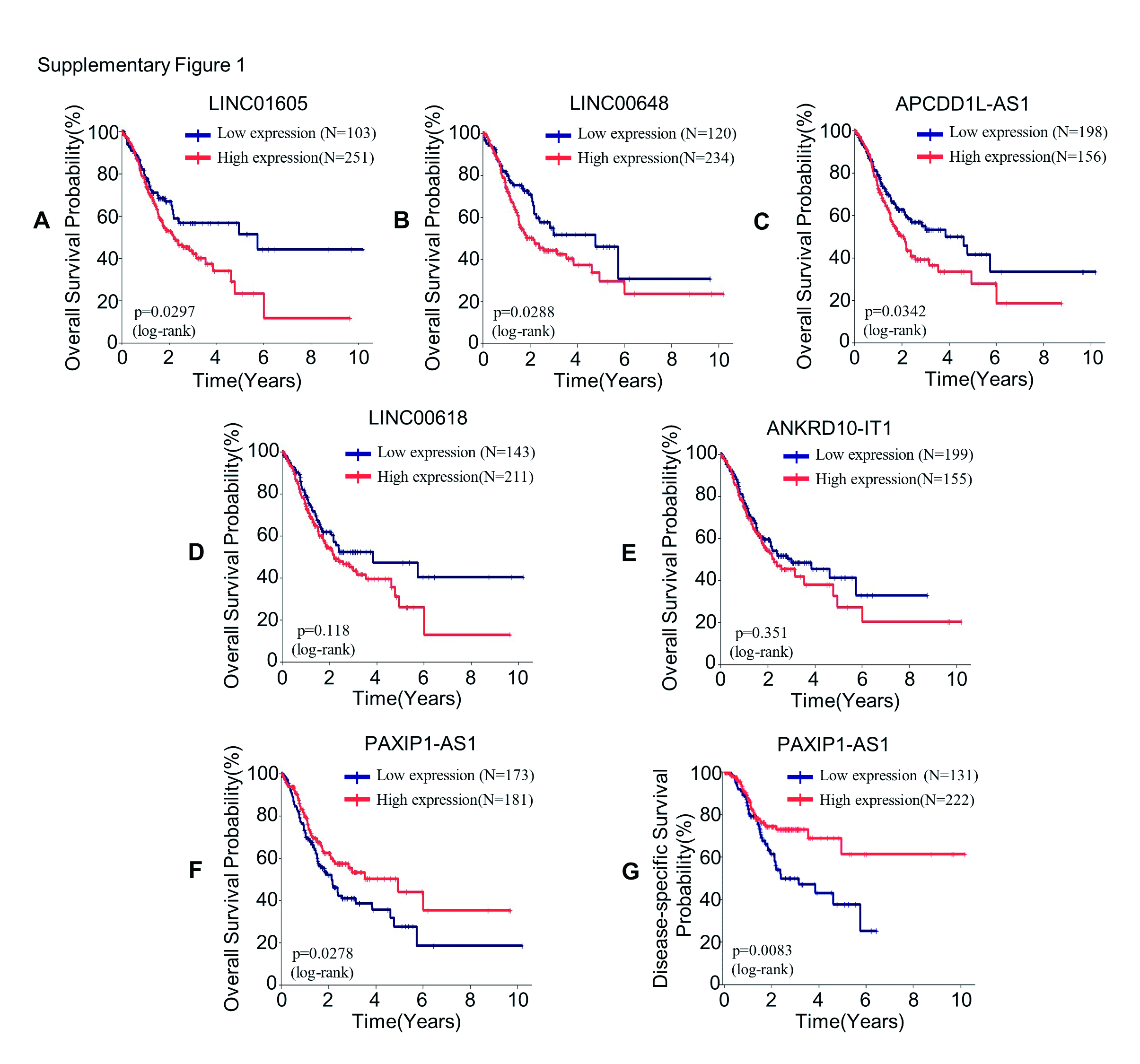

Supplement: Supplementary file 2 — Supplementary Figure 1 [file 41419_2023_5862_MOESM2_ESM.tif]

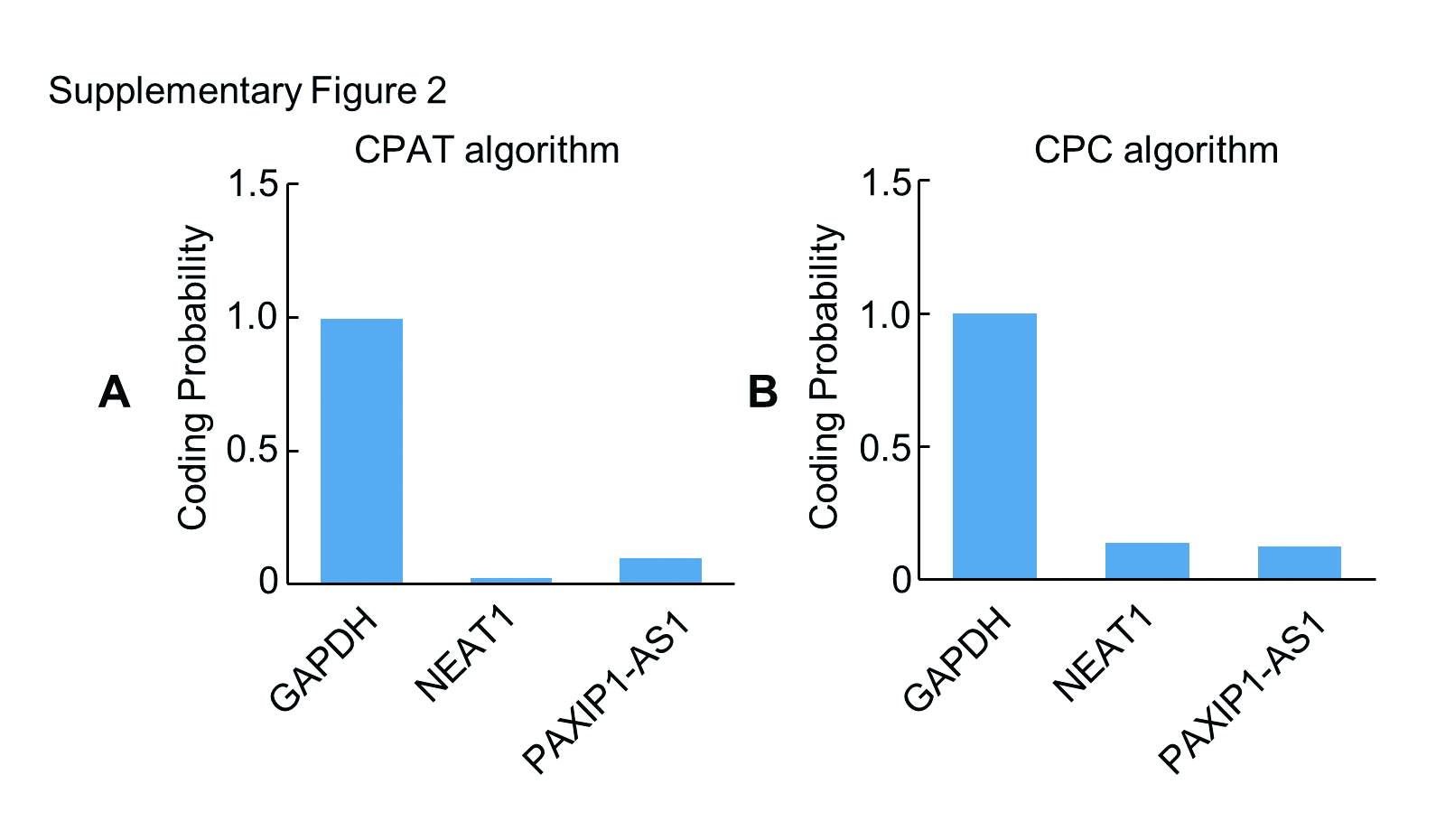

Supplement: Supplementary file 3 — Supplementary Figure 2 [file 41419_2023_5862_MOESM3_ESM.tif]

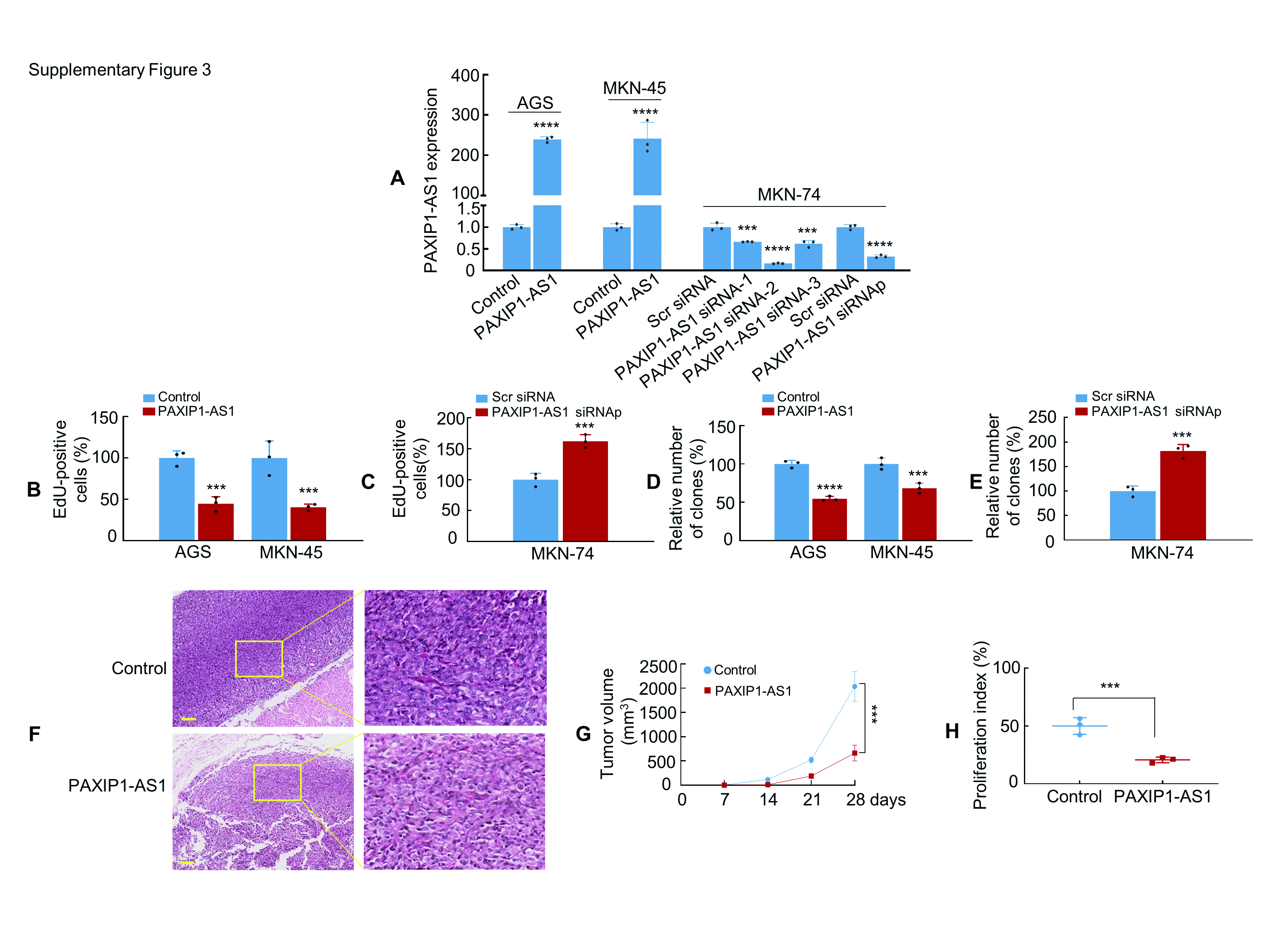

Supplement: Supplementary file 4 — Supplementary Figure 3 [file 41419_2023_5862_MOESM4_ESM.tif]

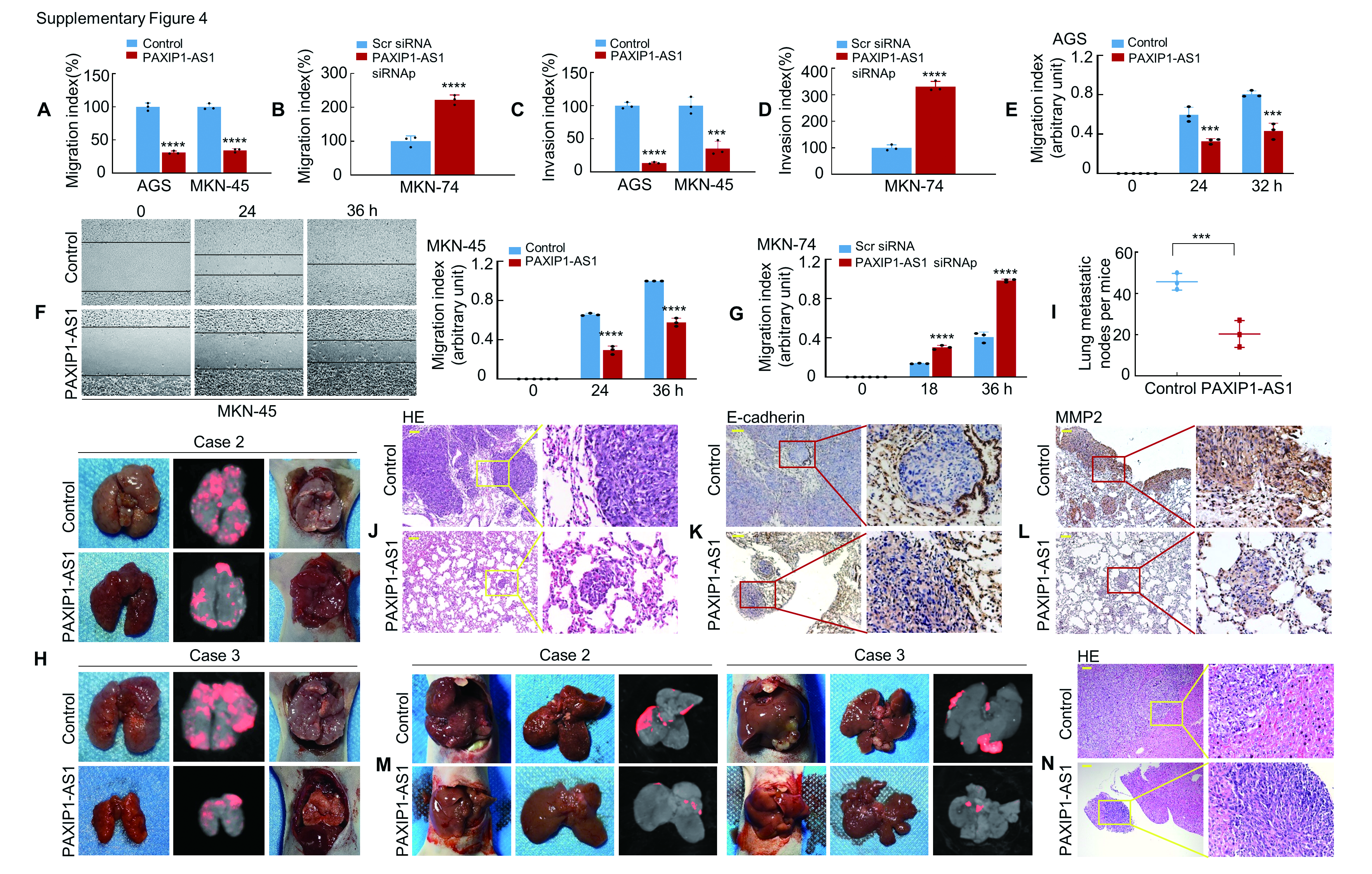

Supplement: Supplementary file 5 — Supplementary Figure 4 [file 41419_2023_5862_MOESM5_ESM.tif]

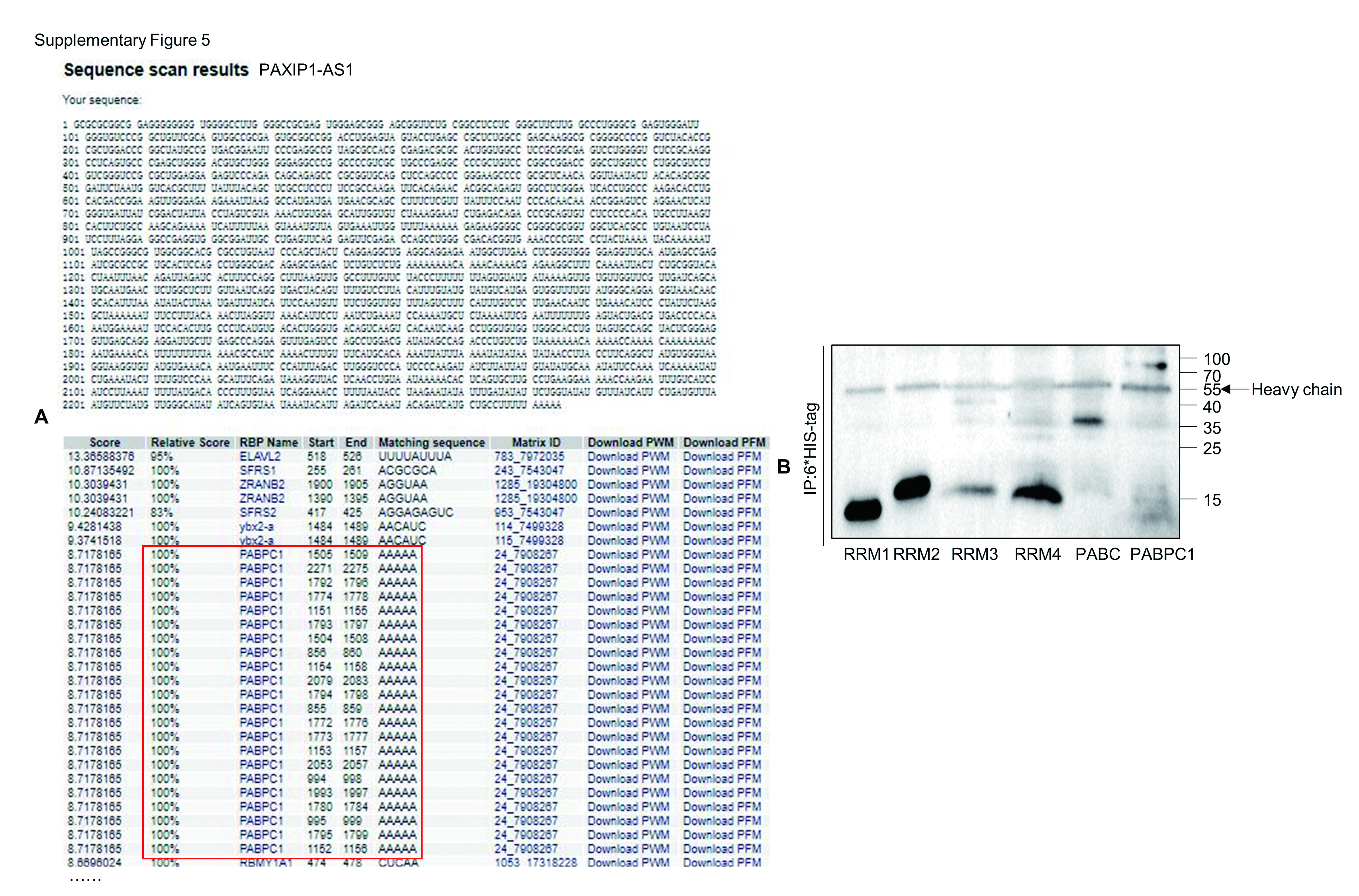

Supplement: Supplementary file 6 — Supplementary Figure 5 [file 41419_2023_5862_MOESM6_ESM.tif]

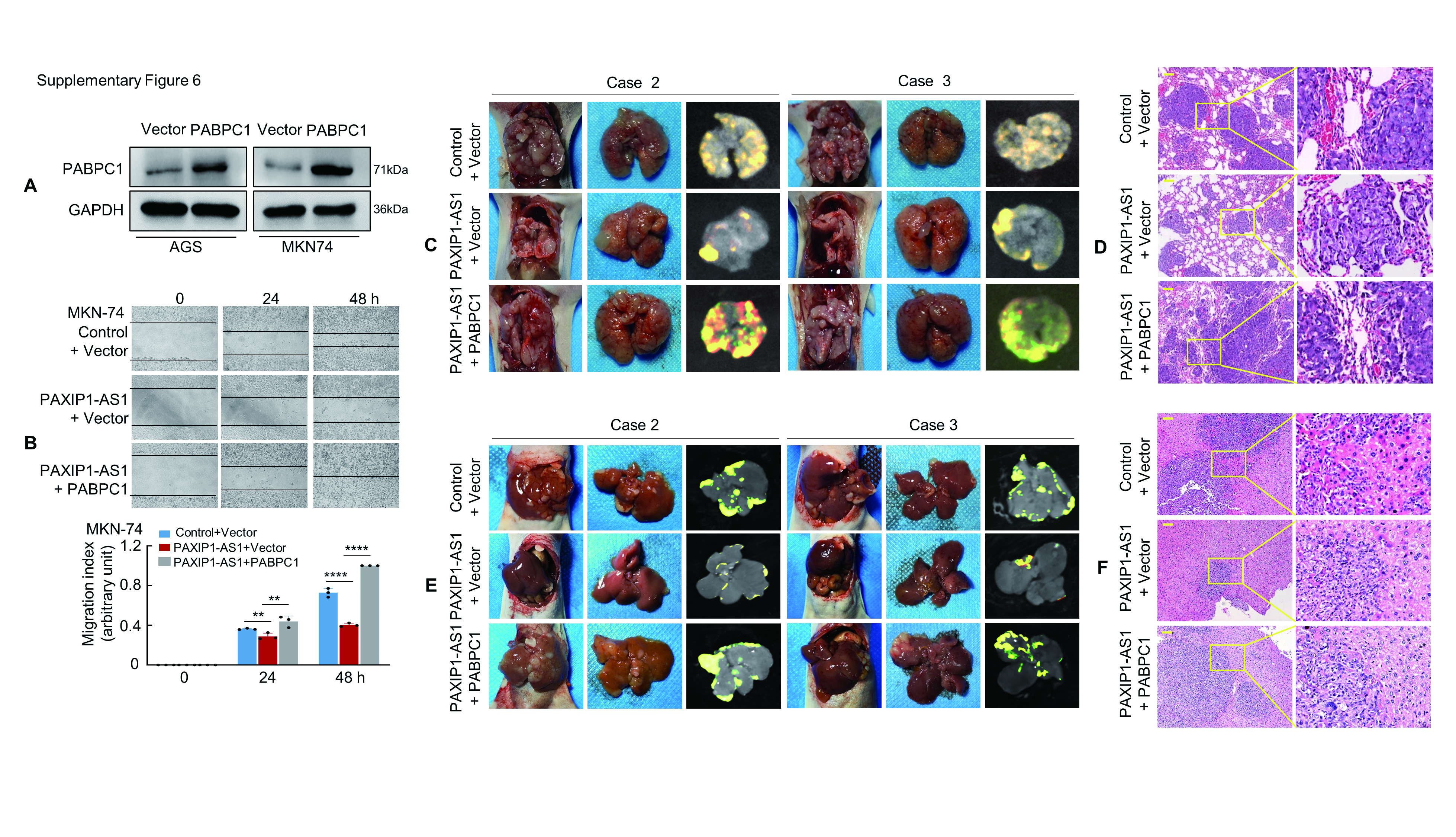

Supplement: Supplementary file 7 — Supplementary Figure 6 [file 41419_2023_5862_MOESM7_ESM.tif]

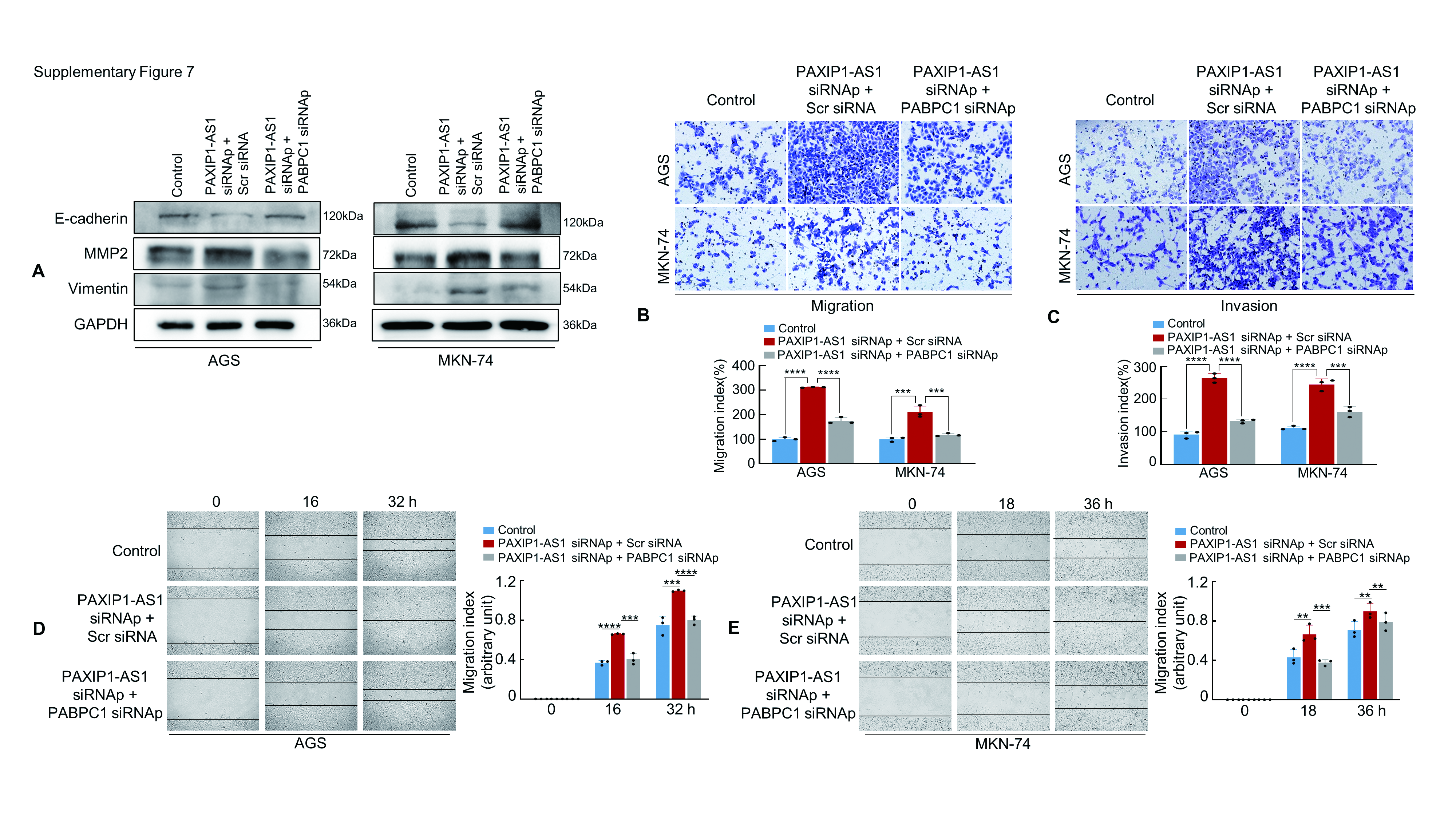

Supplement: Supplementary file 8 — Supplementary Figure 7 [file 41419_2023_5862_MOESM8_ESM.tif]

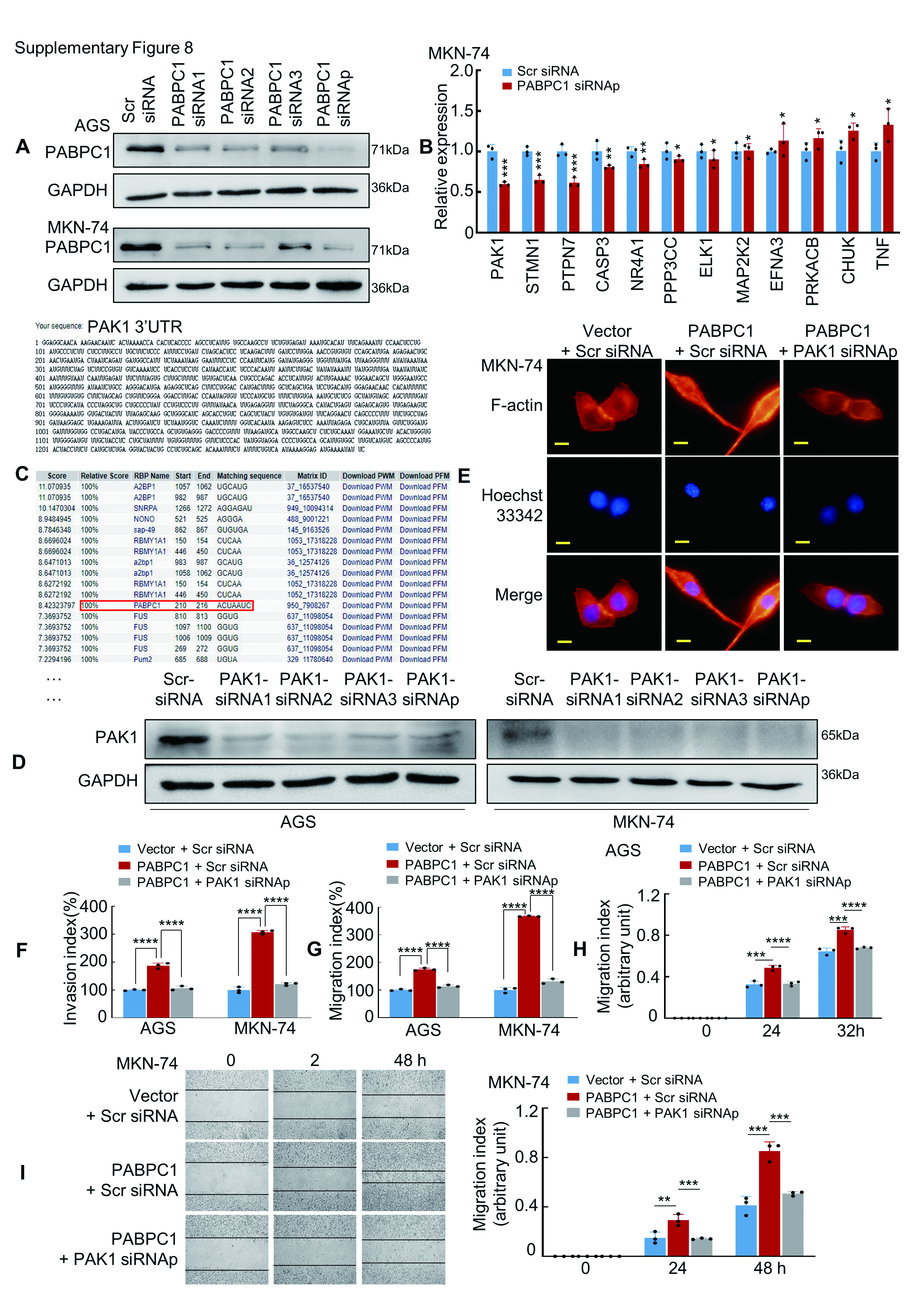

Supplement: Supplementary file 9 — Supplementary Figure 8 [file 41419_2023_5862_MOESM9_ESM.tif]

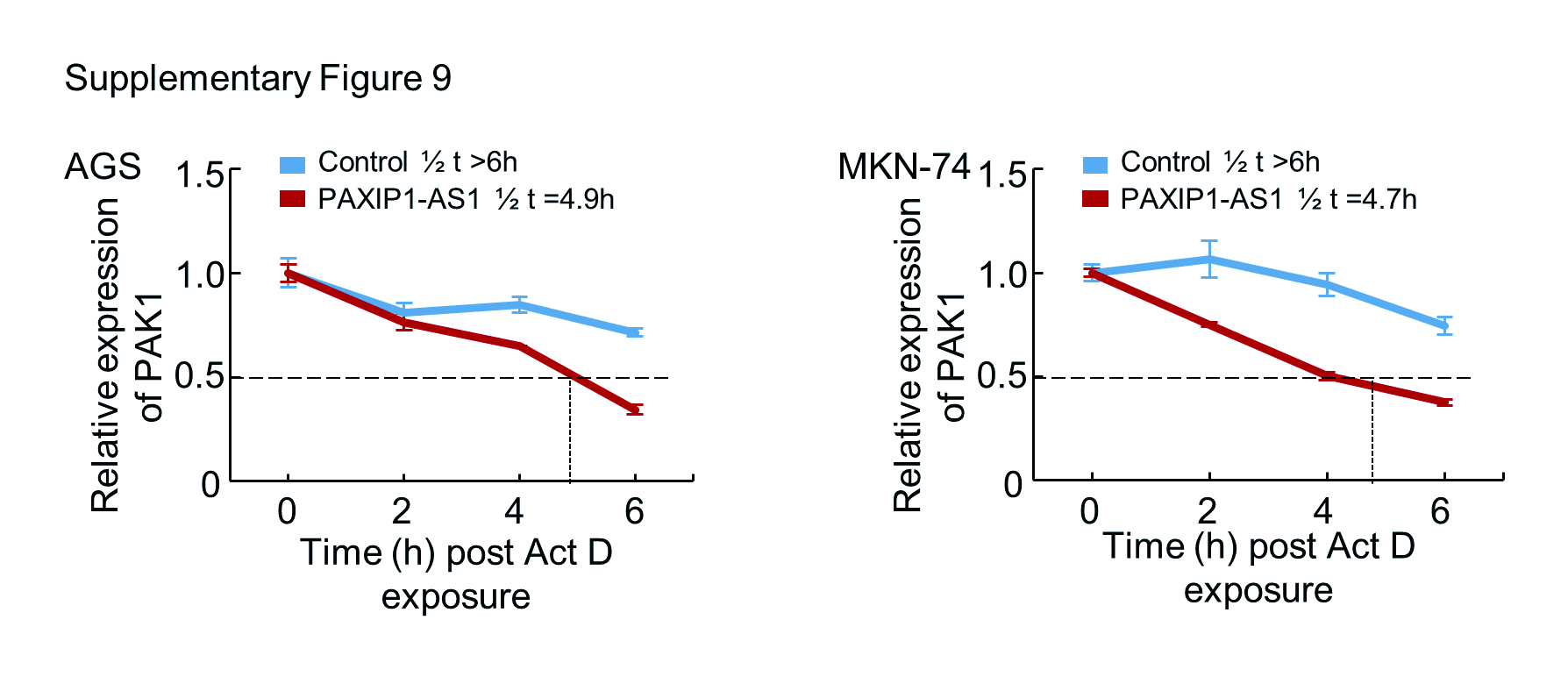

Supplement: Supplementary file 10 — Supplementary Figure 9 [file 41419_2023_5862_MOESM10_ESM.tif]

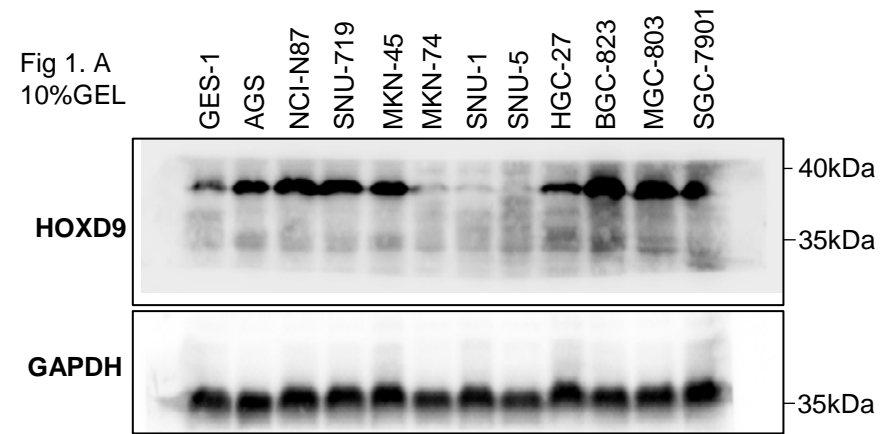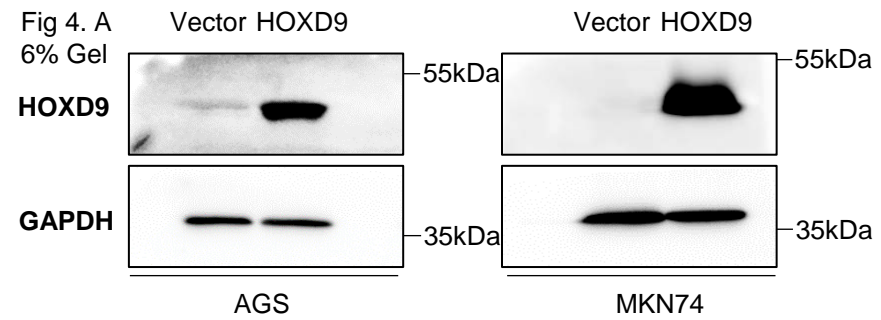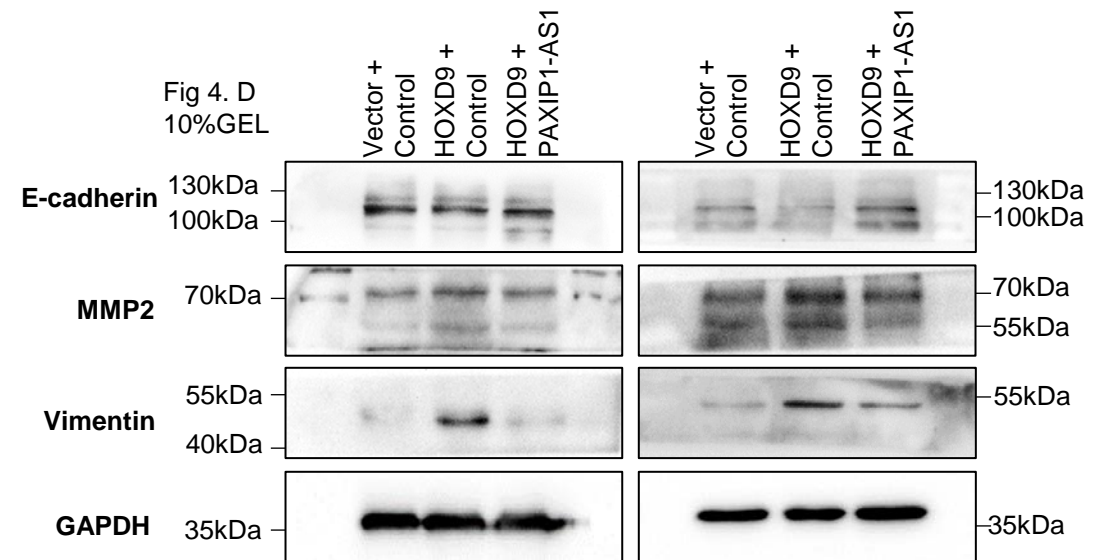

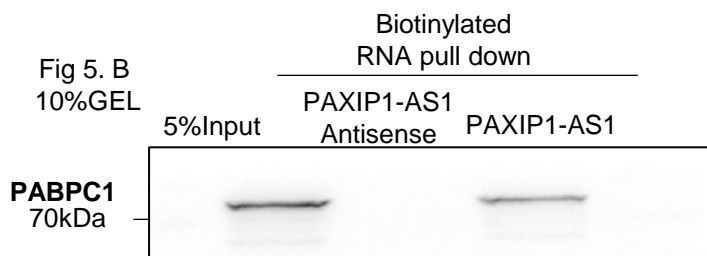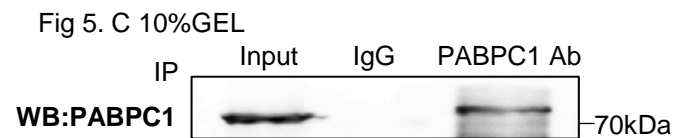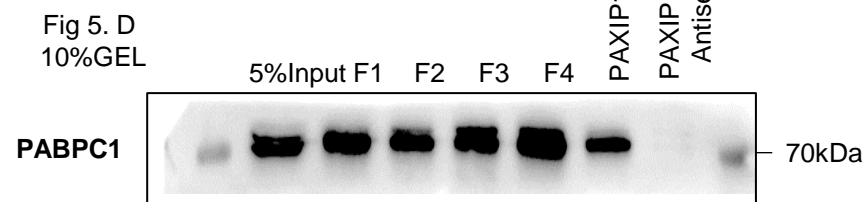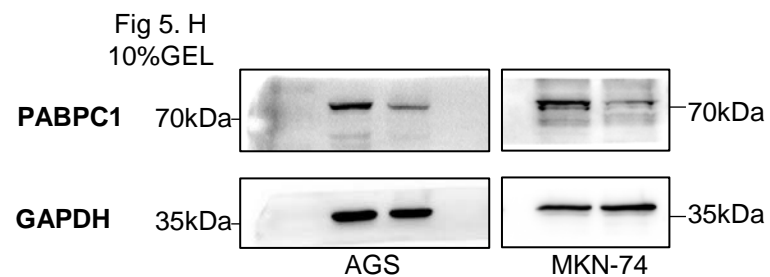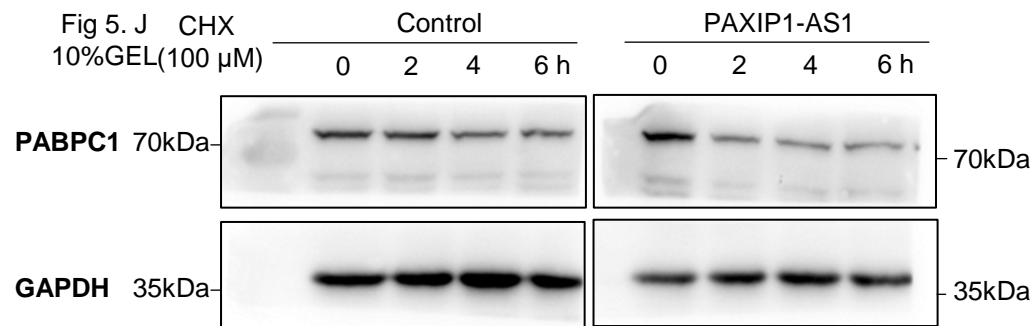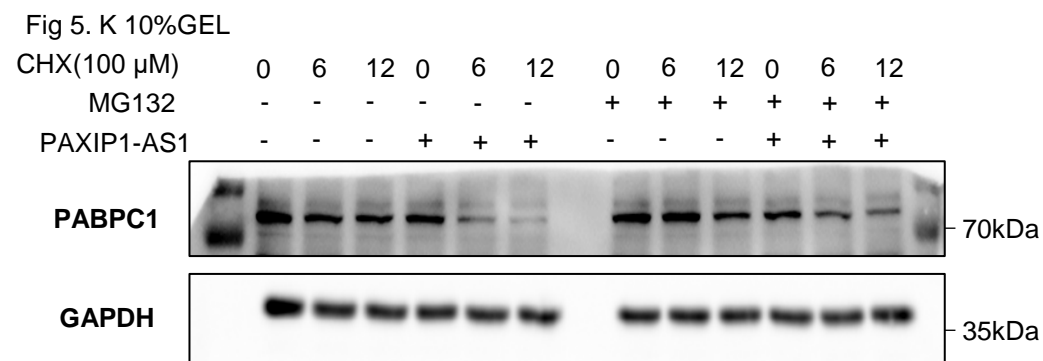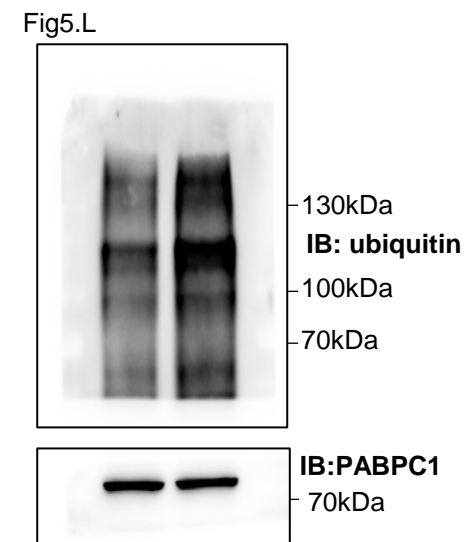

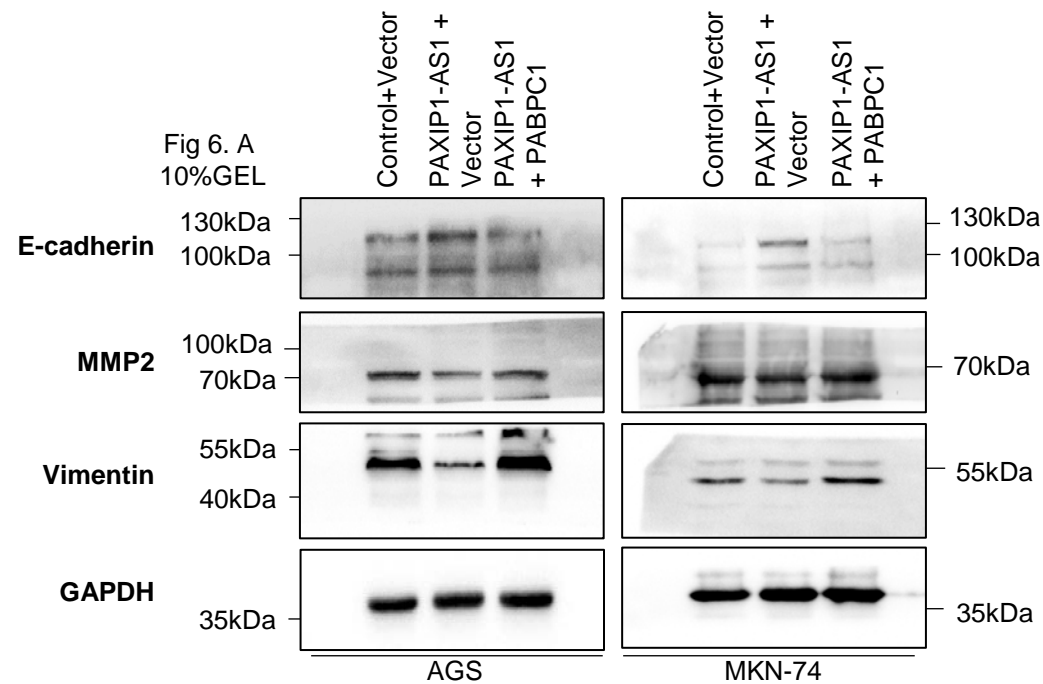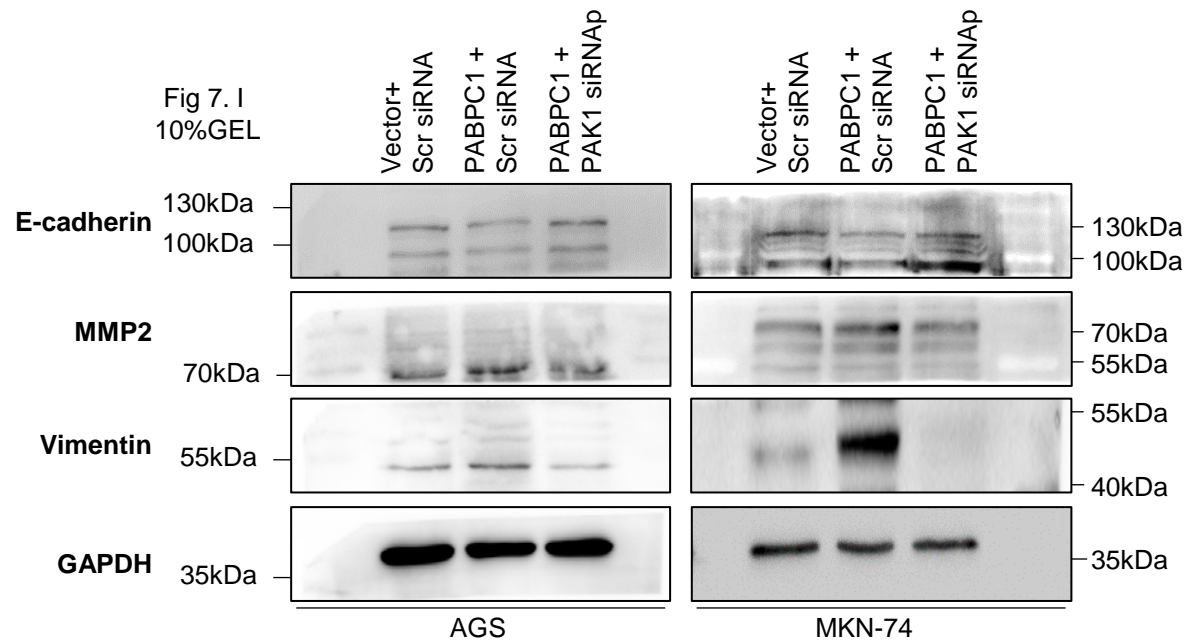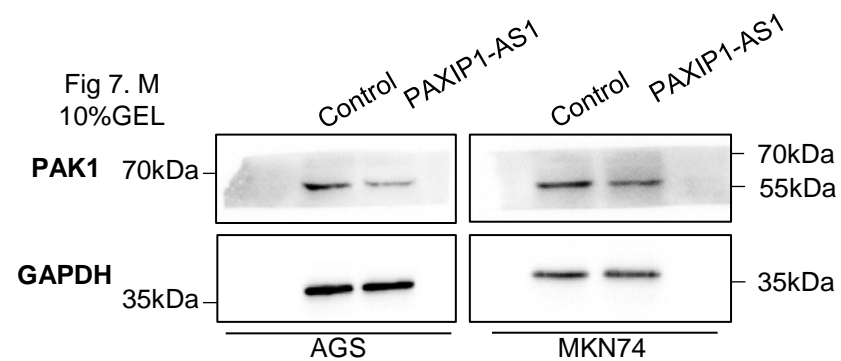

Fig 8. B 10%GEL

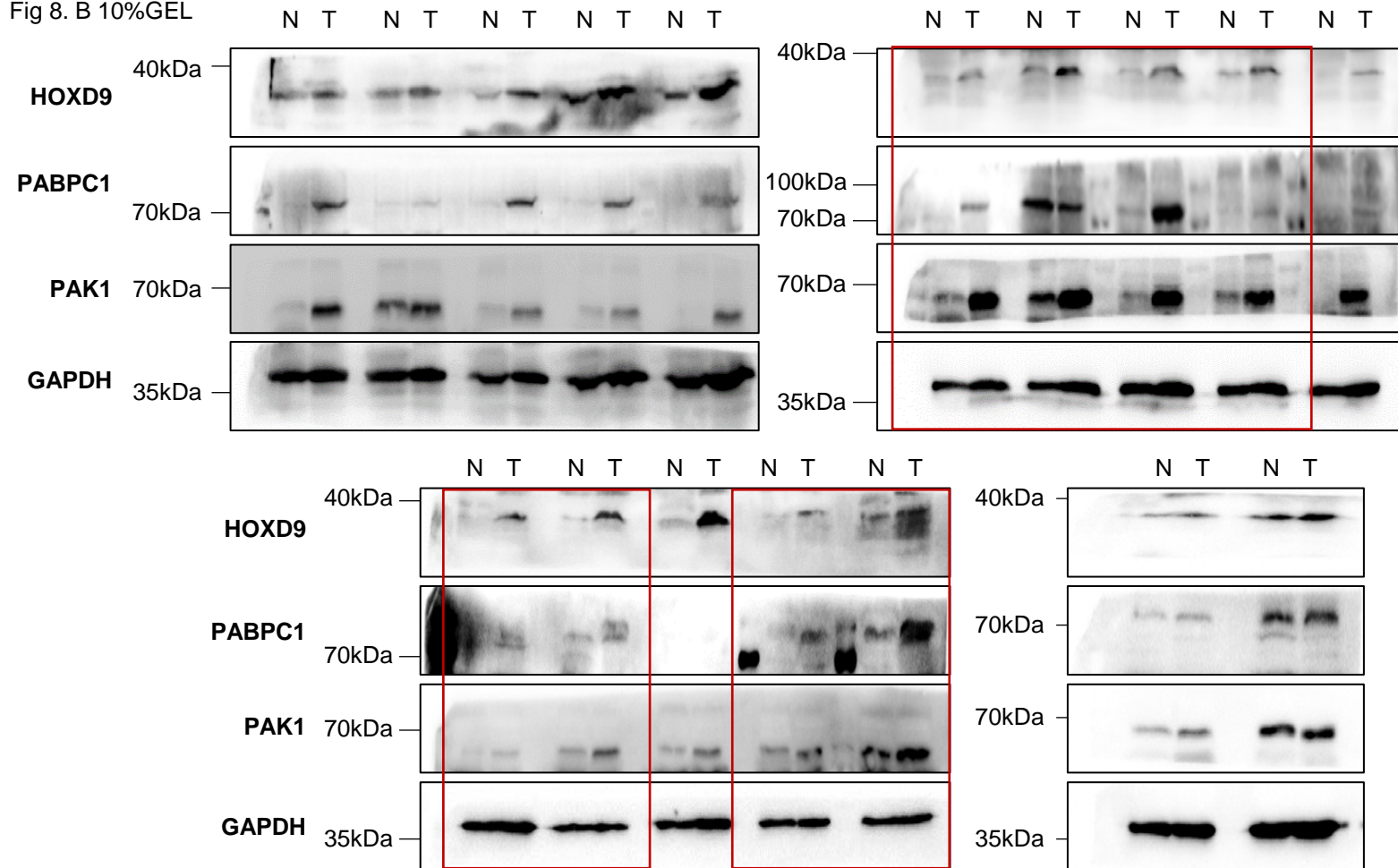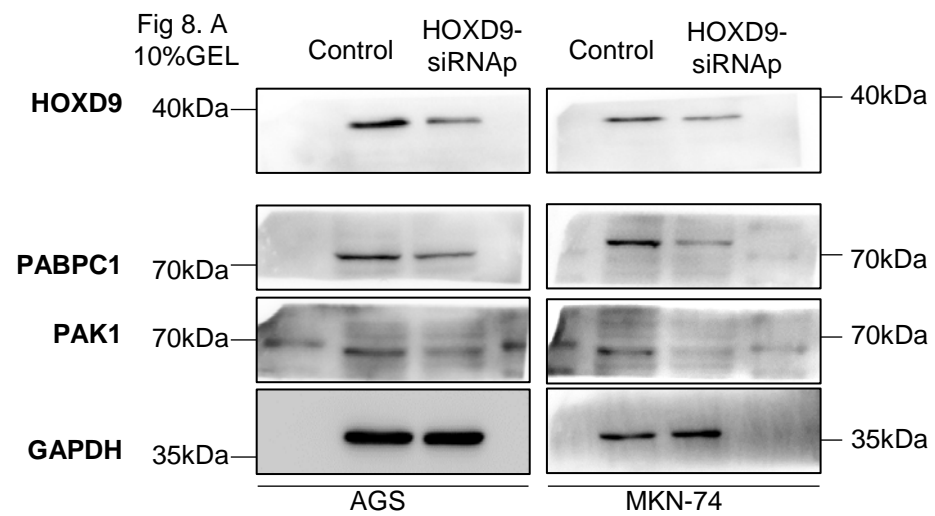

FigS5.B 15%GEL

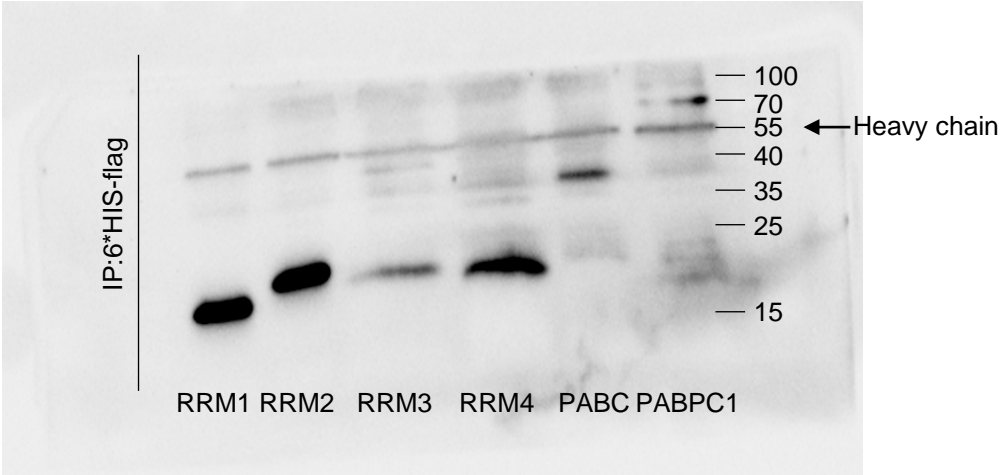

Fig S6. A  
10%GEL

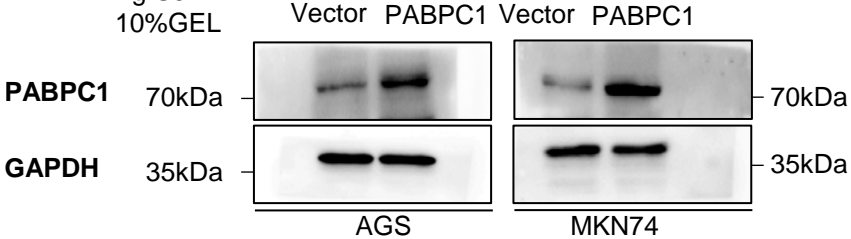

Fig S7. A  
10%GEL

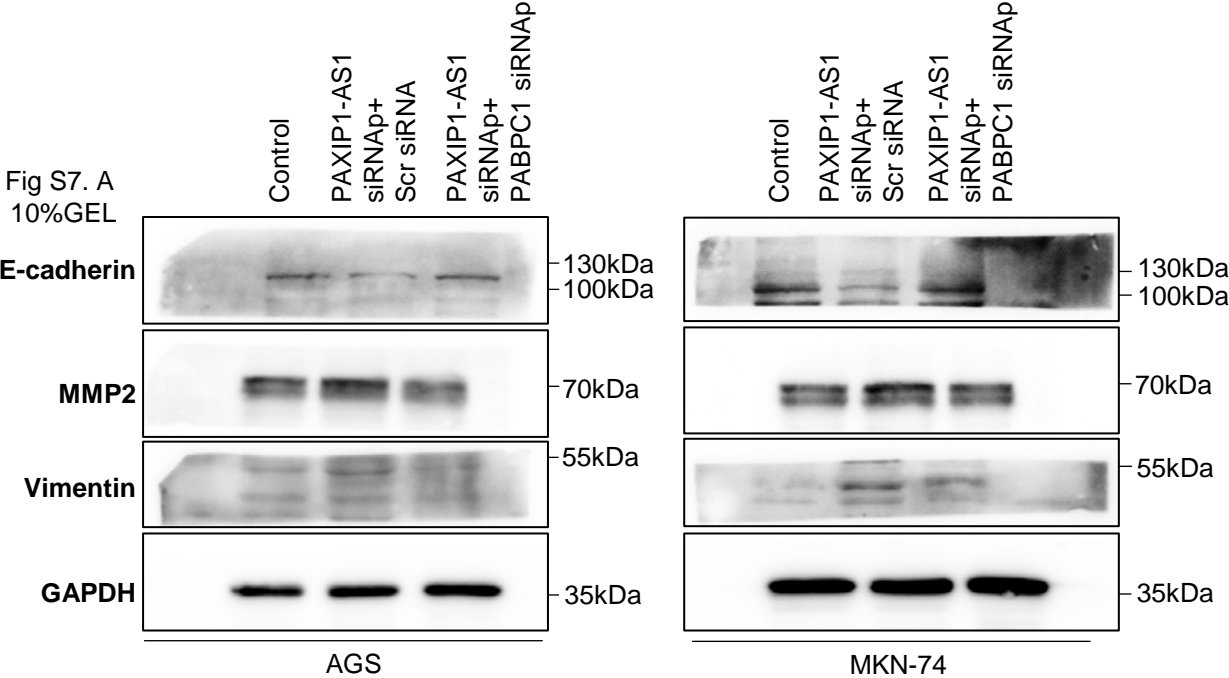

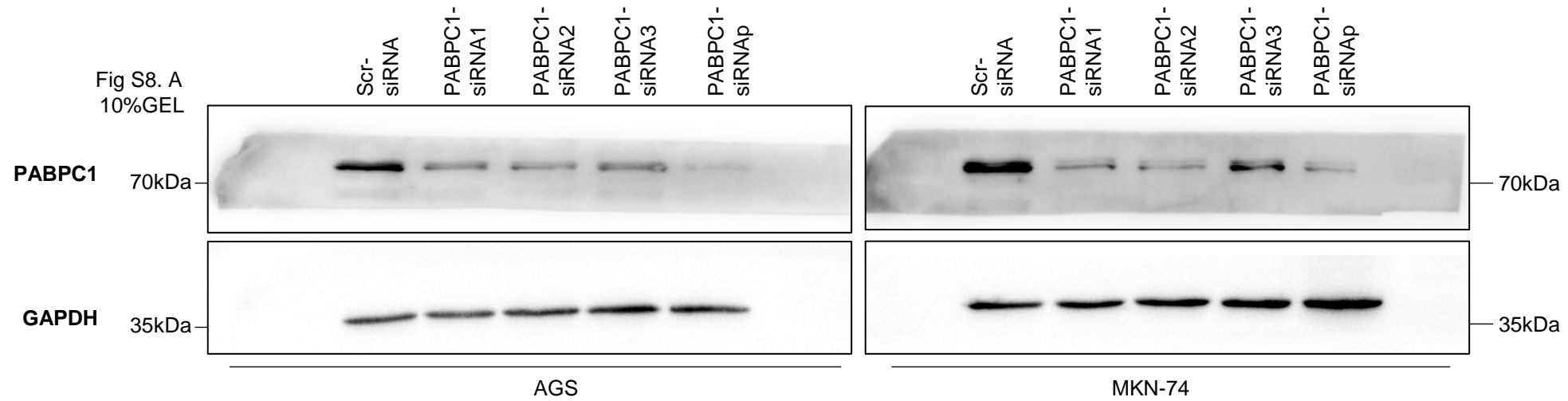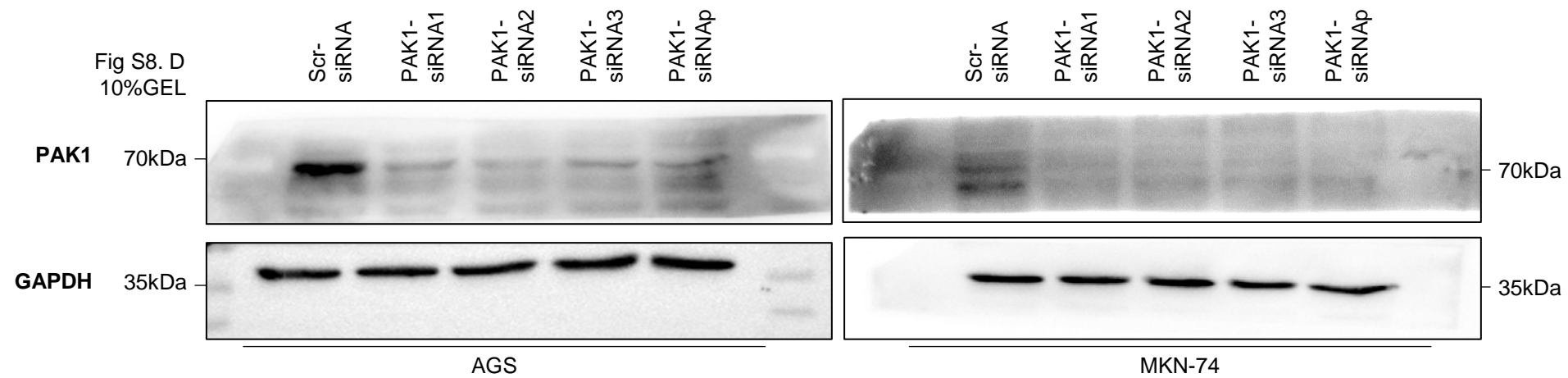

Supplement: Supplementary file 12 — Raw data of western blotting [file 41419_2023_5862_MOESM12_ESM.pdf]
