# Supplementary material for: The HOXD9-mediated PAXIP1-AS1 regulates gastric cancer progression through PABPC1/PAK1 modulation
Source: Cell Death Dis. 2023 May 24;14(5):341. doi: 10.1038/s41419-023-05862-5 (PMC10209196; doi:10.1038/s41419-023-05862-5)
Supplement: Supplementary file 11 — Supplementary Tables [file 41419_2023_5862_MOESM11_ESM.docx]

**Supplementary Table 1.** Differentially expressed genes (FC>1.2 or <0.83, P<0.05) in the RNA sequence

**Track_id Gene_Name Locus Strand Gene_Type log2FC Fold_Changp_value q_value Test_FPKMControl_FPHOXD9-1 HOXD9-2 HOXD9-3 Vector-1 Vector-2 Vector-3**

| ENSG00000001629.9_3 | ANKIB1 | chr7:918755+ | protein_codi-0.349209 | 0.7850144 | 0.0411695 | 0.3138435 | 3.0656084 | 3.4148174 | 8.2999627 | 7.7819986 | 6.1853132 | 9.3008593 | 10.138524 | 9.5727093 |
| --- | --- | --- | --- | --- | --- | --- | --- | --- | --- | --- | --- | --- | --- | --- |
| ENSG00000003509.15_2 | NDUFAF7 | chr2:374587+ | protein_codi-0.349362 | 0.7849311 | 0.0081993 | 0.210149 | 2.1878485 | 2.5372106 | 3.6489486 | 3.6222951 | 3.4016107 | 4.3757841 | 5.3136352 | 4.762463 |
| ENSG00000004478.7_2 | FKBP4 | chr12:29041+ | protein_codi0.3377572 | 1.2637904 | 0.0033981 | 0.1733337 | 5.6622875 | 5.3245303 | 51.629242 | 49.655142 | 47.719687 | 41.188021 | 36.983663 | 39.155407 |
| ENSG00000005810.17_1 | MYCBP2 | chr13:77618- | protein_codi-0.39208 | 0.7620301 | 0.0175763 | 0.2506617 | 2.6704892 | 3.0625694 | 5.5339009 | 5.9321464 | 4.6970731 | 7.2925367 | 7.9617738 | 6.8468323 |
| ENSG00000006459.10_2 | KDM7A | chr7:139784- | protein_codi-0.563715 | 0.676558 | 0.0099364 | 0.2170995 | 2.1751727 | 2.7388873 | 3.9559239 | 3.775522 | 2.8925232 | 5.2437751 | 6.151596 | 5.6621044 |
| ENSG00000006611.15_2 | USH1C | chr11:17515- | protein_codi-0.310274 | 0.8064888 | 0.0013686 | 0.1390335 | 2.9516324 | 3.261906 | 6.5980237 | 6.4916218 | 7.1341679 | 8.4707653 | 8.771883 | 8.5374286 |
| ENSG00000006652.13_3 | IFRD1 | chr7:112063+ | protein_codi-0.405175 | 0.7551449 | 0.0027274 | 0.1614437 | 1.6155337 | 2.0207082 | 2.1275902 | 2.0037182 | 2.0626916 | 2.9384868 | 2.8559052 | 3.3997261 |
| ENSG00000008294.20_2 | SPAG9 | chr17:49039- | protein_codi-0.337629 | 0.7913409 | 0.0330298 | 0.2965163 | 4.5314656 | 4.8690945 | 24.480358 | 22.547181 | 19.61469 | 28.021169 | 30.379819 | 26.40723 |
| ENSG00000011347.9_3 | SYT7 | chr11:61282- | protein_codi-0.269355 | 0.8296902 | 0.0278113 | 0.2861428 | 3.2484494 | 3.5178047 | 8.5509151 | 7.9754816 | 9.0124276 | 11.489678 | 10.225645 | 9.7184419 |
| ENSG00000011454.16_3 | RABGAP1 | chr9:125703+ | protein_codi-0.282485 | 0.8221736 | 0.03385 | 0.2991259 | 1.9718212 | 2.2543063 | 3.200346 | 2.957843 | 2.6306832 | 3.5207101 | 4.2170093 | 3.6048258 |
| ENSG00000011523.13_3 | CEP68 | chr2:652835+ | protein_codi-0.431434 | 0.7415242 | 0.0026802 | 0.1614437 | 1.4928504 | 1.9242847 | 1.9996181 | 1.8162044 | 1.6390531 | 2.9963578 | 2.7047381 | 2.6930092 |
| ENSG00000016864.18_3 | GLT8D1 | chr3:527285- | protein_codi-0.283217 | 0.8217566 | 0.0372316 | 0.3067727 | 2.2704488 | 2.5536659 | 3.551334 | 3.9380941 | 3.9971404 | 4.6744865 | 4.4375612 | 5.559328 |
| ENSG00000018408.14_3 | WWTR1 | chr3:149235- | protein_codi-0.353551 | 0.7826554 | 0.0087757 | 0.2115899 | 1.8923768 | 2.2459277 | 2.6087652 | 2.7947336 | 2.7363422 | 3.5011615 | 3.5318087 | 4.232131 |
| ENSG00000018610.13_2 | CXorf56 | chrX:11867- | protein_codi0.3833051 | 1.3043266 | 0.0446634 | 0.3195842 | 4.1172441 | 3.733939 | 17.917775 | 17.772213 | 13.718259 | 11.373716 | 12.35404 | 13.255095 |
| ENSG00000023287.12_3 | RB1CC1 | chr8:535350- | protein_codi-0.426785 | 0.7439177 | 0.0466707 | 0.3240376 | 2.7927901 | 3.2195752 | 6.5047272 | 6.4113979 | 4.9828176 | 7.0965209 | 9.3838633 | 8.6141026 |
| ENSG00000028203.17_2 | VEZT | chr12:95611+ | protein_codi-0.302997 | 0.8105666 | 0.0141833 | 0.2399539 | 2.6083211 | 2.9113184 | 5.0995395 | 5.4979315 | 4.72106 | 6.0205982 | 6.7512541 | 6.8241362 |
| ENSG00000029725.16_3 | RABEP1 | chr17:51855+ | protein_codi-0.330197 | 0.7954277 | 0.0067998 | 0.204539 | 3.1186111 | 3.4488083 | 8.0086789 | 7.707428 | 7.3528651 | 9.7110636 | 10.766843 | 9.329796 |
| ENSG00000033030.13_2 | ZCCHC8 | chr12:12295- | protein_codi-0.292449 | 0.8165148 | 0.0200249 | 0.2575012 | 2.2646033 | 2.5570523 | 3.9897788 | 4.0928161 | 3.366176 | 4.660417 | 5.1569969 | 4.8483177 |
| ENSG00000033170.16_3 | FUT8 | chr14:65877+ | protein_codi-0.33364 | 0.7935319 | 0.0486392 | 0.327235 | 2.5735198 | 2.9071597 | 5.6466529 | 4.9923853 | 4.2956253 | 6.3348849 | 7.2527166 | 5.9732715 |
| ENSG00000035928.15_3 | RFC1 | chr4:392890- | protein_codi-0.332848 | 0.7939675 | 0.0412287 | 0.3138435 | 3.4001174 | 3.7329654 | 10.39921 | 10.203083 | 8.2129695 | 11.480414 | 13.195078 | 12.268961 |
| ENSG00000038219.12_2 | BOD1L1 | chr4:135703- | protein_codi-0.409065 | 0.7531115 | 0.0304005 | 0.2906274 | 2.1081858 | 2.5172504 | 3.4967045 | 3.7870213 | 2.7232463 | 4.4294797 | 5.2179044 | 4.5577808 |
| ENSG00000042286.14_2 | AIFM2 | chr10:71857- | protein_codi0.3551656 | 1.2791324 | 0.0007695 | 0.1159361 | 3.8718376 | 3.5166719 | 13.860356 | 14.184393 | 12.905644 | 10.520372 | 10.43366 | 10.382022 |
| ENSG00000046604.12_2 | DSG2 | chr18:29078+ | protein_codi-0.33287 | 0.7939552 | 0.0440056 | 0.318365 | 5.3314232 | 5.6642936 | 42.632539 | 41.824699 | 33.934144 | 47.849709 | 53.425457 | 48.057206 |
| ENSG00000047188.15_2 | YTHDC2 | chr5:112849+ | protein_codi-0.334962 | 0.792805 | 0.0428168 | 0.3153643 | 2.121867 | 2.456829 | 3.8902705 | 3.3826224 | 2.8474228 | 4.3416513 | 4.9225325 | 4.230638 |
| ENSG00000053372.4_2 | MRTO4 | chr1:195780+ | protein_codi0.2937256 | 1.2258017 | 0.0156919 | 0.2474439 | 5.0752127 | 4.7814871 | 30.40742 | 33.574339 | 34.284796 | 28.384358 | 25.022684 | 26.204755 |
| ENSG00000054282.15_2 | SDCCAG8 | chr1:243419+ | protein_codi-0.365243 | 0.776338 | 0.0417708 | 0.3140442 | 1.90952 | 2.2747632 | 2.9450922 | 2.9023786 | 2.4441483 | 3.2309433 | 4.4564963 | 3.9086807 |
| ENSG00000054654.16_3 | SYNE2 | chr14:64319+ | protein_codi-0.530366 | 0.692379 | 0.0203089 | 0.2588284 | 2.6588075 | 3.1891736 | 5.5711823 | 6.0202274 | 4.4594349 | 7.3467743 | 9.3194943 | 7.8091432 |
| ENSG00000059804.15_2 | SLC2A3 | chr12:80718- | protein_codi0.3231216 | 1.2510345 | 0.009581 | 0.2144024 | 4.2252243 | 3.9021027 | 19.105253 | 17.629682 | 16.467984 | 14.699113 | 13.394734 | 13.786695 |
| ENSG00000060642.10_3 | PIGV | chr1:271139+ | protein_codi-0.375386 | 0.770899 | 0.0387842 | 0.3100145 | 1.7550477 | 2.1304339 | 2.5654515 | 2.0338812 | 2.5551089 | 3.7798857 | 3.5780583 | 2.8359653 |
| ENSG00000064692.18_3 | SNCAIP | chr5:121647+ | protein_codi-0.386642 | 0.7649081 | 0.0015691 | 0.1419323 | 1.7759373 | 2.1625789 | 2.5229064 | 2.4695064 | 2.2859607 | 3.5520542 | 3.6502017 | 3.2395923 |
| ENSG00000065060.16_2 | UHRF1BP1 | chr6:347597+ | protein_codi-0.471637 | 0.7211458 | 0.0001992 | 0.0943724 | 2.9416732 | 3.4133103 | 6.5204947 | 6.9591166 | 6.576774 | 9.8259231 | 9.2848053 | 9.8608877 |
| ENSG00000065361.14_3 | ERBB3 | chr12:56473+ | protein_codi-0.287966 | 0.8190559 | 0.0083275 | 0.210149 | 3.4992164 | 3.7871826 | 10.243316 | 10.228876 | 10.451876 | 13.97374 | 12.218235 | 12.294222 |
| ENSG00000065911.11_2 | MTHFD2 | chr2:744256+ | protein_codi-0.681438 | 0.6235433 | 0.0018721 | 0.1515851 | 4.4561161 | 5.1375545 | 22.585603 | 21.980429 | 18.510514 | 32.45711 | 34.860137 | 35.355796 |
| ENSG00000065989.15_2 | PDE4A | chr19:10527+ | protein_codi-0.38659 | 0.7649353 | 0.034242 | 0.2993278 | 1.9480537 | 2.334644 | 2.6646592 | 3.5086629 | 2.4768538 | 3.7860881 | 4.0482079 | 4.3122155 |
| ENSG00000066739.11_2 | ATG2B | chr14:96745- | protein_codi-0.368419 | 0.7746307 | 0.0213821 | 0.2625473 | 1.5645258 | 1.9329452 | 2.1084406 | 2.1364477 | 1.6541589 | 2.5239602 | 3.093883 | 2.858842 |
| ENSG00000068079.7_2 | IFI35 | chr17:41158+ | protein_codi0.3801399 | 1.3014681 | 0.0184868 | 0.2558547 | 2.9721264 | 2.5919864 | 7.4424897 | 5.9066198 | 7.2863158 | 5.2676345 | 5.07257 | 4.7586517 |
| ENSG00000068724.15_3 | TTC7A | chr2:471432+ | protein_codi0.3824592 | 1.303562 | 0.0019601 | 0.1522433 | 4.0552362 | 3.672777 | 15.938291 | 14.688438 | 16.289957 | 11.631549 | 11.350257 | 12.295831 |
| ENSG00000068796.16_3 | KIF2A | chr5:616019+ | protein_codi-0.488504 | 0.7127638 | 0.0493954 | 0.3291951 | 3.5887867 | 4.0772908 | 12.153932 | 11.406836 | 9.6728576 | 12.838142 | 18.692004 | 16.651956 |
| ENSG00000069535.13_2 | MAOB | chrX:43625- | protein_codi0.4263498 | 1.3438292 | 0.0049969 | 0.1891494 | 1.8614817 | 1.4351319 | 2.3382786 | 2.8016455 | 2.7808659 | 1.5482335 | 1.8352093 | 1.7367107 |
| ENSG00000069869.15_2 | NEDD4 | chr15:56119- | protein_codi-0.334937 | 0.7928189 | 0.0436389 | 0.3171923 | 1.7843051 | 2.1192419 | 2.7431119 | 2.616929 | 2.0186672 | 3.0970421 | 3.7320536 | 3.2300552 |
| ENSG00000070047.11_2 | PHRF1 | chr11:57648+ | protein_codi0.3207836 | 1.2490087 | 0.0123109 | 0.2343664 | 4.5833582 | 4.2625746 | 22.834226 | 21.510079 | 24.680698 | 19.556638 | 17.563947 | 17.529601 |
| ENSG00000070526.14_3 | ST6GALNAC1 | chr17:74620- | protein_codi-0.378122 | 0.7694386 | 0.0249759 | 0.2761127 | 1.5622758 | 1.9403977 | 2.1026671 | 2.1117148 | 1.6677226 | 3.1218557 | 2.4438838 | 2.9830196 |
| ENSG00000070669.16_3 | ASNS | chr7:974814- | protein_codi-0.53805 | 0.688701 | 0.0121943 | 0.2337722 | 3.546733 | 4.0847833 | 11.330232 | 10.411335 | 10.342567 | 16.750736 | 13.485876 | 18.000532 |
| ENSG00000074071.14_3 | MRPS34 | chr16:18218- | protein_codi0.3068731 | 1.2370237 | 0.0185697 | 0.2558547 | 6.3427185 | 6.0358454 | 76.859945 | 79.099802 | 84.723526 | 70.853404 | 60.181283 | 63.245923 |
| ENSG00000074935.13_2 | TUBE1 | chr6:112391- | protein_codi-0.422224 | 0.7462731 | 0.0005737 | 0.1106916 | 0.7857491 | 1.2079734 | 0.8133792 | 0.6987968 | 0.6633057 | 1.250529 | 1.3229978 | 1.358171 |
| ENSG00000075151.20_3 | EIF4G3 | chr1:211327- | protein_codi-0.298256 | 0.8132347 | 0.0106697 | 0.2227482 | 3.6570612 | 3.9553175 | 11.826607 | 12.246086 | 10.815607 | 14.165587 | 15.48003 | 13.93449 |
| ENSG00000076554.15_4 | TPD52 | chr8:809469- | protein_codi-0.285525 | 0.8204432 | 0.0417569 | 0.3140442 | 2.0520804 | 2.337605 | 3.4344636 | 3.0346897 | 2.9862269 | 3.5510622 | 4.106173 | 4.5572424 |
| ENSG00000077713.18_3 | SLC25A43 | chrX:11853+ | protein_codi-0.371962 | 0.7727312 | 0.0089922 | 0.2129568 | 1.6537153 | 2.0256768 | 1.9929515 | 2.1509083 | 2.3030738 | 3.3358431 | 3.1849361 | 2.7205408 |
| ENSG00000078403.16_3 | MLLT10 | chr10:21813+ | protein_codi-0.316409 | 0.8030665 | 0.0165437 | 0.2498875 | 2.8144106 | 3.1308193 | 6.6145803 | 6.105331 | 5.4333293 | 7.4629654 | 8.2103447 | 7.6221017 |
| ENSG00000079112.9_3 | CDH17 | chr8:951393- | protein_codi-0.352972 | 0.7829695 | 0.0496038 | 0.3295165 | 6.367931 | 6.720903 | 92.609559 | 82.694794 | 70.911124 | 98.722489 | 113.88735 | 101.45068 |
| ENSG00000081386.12_2 | ZNF510 | chr9:995181- | protein_codi-0.286672 | 0.819791 | 0.0463245 | 0.3235065 | 1.851434 | 2.1381059 | 2.9556569 | 2.6361219 | 2.2670398 | 3.2056924 | 3.7904522 | 3.2333872 |
| ENSG00000082269.16_3 | FAM135A | chr6:711226+ | protein_codi-0.274831 | 0.8265473 | 0.0418976 | 0.3142218 | 2.1635687 | 2.4383994 | 3.9168543 | 3.5285202 | 3.038811 | 4.1168216 | 4.4925498 | 4.6665637 |
| ENSG00000082482.13_3 | KCNK2 | chr1:215179+ | protein_codi-0.524292 | 0.6953001 | 0.0373452 | 0.3070134 | 1.2064763 | 1.7307686 | 1.2985049 | 1.5137244 | 1.1271279 | 1.683881 | 2.8239997 | 2.5625314 |
| ENSG00000082512.14_2 | TRAF5 | chr1:211499+ | protein_codi-0.345555 | 0.7870049 | 0.017557 | 0.2506617 | 1.4384254 | 1.7839808 | 1.9662092 | 1.6859583 | 1.4987713 | 2.3161338 | 2.7014702 | 2.3272766 |
| ENSG00000083535.15_3 | PIBF1 | chr13:73356+ | protein_codi-0.281417 | 0.8227823 | 0.0164421 | 0.249764 | 1.3472006 | 1.6286179 | 1.5805348 | 1.6906646 | 1.3717784 | 2.0968268 | 2.2663426 | 1.9228727 |
| ENSG00000083812.11_3 | ZNF324 | chr19:58978+ | protein_codi0.4087313 | 1.3275179 | 0.0012572 | 0.1379943 | 1.9373553 | 1.5286241 | 2.8784011 | 2.5907631 | 3.0342863 | 1.9180166 | 1.890316 | 1.847423 |
| ENSG00000084207.15_2 | GSTP1 | chr11:67351+ | protein_codi0.2796048 | 1.2138623 | 0.0280637 | 0.2866074 | 7.4487946 | 7.1691898 | 172.80606 | 163.97676 | 184.97055 | 132.43714 | 141.89985 | 155.35618 |
| ENSG00000085224.21_4 | ATRX | chrX:76760- | protein_codi-0.444601 | 0.7347873 | 0.0241263 | 0.2713447 | 1.4175463 | 1.8621478 | 1.6809097 | 1.7875872 | 1.5507108 | 2.1692193 | 3.2256575 | 2.5879008 |
| ENSG00000085433.15_3 | WDR47 | chr1:109512- | protein_codi-0.308919 | 0.8072464 | 0.0491686 | 0.3287423 | 1.7974181 | 2.1063371 | 2.7641107 | 2.4717963 | 2.2137619 | 3.4570293 | 3.688946 | 2.8202453 |
| ENSG00000085662.13_2 | AKR1B1 | chr7:134127- | protein_codi0.3182418 | 1.2468102 | 0.0057151 | 0.1954316 | 5.8603614 | 5.5421195 | 59.269449 | 52.919957 | 59.337446 | 46.66953 | 44.095904 | 46.060301 |
| ENSG00000085760.14_2 | MTIF2 | chr2:554637- | protein_codi-0.282365 | 0.822242 | 0.010966 | 0.2246724 | 2.7031508 | 2.9855159 | 5.4290493 | 5.669371 | 5.4410515 | 6.363129 | 7.5161702 | 6.9228744 |
| ENSG00000085978.21_2 | ATG16L1 | chr2:234118+ | protein_codi0.6506059 | 1.5698273 | 0.0024238 | 0.1571562 | 3.1857857 | 2.5351798 | 8.4038814 | 7.0127088 | 8.9991859 | 4.8473093 | 4.7857586 | 4.7567732 |
| ENSG00000087076.8_3 | HSD17B14 | chr19:49316- | protein_codi-0.34219 | 0.7888432 | 0.0258415 | 0.2789399 | 0.3041361 | 0.6463257 | 0.1885761 | 0.2652963 | 0.2515351 | 0.7255633 | 0.3808796 | 0.6091761 |
| ENSG00000087077.13_3 | TRIP6 | chr7:100464+ | protein_codi0.2949621 | 1.2268527 | 0.0405895 | 0.3138435 | 6.0368579 | 5.7418958 | 57.78043 | 64.348774 | 72.681031 | 53.123124 | 54.100317 | 50.393911 |
| ENSG00000087095.12_2 | NLK | chr17:26368+ | protein_codi-0.419565 | 0.7476499 | 0.0078129 | 0.2082114 | 3.6855753 | 4.1051405 | 12.574038 | 11.788883 | 11.270561 | 14.786872 | 17.838079 | 16.138729 |
| ENSG00000088256.8_2 | GNA11 | chr19:30944+ | protein_codi0.3671031 | 1.2897604 | 0.0210558 | 0.2621516 | 4.1960191 | 3.828916 | 16.556936 | 16.795715 | 18.706915 | 11.743266 | 14.575094 | 13.459202 |
| ENSG00000088833.17_3 | NSFL1C | chr20:14228- | protein_codi0.2983751 | 1.2297585 | 0.0045133 | 0.1849138 | 4.777569 | 4.479194 | 27.048192 | 25.880022 | 26.367825 | 22.593582 | 21.396827 | 19.995917 |
| ENSG00000089682.16_2 | RBM41 | chrX:10630- | protein_codi-0.392294 | 0.7619174 | 0.0195308 | 0.2575012 | 1.1956663 | 1.5879598 | 1.4483455 | 1.3476058 | 1.0907227 | 1.6966595 | 2.1899609 | 2.158342 |
| ENSG00000089775.11_2 | ZBTB25 | chr14:64915- | protein_codi-0.387636 | 0.7643809 | 0.0173203 | 0.2504633 | 1.2122982 | 1.5999344 | 1.445109 | 1.4541036 | 1.0731141 | 2.260234 | 2.0103673 | 1.8380269 |
| ENSG00000090097.21_3 | PCBP4 | chr3:519914- | protein_codi-0.443483 | 0.7353571 | 0.0115479 | 0.2297054 | 2.7500175 | 3.1935006 | 5.3854733 | 5.4111136 | 6.4368101 | 9.0737739 | 7.7633705 | 7.6727138 |
| ENSG00000090266.12_4 | NDUFB2 | chr7:140390+ | protein_codi0.2751519 | 1.2101215 | 0.027325 | 0.2840194 | 3.5355138 | 3.2603619 | 11.015259 | 9.8589575 | 10.949943 | 9.486465 | 8.2575275 | 8.0630853 |
| ENSG00000090382.6_2 | LYZ | chr12:69742+ | protein_codi-0.599944 | 0.6597797 | 0.0034854 | 0.1738952 | 1.3502424 | 1.9501861 | 1.7233131 | 1.7136685 | 1.2425184 | 2.9338814 | 2.952778 | 2.7108198 |
| ENSG00000090989.17_2 | EXOC1 | chr4:567197+ | protein_codi-0.307349 | 0.8081256 | 0.0081285 | 0.2097267 | 2.3147464 | 2.622095 | 4.3094703 | 3.9544423 | 3.6814279 | 4.9139838 | 5.4260701 | 5.1399116 |
| ENSG00000091039.16_3 | OSBPL8 | chr12:76745- | protein_codi-0.387195 | 0.7646149 | 0.0342703 | 0.2993278 | 2.8578417 | 3.2450364 | 6.6307768 | 6.9236505 | 5.3007774 | 7.916687 | 9.3936473 | 8.1957752 |
| ENSG00000091640.7_2 | SPAG7 | chr17:48625- | protein_codi0.3163658 | 1.2451899 | 0.0492346 | 0.3287423 | 3.5653558 | 3.24899 | 10.796067 | 9.40446 | 12.517002 | 8.908477 | 8.4042573 | 8.2214439 |
| ENSG00000092068.19_4 | SLC7A8 | chr14:23594- | protein_codi-0.296358 | 0.8143055 | 0.0023875 | 0.1571562 | 3.0201983 | 3.3165563 | 7.0938794 | 6.8830737 | 7.3687109 | 9.4319722 | 8.5933383 | 8.8812672 |
| ENSG00000092439.13_4 | TRPM7 | chr15:50844- | protein_codi-0.412787 | 0.7511707 | 0.044772 | 0.3197072 | 2.9323225 | 3.3451098 | 7.0224342 | 7.2904032 | 5.6875839 | 8.1786699 | 10.71488 | 8.7592744 |
| ENSG00000095139.13_2 | ARCN1 | chr11:11844+ | protein_codi-0.668352 | 0.6292252 | 0.0041976 | 0.1849138 | 4.5944099 | 5.2627614 | 23.493916 | 26.086381 | 20.249873 | 34.760145 | 38.924085 | 38.638109 |
| ENSG00000095203.14_2 | EPB41L4B | chr9:111934- | protein_codi-0.302427 | 0.8108872 | 0.0014692 | 0.1390335 | 2.0784428 | 2.3808697 | 3.2971546 | 3.1778928 | 3.1964556 | 4.0179743 | 4.4685545 | 4.149186 |
| ENSG00000095637.21_3 | SORBS1 | chr10:97071- | protein_codi-0.30556 | 0.8091281 | 0.0001944 | 0.0943724 | 0.6585759 | 0.9641359 | 0.5965968 | 0.5634186 | 0.5757336 | 0.991527 | 0.967331 | 0.895125 |
| ENSG00000095951.16_3 | HIVEP1 | chr6:120089+ | protein_codi-0.815622 | 0.5681634 | 0.0413969 | 0.3138435 | 1.3001154 | 2.1157375 | 0.8818099 | 2.5528195 | 1.2334298 | 3.1881818 | 3.3537691 | 3.4648864 |
| ENSG00000096654.15_2 | ZNF184 | chr6:274185- | protein_codi-0.27669 | 0.8254827 | 0.0075213 | 0.2074702 | 1.5788666 | 1.8555567 | 2.1120163 | 2.0493222 | 1.8093991 | 2.4618324 | 2.6859445 | 2.7143246 |
| ENSG00000097096.8_2 | SYDE2 | chr1:856225- | protein_codi-0.285066 | 0.820704 | 0.013483 | 0.2374656 | 2.199374 | 2.4844401 | 3.8442144 | 3.7094325 | 3.2465936 | 4.594209 | 4.8378536 | 4.3663761 |
| ENSG00000099795.6_2 | NDUFB7 | chr19:14676- | protein_codi0.3004912 | 1.2315636 | 0.0442449 | 0.3187947 | 6.8795734 | 6.5790822 | 107.96217 | 111.26283 | 132.46337 | 98.539597 | 88.318252 | 97.303082 |
| ENSG00000099901.16_3 | RANBP1 | chr22:20103+ | protein_codi0.2814441 | 1.2154109 | 9.121E-05 | 0.0866373 | 4.9676842 | 4.6862401 | 30.731125 | 30.410742 | 29.73988 | 24.352078 | 24.86005 | 25.028765 |
| ENSG00000100029.17_2 | PES1 | chr22:30972- | protein_codi0.2771084 | 1.2117637 | 0.017127 | 0.2499474 | 4.4840461 | 4.2069378 | 20.08764 | 22.11893 | 21.988015 | 18.926142 | 17.021773 | 16.539719 |
| ENSG00000100216.5_2 | TOMM22 | chr22:39077+ | protein_codi0.2752066 | 1.2101673 | 0.0010162 | 0.1267443 | 5.5023905 | 5.227184 | 43.798972 | 43.611218 | 45.605983 | 36.333883 | 35.41417 | 37.658405 |
| ENSG00000100271.16_2 | TTLL1 | chr22:43435- | protein_codi-0.410347 | 0.7524425 | 0.0061233 | 0.2017888 | 0.6883732 | 1.09872 | 0.6275175 | 0.4878388 | 0.7281498 | 1.2393799 | 1.177014 | 1.0149024 |
| ENSG00000100485.11_2 | SOS2 | chr14:50583- | protein_codi-0.40035 | 0.7576746 | 0.0259221 | 0.2789646 | 2.1388533 | 2.5392029 | 3.6818206 | 3.6546171 | 2.9199297 | 4.3659259 | 5.4631747 | 4.6628906 |
| ENSG00000100592.15_2 | DAAM1 | chr14:59655+ | protein_codi-0.29673 | 0.8140957 | 0.045867 | 0.3226997 | 1.877182 | 2.1739117 | 3.0330269 | 2.7943116 | 2.2396654 | 3.3491054 | 3.7899679 | 3.4106782 |
| ENSG00000100596.6_3 | SPTLC2 | chr14:77972- | protein_codi-0.340408 | 0.7898181 | 0.0229976 | 0.2666812 | 3.5465963 | 3.887004 | 11.490871 | 11.148419 | 9.5143675 | 12.8345 | 14.801609 | 13.813223 |
| ENSG00000100614.17_3 | PPM1A | chr14:60712+ | protein_codi-0.526975 | 0.6940083 | 0.0263935 | 0.2809032 | 2.2831072 | 2.8100823 | 4.2588813 | 3.115484 | 4.3276656 | 5.2913497 | 6.8820907 | 5.9561979 |
| ENSG00000100784.10_3 | RPS6KA5 | chr14:91314- | protein_codi-0.36808 | 0.7748131 | 0.0101464 | 0.2182659 | 0.5873302 | 0.9554099 | 0.5320109 | 0.5565944 | 0.4222459 | 0.8393363 | 1.1350238 | 0.8567664 |
| ENSG00000100804.18_3 | PSMB5 | chr14:23485- | protein_codi0.2805127 | 1.2146265 | 0.0001957 | 0.0943724 | 5.7405609 | 5.4600482 | 52.508635 | 52.311741 | 52.579217 | 41.887544 | 44.136137 | 43.061501 |
| ENSG00000100889.11_2 | PCK2 | chr14:24563+ | protein_codi-0.76469 | 0.5885797 | 0.0085972 | 0.2113465 | 3.4349805 | 4.1996707 | 10.921502 | 8.950046 | 9.6644982 | 20.188248 | 14.27306 | 18.171649 |
| ENSG00000101000.5_3 | PROCR | chr20:33759+ | protein_codi0.316165 | 1.2450166 | 0.0095952 | 0.2144048 | 3.7372565 | 3.4210915 | 12.432326 | 12.542208 | 12.038818 | 10.646513 | 8.9548747 | 9.6003879 |
| ENSG00000101104.12_2 | PABPC1L | chr20:43538+ | protein_codi-0.320565 | 0.800756 | 0.028532 | 0.2885442 | 2.3393975 | 2.6599628 | 4.2854065 | 3.4911702 | 4.4607026 | 5.0072438 | 5.3734236 | 5.5938301 |
| ENSG00000101335.9_2 | MYL9 | chr20:35169+ | protein_codi0.4048982 | 1.3239955 | 0.0010989 | 0.130843 | 4.5430171 | 4.138119 | 21.007728 | 22.591327 | 23.402016 | 16.839828 | 16.126511 | 16.866283 |
| ENSG00000101447.14_2 | FAM83D | chr20:37554+ | protein_codi0.3102854 | 1.2399529 | 0.0005963 | 0.1106916 | 5.7136751 | 5.4033897 | 52.374072 | 52.640826 | 49.482046 | 40.753788 | 42.149908 | 41.079579 |
| ENSG00000101493.10_3 | ZNF516 | chr18:74070- | protein_codi-0.534251 | 0.6905173 | 0.0138318 | 0.2388191 | 2.2560162 | 2.7902667 | 3.5220159 | 3.8660186 | 3.9531305 | 4.864821 | 6.7122634 | 6.3185547 |
| ENSG00000101577.9_3 | LPIN2 | chr18:29169- | protein_codi-0.487731 | 0.7131456 | 3.385E-05 | 0.061109 | 3.5828547 | 4.0705861 | 11.168765 | 11.090935 | 10.693212 | 15.528964 | 15.727104 | 16.156913 |
| ENSG00000101945.16_2 | SUV39H1 | chrX:48553+ | protein_codi1.060782 | 2.086062 | 0.0002692 | 0.0943724 | 3.1963777 | 2.1355957 | 7.6178239 | 7.8522247 | 9.0964032 | 3.2013676 | 3.7205074 | 3.2781473 |
| ENSG00000101972.18_3 | STAG2 | chrX:12309+ | protein_codi-0.469805 | 0.7220624 | 0.0192383 | 0.2572494 | 3.5170499 | 3.9868545 | 10.228028 | 11.446785 | 9.7362321 | 13.546751 | 17.366752 | 13.917291 |
| ENSG00000102243.12_2 | VGLL1 | chrX:13561+ | protein_codi0.3245187 | 1.2522466 | 0.0118699 | 0.2323938 | 3.7514535 | 3.4269348 | 12.139455 | 12.023075 | 13.276086 | 10.378872 | 8.9024437 | 10.040504 |
| ENSG00000102804.14_4 | TSC22D1 | chr13:45006- | protein_codi-0.390048 | 0.7631041 | 0.0279352 | 0.2862563 | 3.2574697 | 3.6475179 | 7.8412511 | 8.5091365 | 9.4023991 | 10.353821 | 11.23985 | 13.161811 |
| ENSG00000102897.9_2 | LYRM1 | chr16:20911+ | protein_codi-0.323008 | 0.7994013 | 0.0353307 | 0.3022076 | 2.2704573 | 2.5934654 | 4.1697413 | 3.3514091 | 3.9926198 | 4.8672283 | 5.6350051 | 4.6475282 |
| ENSG00000102978.12_3 | POLR2C | chr16:57496+ | protein_codi0.263568 | 1.2004439 | 0.0242403 | 0.2722243 | 4.7070344 | 4.4434664 | 24.537313 | 25.714645 | 25.118668 | 22.444835 | 18.782196 | 21.208967 |
| ENSG00000103018.16_3 | CYB5B | chr16:69458+ | protein_codi-0.327768 | 0.7967682 | 0.0151612 | 0.2436202 | 3.5623563 | 3.8901244 | 11.766548 | 9.7203415 | 11.046093 | 13.50497 | 14.483985 | 13.512186 |
| ENSG00000103187.7_2 | COTL1 | chr16:84599- | protein_codi0.3059242 | 1.2362103 | 0.0193078 | 0.2575012 | 4.7183928 | 4.4124686 | 23.624968 | 25.497102 | 26.961418 | 22.096479 | 19.047857 | 19.856551 |
| ENSG00000103351.12_3 | CLUAP1 | chr16:35509+ | protein_codi-0.315858 | 0.8033732 | 0.0279136 | 0.2862563 | 0.8966598 | 1.2125175 | 0.8793513 | 0.6781275 | 1.0461223 | 1.1995985 | 1.441901 | 1.3170798 |
| ENSG00000103479.15_3 | RBL2 | chr16:53467+ | protein_codi-0.37127 | 0.7731016 | 0.0072249 | 0.2074372 | 2.1894117 | 2.5606818 | 3.6773556 | 3.7998577 | 3.2267548 | 4.5937894 | 5.2673973 | 4.8577756 |
| ENSG00000103852.12_3 | TTC23 | chr15:99676- | protein_codi-0.4915 | 0.7112851 | 0.0021689 | 0.1530585 | 0.9992926 | 1.4907928 | 1.0475299 | 1.0136467 | 0.9374816 | 1.5734461 | 1.9994295 | 1.8758506 |
| ENSG00000104093.13_3 | DMXL2 | chr15:51739- | protein_codi-0.419816 | 0.7475198 | 0.0321722 | 0.2958398 | 1.7311638 | 2.1509801 | 2.4184661 | 2.6116346 | 1.9638866 | 3.072926 | 4.0833771 | 3.2312606 |
| ENSG00000104133.14_4 | SPG11 | chr15:44854- | protein_codi-0.3342 | 0.7932239 | 0.0189589 | 0.2567575 | 2.2074804 | 2.5416803 | 3.7793569 | 3.9544577 | 3.160906 | 4.5475844 | 5.1413096 | 4.7943023 |
| ENSG00000104213.12_2 | PDGFRL | chr8:174339+ | protein_codi-0.278021 | 0.8247216 | 0.0247311 | 0.2747088 | 1.4644842 | 1.7425051 | 1.7981085 | 1.5287344 | 1.9702515 | 2.274412 | 2.540702 | 2.2315899 |
| ENSG00000104231.10_3 | ZFAND1 | chr8:826135- | protein_codi-0.501935 | 0.706159 | 0.006552 | 0.2040161 | 1.8768945 | 2.3788296 | 2.7893238 | 3.0090895 | 2.2613482 | 4.0398454 | 4.4809213 | 4.0936186 |
| ENSG00000104517.12_3 | UBR5 | chr8:103265- | protein_codi-0.311501 | 0.8058032 | 0.0149133 | 0.2432408 | 3.4721792 | 3.7836798 | 10.747882 | 10.473058 | 9.1403162 | 12.437592 | 13.519232 | 12.388694 |
| ENSG00000104518.10_3 | GSDMD | chr8:144635+ | protein_codi-0.356235 | 0.7812006 | 0.0093707 | 0.2136778 | 1.7125358 | 2.0687708 | 2.537769 | 2.0775776 | 2.2332214 | 3.3136782 | 2.9263767 | 3.3595981 |
| ENSG00000104765.15_3 | BNIP3L | chr8:262404+ | protein_codi-0.426004 | 0.7443205 | 0.0054571 | 0.1925603 | 2.2025847 | 2.6285888 | 3.7573617 | 3.887838 | 3.1942011 | 4.8719987 | 5.4029132 | 5.2905508 |
| ENSG00000105254.11_3 | TBCB | chr19:36605+ | protein_codi0.3269661 | 1.2543727 | 0.0025666 | 0.1603768 | 4.2162803 | 3.8893142 | 17.688609 | 17.50816 | 17.566938 | 13.66389 | 13.062794 | 14.779014 |
| ENSG00000105289.14_3 | TJP3 | chr19:37081+ | protein_codi-0.300984 | 0.8116985 | 0.0209499 | 0.2621516 | 3.5820707 | 3.8830548 | 10.690407 | 10.661364 | 11.599497 | 14.601803 | 12.340725 | 14.430988 |
| ENSG00000105426.16_3 | PTPRS | chr19:51585- | protein_codi-0.308194 | 0.8076522 | 0.0169607 | 0.2499474 | 1.6215709 | 1.9297648 | 1.9723645 | 1.982044 | 2.2870652 | 2.9885391 | 2.4961331 | 2.9659706 |
| ENSG00000105447.12_3 | GRWD1 | chr19:48949+ | protein_codi0.3150564 | 1.2440603 | 0.0068534 | 0.2047074 | 5.0153048 | 4.7002484 | 30.382038 | 30.008319 | 33.762596 | 26.090187 | 24.087871 | 24.850649 |
| ENSG00000105514.7_3 | RAB3D | chr19:11432- | protein_codi-0.276165 | 0.8257833 | 0.0035713 | 0.1753067 | 3.7629423 | 4.0391072 | 12.732582 | 12.948479 | 12.061602 | 15.620791 | 14.69907 | 16.027531 |
| ENSG00000105523.3_3 | FAM83E | chr19:49104- | protein_codi-0.277534 | 0.8249998 | 0.0017177 | 0.1452155 | 0.7525935 | 1.0301278 | 0.7129614 | 0.6473035 | 0.6948781 | 1.1168532 | 1.0562464 | 0.9567358 |
| ENSG00000105538.9_3 | RASIP1 | chr19:49223- | protein_codi-0.275793 | 0.8259964 | 0.0087076 | 0.2115899 | 2.3092392 | 2.5850317 | 3.6664186 | 3.8944557 | 4.3304355 | 5.100426 | 4.9827545 | 4.9190896 |
| ENSG00000105707.13_2 | HPN | chr19:35531+ | protein_codi-0.316583 | 0.8029696 | 0.0387413 | 0.3099894 | 1.2839637 | 1.6005465 | 1.2227103 | 1.3892501 | 1.7188813 | 2.1764073 | 1.788416 | 2.1487902 |
| ENSG00000105732.12_3 | ZNF574 | chr19:42572+ | protein_codi0.2695193 | 1.2054061 | 0.0451963 | 0.3212296 | 4.2674547 | 3.9979354 | 17.983566 | 16.999005 | 19.905921 | 16.084559 | 13.559273 | 15.396531 |
| ENSG00000105856.13_4 | HBP1 | chr7:106809+ | protein_codi-0.42608 | 0.7442812 | 0.0125373 | 0.2343664 | 1.8720054 | 2.2980857 | 2.9322284 | 2.7174222 | 2.3551252 | 3.4868041 | 4.3370648 | 3.9675079 |
| ENSG00000105865.10_2 | DUS4L | chr7:107203+ | protein_codi-0.311013 | 0.8060754 | 0.0328315 | 0.296321 | 1.199593 | 1.5106063 | 1.2417327 | 1.4200669 | 1.233208 | 1.5284942 | 2.1136252 | 1.9382257 |
| ENSG00000105976.14_3 | MET | chr7:116312+ | protein_codi-0.433619 | 0.740402 | 0.0383914 | 0.3089378 | 4.6042083 | 5.0378276 | 25.045713 | 26.264701 | 19.261772 | 29.839774 | 31.968572 | 33.865854 |
| ENSG00000106070.17_3 | GRB10 | chr7:506577- | protein_codi-0.272521 | 0.8278716 | 0.0074717 | 0.2074702 | 2.0017962 | 2.2743172 | 3.1872741 | 2.9512126 | 2.8827579 | 4.000272 | 3.5557413 | 3.9700462 |
| ENSG00000106105.13_2 | GARS | chr7:306342+ | protein_codi-0.425944 | 0.7443514 | 0.0034564 | 0.1733337 | 5.45528 | 5.8812243 | 43.538353 | 45.808694 | 39.508678 | 56.664126 | 56.6784 | 60.568109 |
| ENSG00000106261.16_2 | ZKSCAN1 | chr7:996131+ | protein_codi-0.353577 | 0.7826412 | 0.0163543 | 0.2492933 | 2.4210767 | 2.7746538 | 4.7160711 | 4.6201897 | 3.7819114 | 6.0404233 | 5.5317338 | 5.9684279 |
| ENSG00000106351.12_3 | AGFG2 | chr7:100136+ | protein_codi0.4143826 | 1.3327282 | 0.0272156 | 0.2832694 | 3.6621659 | 3.2477833 | 10.096428 | 11.53671 | 13.584737 | 8.6052603 | 7.9825863 | 8.9341351 |
| ENSG00000107242.17_3 | PIP5K1B | chr9:713203+ | protein_codi-0.478041 | 0.7179516 | 0.0034569 | 0.1733337 | 3.049492 | 3.5275334 | 7.4734712 | 7.5915935 | 6.7952599 | 9.5910891 | 11.273093 | 10.797377 |
| ENSG00000107863.17_3 | ARHGAP21 | chr10:24872- | protein_codi-0.459759 | 0.7271078 | 0.004481 | 0.1849138 | 2.3805992 | 2.840358 | 4.2934211 | 4.2945645 | 4.038817 | 6.9462113 | 5.9061467 | 5.6942653 |
| ENSG00000107968.9_2 | MAP3K8 | chr10:30722+ | protein_codi-0.302005 | 0.8111244 | 0.0170253 | 0.2499474 | 2.7767891 | 3.078794 | 6.0667245 | 5.3500982 | 6.1728521 | 6.9298878 | 8.0092622 | 7.4425134 |
| ENSG00000107984.9_2 | DKK1 | chr10:54074+ | protein_codi0.7179522 | 1.6448456 | 0.0002254 | 0.0943724 | 2.7449607 | 2.0270085 | 5.9491678 | 5.6385896 | 5.5303716 | 3.0295016 | 3.3491604 | 2.8629076 |
| ENSG00000108106.13_2 | UBE2S | chr19:55911- | protein_codi0.3810627 | 1.3023008 | 0.0286641 | 0.2885442 | 6.9901933 | 6.6091305 | 122.66218 | 116.62359 | 140.26748 | 106.25586 | 87.102903 | 97.452856 |
| ENSG00000108244.16_3 | KRT23 | chr17:39078- | protein_codi-0.641839 | 0.6408955 | 0.0008121 | 0.1165864 | 2.4580108 | 3.0998498 | 4.6093701 | 4.4031662 | 4.4732147 | 7.7701267 | 6.8230047 | 8.1846768 |
| ENSG00000108306.11_3 | FBXL20 | chr17:37408- | protein_codi-0.343265 | 0.7882554 | 0.0029057 | 0.162509 | 1.0891901 | 1.4324551 | 1.2451435 | 1.0827756 | 1.0594445 | 1.6380084 | 1.8355661 | 1.6285721 |
| ENSG00000108443.13_3 | RPS6KB1 | chr17:57970+ | protein_codi-0.340668 | 0.7896755 | 0.0312334 | 0.2933365 | 2.1580164 | 2.4986846 | 3.5855301 | 3.8168224 | 3.0246937 | 4.3278208 | 5.2275078 | 4.4409297 |
| ENSG00000108523.15_3 | RNF167 | chr17:48433+ | protein_codi0.4772276 | 1.392066 | 0.0351206 | 0.3014271 | 5.0346211 | 4.5573935 | 28.8755 | 30.516207 | 36.399588 | 22.737994 | 25.978806 | 19.383075 |
| ENSG00000108829.9_3 | LRRC59 | chr17:48452- | protein_codi0.2911663 | 1.2236291 | 0.005118 | 0.1896292 | 5.7262538 | 5.4350875 | 52.707896 | 53.596284 | 49.596592 | 42.224081 | 40.236794 | 44.432063 |
| ENSG00000108840.15_4 | HDAC5 | chr17:42154- | protein_codi-0.279203 | 0.8240462 | 0.0483655 | 0.3264562 | 1.8104443 | 2.0896472 | 2.488938 | 2.1353104 | 2.944758 | 3.4129797 | 3.2112381 | 3.1495184 |
| ENSG00000109255.11_2 | NMU | chr4:564613- | protein_codi0.4752733 | 1.3901816 | 0.0016705 | 0.1430793 | 3.198054 | 2.7227807 | 8.9538309 | 7.6417321 | 7.9854429 | 5.4870011 | 5.5677555 | 5.7523512 |
| ENSG00000109618.11_2 | SEPSECS | chr4:251216- | protein_codi-0.306012 | 0.8088744 | 0.0292565 | 0.2885442 | 1.4838747 | 1.7898871 | 1.8691263 | 1.8541332 | 1.6720738 | 2.094693 | 2.794762 | 2.5206837 |
| ENSG00000109654.14_3 | TRIM2 | chr4:154073+ | protein_codi-0.428749 | 0.7429055 | 0.0045644 | 0.1849138 | 2.0141614 | 2.4429109 | 3.3039517 | 3.063007 | 2.7692419 | 4.0739767 | 4.6588418 | 4.5987659 |
| ENSG00000109670.13_3 | FBXW7 | chr4:153242- | protein_codi-0.281032 | 0.8230021 | 0.0488826 | 0.3279747 | 2.3626219 | 2.6436539 | 4.3634611 | 4.2947961 | 3.7903338 | 4.6804859 | 5.9757777 | 5.1585609 |
| ENSG00000109686.17_4 | SH3D19 | chr4:152023- | protein_codi-0.308767 | 0.8073313 | 0.0143057 | 0.2410739 | 3.7916842 | 4.1004514 | 13.275235 | 13.621038 | 11.725374 | 16.007256 | 17.097595 | 15.399155 |
| ENSG00000109756.8_3 | RAPGEF2 | chr4:160025+ | protein_codi-0.281416 | 0.8227833 | 0.0331077 | 0.2965163 | 2.6286068 | 2.9100224 | 5.8787438 | 5.1126356 | 4.6251076 | 6.5040391 | 6.7603303 | 6.2918336 |
| ENSG00000109762.15_2 | SNX25 | chr4:186125+ | protein_codi-0.319986 | 0.8010779 | 0.0475855 | 0.3246711 | 2.0002065 | 2.320192 | 3.0549968 | 2.9619483 | 2.9853558 | 3.3801911 | 3.9530046 | 4.7409048 |
| ENSG00000109787.12_2 | KLF3 | chr4:386658+ | protein_codi-0.38205 | 0.7673467 | 0.0041 | 0.182744 | 3.3684329 | 3.7504825 | 9.248825 | 9.9220813 | 8.8405308 | 11.673228 | 13.197376 | 12.549644 |
| ENSG00000109971.13_4 | HSPA8 | chr11:12292- | protein_codi0.437058 | 1.3538407 | 0.0055241 | 0.1927541 | 7.7583762 | 7.3213182 | 226.60371 | 229.15582 | 192.78073 | 158.14358 | 159.17943 | 159.47719 |
| ENSG00000110047.17_2 | EHD1 | chr11:64619- | protein_codi0.3309237 | 1.2578184 | 0.0008353 | 0.1187924 | 4.3986018 | 4.0676781 | 20.726196 | 19.463679 | 20.104053 | 16.326304 | 15.753252 | 15.243303 |
| ENSG00000110195.12_2 | FOLR1 | chr11:71900+ | protein_codi-0.408639 | 0.7533336 | 0.0249832 | 0.2761127 | 1.8344324 | 2.2430716 | 2.2928465 | 2.5537505 | 2.8761371 | 4.3976608 | 3.3841816 | 3.4833371 |
| ENSG00000110375.2_2 | UPK2 | chr11:11879+ | protein_codi-0.588494 | 0.6650368 | 0.0083844 | 0.210538 | 1.4107627 | 1.9992567 | 1.733986 | 1.4568686 | 1.7981272 | 3.5686669 | 2.5367652 | 2.9546948 |
| ENSG00000110619.17_3 | CARS | chr11:30221- | protein_codi-0.464889 | 0.724527 | 2.85E-05 | 0.061109 | 2.8623709 | 3.3272596 | 6.3250087 | 6.2147193 | 6.2769758 | 9.0557053 | 8.7804268 | 9.2812153 |
| ENSG00000110717.12_3 | NDUFS8 | chr11:67798+ | protein_codi0.265461 | 1.2020201 | 0.0127575 | 0.2349473 | 3.8188261 | 3.553365 | 13.107156 | 12.78479 | 13.451201 | 10.138978 | 10.421468 | 11.718651 |
| ENSG00000110723.11_4 | EXPH5 | chr11:10837- | protein_codi-0.506354 | 0.7039995 | 0.0184486 | 0.2558547 | 0.9990581 | 1.5054117 | 1.0952498 | 0.7768212 | 1.1446666 | 1.7827953 | 2.2309594 | 1.5451269 |
| ENSG00000110871.14_3 | COQ5 | chr12:12094- | protein_codi-0.317642 | 0.8023804 | 0.0066839 | 0.2040161 | 2.5287254 | 2.8463672 | 4.5926437 | 4.8730106 | 4.8504421 | 5.6616763 | 6.6069045 | 6.3406459 |
| ENSG00000111057.10_3 | KRT18 | chr12:53342+ | protein_codi0.3107442 | 1.2403473 | 0.0018661 | 0.1515851 | 8.215847 | 7.9051028 | 293.6334 | 288.3222 | 307.30843 | 245.03083 | 228.14031 | 243.30265 |
| ENSG00000111144.9_2 | LTA4H | chr12:96394- | protein_codi-0.274029 | 0.827007 | 0.0169426 | 0.2499474 | 3.632557 | 3.9065856 | 12.60811 | 11.041027 | 10.642989 | 13.739608 | 14.165334 | 14.088999 |
| ENSG00000111252.10_2 | SH2B3 | chr12:11184+ | protein_codi-0.900698 | 0.5356274 | 6.676E-05 | 0.0781476 | 2.2602627 | 3.1609611 | 3.7118523 | 3.8030342 | 3.8586238 | 8.1309595 | 7.3520627 | 8.382577 |
| ENSG00000111266.8_3 | DUSP16 | chr12:12627- | protein_codi-0.309343 | 0.8070089 | 0.0002995 | 0.0943724 | 4.2083974 | 4.5177409 | 17.715093 | 17.187253 | 17.561047 | 21.312156 | 22.589193 | 21.838729 |
| ENSG00000111269.2_2 | CREBL2 | chr12:12764+ | protein_codi-0.33485 | 0.7928666 | 0.0289703 | 0.2885442 | 3.2535601 | 3.5884101 | 9.54671 | 8.6036974 | 7.5645012 | 10.217242 | 11.454674 | 11.457726 |
| ENSG00000111276.10_3 | CDKN1B | chr12:12867+ | protein_codi-0.269875 | 0.8293912 | 0.0050805 | 0.1896292 | 5.0635767 | 5.3334521 | 32.77516 | 32.513652 | 32.040475 | 40.562013 | 40.777848 | 36.752401 |
| ENSG00000111335.12_2 | OAS2 | chr12:11341+ | protein_codi0.7123492 | 1.6384699 | 0.000381 | 0.0950529 | 2.5360164 | 1.8236672 | 4.9108008 | 4.6489723 | 4.8429911 | 2.3124707 | 2.8320319 | 2.4942536 |
| ENSG00000111371.15_3 | SLC38A1 | chr12:46576- | protein_codi-0.542645 | 0.6865111 | 0.0095229 | 0.2139 | 4.2256206 | 4.7682657 | 19.235453 | 18.963314 | 15.209519 | 25.297808 | 28.316689 | 25.250559 |
| ENSG00000111665.11_2 | CDCA3 | chr12:69539- | protein_codi0.31856 | 1.2470851 | 0.0089868 | 0.2129568 | 3.458775 | 3.1402151 | 9.5631662 | 9.6536092 | 10.811206 | 7.7633459 | 7.4038315 | 8.3057098 |
| ENSG00000111731.12_3 | C2CD5 | chr12:22601- | protein_codi-0.388643 | 0.7638475 | 0.0310069 | 0.2925182 | 2.0674355 | 2.4560789 | 3.4857148 | 3.3167303 | 2.8027146 | 3.9508827 | 5.2021174 | 4.380686 |
| ENSG00000111885.6_3 | MAN1A1 | chr6:119498- | protein_codi-0.389011 | 0.7636529 | 0.0183887 | 0.2558547 | 1.9455431 | 2.3345541 | 3.179049 | 3.066041 | 2.363189 | 3.908889 | 4.188368 | 4.038467 |
| ENSG00000111911.6_2 | HINT3 | chr6:126277+ | protein_codi-0.355066 | 0.7818338 | 0.0307924 | 0.2917611 | 1.8295795 | 2.1846456 | 2.934741 | 2.674532 | 2.10568 | 3.310882 | 3.635187 | 3.702168 |
| ENSG00000111912.19_4 | NCOA7 | chr6:126102+ | protein_codi-0.535523 | 0.6899085 | 0.0343429 | 0.2993278 | 1.3556693 | 1.8911924 | 1.7572411 | 1.8889508 | 1.1041489 | 2.7907967 | 3.0919636 | 2.2904451 |
| ENSG00000112110.9_2 | MRPL18 | chr6:160210+ | protein_codi0.2736429 | 1.2088564 | 0.0043242 | 0.1849138 | 5.3119358 | 5.0382929 | 37.596675 | 37.852818 | 40.800705 | 31.937063 | 32.988072 | 30.697168 |
| ENSG00000112367.10_2 | FIG4 | chr6:110012+ | protein_codi-0.277741 | 0.8248816 | 0.0249248 | 0.2761127 | 2.1642415 | 2.4419825 | 3.5915796 | 3.6449043 | 3.2224642 | 3.9643132 | 4.5961036 | 4.7754393 |
| ENSG00000112406.4_2 | HECA | chr6:139456+ | protein_codi-0.437596 | 0.7383637 | 0.0155398 | 0.2460663 | 2.4483926 | 2.885989 | 5.029293 | 4.474683 | 3.925989 | 5.966272 | 7.112499 | 6.147475 |
| ENSG00000112494.9_2 | UNC93A | chr6:167684+ | protein_codi-0.491426 | 0.7113217 | 0.0119265 | 0.2323938 | 0.6320914 | 1.1235172 | 0.6574723 | 0.5505062 | 0.4484904 | 1.3352124 | 0.903847 | 1.3263697 |
| ENSG00000112715.21_4 | VEGFA | chr6:437379+ | protein_codi-0.455544 | 0.7292349 | 0.0001703 | 0.0943724 | 2.9560055 | 3.4115499 | 6.5553582 | 6.6350398 | 7.0997678 | 9.5499664 | 9.5522772 | 9.8227919 |
| ENSG00000112759.16_2 | SLC29A1 | chr6:441872+ | protein_codi0.3193571 | 1.2477744 | 0.0010465 | 0.1292718 | 3.3850164 | 3.0656593 | 9.1067226 | 9.7503482 | 9.4940119 | 7.2973289 | 7.1483583 | 7.6807606 |
| ENSG00000113407.13_3 | TARS | chr5:334408+ | protein_codi-0.357001 | 0.7807858 | 0.0291106 | 0.2885442 | 4.6025918 | 4.9595931 | 25.927782 | 23.461735 | 20.770415 | 28.452301 | 32.773639 | 29.287388 |
| ENSG00000114739.13_2 | ACVR2B | chr3:384953+ | protein_codi-0.390954 | 0.762625 | 0.0024319 | 0.1571562 | 1.7444088 | 2.1353631 | 2.5031639 | 2.3974379 | 2.1604299 | 3.3198617 | 3.6196872 | 3.2495469 |
| ENSG00000114742.13_3 | WDR48 | chr3:390934+ | protein_codi-0.295239 | 0.8149375 | 0.0270629 | 0.2827449 | 2.4367133 | 2.731952 | 4.8312731 | 4.3674604 | 4.0703641 | 5.1582287 | 6.1778449 | 5.63359 |
| ENSG00000114744.8_2 | COMMD2 | chr3:149456- | protein_codi-0.310849 | 0.806167 | 0.0491236 | 0.3287223 | 2.2076656 | 2.5185149 | 3.829063 | 3.9448817 | 3.1276413 | 4.1815454 | 4.8777537 | 5.1769622 |
| ENSG00000114796.15_2 | KLHL24 | chr3:183353+ | protein_codi-0.326999 | 0.7971928 | 0.0007365 | 0.114143 | 0.4140699 | 0.7410692 | 0.3571001 | 0.3556803 | 0.2857994 | 0.6205664 | 0.7148791 | 0.6801647 |
| ENSG00000114861.18_3 | FOXP1 | chr3:710038- | protein_codi-0.416428 | 0.7492774 | 0.0051407 | 0.1896292 | 2.8837594 | 3.3001876 | 6.6569017 | 6.7789855 | 5.7502244 | 8.3688454 | 9.2343555 | 8.9682708 |
| ENSG00000115350.11_2 | POLE4 | chr2:751856+ | protein_codi0.333925 | 1.2604379 | 0.0044949 | 0.1849138 | 2.401822 | 2.067897 | 3.897191 | 4.4822151 | 4.497412 | 3.2967698 | 3.117191 | 3.166333 |
| ENSG00000115386.5_2 | REG1A | chr2:793474+ | protein_codi-0.543648 | 0.6860338 | 4.646E-05 | 0.061109 | 3.3713728 | 3.9150211 | 9.4103596 | 9.4897919 | 9.1489326 | 13.707716 | 13.968232 | 14.591957 |
| ENSG00000115457.9_3 | IGFBP2 | chr2:217497+ | protein_codi-0.281783 | 0.8225737 | 0.0128957 | 0.2354662 | 2.0582055 | 2.3399887 | 2.9451283 | 3.037899 | 3.5344878 | 4.2013724 | 3.9337954 | 4.0573298 |
| ENSG00000115548.16_2 | KDM3A | chr2:866677+ | protein_codi-0.339554 | 0.7902857 | 0.0049865 | 0.1891494 | 1.5000198 | 1.8395737 | 1.9307031 | 1.7902014 | 1.7672315 | 2.3698735 | 2.8396151 | 2.5432294 |
| ENSG00000115758.12_3 | ODC1 | chr2:105800- | protein_codi0.285677 | 1.2189821 | 0.0004751 | 0.1106916 | 6.2590451 | 5.9733681 | 76.551932 | 76.532648 | 73.714296 | 61.785675 | 60.307812 | 63.433624 |
| ENSG00000115902.10_2 | SLC1A4 | chr2:652156+ | protein_codi-0.625962 | 0.6479875 | 0.0073622 | 0.2074702 | 1.2834387 | 1.9094008 | 1.5225083 | 1.3814588 | 1.40096 | 3.0301142 | 2.1750452 | 3.1427955 |
| ENSG00000116030.16_2 | SUMO1 | chr2:203070- | protein_codi-0.283223 | 0.8217532 | 0.018242 | 0.2547056 | 3.7994736 | 4.0826966 | 13.239735 | 13.578185 | 12.003527 | 17.250799 | 15.32957 | 15.322465 |
| ENSG00000116151.13_4 | MORN1 | chr1:225269- | protein_codi-0.281129 | 0.8229467 | 0.002048 | 0.1522433 | 0.7769896 | 1.0581187 | 0.6552235 | 0.8037665 | 0.685212 | 1.1153469 | 1.0663788 | 1.0653083 |
| ENSG00000116191.17_3 | RALGPS2 | chr1:178694+ | protein_codi-0.353205 | 0.7828433 | 0.0489882 | 0.3285381 | 1.7897832 | 2.1429879 | 2.8757164 | 2.5941868 | 1.9674451 | 3.1135998 | 3.6495609 | 3.5048145 |
| ENSG00000116285.12_2 | ERRFI1 | chr1:806446- | protein_codi-0.706955 | 0.6126117 | 0.0001139 | 0.0866373 | 4.3865411 | 5.0934963 | 20.942537 | 20.076106 | 18.786277 | 33.028555 | 33.939052 | 32.475797 |
| ENSG00000116497.17_3 | S100PBP | chr1:332823+ | protein_codi-0.269832 | 0.829416 | 0.0335841 | 0.2981619 | 1.8353126 | 2.1051448 | 2.6117379 | 2.7349116 | 2.3686501 | 2.8900598 | 3.5172711 | 3.5321343 |
| ENSG00000116574.5_2 | RHOU | chr1:228870+ | protein_codi-0.346382 | 0.7865541 | 0.0095445 | 0.2139 | 3.9832053 | 4.3295874 | 15.892999 | 15.378016 | 13.296336 | 19.287148 | 19.023981 | 19.009472 |
| ENSG00000116668.12_3 | SWT1 | chr1:185126+ | protein_codi-0.371114 | 0.7731853 | 0.0470513 | 0.3240376 | 1.0220464 | 1.3931604 | 1.1193356 | 1.2568895 | 0.7510151 | 1.4837577 | 1.8961479 | 1.5189482 |
| ENSG00000116701.14_2 | NCF2 | chr1:183524- | protein_codi0.2813023 | 1.2152914 | 0.0227271 | 0.2664462 | 1.9078932 | 1.6265909 | 2.7209089 | 2.9408958 | 2.6037552 | 2.0929864 | 1.8421167 | 2.3491766 |
| ENSG00000116761.11_2 | CTH | chr1:708769+ | protein_codi-1.012436 | 0.4957084 | 0.0015926 | 0.1419323 | 1.8598311 | 2.8722675 | 2.8190692 | 2.9516057 | 2.1685731 | 7.2592196 | 5.751774 | 6.0397805 |
| ENSG00000116852.14_2 | KIF21B | chr1:200938- | protein_codi-0.751002 | 0.5941909 | 0.0132795 | 0.2355902 | 1.0992341 | 1.8502357 | 1.1582765 | 1.0543424 | 1.2178256 | 3.2894484 | 1.8585419 | 2.8228153 |
| ENSG00000116990.10_3 | MYCL | chr1:403610- | protein_codi-0.322199 | 0.7998498 | 0.0170075 | 0.2499474 | 0.7919486 | 1.1141476 | 0.7047493 | 0.6219091 | 0.8772139 | 1.3277068 | 1.0598659 | 1.1154771 |
| ENSG00000117318.8_2 | ID3 | chr1:238844- | protein_codi0.4041593 | 1.3233176 | 0.0090398 | 0.2129568 | 4.9165778 | 4.5124185 | 28.07927 | 27.937136 | 31.739584 | 23.606927 | 20.188432 | 21.801518 |
| ENSG00000117399.13_2 | CDC20 | chr1:438246+ | protein_codi0.4012032 | 1.3206088 | 0.002065 | 0.1522433 | 7.1919334 | 6.7907302 | 144.82446 | 142.89502 | 147.96591 | 117.84221 | 103.45677 | 108.32844 |
| ENSG00000117410.13_3 | ATP6V0B | chr1:444401+ | protein_codi0.3013626 | 1.2323078 | 0.0103196 | 0.2199347 | 3.5449325 | 3.2435698 | 10.733842 | 10.090376 | 11.218179 | 9.104827 | 7.9172848 | 8.4291801 |
| ENSG00000117632.22_3 | STMN1 | chr1:262106- | protein_codi-0.456279 | 0.7288637 | 0.0003116 | 0.0943724 | 4.160469 | 4.616748 | 17.006573 | 17.443245 | 16.219063 | 22.888091 | 24.406766 | 23.333694 |
| ENSG00000117676.13_3 | RPS6KA1 | chr1:268562+ | protein_codi0.2686327 | 1.2046656 | 0.0210681 | 0.2621516 | 4.1030169 | 3.8343842 | 16.652601 | 17.292343 | 14.715038 | 13.874975 | 13.040813 | 12.897781 |
| ENSG00000118263.14_3 | KLF7 | chr2:207938- | protein_codi-0.292888 | 0.8162665 | 0.023633 | 0.2689009 | 0.9926343 | 1.2855222 | 1.1152155 | 0.9254618 | 0.9344102 | 1.2928047 | 1.6807026 | 1.3568207 |
| ENSG00000118482.11_2 | PHF3 | chr6:643457+ | protein_codi-0.475813 | 0.7190617 | 0.0430068 | 0.31611 | 2.9751409 | 3.4509535 | 7.3627979 | 7.6764781 | 5.7007725 | 9.026942 | 11.796646 | 9.1919404 |
| ENSG00000118523.5_2 | CTGF | chr6:132269- | protein_codi0.6003698 | 1.5161051 | 0.0002021 | 0.0943724 | 2.8080418 | 2.207672 | 5.869455 | 5.992358 | 6.151028 | 3.863107 | 3.621427 | 3.385676 |
| ENSG00000118762.7_2 | PKD2 | chr4:889288+ | protein_codi-0.281865 | 0.822527 | 0.0190544 | 0.257049 | 2.0376466 | 2.3195118 | 3.4100401 | 2.7726197 | 3.1599939 | 3.7910333 | 4.2023011 | 3.9900302 |
| ENSG00000118961.14_3 | LDAH | chr2:208837- | protein_codi-0.338232 | 0.7910099 | 0.0315712 | 0.2945658 | 1.786221 | 2.1244534 | 2.718443 | 2.5771207 | 2.0847757 | 3.489998 | 3.6217501 | 2.9950291 |
| ENSG00000119138.4_2 | KLF9 | chr9:729995- | protein_codi-0.62496 | 0.648438 | 0.0031561 | 0.1682903 | 0.7055277 | 1.3304872 | 0.713148 | 0.584421 | 0.597679 | 1.772191 | 1.231541 | 1.571116 |
| ENSG00000119326.14_3 | CTNNAL1 | chr9:111704- | protein_codi-0.391866 | 0.7621432 | 0.0415701 | 0.3140442 | 4.8211031 | 5.2129692 | 27.983461 | 29.951871 | 24.179832 | 31.569533 | 40.133153 | 37.086998 |
| ENSG00000119699.7_2 | TGFB3 | chr14:76424- | protein_codi-0.540906 | 0.6873394 | 0.0066785 | 0.2040161 | 1.6684622 | 2.2093677 | 2.1262223 | 2.4182792 | 2.0056894 | 4.2312303 | 3.3871539 | 3.3099392 |
| ENSG00000119950.20_4 | MXI1 | chr10:11196+ | protein_codi-0.495999 | 0.7090703 | 0.0291899 | 0.2885442 | 1.4754096 | 1.971409 | 2.2527561 | 1.4073902 | 1.7455517 | 2.6783925 | 2.7424328 | 3.380742 |
| ENSG00000120008.15_2 | WDR11 | chr10:12261+ | protein_codi-0.282513 | 0.8221577 | 0.0226533 | 0.2664462 | 2.308926 | 2.5914389 | 3.9954196 | 4.300768 | 3.5947 | 4.99276 | 5.4216104 | 4.6889449 |
| ENSG00000120068.6_3 | HOXB8 | chr17:46688- | protein_codi-0.295638 | 0.814712 | 0.0423783 | 0.3149427 | 0.8157461 | 1.111384 | 0.8151716 | 0.6110556 | 0.8649358 | 1.269804 | 1.2874437 | 0.9424102 |
| ENSG00000120262.9_2 | CCDC170 | chr6:151815+ | protein_codi-0.3162 | 0.8031828 | 0.0045334 | 0.1849138 | 1.6039683 | 1.9201681 | 1.9136438 | 2.2582198 | 1.9587679 | 2.692301 | 2.8901417 | 2.7741753 |
| ENSG00000120370.12_3 | GORAB | chr1:170501+ | protein_codi-0.401579 | 0.7570294 | 0.0416778 | 0.3140442 | 1.3909509 | 1.7925296 | 1.9242832 | 1.5607903 | 1.4085759 | 2.4525636 | 2.9493483 | 2.0489369 |
| ENSG00000120727.12_3 | PAIP2 | chr5:138677+ | protein_codi-0.441569 | 0.7363336 | 0.0182142 | 0.2547056 | 3.7964778 | 4.2380464 | 12.343903 | 12.047574 | 14.408077 | 15.828788 | 19.601374 | 18.381537 |
| ENSG00000121073.13_2 | SLC35B1 | chr17:47778- | protein_codi0.2672688 | 1.2035273 | 0.0070989 | 0.2060586 | 2.9123703 | 2.6451015 | 6.5556265 | 6.2037506 | 6.8397699 | 5.2861745 | 4.9555306 | 5.5381936 |
| ENSG00000121075.9_2 | TBX4 | chr17:59529+ | protein_codi0.286987 | 1.2200896 | 0.0214341 | 0.2627512 | 0.6866162 | 0.3996291 | 0.6530252 | 0.4995149 | 0.6820772 | 0.4310961 | 0.2717433 | 0.2613419 |
| ENSG00000121851.12_3 | POLR3GL | chr1:145456- | protein_codi-0.295197 | 0.8149609 | 0.0387461 | 0.3099894 | 1.358473 | 1.6536703 | 1.3737983 | 1.8417665 | 1.4991435 | 2.0133487 | 2.4162294 | 2.025636 |
| ENSG00000122711.8_2 | SPINK4 | chr9:332183+ | protein_codi0.4835064 | 1.3981377 | 0.0181474 | 0.2544433 | 1.3876417 | 0.9041353 | 1.6216858 | 1.9397121 | 1.3242341 | 1.0792717 | 0.7363048 | 0.815425 |
| ENSG00000122779.17_3 | TRIM24 | chr7:138145+ | protein_codi-0.294162 | 0.8155457 | 0.0394179 | 0.3123648 | 3.6419935 | 3.9361559 | 12.337314 | 11.92033 | 10.290312 | 13.429014 | 15.69715 | 13.887576 |
| ENSG00000122786.19_3 | CALD1 | chr7:134429+ | protein_codi-0.507221 | 0.7035762 | 0.0096649 | 0.2153308 | 2.5208344 | 3.0280557 | 4.4955844 | 4.6937118 | 5.0413073 | 6.2657657 | 8.2645184 | 7.0631096 |
| ENSG00000123106.10_3 | CCDC91 | chr12:28286+ | protein_codi-0.335403 | 0.7925629 | 0.0045109 | 0.1849138 | 1.5261187 | 1.8615214 | 1.9888615 | 1.8001126 | 1.8545736 | 2.4080409 | 2.8513788 | 2.6559508 |
| ENSG00000123136.14_2 | DDX39A | chr19:14519- | protein_codi0.2719608 | 1.2074478 | 0.0006683 | 0.114143 | 4.9870603 | 4.7150995 | 30.357853 | 30.053907 | 31.756814 | 25.700676 | 24.728154 | 25.377174 |
| ENSG00000123179.13_2 | EBPL | chr13:50234- | protein_codi0.6225872 | 1.5396338 | 0.0479834 | 0.3253803 | 3.7104673 | 3.0878801 | 13.413955 | 8.942992 | 14.652569 | 7.8360585 | 6.5145705 | 8.2570119 |
| ENSG00000123405.13_2 | NFE2 | chr12:54685- | protein_codi-0.383075 | 0.7668014 | 0.0109515 | 0.2246724 | 1.4069813 | 1.7900564 | 1.7562149 | 1.6133685 | 1.5889101 | 2.8377548 | 2.4008561 | 2.168958 |
| ENSG00000123416.15_3 | TUBA1B | chr12:49521- | protein_codi0.3695661 | 1.2919642 | 0.0026363 | 0.1614437 | 7.9778656 | 7.6082994 | 262.7828 | 251.94664 | 239.13481 | 201.84194 | 196.82312 | 184.15875 |
| ENSG00000123684.12_2 | LPGAT1 | chr1:211916- | protein_codi-0.286724 | 0.8197612 | 0.0227202 | 0.2664462 | 2.5245568 | 2.8112813 | 5.0746527 | 5.0170122 | 4.2119428 | 5.75421 | 6.3415653 | 5.9739165 |
| ENSG00000123836.14_3 | PFKFB2 | chr1:207207+ | protein_codi-0.279465 | 0.8238963 | 0.0322785 | 0.2958398 | 2.5588577 | 2.838323 | 5.2928679 | 4.9625902 | 4.4524935 | 5.8655197 | 6.7979072 | 5.8329708 |
| ENSG00000123843.12_3 | C4BPB | chr1:207262+ | protein_codi0.3036559 | 1.2342682 | 0.008581 | 0.2112902 | 3.3497744 | 3.0461185 | 9.9151704 | 8.6573729 | 9.0521321 | 7.525717 | 7.4034162 | 6.8656083 |
| ENSG00000124107.5_2 | SLPI | chr20:43880- | protein_codi-0.315298 | 0.8036847 | 0.0066925 | 0.2040161 | 4.5892154 | 4.9045138 | 22.107439 | 22.084358 | 25.145977 | 29.500612 | 28.638376 | 28.720381 |
| ENSG00000124177.14_3 | CHD6 | chr20:40030- | protein_codi-0.279038 | 0.8241402 | 0.0035371 | 0.1749275 | 2.8971081 | 3.1761463 | 6.5598999 | 6.6940349 | 6.1068815 | 7.8296187 | 8.4099755 | 7.8882343 |
| ENSG00000124602.9_3 | UNC5CL | chr6:409946- | protein_codi0.2638188 | 1.2006526 | 0.0186709 | 0.2558547 | 2.7673906 | 2.5035718 | 5.3172811 | 5.7841677 | 6.3650548 | 4.7424613 | 4.8006981 | 4.4748509 |
| ENSG00000124733.3_2 | MEA1 | chr6:429798- | protein_codi0.2652758 | 1.2018658 | 0.0311328 | 0.2933365 | 6.0478645 | 5.7825887 | 61.672112 | 63.285862 | 70.874702 | 57.704964 | 50.535778 | 54.133339 |
| ENSG00000124762.13_2 | CDKN1A | chr6:366442+ | protein_codi0.3069025 | 1.2370489 | 0.0146788 | 0.242791 | 6.3098062 | 6.0029037 | 77.726856 | 73.608576 | 83.998481 | 67.708784 | 60.182424 | 61.736937 |
| ENSG00000125247.15_3 | TMTC4 | chr13:10125- | protein_codi-0.279022 | 0.8241494 | 0.001962 | 0.1522433 | 1.5779684 | 1.8569906 | 1.9128847 | 2.0461005 | 1.9990237 | 2.4876176 | 2.7805812 | 2.6053065 |
| ENSG00000125285.5_3 | SOX21 | chr13:95361- | protein_codi-0.289921 | 0.8179468 | 0.0195708 | 0.2575012 | 0.379367 | 0.6692881 | 0.34892 | 0.31675 | 0.239119 | 0.731539 | 0.47279 | 0.577082 |
| ENSG00000125482.12_2 | TTF1 | chr9:135250- | protein_codi-0.311193 | 0.8059753 | 0.0224667 | 0.2659269 | 2.2753027 | 2.5864953 | 3.691091 | 4.3403884 | 3.528521 | 5.2545064 | 5.149308 | 4.634024 |
| ENSG00000125772.12_3 | GPCPD1 | chr20:55250- | protein_codi-0.690191 | 0.6197718 | 0.0045648 | 0.1849138 | 1.2729777 | 1.9631687 | 1.4754876 | 1.680843 | 1.1265776 | 2.5628763 | 3.2027711 | 2.9589636 |
| ENSG00000125826.20_3 | RBCK1 | chr20:38814+ | protein_codi-0.327778 | 0.7967627 | 0.0045523 | 0.1849138 | 4.1314652 | 4.4592431 | 16.008628 | 15.817268 | 17.821754 | 21.716027 | 20.462175 | 20.831908 |
| ENSG00000125966.9_2 | MMP24 | chr20:33814+ | protein_codi0.3109918 | 1.2405603 | 0.0087013 | 0.2115899 | 2.0007078 | 1.689716 | 2.853582 | 3.062462 | 3.094164 | 2.363213 | 1.973364 | 2.357091 |
| ENSG00000125968.8_2 | ID1 | chr20:30193+ | protein_codi0.3169983 | 1.2457359 | 0.013104 | 0.2354662 | 7.8071726 | 7.4901743 | 227.24776 | 209.49889 | 232.84229 | 191.52621 | 166.24396 | 179.49331 |
| ENSG00000125977.6_2 | EIF2S2 | chr20:32676- | protein_codi-0.323934 | 0.7988883 | 0.034078 | 0.2993278 | 5.7087527 | 6.032687 | 56.450195 | 52.678387 | 45.390224 | 60.375328 | 67.113167 | 66.117279 |
| ENSG00000127328.21_3 | RAB3IP | chr12:70132+ | protein_codi-0.283002 | 0.8218793 | 0.0005494 | 0.1106916 | 1.3077432 | 1.5907447 | 1.4256282 | 1.4764672 | 1.5255325 | 1.9422998 | 2.1027125 | 1.9933479 |
| ENSG00000127947.15_2 | PTPN12 | chr7:771665+ | protein_codi-0.544134 | 0.6858032 | 0.0413379 | 0.3138435 | 3.3130244 | 3.857158 | 11.179973 | 8.7781403 | 7.2424554 | 12.113563 | 15.050658 | 13.459296 |
| ENSG00000127980.15_2 | PEX1 | chr7:921163- | protein_codi-0.386944 | 0.7647479 | 0.001619 | 0.1419323 | 1.771948 | 2.1588919 | 2.5344839 | 2.4554304 | 2.261372 | 3.6596262 | 3.2597329 | 3.4868363 |
| ENSG00000128165.8_2 | ADM2 | chr22:50919+ | protein_codi-1.219747 | 0.4293579 | 0.0011109 | 0.130843 | 2.1128177 | 3.332565 | 3.2146918 | 3.311377 | 3.4533078 | 10.030809 | 7.255686 | 10.22653 |
| ENSG00000128228.4_2 | SDF2L1 | chr22:21996+ | protein_codi0.3328095 | 1.2594637 | 0.0047294 | 0.1853643 | 5.7200488 | 5.3872393 | 50.792889 | 50.089026 | 54.350438 | 42.768257 | 38.320343 | 41.597619 |
| ENSG00000128272.14_2 | ATF4 | chr22:39915+ | protein_codi-0.391584 | 0.7622923 | 0.0003033 | 0.0943724 | 6.9079322 | 7.2995159 | 118.83834 | 119.51877 | 118.90406 | 157.84238 | 149.82969 | 162.17982 |
| ENSG00000128298.16_2 | BAIAP2L2 | chr22:38480- | protein_codi-0.358777 | 0.7798252 | 0.0065867 | 0.2040161 | 0.3658473 | 0.7246246 | 0.2432053 | 0.2480152 | 0.3792098 | 0.7518994 | 0.653173 | 0.5580226 |
| ENSG00000128510.10_2 | CPA4 | chr7:129932+ | protein_codi0.6815184 | 1.6038268 | 0.0014758 | 0.1390335 | 2.2568813 | 1.5753629 | 3.8419937 | 3.8214178 | 3.6770057 | 1.9756999 | 1.6890171 | 2.3075907 |
| ENSG00000128626.11_2 | MRPS12 | chr19:39421+ | protein_codi0.2797807 | 1.2140104 | 0.0094204 | 0.2138428 | 5.3335476 | 5.0537669 | 38.708736 | 37.238703 | 42.180159 | 33.312197 | 30.970735 | 32.404511 |
| ENSG00000128683.13_3 | GAD1 | chr2:171669+ | protein_codi-0.362894 | 0.7776033 | 0.0195952 | 0.2575012 | 1.8098902 | 2.1727839 | 2.3203867 | 2.4630025 | 2.7484542 | 3.1539336 | 4.0242041 | 3.3923056 |
| ENSG00000128709.12_3 | HOXD9 | chr2:176987+ | protein_codi4.8238177 | 28.321341 | 4.53E-07 | 0.0068937 | 4.8578948 | 0.0340772 | 26.581068 | 26.364252 | 31.308851 | 0 | 0.041294 | 0.030864 |
| ENSG00000128833.12_2 | MYO5C | chr15:52484- | protein_codi-0.289888 | 0.8179656 | 0.0331664 | 0.2967039 | 2.5731125 | 2.8630003 | 5.3348991 | 5.1424443 | 4.4159071 | 6.0151733 | 6.8928243 | 5.9546632 |
| ENSG00000128965.11_2 | CHAC1 | chr15:41245+ | protein_codi-1.772751 | 0.2926502 | 0.0004895 | 0.1106916 | 3.3129247 | 5.0856756 | 7.9615295 | 11.467207 | 7.7845151 | 33.825059 | 30.733331 | 34.43359 |
| ENSG00000129003.15_3 | VPS13C | chr15:62144- | protein_codi-0.645048 | 0.6394716 | 0.0326509 | 0.2958398 | 2.9611368 | 3.6061846 | 7.4948473 | 7.5215991 | 5.5237348 | 9.7904619 | 14.094495 | 10.087933 |
| ENSG00000129292.20_3 | PHF20L1 | chr8:133787+ | protein_codi-0.302705 | 0.810731 | 0.0425243 | 0.3149427 | 1.1517031 | 1.4544079 | 1.3747264 | 1.3253043 | 0.9860864 | 1.5580469 | 1.9731174 | 1.7060801 |
| ENSG00000129493.14_3 | HEATR5A | chr14:31760- | protein_codi-0.280563 | 0.8232695 | 0.0421592 | 0.3149427 | 3.0438264 | 3.3243896 | 7.7959717 | 7.6615703 | 6.3615402 | 8.714265 | 9.7084469 | 8.6624346 |
| ENSG00000129911.8_2 | KLF16 | chr19:18523- | protein_codi0.4627908 | 1.3782053 | 0.0431989 | 0.3166579 | 4.838142 | 4.3753512 | 28.651246 | 22.611756 | 32.427687 | 21.399432 | 18.481278 | 19.487142 |
| ENSG00000129968.15_2 | ABHD17A | chr19:18768- | protein_codi0.6990661 | 1.6234535 | 0.0040381 | 0.182744 | 3.4291296 | 2.7300635 | 9.6079428 | 9.3301405 | 10.404472 | 6.7220851 | 5.0897284 | 5.2109852 |
| ENSG00000130066.16_2 | SAT1 | chrX:23801+ | protein_codi-0.313697 | 0.8045772 | 0.0048562 | 0.1875771 | 5.0838733 | 5.3975706 | 32.477711 | 33.501327 | 32.775638 | 38.388371 | 43.829067 | 41.419376 |
| ENSG00000130165.10_3 | ELOF1 | chr19:11661- | protein_codi0.3531001 | 1.2773024 | 0.0087711 | 0.2115899 | 3.3389236 | 2.9858235 | 9.3898216 | 8.4509687 | 9.5502807 | 7.2063457 | 7.2488865 | 6.3438222 |
| ENSG00000130204.12_4 | TOMM40 | chr19:45393+ | protein_codi0.342991 | 1.2683835 | 0.0049547 | 0.1885042 | 5.9399811 | 5.59699 | 58.433903 | 57.834018 | 65.172059 | 49.256309 | 46.070981 | 46.933795 |
| ENSG00000130303.12_3 | BST2 | chr19:17513- | protein_codi1.1395198 | 2.2030769 | 0.0003594 | 0.0943724 | 3.324984 | 2.1854642 | 9.5614639 | 8.4778116 | 9.0537386 | 3.147524 | 4.1316318 | 3.4220783 |
| ENSG00000130513.6_3 | GDF15 | chr19:18485+ | protein_codi-0.288407 | 0.8188056 | 0.0413657 | 0.3138435 | 6.4048702 | 6.6932773 | 83.598726 | 77.033326 | 91.157396 | 107.42091 | 93.138839 | 107.58001 |
| ENSG00000130600.17_3 | H19 | chr11:20163- | processed_tr-0.886962 | 0.5407516 | 0.0048109 | 0.1867542 | 2.3078201 | 3.1947821 | 3.7320206 | 3.4825349 | 4.7226723 | 8.0096936 | 9.5863483 | 7.0485856 |
| ENSG00000130706.12_3 | ADRM1 | chr20:60877+ | protein_codi0.369253 | 1.2916838 | 0.0092274 | 0.2129568 | 7.7598329 | 7.39058 | 212.132 | 207.80408 | 227.79144 | 180.05124 | 153.10951 | 168.32763 |
| ENSG00000130766.4_2 | SESN2 | chr1:285860+ | protein_codi-0.562258 | 0.6772413 | 0.0001044 | 0.0866373 | 4.2775369 | 4.839795 | 18.133541 | 17.848707 | 19.226645 | 28.413494 | 27.173027 | 27.339375 |
| ENSG00000131051.22_3 | RBM39 | chr20:34289- | protein_codi-0.3192 | 0.8015142 | 0.0149319 | 0.2432408 | 3.4649412 | 3.7841412 | 10.136725 | 10.518409 | 9.4955128 | 11.554707 | 13.304115 | 13.5597 |
| ENSG00000131152.4_4 | AC010531.1 | chr16:87336- | protein_codi-0.603101 | 0.6583375 | 0.0355435 | 0.3030061 | 0.5782173 | 1.181318 | 0.388625 | 0.427731 | 0.678614 | 0.987624 | 1.883334 | 1.035201 |
| ENSG00000131153.8_3 | GINS2 | chr16:85709- | protein_codi0.2730126 | 1.2083284 | 0.000172 | 0.0943724 | 3.1844957 | 2.9114831 | 8.1516819 | 7.9232896 | 8.2015346 | 6.6787214 | 6.4659048 | 6.4295045 |
| ENSG00000131791.7_2 | PRKAB2 | chr1:146626- | protein_codi-0.343734 | 0.787999 | 0.0180657 | 0.2536037 | 2.3598713 | 2.7036055 | 4.4458819 | 4.1668715 | 3.8070675 | 5.0233194 | 6.1473722 | 5.4212081 |
| ENSG00000132003.9_3 | ZSWIM4 | chr19:13906+ | protein_codi-0.321878 | 0.800028 | 0.0292612 | 0.2885442 | 2.0107422 | 2.3326199 | 2.8284532 | 2.8212022 | 3.473607 | 4.1123612 | 3.6533036 | 4.372578 |
| ENSG00000132256.18_3 | TRIM5 | chr11:56847- | protein_codi0.399674 | 1.3192098 | 0.0094759 | 0.2138428 | 1.2893958 | 0.8897217 | 1.2288239 | 1.4966357 | 1.6242757 | 0.9810055 | 0.8152032 | 0.7688372 |
| ENSG00000132356.11_2 | PRKAA1 | chr5:407594- | protein_codi-0.301506 | 0.8114049 | 0.0406973 | 0.3138435 | 2.0163756 | 2.3178817 | 3.2324237 | 3.1912675 | 2.7327949 | 3.4608961 | 4.3990964 | 4.1465072 |
| ENSG00000132436.11_4 | FIGNL1 | chr7:505118- | protein_codi-0.560678 | 0.6779836 | 0.0186766 | 0.2558547 | 1.5005989 | 2.0612767 | 2.1814539 | 1.9434251 | 1.4193418 | 3.1233661 | 3.6614965 | 2.7821703 |
| ENSG00000132522.15_3 | GPS2 | chr17:72146- | protein_codi0.2731407 | 1.2084357 | 0.0262838 | 0.2808714 | 3.8455218 | 3.5723811 | 13.986569 | 12.331472 | 13.868662 | 11.832944 | 10.201321 | 10.710786 |
| ENSG00000132549.18_3 | VPS13B | chr8:100025+ | protein_codi-0.400403 | 0.7576464 | 0.0299168 | 0.2902373 | 1.717944 | 2.1183473 | 2.2893668 | 2.4498648 | 2.1372103 | 2.8721815 | 4.058591 | 3.1790061 |
| ENSG00000132661.3_3 | NXT1 | chr20:23331+ | protein_codi0.2781505 | 1.2126394 | 0.0077531 | 0.2077093 | 5.1987975 | 4.920647 | 34.497627 | 34.437046 | 38.384483 | 30.15164 | 28.355066 | 29.382467 |
| ENSG00000132773.11_2 | TOE1 | chr1:458053+ | protein_codi0.2801652 | 1.2143339 | 0.015946 | 0.2484394 | 3.6036093 | 3.3234441 | 11.194702 | 10.538461 | 11.766281 | 9.5975846 | 8.3014274 | 9.1768312 |
| ENSG00000132846.5_4 | ZBED3 | chr5:763678- | protein_codi-0.502396 | 0.7059334 | 0.0003104 | 0.0943724 | 2.4887305 | 2.9911264 | 4.5754131 | 4.692355 | 4.5715826 | 7.3280227 | 6.5364852 | 7.008397 |
| ENSG00000133048.12_2 | CHI3L1 | chr1:203148- | protein_codi0.3888679 | 1.3093655 | 0.00376 | 0.1787164 | 1.7036542 | 1.3147863 | 2.1333826 | 2.0984671 | 2.5595306 | 1.4784565 | 1.4946984 | 1.4898376 |
| ENSG00000133121.20_3 | STARD13 | chr13:33677- | protein_codi-0.472832 | 0.7205486 | 0.0005598 | 0.1106916 | 0.5823636 | 1.055196 | 0.5246945 | 0.4820626 | 0.4855161 | 1.1576924 | 1.1302609 | 0.9521565 |
| ENSG00000133401.15_3 | PDZD2 | chr5:316395+ | protein_codi-0.380212 | 0.7683245 | 0.0085581 | 0.2112786 | 2.0789077 | 2.4591201 | 3.4418994 | 3.1991498 | 3.0430642 | 4.858551 | 4.0113944 | 4.6631479 |
| ENSG00000133639.4_3 | BTG1 | chr12:92534- | protein_codi-0.356627 | 0.7809886 | 0.0022811 | 0.1570628 | 1.9367668 | 2.2933933 | 3.0255194 | 2.8760378 | 2.5963735 | 4.0078594 | 3.7839268 | 3.9170417 |
| ENSG00000133706.17_2 | LARS | chr5:145492- | protein_codi-0.384157 | 0.7662265 | 0.0366246 | 0.3055634 | 3.2176843 | 3.6018414 | 8.6155272 | 9.4625602 | 7.0029005 | 10.337784 | 11.529762 | 11.598415 |
| ENSG00000133794.17_3 | ARNTL | chr11:13298+ | protein_codi-0.342242 | 0.7888145 | 0.0016178 | 0.1419323 | 0.7368131 | 1.0790551 | 0.6857313 | 0.6987102 | 0.6162215 | 1.0229525 | 1.0986993 | 1.2209971 |
| ENSG00000134184.12_3 | GSTM1 | chr1:110230+ | protein_codi0.283912 | 1.2174917 | 0.0139679 | 0.2388191 | 4.4371524 | 4.1532405 | 19.690202 | 21.863964 | 20.489765 | 16.008084 | 16.2954 | 18.149783 |
| ENSG00000134294.13_2 | SLC38A2 | chr12:46751- | protein_codi-0.583332 | 0.6674205 | 0.0417486 | 0.3140442 | 4.864713 | 5.4480451 | 32.425153 | 30.903801 | 22.193047 | 37.446416 | 48.738908 | 42.503371 |
| ENSG00000134321.11_2 | RSAD2 | chr2:700593+ | protein_codi0.3707142 | 1.2929928 | 0.0141053 | 0.2399539 | 2.0977239 | 1.7270097 | 3.4904806 | 3.2700502 | 3.0898525 | 2.0675337 | 2.6879216 | 2.2068168 |
| ENSG00000134326.11_4 | CMPK2 | chr2:698070- | protein_codi0.4794942 | 1.3942547 | 0.0007148 | 0.114143 | 1.8113382 | 1.3318441 | 2.6071337 | 2.5878226 | 2.340475 | 1.5742944 | 1.5887594 | 1.3934544 |
| ENSG00000134444.13_3 | KIAA1468 | chr18:59854+ | protein_codi-0.429036 | 0.742758 | 0.0343449 | 0.2993278 | 2.1554514 | 2.5844872 | 3.7006127 | 3.8959388 | 2.8421661 | 4.4201297 | 5.6062625 | 5.0264143 |
| ENSG00000134684.10_2 | YARS | chr1:332408- | protein_codi-0.313035 | 0.8049468 | 0.0175365 | 0.2506617 | 4.0177714 | 4.330806 | 15.860019 | 14.43088 | 15.336551 | 19.944686 | 17.246679 | 20.323075 |
| ENSG00000134698.10_2 | AGO4 | chr1:362737+ | protein_codi-0.304131 | 0.8099299 | 0.0230321 | 0.2666812 | 2.1003361 | 2.4044671 | 3.5782994 | 3.4594053 | 2.8619921 | 4.2427564 | 4.5577001 | 4.0932545 |
| ENSG00000134853.11_3 | PDGFRA | chr4:550952+ | protein_codi-0.350856 | 0.7841185 | 0.0229733 | 0.2666812 | 1.8085188 | 2.1593752 | 2.8720963 | 2.5929222 | 2.0893085 | 3.3540393 | 3.4957125 | 3.5542752 |
| ENSG00000134884.13_3 | ARGLU1 | chr13:10719- | protein_codi-0.298565 | 0.8130605 | 0.0201582 | 0.2576869 | 4.0153326 | 4.3138979 | 14.514778 | 15.76147 | 15.261065 | 17.04121 | 20.377589 | 19.399222 |
| ENSG00000134897.13_3 | BIVM | chr13:10345+ | protein_codi-0.411177 | 0.7520099 | 0.0066913 | 0.2040161 | 1.1888087 | 1.5999852 | 1.3065502 | 1.321252 | 1.2126669 | 1.8380176 | 2.367506 | 1.9147891 |
| ENSG00000135069.13_2 | PSAT1 | chr9:809120+ | protein_codi-0.492982 | 0.7105547 | 0.0049551 | 0.1885042 | 6.1231105 | 6.616093 | 70.832617 | 73.977833 | 61.873149 | 99.239497 | 91.454005 | 100.85039 |
| ENSG00000135100.17_2 | HNF1A | chr12:12141+ | protein_codi-0.384941 | 0.7658103 | 0.0141196 | 0.2399539 | 0.5450788 | 0.9300199 | 0.3766428 | 0.5132362 | 0.4911704 | 0.7933876 | 1.1350783 | 0.8063576 |
| ENSG00000135114.12_2 | OASL | chr12:12145- | protein_codi0.3478284 | 1.2726436 | 0.0149515 | 0.2432408 | 4.5825247 | 4.2346963 | 20.675347 | 23.014714 | 25.4234 | 17.304364 | 18.036141 | 18.150419 |
| ENSG00000135164.18_3 | DMTF1 | chr7:867816+ | protein_codi-0.473444 | 0.7202431 | 0.041082 | 0.3138435 | 1.7279097 | 2.2013538 | 2.3232136 | 2.541475 | 2.0882717 | 2.790794 | 4.3910723 | 3.7600908 |
| ENSG00000135241.16_3 | PNPLA8 | chr7:108110- | protein_codi-0.31244 | 0.8052784 | 0.0250216 | 0.2761127 | 1.8143009 | 2.1267413 | 2.4060531 | 2.7854794 | 2.3736862 | 3.233956 | 3.8143582 | 3.086524 |
| ENSG00000135363.11_2 | LMO2 | chr11:33880- | protein_codi-0.471951 | 0.7209892 | 0.0062776 | 0.2035654 | 3.0002111 | 3.4721616 | 6.3205205 | 7.0766033 | 7.6634307 | 10.764059 | 9.3166917 | 10.260995 |
| ENSG00000135451.12_4 | TROAP | chr12:49717+ | protein_codi0.266512 | 1.202896 | 0.0193778 | 0.2575012 | 3.8964906 | 3.6299787 | 13.60919 | 13.0081 | 15.138988 | 11.701523 | 10.77851 | 11.683855 |
| ENSG00000135480.15_3 | KRT7 | chr12:52626+ | protein_codi0.2707192 | 1.2064091 | 0.0212298 | 0.262461 | 1.3697039 | 1.0989847 | 1.3620724 | 1.642623 | 1.7646331 | 1.230659 | 1.0933883 | 1.1047433 |
| ENSG00000136044.11_2 | APPL2 | chr12:10556- | protein_codi-0.3021 | 0.8110709 | 0.0325754 | 0.2958398 | 2.3763568 | 2.6784568 | 4.3695753 | 4.4207531 | 3.8091075 | 5.4916573 | 5.9592574 | 4.8072301 |
| ENSG00000136169.16_3 | SETDB2 | chr13:50018+ | protein_codi-0.368201 | 0.7747481 | 0.0081058 | 0.2097267 | 0.7083379 | 1.0765386 | 0.6817691 | 0.6595274 | 0.562939 | 1.023224 | 1.3167205 | 1.0012145 |
| ENSG00000136271.10_3 | DDX56 | chr7:446050- | protein_codi0.302851 | 1.2335797 | 0.0363961 | 0.3050104 | 4.2565239 | 3.9536729 | 18.28069 | 17.439694 | 18.640288 | 16.283633 | 12.794308 | 14.602259 |
| ENSG00000136371.10_3 | MTHFS | chr15:80125- | protein_codi0.2633702 | 1.2002793 | 0.0016952 | 0.1441094 | 3.4023007 | 3.1389305 | 9.2422013 | 9.4687998 | 10.022839 | 7.9732323 | 7.8155958 | 7.6404459 |
| ENSG00000136628.17_2 | EPRS | chr1:220141- | protein_codi-0.387558 | 0.7644223 | 0.0394766 | 0.3123847 | 5.3034308 | 5.6909891 | 41.322361 | 42.420994 | 32.512404 | 48.82969 | 54.307556 | 49.026784 |
| ENSG00000137145.20_2 | DENND4C | chr9:192304+ | protein_codi-0.518813 | 0.697946 | 0.0305064 | 0.2908365 | 3.4600671 | 3.9788798 | 10.918724 | 10.944939 | 8.3613443 | 13.545583 | 17.280118 | 13.742678 |
| ENSG00000137193.13_2 | PIM1 | chr6:371379+ | protein_codi-0.300547 | 0.8119445 | 0.0326071 | 0.2958398 | 3.1609012 | 3.4614481 | 7.1418098 | 8.2109306 | 8.5401178 | 9.9931773 | 9.2241551 | 10.891825 |
| ENSG00000137267.5_2 | TUBB2A | chr6:315390- | protein_codi0.3997153 | 1.3192476 | 0.0029522 | 0.1627656 | 5.7240523 | 5.324337 | 50.849721 | 52.110437 | 52.630028 | 42.367673 | 36.65084 | 38.392597 |
| ENSG00000137285.9_2 | TUBB2B | chr6:322449- | protein_codi0.3945206 | 1.3145059 | 0.0036396 | 0.1767394 | 4.2251695 | 3.8306489 | 17.43083 | 17.362226 | 18.330275 | 14.371521 | 12.327827 | 13.058677 |
| ENSG00000137648.17_3 | TMPRSS4 | chr11:11794+ | protein_codi-0.279347 | 0.8239638 | 0.0045968 | 0.1849138 | 2.3653249 | 2.6446721 | 4.1657401 | 4.2494527 | 4.0449242 | 4.9798574 | 5.1500706 | 5.6497601 |
| ENSG00000137720.7_2 | C11orf1 | chr11:11174+ | protein_codi0.3181234 | 1.2467078 | 0.0207658 | 0.2615337 | 1.7561508 | 1.4380274 | 2.5447512 | 2.0195055 | 2.6011455 | 1.6621351 | 1.6527268 | 1.8167322 |
| ENSG00000137876.9_2 | RSL24D1 | chr15:55473- | protein_codi-0.434126 | 0.7401422 | 0.0350555 | 0.3014271 | 3.8786442 | 4.3127698 | 14.535398 | 14.896637 | 11.886562 | 16.328 | 20.663593 | 19.909337 |
| ENSG00000138386.16_3 | NAB1 | chr2:191511+ | protein_codi-0.371623 | 0.7729126 | 0.0432962 | 0.3166579 | 2.7644782 | 3.136101 | 6.4520779 | 5.6661867 | 5.3156446 | 7.7872464 | 8.9889373 | 6.7412181 |
| ENSG00000138439.11_2 | FAM117B | chr2:203499+ | protein_codi-0.292831 | 0.8162989 | 0.013225 | 0.2354662 | 2.0233234 | 2.316154 | 3.2688332 | 3.1660409 | 2.7775511 | 4.186022 | 4.0691054 | 3.6981819 |
| ENSG00000138688.15_3 | KIAA1109 | chr4:123073+ | protein_codi-0.466036 | 0.7239511 | 0.0360143 | 0.3039541 | 2.0866074 | 2.5526432 | 3.6169053 | 3.5166936 | 2.6747064 | 4.2701047 | 5.7680927 | 4.6621445 |
| ENSG00000138760.9_2 | SCARB2 | chr4:770798- | protein_codi-0.459688 | 0.7271437 | 0.0120575 | 0.2323938 | 3.4015267 | 3.8612142 | 9.443822 | 9.77493 | 9.4860218 | 13.415771 | 15.537819 | 11.873831 |
| ENSG00000138764.14_3 | CCNG2 | chr4:780783+ | protein_codi-0.46116 | 0.7264019 | 0.004056 | 0.182744 | 1.8887528 | 2.349913 | 2.7139895 | 2.8632221 | 2.5393492 | 3.659706 | 4.4791359 | 4.1893421 |
| ENSG00000138767.12_3 | CNOT6L | chr4:786345- | protein_codi-0.47055 | 0.7216896 | 0.0492873 | 0.3289498 | 1.8274558 | 2.2980054 | 2.8447878 | 3.0725953 | 1.8550478 | 3.5779016 | 4.2428295 | 3.9553398 |
| ENSG00000138780.14_3 | GSTCD | chr4:106629+ | protein_codi-0.413762 | 0.7506636 | 0.0382578 | 0.3085154 | 2.1131827 | 2.5269443 | 3.1895719 | 3.3814882 | 3.411668 | 5.1971266 | 3.7936294 | 5.4447082 |
| ENSG00000138814.16_2 | PPP3CA | chr4:101944- | protein_codi-0.278454 | 0.8244738 | 0.0236894 | 0.2692169 | 2.5836805 | 2.8621349 | 4.590275 | 5.6008423 | 4.8379919 | 6.3291572 | 6.5145773 | 5.9792187 |
| ENSG00000139178.10_3 | C1RL | chr12:72421- | protein_codi-0.30719 | 0.8082145 | 0.0095433 | 0.2139 | 2.8622832 | 3.169473 | 6.1080468 | 5.8993078 | 6.8404956 | 8.4427234 | 7.745971 | 7.8188926 |
| ENSG00000139269.2_2 | INHBE | chr12:57846+ | protein_codi-0.834452 | 0.5607959 | 0.0125751 | 0.2343664 | 0.1765445 | 1.0109969 | 0.1629036 | 0.1205283 | 0.1078194 | 1.3978383 | 0.5501641 | 1.2020319 |
| ENSG00000139289.13_2 | PHLDA1 | chr12:76419- | protein_codi-0.299576 | 0.8124913 | 0.0027899 | 0.162234 | 3.8674814 | 4.1670571 | 14.517976 | 13.286065 | 13.02607 | 17.028468 | 17.107989 | 16.758181 |
| ENSG00000139514.12_3 | SLC7A1 | chr13:30083- | protein_codi-0.562733 | 0.6770185 | 0.0019827 | 0.1522433 | 4.9184343 | 5.4811671 | 28.312954 | 30.8055 | 28.663812 | 46.580614 | 39.661184 | 45.065639 |
| ENSG00000139793.18_3 | MBNL2 | chr13:97873+ | protein_codi-0.36961 | 0.7739916 | 0.0498308 | 0.3301996 | 2.912056 | 3.2816661 | 7.3432185 | 6.3918842 | 5.914485 | 7.829162 | 10.246093 | 8.2622953 |
| ENSG00000139946.9_2 | PELI2 | chr14:56584+ | protein_codi-0.432598 | 0.7409264 | 0.0192423 | 0.2572494 | 2.3405131 | 2.7731109 | 4.3342614 | 4.5039607 | 3.4253168 | 5.3542598 | 6.2999819 | 5.8862369 |
| ENSG00000140044.12_2 | JDP2 | chr14:75894+ | protein_codi-0.426112 | 0.7442648 | 0.000588 | 0.1106916 | 0.9275613 | 1.3536733 | 0.912084 | 0.8251582 | 0.9718044 | 1.6386178 | 1.4653693 | 1.5658445 |
| ENSG00000140262.17_2 | TCF12 | chr15:57210+ | protein_codi-0.505424 | 0.7044535 | 0.0336319 | 0.2984107 | 1.7662904 | 2.2717141 | 2.5508038 | 2.8649598 | 1.8684518 | 4.4678856 | 3.7042221 | 3.3777908 |
| ENSG00000140455.16_4 | USP3 | chr15:63796+ | protein_codi-0.288472 | 0.8187686 | 0.0362702 | 0.3048333 | 2.2056419 | 2.4941143 | 3.4823601 | 4.1394736 | 3.2605705 | 4.856828 | 4.2867381 | 4.7750979 |
| ENSG00000140750.16_3 | ARHGAP17 | chr16:24930- | protein_codi0.3533117 | 1.2774897 | 0.0090292 | 0.2129568 | 2.9186681 | 2.5653564 | 6.5153785 | 6.6483985 | 6.5213986 | 5.2380781 | 5.2234768 | 4.3415073 |
| ENSG00000140832.9_3 | MARVELD3 | chr16:71660+ | protein_codi0.3009382 | 1.2319453 | 0.0020776 | 0.1522433 | 3.2027729 | 2.9018347 | 8.2509923 | 8.3723142 | 8.0023719 | 6.1714221 | 6.8604132 | 6.4057182 |
| ENSG00000140961.12_3 | OSGIN1 | chr16:83981+ | protein_codi0.4405011 | 1.3570756 | 0.0137651 | 0.2388191 | 3.2540034 | 2.8135023 | 8.5560938 | 7.7067059 | 9.4357483 | 6.6158625 | 5.4005521 | 6.1270408 |
| ENSG00000141198.15_4 | TOM1L1 | chr17:52976+ | protein_codi-0.346654 | 0.7864061 | 0.0294496 | 0.2891776 | 1.9094165 | 2.2560702 | 3.028844 | 2.8322021 | 2.4335716 | 3.2899868 | 3.9430848 | 4.1402043 |
| ENSG00000141552.17_3 | ANAPC11 | chr17:79848+ | protein_codi0.6030125 | 1.5188849 | 0.0294746 | 0.2891776 | 2.8391912 | 2.2361786 | 7.1105692 | 7.0238105 | 4.6313579 | 3.6991013 | 3.4099385 | 4.0468825 |
| ENSG00000142530.10_3 | FAM71E1 | chr19:50970- | protein_codi-0.320141 | 0.8009913 | 0.0321732 | 0.2958398 | 1.6057572 | 1.9258987 | 2.1534944 | 2.3183929 | 1.6941599 | 2.8933161 | 2.5907704 | 2.9242124 |
| ENSG00000142627.12_2 | EPHA2 | chr1:164508- | protein_codi0.2968729 | 1.2284788 | 0.0289939 | 0.2885442 | 6.0992427 | 5.8023697 | 64.582785 | 64.335398 | 74.201511 | 59.114021 | 51.215264 | 54.371811 |
| ENSG00000142871.16_3 | CYR61 | chr1:860464+ | protein_codi0.5420632 | 1.4560533 | 0.00336 | 0.172735 | 3.7431677 | 3.2011045 | 12.69164 | 12.169233 | 12.316843 | 9.1977113 | 7.3104351 | 8.1782304 |
| ENSG00000142910.15_2 | TINAGL1 | chr1:320420+ | protein_codi0.3554219 | 1.2793597 | 0.0113076 | 0.2280969 | 5.0995775 | 4.7441556 | 32.644893 | 32.204561 | 35.079495 | 28.131208 | 23.577104 | 25.885009 |
| ENSG00000143252.14_2 | SDHC | chr1:161284+ | protein_codi0.3442265 | 1.2694702 | 0.0113543 | 0.2280969 | 3.3802306 | 3.0360041 | 9.1023574 | 10.435723 | 8.7716089 | 7.5718233 | 7.1742205 | 6.8752686 |
| ENSG00000143412.9_2 | ANXA9 | chr1:150954+ | protein_codi-0.488278 | 0.7128756 | 0.0014815 | 0.1390335 | 3.5148544 | 4.0031321 | 10.147305 | 10.638721 | 10.512103 | 15.012731 | 13.930216 | 16.244753 |
| ENSG00000143507.17_2 | DUSP10 | chr1:221874- | protein_codi0.2729633 | 1.2082871 | 0.006448 | 0.2040161 | 2.3294363 | 2.056473 | 4.2931591 | 3.8805156 | 3.9148462 | 3.3448951 | 2.9798438 | 3.162314 |
|  |  |  |  |  |  |  |  |  |  |  |  |  |  |  |
| ENSG00000143537.13_3 | ADAM15 | chr1:155023+ | protein_codi0.2734717 | 1.208713 | 0.0301905 | 0.2902479 | 4.3395334 | 4.0660617 | 18.839758 | 18.29175 | 20.681094 | 17.016448 | 14.40174 | 15.934824 |
| ENSG00000143776.18_3 | CDC42BPA | chr1:227177- | protein_codi-0.455611 | 0.7292012 | 0.0161191 | 0.2484394 | 2.8884187 | 3.3440298 | 6.5733476 | 7.0152089 | 5.6880464 | 8.6911444 | 10.397125 | 8.4795872 |
| ENSG00000144224.16_2 | UBXN4 | chr2:136499+ | protein_codi-0.352362 | 0.7833005 | 0.0447269 | 0.3195842 | 4.1332031 | 4.4855653 | 18.021852 | 16.848947 | 14.914351 | 19.090407 | 24.280803 | 21.135511 |
| ENSG00000144426.18_3 | NBEAL1 | chr2:203879+ | protein_codi-0.380743 | 0.768042 | 0.0466192 | 0.3240376 | 1.5828048 | 1.9635476 | 2.2791355 | 2.0937066 | 1.6495779 | 2.4671441 | 3.3804113 | 2.9063705 |
| ENSG00000144535.19_3 | DIS3L2 | chr2:232825+ | protein_codi-0.307899 | 0.8078173 | 0.0032041 | 0.1692945 | 2.0021876 | 2.3100866 | 3.0394359 | 2.9672072 | 3.0118989 | 3.6530244 | 4.2078956 | 4.032902 |
| ENSG00000144655.14_2 | CSRNP1 | chr3:391833- | protein_codi0.4264785 | 1.3439491 | 0.0131175 | 0.2354662 | 2.9093032 | 2.4828247 | 5.9207513 | 6.4918123 | 7.1775352 | 5.1605791 | 4.436773 | 4.2149646 |
| ENSG00000144827.8_2 | ABHD10 | chr3:111697+ | protein_codi-0.467408 | 0.7232629 | 0.0178354 | 0.2517639 | 2.6889989 | 3.1564069 | 5.4108279 | 4.76605 | 6.2546339 | 7.0914507 | 8.1258747 | 8.5988368 |
| ENSG00000145332.13_2 | KLHL8 | chr4:880812- | protein_codi-0.282214 | 0.8223283 | 0.0424162 | 0.3149427 | 1.1894036 | 1.4716172 | 1.4814505 | 1.3341267 | 1.0478986 | 1.6257667 | 1.9597269 | 1.7447008 |
| ENSG00000145555.14_4 | MYO10 | chr5:166620- | protein_codi-0.365145 | 0.7763906 | 0.0020557 | 0.1522433 | 3.8078531 | 4.1729986 | 13.402567 | 13.139006 | 12.48886 | 17.772803 | 17.369124 | 16.020632 |
| ENSG00000145912.8_2 | NHP2 | chr5:177576- | protein_codi0.2733228 | 1.2085882 | 0.00377 | 0.1787164 | 5.8882591 | 5.6149363 | 56.80139 | 57.219 | 60.748272 | 49.841635 | 45.934242 | 48.326854 |
| ENSG00000146232.15_3 | NFKBIE | chr6:442259- | protein_codi0.4518381 | 1.3677818 | 0.0192552 | 0.2572494 | 2.1694328 | 1.7175947 | 4.1061491 | 3.4360318 | 3.0188787 | 2.4674971 | 2.0091399 | 2.4094483 |
| ENSG00000146278.10_2 | PNRC1 | chr6:897904+ | protein_codi-0.349791 | 0.7846977 | 0.0117668 | 0.2316371 | 3.887276 | 4.2370672 | 13.815773 | 14.884002 | 12.768157 | 16.599533 | 18.942434 | 18.106171 |
| ENSG00000146733.13_2 | PSPH | chr7:560787- | protein_codi-0.549447 | 0.6832819 | 0.01675 | 0.2498875 | 3.0455027 | 3.59495 | 7.1568489 | 7.9446206 | 6.7139826 | 10.189305 | 9.9884357 | 13.34909 |
| ENSG00000147162.13_2 | OGT | chrX:70752+ | protein_codi-0.461411 | 0.7262755 | 0.0134013 | 0.2371247 | 3.7637911 | 4.2252023 | 13.512238 | 12.658977 | 11.644147 | 15.755911 | 19.935406 | 17.650415 |
| ENSG00000147251.15_2 | DOCK11 | chrX:11762+ | protein_codi-0.570226 | 0.6735114 | 0.0065077 | 0.2040161 | 2.0388699 | 2.6090956 | 3.4377964 | 3.3132383 | 2.6250344 | 5.2155705 | 5.4909188 | 4.6293847 |
| ENSG00000147419.17_2 | CCDC25 | chr8:275908- | protein_codi-0.296092 | 0.8144556 | 0.0078631 | 0.2082139 | 2.264187 | 2.560279 | 3.6427726 | 4.0958096 | 3.6856946 | 4.6522515 | 5.2377773 | 4.8198386 |
| ENSG00000147421.17_3 | HMBOX1 | chr8:287479+ | protein_codi-0.413028 | 0.7510454 | 0.0498975 | 0.3302994 | 2.0416537 | 2.4546817 | 3.9552661 | 2.4779841 | 3.0495099 | 4.558772 | 4.3731194 | 4.5156043 |
| ENSG00000147548.16_3 | NSD3 | chr8:381272- | protein_codi-0.272111 | 0.8281068 | 0.0327004 | 0.2958398 | 2.5284823 | 2.8005936 | 5.1199846 | 4.8592119 | 4.3562089 | 5.6791884 | 6.6052767 | 5.6580845 |
| ENSG00000147586.9_3 | MRPS28 | chr8:808309- | protein_codi0.3237927 | 1.2516166 | 0.0284917 | 0.2885442 | 3.3311785 | 3.0073859 | 9.4889896 | 8.443763 | 9.2914185 | 7.9174361 | 7.0098161 | 6.2790971 |
| ENSG00000147894.14_2 | C9orf72 | chr9:275465- | protein_codi-0.317638 | 0.8023827 | 0.0417231 | 0.3140442 | 1.0507169 | 1.3683545 | 1.2210421 | 1.1808082 | 0.8353446 | 1.4769498 | 1.8139932 | 1.4689199 |
| ENSG00000148225.15_3 | WDR31 | chr9:116075- | protein_codi-0.327597 | 0.7968627 | 0.006525 | 0.2040161 | 0.7137252 | 1.0413221 | 0.6772731 | 0.6936434 | 0.5528599 | 1.0653744 | 0.9372148 | 1.1788685 |
| ENSG00000148498.15_4 | PARD3 | chr10:34398- | protein_codi-0.293844 | 0.8157259 | 0.0088548 | 0.2121942 | 4.7378862 | 5.0317299 | 27.428045 | 26.077042 | 23.682513 | 31.423199 | 32.396451 | 31.325756 |
| ENSG00000148634.15_3 | HERC4 | chr10:69681- | protein_codi-0.324338 | 0.7986646 | 0.0437082 | 0.317348 | 1.8959541 | 2.2202925 | 2.9585678 | 2.7779826 | 2.4468241 | 3.2342335 | 4.2846715 | 3.5220055 |
| ENSG00000148832.15_3 | PAOX | chr10:13519+ | protein_codi-0.281333 | 0.8228306 | 0.01455 | 0.2418307 | 0.8652069 | 1.1465395 | 0.7742098 | 0.8850869 | 0.8072712 | 1.1900293 | 1.0646338 | 1.3995808 |
| ENSG00000149150.8_3 | SLC43A1 | chr11:57252- | protein_codi-0.518186 | 0.6982492 | 0.002111 | 0.1522433 | 0.4971734 | 1.0153594 | 0.390511 | 0.3552487 | 0.4921033 | 1.1844513 | 0.8920736 | 0.9983914 |
| ENSG00000149177.12_2 | PTPRJ | chr11:48002+ | protein_codi-0.305254 | 0.8092996 | 0.0121413 | 0.2335695 | 3.1311803 | 3.4364346 | 7.8884244 | 8.4313308 | 7.0230564 | 9.6108932 | 10.043367 | 9.8281989 |
| ENSG00000149308.16_2 | NPAT | chr11:10802- | protein_codi-0.400498 | 0.7575966 | 0.0366328 | 0.3055634 | 2.045863 | 2.4463612 | 3.2800259 | 2.9912327 | 3.1214019 | 4.1385435 | 5.4754718 | 3.8660085 |
| ENSG00000149311.18_2 | ATM | chr11:10809+ | protein_codi-0.481951 | 0.7160086 | 0.0240903 | 0.271247 | 1.5726619 | 2.0546132 | 1.9377898 | 2.2833716 | 1.7284406 | 2.7281045 | 3.8292473 | 2.9822917 |
| ENSG00000149591.16_3 | TAGLN | chr11:11707+ | protein_codi0.5653964 | 1.4797941 | 0.001928 | 0.1517515 | 2.6172194 | 2.0518229 | 4.836895 | 5.4288653 | 5.1555717 | 2.7844081 | 3.2812485 | 3.3995913 |
| ENSG00000149634.4_2 | SPATA25 | chr20:44515- | protein_codi-0.382834 | 0.7669298 | 0.026107 | 0.2797678 | 1.0209718 | 1.4038053 | 1.259019 | 0.881982 | 0.965595 | 1.596204 | 1.437275 | 1.92765 |
| ENSG00000149806.10_3 | FAU | chr11:64888- | protein_codi0.2843622 | 1.2178718 | 0.0212984 | 0.2625473 | 6.1538115 | 5.8694493 | 66.476887 | 69.942993 | 74.401683 | 59.772533 | 52.656326 | 60.279133 |
| ENSG00000150347.14_2 | ARID5B | chr10:63661+ | protein_codi-0.37201 | 0.7727053 | 0.0101552 | 0.2182659 | 4.9092419 | 5.2812518 | 30.327602 | 30.472158 | 26.519056 | 36.10204 | 40.647703 | 37.058946 |
| ENSG00000150593.17_3 | PDCD4 | chr10:11263+ | protein_codi-0.825386 | 0.564331 | 0.0130574 | 0.2354662 | 2.0878541 | 2.9132405 | 3.0043039 | 3.7887768 | 3.0065306 | 5.0030161 | 8.0454239 | 6.8726317 |
| ENSG00000150712.10_2 | MTMR12 | chr5:322271- | protein_codi-0.27623 | 0.8257459 | 0.0468601 | 0.3240376 | 2.7942682 | 3.0704984 | 6.4376329 | 6.1458527 | 5.2803932 | 8.1210129 | 7.2852785 | 6.8448772 |
| ENSG00000150756.13_4 | FAM173B | chr5:102264- | protein_codi-0.308526 | 0.8074662 | 3.026E-05 | 0.061109 | 1.4895533 | 1.7980796 | 1.7932544 | 1.842117 | 1.7889983 | 2.4363574 | 2.5324484 | 2.4646032 |
| ENSG00000150760.12_2 | DOCK1 | chr10:12859+ | protein_codi-0.278526 | 0.8244332 | 0.0130204 | 0.2354662 | 3.789958 | 4.0684835 | 13.300525 | 13.476525 | 11.783707 | 15.228856 | 16.490903 | 15.638211 |
| ENSG00000150907.7_3 | FOXO1 | chr13:41044- | protein_codi-0.369148 | 0.7742398 | 0.0014142 | 0.1390335 | 2.1167356 | 2.4858832 | 3.522072 | 3.389067 | 3.1104845 | 4.5631797 | 4.7781379 | 4.4684623 |
| ENSG00000150995.18_3 | ITPR1 | chr3:453503+ | protein_codi-0.322237 | 0.7998286 | 0.0030637 | 0.1668001 | 0.9801304 | 1.3023676 | 1.071034 | 0.9923123 | 0.8603785 | 1.3922843 | 1.5114726 | 1.4969734 |
| ENSG00000151012.13_2 | SLC7A11 | chr4:139085- | protein_codi-1.11317 | 0.4622771 | 0.0003163 | 0.0943724 | 0.9349384 | 2.0481088 | 1.022603 | 1.035937 | 0.696909 | 2.903545 | 3.212063 | 3.3020205 |
| ENSG00000151014.5_2 | NOCT | chr4:139936+ | protein_codi0.3188793 | 1.2473612 | 0.0162621 | 0.248954 | 2.9556377 | 2.6367584 | 7.3912807 | 6.3772775 | 6.5419296 | 4.7634208 | 5.3918125 | 5.5301892 |
| ENSG00000151090.17_3 | THRB | chr3:241586- | protein_codi-0.375992 | 0.7705753 | 0.0002043 | 0.0943724 | 1.4369581 | 1.8129503 | 1.767634 | 1.7476624 | 1.6099399 | 2.5684972 | 2.4759057 | 2.4970668 |
| ENSG00000151694.12_2 | ADAM17 | chr2:962861- | protein_codi-0.314453 | 0.804156 | 0.0250186 | 0.2761127 | 3.202008 | 3.5164607 | 8.7958065 | 8.7424282 | 7.1657102 | 10.165009 | 10.845803 | 10.330703 |
| ENSG00000152137.6_3 | HSPB8 | chr12:11961+ | protein_codi0.3320633 | 1.2588124 | 0.0056361 | 0.1931637 | 4.8661977 | 4.5341344 | 29.555864 | 27.311743 | 27.678289 | 23.524179 | 20.713519 | 22.356442 |
| ENSG00000152409.8_2 | JMY | chr5:785320+ | protein_codi-0.557343 | 0.6795525 | 0.011141 | 0.2269504 | 2.1639195 | 2.7212627 | 3.939128 | 3.789583 | 2.804224 | 5.687413 | 5.8464368 | 5.263576 |
| ENSG00000152818.18_3 | UTRN | chr6:144606+ | protein_codi-0.393776 | 0.7611351 | 0.037216 | 0.3067727 | 2.9592734 | 3.353049 | 7.5231206 | 7.0402267 | 5.8647267 | 8.8731537 | 10.503194 | 8.3935684 |
| ENSG00000153339.13_3 | TRAPPC8 | chr18:29409- | protein_codi-0.314469 | 0.8041471 | 0.0334891 | 0.2981619 | 2.1840221 | 2.4984908 | 3.7571194 | 3.8260669 | 3.0872493 | 4.5240101 | 5.143736 | 4.3171026 |
| ENSG00000153485.5_3 | TMEM251 | chr14:93651+ | protein_codi-0.803836 | 0.5728242 | 0.0099451 | 0.2170995 | 2.2445245 | 3.0483602 | 3.7263423 | 4.7150823 | 2.9396707 | 6.8276783 | 8.2412346 | 6.8267351 |
| ENSG00000153714.5_2 | LURAP1L | chr9:127750+ | protein_codi-0.360711 | 0.7787805 | 0.0041255 | 0.1830255 | 2.1533241 | 2.5140355 | 3.5775363 | 3.2717071 | 3.5020689 | 4.4349685 | 4.5860992 | 5.1389572 |
| ENSG00000153879.8_2 | CEBPG | chr19:33864+ | protein_codi-0.540682 | 0.6874461 | 0.0059415 | 0.1991444 | 4.1156258 | 4.6563074 | 17.693559 | 17.365834 | 14.173223 | 23.55048 | 25.324285 | 23.811214 |
| ENSG00000154319.14_3 | FAM167A | chr8:112789- | protein_codi-0.32971 | 0.7956966 | 0.0267265 | 0.2810959 | 0.7023689 | 1.0320786 | 0.5656615 | 0.6238842 | 0.6945305 | 1.1475816 | 0.8054891 | 1.2055417 |
| ENSG00000154359.12_2 | LONRF1 | chr8:125794- | protein_codi-0.439058 | 0.7376161 | 0.025588 | 0.2777269 | 2.0484316 | 2.4874895 | 3.8149245 | 3.1260171 | 2.5628499 | 4.4940239 | 4.7177112 | 4.6145393 |
| ENSG00000154743.17_2 | TSEN2 | chr3:125259+ | protein_codi-0.325578 | 0.7979784 | 0.0235908 | 0.2689009 | 1.2116163 | 1.5371946 | 1.4010329 | 1.3964632 | 1.1588857 | 1.6117723 | 2.0704242 | 2.0485364 |
| ENSG00000155111.14_3 | CDK19 | chr6:110931- | protein_codi-0.351659 | 0.7836823 | 0.0117821 | 0.231639 | 1.3416809 | 1.6933401 | 1.7471946 | 1.5311667 | 1.3412534 | 2.0938462 | 2.4317766 | 2.1858161 |
| ENSG00000155189.11_2 | AGPAT5 | chr8:656587+ | protein_codi-0.51501 | 0.699788 | 0.0173996 | 0.2506617 | 3.0900792 | 3.6050894 | 7.7167555 | 7.3873709 | 7.445762 | 9.3286118 | 11.357332 | 13.11736 |
| ENSG00000155313.15_2 | USP25 | chr21:17102+ | protein_codi-0.390116 | 0.7630681 | 0.0482864 | 0.3263809 | 1.9155252 | 2.3056415 | 2.9625645 | 3.0854747 | 2.3164449 | 3.5145326 | 4.6852893 | 3.7079994 |
| ENSG00000155792.9_2 | DEPTOR | chr8:120885+ | protein_codi-0.582405 | 0.6678497 | 0.0012189 | 0.1372783 | 1.1526882 | 1.7350927 | 1.4074669 | 1.1628308 | 1.1105629 | 2.3252565 | 2.1712279 | 2.4985837 |
| ENSG00000155850.7_2 | SLC26A2 | chr5:149340+ | protein_codi-0.61754 | 0.6517812 | 0.0139119 | 0.2388191 | 3.8132608 | 4.4308011 | 14.595901 | 14.041975 | 10.84138 | 19.027391 | 23.010409 | 19.863548 |
| ENSG00000155903.11_3 | RASA2 | chr3:141205+ | protein_codi-0.445391 | 0.7343855 | 0.0455474 | 0.3217476 | 2.1907108 | 2.6361013 | 4.0159399 | 3.6504399 | 3.0790834 | 4.2807761 | 6.1767316 | 5.3388754 |
| ENSG00000155918.7_2 | RAET1L | chr6:150339- | protein_codi0.2770802 | 1.21174 | 0.0268969 | 0.2817275 | 1.8422795 | 1.5651994 | 2.5018606 | 2.6862687 | 2.5715633 | 2.0723795 | 2.1715961 | 1.6592744 |
| ENSG00000156299.13_3 | TIAM1 | chr21:32490- | protein_codi-0.440808 | 0.736722 | 0.0479814 | 0.3253803 | 2.117531 | 2.5583387 | 4.3564894 | 2.7998136 | 3.0149346 | 4.943496 | 5.0732272 | 4.6617223 |
| ENSG00000157077.14_3 | ZFYVE9 | chr1:526077+ | protein_codi-0.377935 | 0.7695384 | 0.0120388 | 0.2323938 | 2.4910135 | 2.8689483 | 4.9795249 | 4.8478625 | 4.0809564 | 5.9035265 | 6.7046593 | 6.3298318 |
| ENSG00000157106.12 | SMG1 | chr16:18816- | protein_codi-0.353427 | 0.7827225 | 0.0488586 | 0.327958 | 2.699475 | 3.0529022 | 5.8695828 | 5.9511928 | 4.7395675 | 6.4796956 | 8.2630928 | 7.2490791 |
| ENSG00000157426.13_2 | AASDH | chr4:572044- | protein_codi-0.36702 | 0.7753824 | 0.0339863 | 0.2993278 | 1.5341818 | 1.9012019 | 1.9414261 | 2.0916671 | 1.67149 | 2.2944095 | 3.1604436 | 2.8022367 |
| ENSG00000157613.10_3 | CREB3L1 | chr11:46299+ | protein_codi-0.297796 | 0.8134942 | 0.0291251 | 0.2885442 | 3.0330705 | 3.3308665 | 6.5575872 | 7.4217373 | 7.6168942 | 9.52361 | 8.1599294 | 9.568548 |
| ENSG00000157796.17_3 | WDR19 | chr4:391840+ | protein_codi-0.297946 | 0.8134098 | 0.0231263 | 0.2666812 | 1.2115894 | 1.509535 | 1.3398628 | 1.3747624 | 1.2354432 | 1.6020756 | 2.1430856 | 1.8220832 |
| ENSG00000158246.7_2 | FAM46B | chr1:273315- | protein_codi0.2729807 | 1.2083017 | 0.0194549 | 0.2575012 | 3.6619066 | 3.388926 | 11.0645 | 11.843739 | 12.086708 | 10.088624 | 8.61482 | 9.781676 |
| ENSG00000158483.15_3 | FAM86C1 | chr11:71498+ | protein_codi-0.351886 | 0.7835592 | 0.0227518 | 0.2664462 | 1.5696848 | 1.9215706 | 2.1240097 | 1.6427394 | 2.1681077 | 2.8042607 | 2.5609076 | 3.0134718 |
| ENSG00000158710.14_2 | TAGLN2 | chr1:159887- | protein_codi0.305175 | 1.2355685 | 0.0201056 | 0.2575998 | 8.0767277 | 7.7715526 | 264.72992 | 261.63138 | 280.98423 | 237.57171 | 198.21967 | 218.51299 |
| ENSG00000158966.13_2 | CACHD1 | chr1:649364+ | protein_codi-0.429834 | 0.7423474 | 0.0020876 | 0.1522433 | 3.7186128 | 4.1484464 | 12.428368 | 12.850313 | 11.267588 | 16.672309 | 17.419646 | 16.133489 |
| ENSG00000160072.19_2 | ATAD3B | chr1:140714+ | protein_codi0.3578223 | 1.2814901 | 0.0028121 | 0.162509 | 4.4167882 | 4.0589659 | 20.42679 | 19.376012 | 21.319318 | 16.346948 | 14.823089 | 15.869302 |
| ENSG00000160223.16_2 | ICOSLG | chr21:45642- | protein_codi0.2981022 | 1.229526 | 0.0198743 | 0.2575012 | 1.7575741 | 1.4594719 | 2.3284247 | 2.2836847 | 2.5371 | 1.8818441 | 1.4882134 | 1.9005231 |
| ENSG00000160256.12_2 | FAM207A | chr21:46359+ | protein_codi0.4562387 | 1.3719602 | 0.0408179 | 0.3138435 | 5.1571434 | 4.7009047 | 33.540429 | 33.030137 | 37.652104 | 30.632551 | 22.222751 | 22.94926 |
| ENSG00000160570.13_2 | DEDD2 | chr19:42702- | protein_codi-0.516979 | 0.6988339 | 0.0335541 | 0.2981619 | 3.3132382 | 3.8302167 | 10.554412 | 8.2513567 | 8.1875333 | 15.630931 | 12.864993 | 11.479414 |
| ENSG00000160783.19_4 | PMF1 | chr1:156182+ | protein_codi0.8107999 | 1.7541838 | 0.0085287 | 0.2112786 | 4.157851 | 3.3470511 | 17.260696 | 13.877448 | 19.934754 | 10.127602 | 8.0506834 | 9.4617754 |
| ENSG00000160789.19_2 | LMNA | chr1:156052+ | protein_codi0.2789684 | 1.213327 | 0.005605 | 0.1931245 | 5.8801246 | 5.6011562 | 60.745287 | 55.092064 | 57.989726 | 45.980642 | 47.048478 | 49.669833 |
| ENSG00000161091.12_3 | MFSD12 | chr19:35382- | protein_codi0.3024779 | 1.2332607 | 0.0386538 | 0.3095761 | 3.401848 | 3.0993701 | 10.707165 | 8.5575808 | 9.55298 | 7.3917224 | 7.1495054 | 8.2050839 |
| ENSG00000161921.14_3 | CXCL16 | chr17:46368- | protein_codi0.3262175 | 1.2537221 | 0.0024808 | 0.1575693 | 5.4510371 | 5.1248195 | 40.370383 | 42.805057 | 45.191622 | 33.593712 | 33.492288 | 34.600332 |
| ENSG00000161980.5_2 | POLR3K | chr16:96407- | protein_codi0.3880827 | 1.3086531 | 0.001316 | 0.1390335 | 3.8434435 | 3.4553608 | 14.051099 | 13.031869 | 13.005274 | 10.49658 | 9.783817 | 9.6453645 |
| ENSG00000161981.10_2 | SNRNP25 | chr16:10301+ | protein_codi0.5009095 | 1.4151053 | 0.0105512 | 0.2222535 | 3.8720146 | 3.3711052 | 12.890383 | 13.215169 | 14.896861 | 8.1359931 | 9.7085899 | 10.322003 |
| ENSG00000162069.14_3 | BICDL2 | chr16:30776- | protein_codi-0.278224 | 0.8246055 | 0.0394493 | 0.3123648 | 1.6555559 | 1.93378 | 2.0155415 | 1.9025998 | 2.5724282 | 2.8091221 | 2.8588569 | 2.793969 |
| ENSG00000163072.15_3 | NOSTRIN | chr2:169643+ | protein_codi-0.456365 | 0.7288203 | 0.0347567 | 0.3007844 | 2.2160062 | 2.6723712 | 4.0727736 | 3.8488446 | 3.0772679 | 4.5601997 | 6.2150175 | 5.4574921 |
| ENSG00000163121.9_2 | NEURL3 | chr2:971633- | protein_codi-0.34219 | 0.7888431 | 0.0269289 | 0.281827 | 2.5297575 | 2.8719472 | 4.5461554 | 4.2791002 | 5.5772856 | 6.4411585 | 6.0381263 | 6.4908165 |
| ENSG00000163138.18_3 | PACRGL | chr4:206979+ | protein_codi-0.2818 | 0.8225638 | 0.0247314 | 0.2747088 | 1.1784497 | 1.4602502 | 1.4444526 | 1.2499163 | 1.1081338 | 1.5625023 | 1.849393 | 1.8531246 |
| ENSG00000163220.10_2 | S100A9 | chr1:153330+ | protein_codi-0.8377 | 0.5595349 | 0.0403382 | 0.3130173 | 1.4266998 | 2.2643998 | 1.811942 | 2.33069 | 1.074425 | 3.068205 | 5.292327 | 3.332541 |
| ENSG00000163286.8_3 | ALPPL2 | chr2:233271+ | protein_codi-0.696212 | 0.6171906 | 0.0023509 | 0.1571562 | 2.767311 | 3.4635231 | 5.339655 | 5.909803 | 6.204429 | 10.705506 | 8.79756 | 10.704825 |
| ENSG00000163295.4_2 | ALPI | chr2:233320+ | protein_codi-0.438985 | 0.7376536 | 0.0124214 | 0.2343664 | 2.2655366 | 2.7045213 | 3.6293216 | 3.9097801 | 3.8910716 | 6.1702351 | 4.7172895 | 5.7562002 |
| ENSG00000163463.11_3 | KRTCAP2 | chr1:155141- | protein_codi0.4697066 | 1.3848278 | 0.0022165 | 0.1540141 | 3.8252316 | 3.355525 | 13.313204 | 12.958748 | 13.254273 | 9.2427868 | 8.448477 | 10.080544 |
| ENSG00000163513.17_2 | TGFBR2 | chr3:306479+ | protein_codi-0.43846 | 0.7379218 | 0.0013937 | 0.1390335 | 3.9753804 | 4.4138406 | 15.307909 | 14.611413 | 14.285714 | 19.721463 | 21.784998 | 19.512843 |
| ENSG00000163554.12_3 | SPTA1 | chr1:158580- | protein_codi-0.479104 | 0.7174232 | 0.0040686 | 0.182744 | 1.3071659 | 1.7862696 | 1.5607168 | 1.4939769 | 1.3726544 | 2.2731934 | 2.8226899 | 2.2796043 |
| ENSG00000163611.11_2 | SPICE1 | chr3:113161- | protein_codi-0.371191 | 0.773144 | 0.0078474 | 0.2082139 | 1.4475213 | 1.8187123 | 1.929919 | 1.6664476 | 1.5968885 | 2.4393562 | 2.7951959 | 2.3631751 |
| ENSG00000163629.12_3 | PTPN13 | chr4:875154+ | protein_codi-0.322178 | 0.7998615 | 0.00069 | 0.114143 | 0.6130761 | 0.935254 | 0.5827872 | 0.5303642 | 0.4772214 | 0.8652621 | 0.9418385 | 0.930481 |
| ENSG00000163683.11_4 | SMIM14 | chr4:395479- | protein_codi-0.418488 | 0.7482084 | 0.0156637 | 0.247255 | 1.3657329 | 1.7842209 | 1.7621191 | 1.6539788 | 1.3347472 | 2.1107134 | 2.6163388 | 2.6323103 |
| ENSG00000163866.8_3 | SMIM12 | chr1:351783- | protein_codi0.3173441 | 1.2460345 | 0.0313254 | 0.2933365 | 1.9795401 | 1.662196 | 2.6183378 | 3.3116566 | 2.9314211 | 1.9510887 | 2.1220665 | 2.4410279 |
| ENSG00000163884.3_2 | KLF15 | chr3:126061- | protein_codi-0.32544 | 0.798055 | 0.0132183 | 0.2354662 | 1.157927 | 1.4833669 | 1.2011376 | 1.2292549 | 1.264151 | 2.09068 | 1.5877935 | 1.7329404 |
| ENSG00000163993.6_2 | S100P | chr4:669479+ | protein_codi-0.521275 | 0.6967556 | 0.0291737 | 0.2885442 | 3.885379 | 4.4066543 | 12.920165 | 12.970888 | 15.595046 | 22.190634 | 16.652462 | 22.307058 |
| ENSG00000164002.11_3 | EXO5 | chr1:409744+ | protein_codi0.2915842 | 1.2239836 | 0.0494323 | 0.3291951 | 1.9766089 | 1.6850247 | 3.0447008 | 2.5758987 | 3.2148691 | 2.5304482 | 2.2054826 | 1.9376881 |
| ENSG00000164045.11_3 | CDC25A | chr3:481986- | protein_codi0.2985953 | 1.2299463 | 0.0014891 | 0.1390335 | 3.9097367 | 3.6111414 | 13.300034 | 14.640831 | 14.179139 | 11.199866 | 11.141242 | 11.318773 |
| ENSG00000164142.15_3 | FAM160A1 | chr4:152330+ | protein_codi-0.284649 | 0.8209413 | 0.0036766 | 0.1776087 | 2.7756562 | 3.0603051 | 5.6176345 | 6.0541485 | 5.8789144 | 7.4176422 | 7.6884455 | 6.9359403 |
| ENSG00000164294.13_2 | GPX8 | chr5:544559+ | protein_codi-0.455414 | 0.7293007 | 0.0076309 | 0.2074702 | 0.6154201 | 1.0708345 | 0.6176902 | 0.366458 | 0.6266276 | 1.1950406 | 1.1146698 | 0.996985 |
| ENSG00000164300.16_2 | SERINC5 | chr5:794074- | protein_codi-0.327852 | 0.796722 | 0.0079165 | 0.2084187 | 4.8226117 | 5.1504634 | 28.636556 | 28.122712 | 25.25384 | 32.889811 | 36.141981 | 34.595716 |
| ENSG00000164414.17_3 | SLC35A1 | chr6:881803+ | protein_codi-0.322796 | 0.7995189 | 0.015409 | 0.2452764 | 2.2970142 | 2.6198101 | 4.1196423 | 3.8317594 | 3.7980591 | 4.9557497 | 5.7941381 | 4.7392276 |
| ENSG00000164463.12_2 | CREBRF | chr5:172483+ | protein_codi-0.36336 | 0.7773521 | 0.0369804 | 0.3060351 | 0.7452712 | 1.1086311 | 0.7920032 | 0.7159076 | 0.5318433 | 0.9523344 | 1.450484 | 1.0959819 |
| ENSG00000164609.9_2 | SLU7 | chr5:159828- | protein_codi-0.308716 | 0.8073602 | 0.0350386 | 0.3014271 | 2.3341928 | 2.6429085 | 4.4166769 | 4.1005988 | 3.6412185 | 4.7800492 | 5.8832108 | 5.124372 |
| ENSG00000164654.15_2 | MIOS | chr7:760650+ | protein_codi-0.334759 | 0.7929164 | 0.0196606 | 0.2575012 | 3.4963324 | 3.8310917 | 10.901072 | 10.53112 | 9.4723767 | 12.66531 | 14.664267 | 12.467625 |
| ENSG00000164930.11_2 | FZD6 | chr8:104310+ | protein_codi-0.280204 | 0.8234744 | 0.0350929 | 0.3014271 | 2.7448165 | 3.0250207 | 5.5574034 | 6.104167 | 5.4650398 | 6.84075 | 8.0645123 | 6.588638 |
| ENSG00000164961.15_3 | WASHC5 | chr8:126036- | protein_codi-0.328488 | 0.7963708 | 0.032949 | 0.296327 | 3.1860725 | 3.5145603 | 8.8694352 | 8.3179716 | 7.1977837 | 10.139034 | 11.482509 | 9.7353139 |
| ENSG00000165240.18_3 | ATP7A | chrX:77166+ | protein_codi-0.404082 | 0.7557172 | 0.0362868 | 0.3048333 | 1.9035086 | 2.3075902 | 2.8732912 | 3.0903359 | 2.3052206 | 3.5769505 | 4.6190377 | 3.7176256 |
| ENSG00000165359.15_3 | INTS6L | chrX:13465+ | protein_codi-0.287089 | 0.8195539 | 0.0066722 | 0.2040161 | 0.7239614 | 1.0110506 | 0.6226651 | 0.6928672 | 0.6404028 | 0.9711956 | 1.1638477 | 0.9191692 |
| ENSG00000165685.8_3 | TMEM52B | chr12:10323+ | protein_codi-0.610665 | 0.6548948 | 0.002676 | 0.1614437 | 0.7455321 | 1.3561972 | 0.655262 | 0.8177541 | 0.5663208 | 1.4001631 | 1.8032774 | 1.4937979 |
| ENSG00000165714.10_3 | BORCS5 | chr12:12510+ | protein_codi-0.357334 | 0.7806055 | 0.0341722 | 0.2993278 | 2.2233964 | 2.5807309 | 3.2404409 | 3.809703 | 3.993416 | 5.2518778 | 5.4558015 | 4.3048313 |
| ENSG00000165828.14_3 | PRAP1 | chr10:13516+ | protein_codi-0.648326 | 0.6380203 | 0.0200269 | 0.2575012 | 3.5848918 | 4.2332175 | 10.49842 | 9.4334024 | 13.401767 | 20.107995 | 15.444565 | 18.164918 |
| ENSG00000165879.8_2 | FRAT1 | chr10:99079+ | protein_codi-0.469595 | 0.7221673 | 0.0092217 | 0.2129568 | 1.2491595 | 1.7187545 | 1.171144 | 1.436917 | 1.538482 | 2.50669 | 1.976956 | 2.416018 |
| ENSG00000165916.8_3 | PSMC3 | chr11:47440- | protein_codi0.2821551 | 1.21601 | 0.011716 | 0.2313548 | 6.1440576 | 5.8619026 | 68.562245 | 67.916298 | 72.780468 | 61.77819 | 54.222647 | 55.741331 |
| ENSG00000165949.12_3 | IFI27 | chr14:94571+ | protein_codi0.5318549 | 1.4457869 | 0.0045617 | 0.1849138 | 1.8268576 | 1.2950027 | 2.8984919 | 2.325434 | 2.444069 | 1.3182445 | 1.6659295 | 1.3905403 |
| ENSG00000166046.10_3 | TCP11L2 | chr12:10669+ | protein_codi-0.611451 | 0.654538 | 0.0006654 | 0.114143 | 0.4280615 | 1.0395127 | 0.3209564 | 0.3221033 | 0.3945181 | 1.0654235 | 0.9119735 | 1.1992903 |
| ENSG00000166068.12_3 | SPRED1 | chr15:38544+ | protein_codi-0.341338 | 0.7893088 | 0.031288 | 0.2933365 | 2.6863106 | 3.027649 | 5.8971973 | 5.921372 | 4.5861857 | 6.8940124 | 7.2583008 | 7.3186136 |
| ENSG00000166123.13_3 | GPT2 | chr16:46918+ | protein_codi-0.284014 | 0.8213026 | 0.0112569 | 0.2280969 | 3.5568346 | 3.8408489 | 10.994182 | 10.50554 | 10.810386 | 14.600343 | 12.686802 | 12.778311 |
| ENSG00000166166.12_2 | TRMT61A | chr14:10399+ | protein_codi0.3686577 | 1.2911509 | 0.0259193 | 0.2789646 | 3.5579648 | 3.1893072 | 10.007842 | 10.555555 | 11.843087 | 9.023114 | 7.200087 | 8.234433 |
| ENSG00000166311.9_3 | SMPD1 | chr11:64116+ | protein_codi0.2834161 | 1.2170733 | 0.0117221 | 0.2313548 | 2.7628177 | 2.4794016 | 6.076411 | 6.0316341 | 5.2835296 | 4.5088691 | 4.4042333 | 4.8254065 |
| ENSG00000166348.18_3 | USP54 | chr10:75257- | protein_codi-0.487058 | 0.7134787 | 0.0030838 | 0.1668001 | 2.6718498 | 3.1589074 | 5.3040145 | 5.9309874 | 4.9225462 | 8.3340234 | 7.9635175 | 7.5158952 |
| ENSG00000166450.12_3 | PRTG | chr15:55903- | protein_codi-0.291941 | 0.8168023 | 0.0231806 | 0.266822 | 0.6892883 | 0.9812294 | 0.7453664 | 0.6243707 | 0.4788278 | 0.8804124 | 1.0846274 | 0.9627134 |
| ENSG00000166529.14_2 | ZSCAN21 | chr7:996473+ | protein_codi-0.430172 | 0.7421732 | 0.0454329 | 0.3217476 | 2.4226978 | 2.85287 | 3.7015702 | 5.5181669 | 4.0297424 | 5.9783578 | 6.0522648 | 6.6615473 |
| ENSG00000166532.15_4 | RIMKLB | chr12:88341+ | protein_codi-0.472301 | 0.7208138 | 0.0495595 | 0.3295165 | 0.6128103 | 1.0851117 | 0.5195814 | 0.4875431 | 0.5820831 | 0.8435754 | 1.6714155 | 0.9388811 |
| ENSG00000166669.13_3 | ATF7IP2 | chr16:10420+ | protein_codi-0.301313 | 0.8115136 | 0.042519 | 0.3149427 | 1.9503758 | 2.2516887 | 3.1391124 | 2.8802905 | 2.5941269 | 3.3815015 | 4.3275465 | 3.6272958 |
| ENSG00000166750.9_2 | SLFN5 | chr17:33570+ | protein_codi-0.288344 | 0.8188412 | 0.017134 | 0.2499474 | 0.8674413 | 1.1557857 | 0.8728979 | 0.8662507 | 0.7373745 | 1.1380903 | 1.4358055 | 1.1237814 |
| ENSG00000166783.21_4 | MARF1 | chr16:15688- | protein_codi-0.360744 | 0.7787628 | 0.0060165 | 0.2000693 | 2.193209 | 2.5539531 | 3.4238427 | 3.9627528 | 3.3565505 | 4.8112035 | 5.1349078 | 4.6803532 |
| ENSG00000166788.9_4 | SAAL1 | chr11:18091- | protein_codi-0.29603 | 0.8144908 | 0.0037092 | 0.1780801 | 2.3443297 | 2.6403594 | 3.9421074 | 4.4125096 | 3.8958696 | 5.3474914 | 5.2450911 | 5.1142227 |
| ENSG00000166851.14_3 | PLK1 | chr16:23688+ | protein_codi0.3157578 | 1.2446652 | 0.0017888 | 0.1503852 | 4.7558749 | 4.4401172 | 26.233641 | 25.791965 | 26.031663 | 22.008104 | 20.19148 | 19.978925 |
| ENSG00000166855.9_2 | CLPX | chr15:65440- | protein_codi-0.299492 | 0.8125386 | 0.0266218 | 0.2810959 | 3.3816522 | 3.681144 | 9.6711492 | 10.14151 | 8.5231576 | 11.123368 | 12.805212 | 11.610663 |
| ENSG00000166866.12_3 | MYO1A | chr12:57422- | protein_codi-0.339738 | 0.7901849 | 0.0463469 | 0.3235145 | 1.3150921 | 1.6548298 | 1.3380358 | 1.7938921 | 1.3582274 | 2.3797179 | 2.2970987 | 1.8018691 |
| ENSG00000167065.13_3 | DUSP18 | chr22:31048- | protein_codi-0.312654 | 0.8051592 | 0.020787 | 0.2615337 | 1.259125 | 1.5717791 | 1.5578513 | 1.3331013 | 1.2977052 | 2.2438836 | 1.7490316 | 1.9458657 |
| ENSG00000167106.11_2 | FAM102A | chr9:130702- | protein_codi0.6568796 | 1.5766688 | 0.0499422 | 0.3303053 | 3.4289607 | 2.7720811 | 9.1308364 | 9.9145193 | 10.298224 | 5.1991618 | 8.3501883 | 4.4990206 |
| ENSG00000167136.6_2 | ENDOG | chr9:131580+ | protein_codi0.2774001 | 1.2120088 | 0.0176446 | 0.2506617 | 4.1236272 | 3.846227 | 16.727919 | 15.13783 | 17.514139 | 13.900233 | 13.678406 | 12.602451 |
| ENSG00000167183.2_2 | PRR15L | chr17:46029- | protein_codi-0.367426 | 0.775164 | 0.01351 | 0.2376659 | 4.2949824 | 4.6624088 | 18.485855 | 17.525925 | 19.953482 | 24.609051 | 22.158556 | 26.38229 |
| ENSG00000167191.11_3 | GPRC5B | chr16:19868- | protein_codi0.3082684 | 1.2382206 | 0.0151265 | 0.2436202 | 1.8114094 | 1.503141 | 2.543008 | 2.2649417 | 2.7378176 | 2.0307194 | 1.6991653 | 1.784174 |
| ENSG00000167528.12_3 | ZNF641 | chr12:48730- | protein_codi-0.363079 | 0.7775036 | 0.002569 | 0.1603768 | 1.6043776 | 1.9674564 | 2.0237065 | 2.0966756 | 2.0023548 | 2.6385089 | 3.025038 | 3.0841034 |
| ENSG00000167604.14_4 | NFKBID | chr19:36378- | protein_codi0.2754727 | 1.2103906 | 0.0230602 | 0.2666812 | 2.5698459 | 2.2943732 | 5.1907689 | 4.4348694 | 5.2211179 | 3.8459337 | 3.6935522 | 4.1897492 |
| ENSG00000167644.11_3 | C19orf33 | chr19:38794+ | protein_codi0.3550464 | 1.2790267 | 0.0425525 | 0.3149427 | 7.7606057 | 7.4055593 | 206.74872 | 196.57515 | 247.45894 | 178.30787 | 153.50363 | 174.93344 |
| ENSG00000167680.15_2 | SEMA6B | chr19:45426- | protein_codi-0.427187 | 0.7437103 | 0.0090962 | 0.2129568 | 0.6814102 | 1.1085976 | 0.5890362 | 0.4713797 | 0.7640716 | 1.3023747 | 1.0548326 | 1.1193903 |
| ENSG00000167685.14_3 | ZNF444 | chr19:56643+ | protein_codi0.5091515 | 1.4232129 | 0.0313423 | 0.2933365 | 2.6893642 | 2.1802127 | 5.7832955 | 5.6967453 | 4.9079035 | 4.5163202 | 3.2067549 | 3.0117293 |
| ENSG00000167767.13_2 | KRT80 | chr12:52562- | protein_codi0.6247759 | 1.5419713 | 0.0410856 | 0.3138435 | 4.1794869 | 3.554711 | 15.09442 | 14.984623 | 22.124642 | 12.776361 | 9.7297665 | 9.9773901 |
| ENSG00000167779.7_2 | IGFBP6 | chr12:53491+ | protein_codi0.5338223 | 1.4477598 | 0.0010882 | 0.130843 | 6.0492398 | 5.5154175 | 61.397796 | 65.619428 | 68.861468 | 47.486308 | 43.207659 | 43.647965 |
| ENSG00000167815.11_3 | PRDX2 | chr19:12907- | protein_codi0.5618462 | 1.476157 | 0.0473075 | 0.3240376 | 5.7893182 | 5.227472 | 63.377727 | 61.198158 | 41.243717 | 37.902598 | 34.627388 | 36.941545 |
| ENSG00000167900.11_2 | TK1 | chr17:76170- | protein_codi0.2749822 | 1.2099792 | 0.0460259 | 0.3226997 | 6.2620208 | 5.9870386 | 72.418446 | 74.794488 | 80.231692 | 69.949833 | 56.887956 | 61.129095 |
| ENSG00000168209.4_2 | DDIT4 | chr10:74033+ | protein_codi-2.408361 | 0.1883697 | 1.383E-05 | 0.061109 | 3.1179346 | 5.526296 | 7.6269983 | 7.9696176 | 7.4555506 | 47.225614 | 39.702691 | 48.870272 |
| ENSG00000168394.10_2 | TAP1 | chr6:328129- | protein_codi0.3597343 | 1.2831895 | 0.0160559 | 0.2484394 | 3.7802674 | 3.4205331 | 13.311691 | 11.197082 | 13.858493 | 10.009368 | 9.4794383 | 9.6401771 |
| ENSG00000168672.3_2 | FAM84B | chr8:127564- | protein_codi-0.621918 | 0.6498065 | 0.0001926 | 0.0943724 | 3.1125457 | 3.7344636 | 8.0361297 | 7.8211623 | 7.117094 | 12.4198 | 12.274784 | 12.236805 |
| ENSG00000168679.17_3 | SLC16A4 | chr1:110905- | protein_codi-0.275675 | 0.826064 | 0.016589 | 0.2498875 | 2.0486315 | 2.324306 | 3.1161917 | 3.4213147 | 2.8909132 | 3.946633 | 3.7820831 | 4.3104423 |
| ENSG00000168710.17_2 | AHCYL1 | chr1:110527+ | protein_codi0.3529997 | 1.2772135 | 0.0063541 | 0.2040161 | 4.6133834 | 4.2603837 | 21.461332 | 24.224778 | 24.884365 | 18.800322 | 17.978209 | 17.731925 |
| ENSG00000168765.16_2 | GSTM4 | chr1:110198+ | protein_codi0.4313753 | 1.3485185 | 0.0003097 | 0.0943724 | 4.9738082 | 4.5424329 | 30.277894 | 30.677458 | 30.319047 | 23.186922 | 21.20236 | 22.563831 |
| ENSG00000168916.15_3 | ZNF608 | chr5:123972- | protein_codi-0.351883 | 0.7835609 | 0.0032825 | 0.1710595 | 1.767506 | 2.1193886 | 2.5942947 | 2.4445015 | 2.1876961 | 3.2216574 | 3.4705611 | 3.3466397 |
| ENSG00000168944.15_3 | CEP120 | chr5:122680- | protein_codi-0.441975 | 0.7361262 | 0.0200818 | 0.2575998 | 2.1207881 | 2.5627631 | 3.825733 | 3.5223105 | 2.7699781 | 4.6636566 | 5.336558 | 4.7471877 |
| ENSG00000169223.14_2 | LMAN2 | chr5:176758- | protein_codi0.2717691 | 1.2072874 | 0.0175635 | 0.2506617 | 5.5739757 | 5.3022066 | 48.907581 | 47.515103 | 43.643491 | 40.970022 | 36.198338 | 38.346576 |
| ENSG00000169504.14_2 | CLIC4 | chr1:250718+ | protein_codi-0.625088 | 0.6483802 | 0.0380103 | 0.3079885 | 4.1239723 | 4.7490602 | 19.783108 | 18.078385 | 12.367985 | 23.640756 | 27.253134 | 26.932388 |
| ENSG00000169627.7_2 | BOLA2B | chr16:30204- | protein_codi0.3428778 | 1.268284 | 0.0109701 | 0.2246724 | 4.7404156 | 4.3975378 | 24.754059 | 24.503276 | 28.079049 | 20.455915 | 18.802442 | 21.034671 |
| ENSG00000169689.14_3 | CENPX | chr17:79976- | protein_codi0.2959354 | 1.2276807 | 0.018603 | 0.2558547 | 4.80796 | 4.5120246 | 26.808877 | 26.126313 | 28.1371 | 24.1161 | 20.333346 | 21.169355 |
| ENSG00000169826.7_2 | CSGALNACT2 | chr10:43633+ | protein_codi-0.354779 | 0.7819896 | 0.0293342 | 0.2885442 | 1.5105441 | 1.8653228 | 1.819489 | 2.213191 | 1.552997 | 2.567498 | 2.882298 | 2.492221 |
| ENSG00000169914.5_2 | OTUD3 | chr1:202090+ | protein_codi-0.279098 | 0.8241063 | 0.0322697 | 0.2958398 | 2.4219427 | 2.7010403 | 4.340249 | 4.8075287 | 3.9622575 | 5.3463762 | 6.0223465 | 5.1698471 |
| ENSG00000170296.9_3 | GABARAP | chr17:71433- | protein_codi-0.271197 | 0.8286316 | 0.0077945 | 0.2080856 | 3.7571787 | 4.0283759 | 13.518377 | 12.20904 | 11.890836 | 15.692488 | 15.174488 | 15.092899 |
| ENSG00000170373.8_2 | CST1 | chr20:23728- | protein_codi0.5644242 | 1.4787972 | 0.0119805 | 0.2323938 | 8.8453794 | 8.2809552 | 482.0116 | 398.28351 | 503.58537 | 342.74683 | 304.72168 | 285.34053 |
| ENSG00000170477.12_2 | KRT4 | chr12:53200- | protein_codi-0.812139 | 0.5695368 | 0.0360436 | 0.3040329 | 0.2793048 | 1.0914437 | 0.2894035 | 0.2812823 | 0.0819412 | 1.073883 | 1.9066073 | 0.6050969 |
| ENSG00000170689.9_3 | HOXB9 | chr17:46698- | protein_codi-0.504168 | 0.7050667 | 0.0053735 | 0.1925603 | 3.2168306 | 3.720999 | 8.153584 | 8.828428 | 7.933308 | 13.51693 | 10.922612 | 12.248014 |
| ENSG00000170727.7 | BOP1 | chr8:145486- | protein_codi0.3176313 | 1.2462826 | 0.0149937 | 0.2432408 | 4.0641306 | 3.7464993 | 14.919667 | 15.671149 | 16.635027 | 13.154955 | 11.273247 | 12.917398 |
| ENSG00000170848.15_3 | PSG6 | chr19:43406- | protein_codi-0.33938 | 0.7903811 | 0.0031967 | 0.1692945 | 2.9209862 | 3.2603658 | 6.5662621 | 6.1172713 | 7.06712 | 8.5218241 | 8.7478955 | 8.4792217 |
| ENSG00000170871.11_2 | KIAA0232 | chr4:678310+ | protein_codi-0.369052 | 0.7742913 | 0.0435727 | 0.3170949 | 3.0245394 | 3.3935911 | 7.4608067 | 7.7265419 | 6.2975542 | 8.468879 | 10.952249 | 9.2558101 |
| ENSG00000171109.18_3 | MFN1 | chr3:179065+ | protein_codi-0.322484 | 0.799692 | 0.0334105 | 0.2978369 | 2.1012874 | 2.4237711 | 3.5467764 | 3.57146 | 2.800958 | 4.0377404 | 4.69343 | 4.3860905 |
| ENSG00000171224.8_2 | C10orf35 | chr10:71390+ | protein_codi0.3667352 | 1.2894316 | 0.0028689 | 0.162509 | 3.2799991 | 2.9132639 | 8.3863118 | 8.6857075 | 9.0811026 | 7.0030196 | 6.1402275 | 6.4812394 |
| ENSG00000171401.14_3 | KRT13 | chr17:39657- | protein_codi-0.549678 | 0.6831724 | 0.0284997 | 0.2885442 | 1.2577229 | 1.8074013 | 1.3272647 | 1.3581009 | 1.4913156 | 3.0792105 | 2.7328112 | 1.8160082 |
| ENSG00000171448.8_2 | ZBTB26 | chr9:125677- | protein_codi-0.281183 | 0.822916 | 0.0354512 | 0.3025562 | 1.2305193 | 1.5117023 | 1.4911194 | 1.4385056 | 1.1269212 | 1.6482923 | 2.0048263 | 1.9135197 |
| ENSG00000171522.5_2 | PTGER4 | chr5:406796+ | protein_codi0.270149 | 1.2059324 | 0.0416089 | 0.3140442 | 1.0640743 | 0.7939253 | 1.0189537 | 1.2191402 | 1.040064 | 0.6722596 | 0.9343105 | 0.6112248 |
| ENSG00000171557.16_4 | FGG | chr4:155525- | protein_codi-0.452118 | 0.730969 | 0.0080447 | 0.2096161 | 4.4131146 | 4.8652325 | 21.327465 | 20.793584 | 18.873372 | 25.335513 | 30.532975 | 28.81508 |
| ENSG00000171560.14_2 | FGA | chr4:155504- | protein_codi-0.544083 | 0.6858272 | 0.0002904 | 0.0943724 | 3.1253841 | 3.6694671 | 7.8966651 | 7.3645922 | 7.9295801 | 11.274096 | 12.307098 | 11.612062 |
| ENSG00000171564.11_2 | FGB | chr4:155484+ | protein_codi-0.487324 | 0.7133471 | 0.0138778 | 0.2388191 | 3.2152231 | 3.702547 | 8.7751545 | 8.3951095 | 7.7218836 | 10.910739 | 14.062958 | 11.299407 |
| ENSG00000171843.15_2 | MLLT3 | chr9:203416- | protein_codi-0.396679 | 0.7596048 | 0.0215595 | 0.2632991 | 1.4569071 | 1.8535862 | 1.8890823 | 1.8625279 | 1.5015465 | 2.2175776 | 2.7645836 | 2.8968092 |
| ENSG00000171848.14_3 | RRM2 | chr2:102608+ | protein_codi0.3090118 | 1.2388589 | 0.0325695 | 0.2958398 | 3.5775995 | 3.2685876 | 10.639732 | 12.447342 | 9.872134 | 9.0766731 | 8.3411319 | 8.5085016 |
| ENSG00000171940.13_3 | ZNF217 | chr20:52183- | protein_codi-0.29119 | 0.8172278 | 0.0072573 | 0.2074702 | 4.3951205 | 4.6863102 | 20.978097 | 20.682126 | 18.547775 | 24.281108 | 25.535398 | 24.441232 |
| ENSG00000171988.18_3 | JMJD1C | chr10:64926- | protein_codi-0.496913 | 0.7086212 | 0.0471998 | 0.3240376 | 2.6265552 | 3.1234686 | 5.6499838 | 5.8441893 | 4.1745564 | 6.739988 | 9.2264917 | 7.3619248 |
| ENSG00000172009.14_2 | THOP1 | chr19:27854+ | protein_codi0.2736247 | 1.2088411 | 0.0025917 | 0.1609734 | 3.4912174 | 3.2175927 | 10.719183 | 10.041311 | 9.9891848 | 8.4158702 | 7.9563433 | 8.5451896 |
| ENSG00000172086.7_2 | KRCC1 | chr2:883267- | protein_codi-0.434498 | 0.7399511 | 0.0019031 | 0.1517515 | 2.4508531 | 2.8853512 | 4.633339 | 4.532235 | 4.244141 | 5.885288 | 6.613715 | 6.695078 |
| ENSG00000172216.5_2 | CEBPB | chr20:48807+ | protein_codi-0.664695 | 0.6308222 | 0.0032804 | 0.1710595 | 5.1095357 | 5.7742303 | 32.952267 | 33.166557 | 34.473213 | 59.696991 | 46.645092 | 55.68449 |
| ENSG00000172292.14_3 | CERS6 | chr2:169312+ | protein_codi-0.36002 | 0.7791538 | 0.0450112 | 0.3203624 | 3.6841583 | 4.0441783 | 12.955357 | 12.663442 | 10.138465 | 14.407732 | 17.113856 | 15.088193 |
| ENSG00000172336.4_2 | POP7 | chr7:100303+ | protein_codi0.2966792 | 1.2283138 | 0.0299085 | 0.2902373 | 5.8571492 | 5.5604701 | 57.057672 | 51.983601 | 62.318665 | 49.591505 | 44.973125 | 44.188008 |
| ENSG00000172478.17_4 | C2orf54 | chr2:241825- | protein_codi0.4001885 | 1.3196803 | 0.0265952 | 0.2810959 | 0.8332943 | 0.4331058 | 0.9887294 | 0.8351852 | 0.5498303 | 0.4443866 | 0.3130665 | 0.2976674 |
| ENSG00000172738.11_2 | TMEM217 | chr6:371799- | protein_codi-0.316447 | 0.8030453 | 0.0333004 | 0.2974117 | 0.6621529 | 0.9785996 | 0.3887903 | 0.6630868 | 0.7156633 | 0.9095899 | 1.0444221 | 0.9599855 |
| ENSG00000172765.16_3 | TMCC1 | chr3:129366- | protein_codi-0.472589 | 0.7206701 | 0.0236305 | 0.2689009 | 1.958533 | 2.4311221 | 2.9446509 | 2.7510442 | 2.967992 | 4.9463952 | 4.858598 | 3.5027163 |
| ENSG00000172766.18_2 | NAA16 | chr13:41885+ | protein_codi-0.330753 | 0.7951215 | 0.0280159 | 0.2865151 | 1.8248362 | 2.1555889 | 2.2080847 | 2.7283967 | 2.7172636 | 3.1224791 | 3.8419437 | 3.4311102 |
| ENSG00000173166.17_2 | RAPH1 | chr2:204259- | protein_codi-0.419747 | 0.7475557 | 0.0136971 | 0.2388191 | 3.4852577 | 3.9050047 | 10.56829 | 11.172832 | 8.9733377 | 13.133344 | 14.951474 | 13.911623 |
| ENSG00000173273.15_2 | TNKS | chr8:941342+ | protein_codi-0.328403 | 0.7964178 | 0.0086766 | 0.2115899 | 2.4768016 | 2.8052043 | 5.0078188 | 4.2185116 | 4.5018818 | 5.6834125 | 6.24776 | 6.0493626 |
| ENSG00000173276.13_2 | ZBTB21 | chr21:43406- | protein_codi-0.629302 | 0.6464889 | 0.0001029 | 0.0866373 | 1.7349691 | 2.3642716 | 2.1846635 | 2.3695153 | 2.4371796 | 4.0752484 | 4.3226325 | 4.0531964 |
| ENSG00000173334.3_2 | TRIB1 | chr8:126442+ | protein_codi0.2691247 | 1.2050764 | 0.0020548 | 0.1522433 | 3.0639771 | 2.7948525 | 7.3892619 | 7.0865475 | 7.6210502 | 6.0910248 | 6.0460947 | 5.688763 |
| ENSG00000173530.5_2 | TNFRSF10D | chr8:229931- | protein_codi0.2679843 | 1.2041243 | 0.0003063 | 0.0943724 | 3.3963886 | 3.1284043 | 9.734897 | 9.394261 | 9.462924 | 7.922022 | 7.769252 | 7.546824 |
| ENSG00000173614.13_3 | NMNAT1 | chr1:100034+ | protein_codi-0.287864 | 0.8191138 | 0.007538 | 0.2074702 | 0.9097451 | 1.1976094 | 0.9276076 | 0.9554878 | 0.7591707 | 1.2603926 | 1.3965794 | 1.2272725 |
| ENSG00000173681.16_4 | BCLAF3 | chrX:19930- | protein_codi-0.271596 | 0.8284023 | 0.018731 | 0.2558547 | 0.7085672 | 0.9801636 | 0.7273104 | 0.6383359 | 0.5421497 | 0.8341605 | 1.0641811 | 1.0276398 |
| ENSG00000173928.2_2 | SWSAP1 | chr19:11485+ | protein_codi0.3793441 | 1.3007504 | 0.0172132 | 0.2500978 | 2.1104041 | 1.73106 | 3.65689 | 2.860118 | 3.479074 | 2.437028 | 2.438864 | 2.095315 |
| ENSG00000174109.4_3 | C16orf91 | chr16:14697- | protein_codi0.3007925 | 1.2318209 | 0.0127937 | 0.2349473 | 3.7653991 | 3.4646066 | 12.678306 | 11.764253 | 13.403448 | 10.474409 | 10.361088 | 9.3205119 |
| ENSG00000174136.11_2 | RGMB | chr5:981043+ | protein_codi-0.426291 | 0.7441726 | 0.0040584 | 0.182744 | 2.7844582 | 3.210749 | 6.0259977 | 6.2692787 | 5.4034937 | 8.1162188 | 7.8355808 | 8.8524777 |
| ENSG00000174236.3_2 | REP15 | chr12:27849+ | protein_codi-0.363128 | 0.777477 | 0.025309 | 0.2765412 | 0.5779675 | 0.9410956 | 0.547922 | 0.392418 | 0.543256 | 0.769607 | 1.175142 | 0.838779 |
| ENSG00000174442.11_2 | ZWILCH | chr15:66797+ | protein_codi-0.320071 | 0.8010302 | 0.0191913 | 0.2572494 | 2.6810447 | 3.0011162 | 5.9773775 | 5.4539276 | 4.8574466 | 7.4501936 | 6.9478116 | 6.6412383 |
| ENSG00000174485.15_3 | DENND4A | chr15:65950- | protein_codi-0.382393 | 0.7671641 | 0.0282917 | 0.2879695 | 1.0880566 | 1.4704494 | 1.0653048 | 1.4132368 | 0.9276533 | 1.5458052 | 1.90577 | 1.8764828 |
| ENSG00000174547.13_4 | MRPL11 | chr11:66202- | protein_codi0.2964113 | 1.2280858 | 0.0286146 | 0.2885442 | 4.3883849 | 4.0919735 | 21.980115 | 18.013468 | 20.022912 | 15.482993 | 15.699485 | 17.016946 |
| ENSG00000175084.11_2 | DES | chr2:220283+ | protein_codi-0.328022 | 0.7966278 | 0.0224252 | 0.2659269 | 2.1352977 | 2.46332 | 3.0973398 | 3.4301334 | 3.6714015 | 5.0048638 | 4.0498715 | 4.5311594 |
| ENSG00000175166.16_3 | PSMD2 | chr3:184016+ | protein_codi0.2653278 | 1.2019091 | 0.0477392 | 0.3250323 | 5.0651657 | 4.7998379 | 33.967995 | 32.078762 | 31.439967 | 24.861868 | 25.572428 | 30.448101 |
| ENSG00000175197.11_3 | DDIT3 | chr12:57910- | protein_codi-0.8637 | 0.5495414 | 0.0034528 | 0.1733337 | 2.5020208 | 3.3657208 | 4.4396945 | 5.2983245 | 4.3057939 | 9.058596 | 8.0921529 | 10.976906 |
| ENSG00000175324.9_2 | LSM1 | chr8:380208- | protein_codi0.5308178 | 1.4447479 | 0.0007062 | 0.114143 | 4.0157618 | 3.484944 | 15.164158 | 15.264071 | 15.099482 | 10.810977 | 9.3796234 | 10.448553 |
| ENSG00000175455.14_4 | CCDC14 | chr3:123616- | protein_codi-0.444683 | 0.7347456 | 0.0382279 | 0.3085154 | 1.3762318 | 1.8209149 | 1.9548081 | 1.5179323 | 1.3511903 | 2.1762119 | 3.0859484 | 2.3981904 |
| ENSG00000175592.8_3 | FOSL1 | chr11:65659- | protein_codi0.6326897 | 1.5504529 | 0.0080415 | 0.2096161 | 3.9047846 | 3.2720949 | 14.869991 | 11.676252 | 15.703379 | 8.1295755 | 8.8418312 | 9.033898 |
| ENSG00000175701.10_3 | LINC00116 | chr2:110969- | protein_codi0.3001743 | 1.2312932 | 0.0160035 | 0.2484394 | 3.4364793 | 3.136305 | 9.2604164 | 9.5388232 | 10.735267 | 8.2061929 | 7.2727899 | 7.925521 |
| ENSG00000175793.11_3 | SFN | chr1:271896+ | protein_codi0.2699182 | 1.2057395 | 0.0326208 | 0.2958398 | 7.7934372 | 7.523519 | 213.99838 | 208.84763 | 241.01143 | 197.05116 | 173.24484 | 179.50006 |
| ENSG00000175832.12_3 | ETV4 | chr17:41605- | protein_codi0.3097202 | 1.2394673 | 0.0073385 | 0.2074702 | 2.919732 | 2.6100118 | 6.5262046 | 6.3741396 | 6.807165 | 5.3654722 | 4.6440235 | 5.3336798 |
| ENSG00000175866.15_3 | BAIAP2 | chr17:79008+ | protein_codi0.2929051 | 1.2251047 | 0.0067689 | 0.2043689 | 2.4524125 | 2.1595074 | 4.4657097 | 4.597805 | 4.3590134 | 3.2939469 | 3.3135505 | 3.8143532 |
| ENSG00000176105.13_3 | YES1 | chr18:72158- | protein_codi-0.667017 | 0.6298074 | 0.0048937 | 0.1875771 | 3.7374907 | 4.4045081 | 12.88154 | 12.663569 | 11.510904 | 17.990582 | 23.480771 | 19.431667 |
| ENSG00000176532.3_2 | PRR15 | chr7:296034+ | protein_codi0.3283705 | 1.2555944 | 0.0088252 | 0.211819 | 3.9392101 | 3.6108396 | 13.227897 | 14.344626 | 15.533477 | 11.527919 | 10.746999 | 11.391024 |
| ENSG00000176624.10_2 | MEX3C | chr18:48700- | protein_codi-0.299215 | 0.8126946 | 0.0399051 | 0.3123853 | 2.3506814 | 2.6498962 | 3.8492483 | 4.6697836 | 3.8265404 | 4.8133793 | 5.7814115 | 5.271132 |
| ENSG00000176809.10_3 | LRRC37A3 | chr17:62850- | protein_codi-0.297444 | 0.8136927 | 0.0107095 | 0.2229358 | 0.3670876 | 0.6645317 | 0.2246081 | 0.3879275 | 0.2622611 | 0.6683583 | 0.5372467 | 0.5527409 |
| ENSG00000177169.9_3 | ULK1 | chr12:13237+ | protein_codi-0.328548 | 0.7963378 | 0.0301021 | 0.2902479 | 2.6460366 | 2.9745843 | 5.1581798 | 4.9811612 | 5.6584222 | 7.7354325 | 6.0649281 | 6.8691005 |
| ENSG00000177311.11_4 | ZBTB38 | chr3:141043+ | protein_codi-0.578207 | 0.6697956 | 0.0435332 | 0.3170949 | 3.5318295 | 4.1100366 | 10.799649 | 8.8965883 | 12.249676 | 19.903303 | 16.145794 | 13.366811 |
| ENSG00000177469.12_3 | CAVIN1 | chr17:40554- | protein_codi0.3107377 | 1.2403418 | 0.0147881 | 0.2432408 | 4.435763 | 4.1250252 | 20.478191 | 20.529999 | 20.920549 | 17.703861 | 14.75773 | 17.023705 |
| ENSG00000177700.5_2 | POLR2L | chr11:83735- | protein_codi0.2643116 | 1.2010628 | 0.0125751 | 0.2343664 | 6.7620675 | 6.4977559 | 106.06751 | 104.28036 | 112.43586 | 95.676961 | 84.47366 | 88.3106 |
| ENSG00000177853.14_3 | ZNF518A | chr10:97889+ | protein_codi-0.607933 | 0.6561362 | 0.0327585 | 0.2960127 | 1.6117507 | 2.2196836 | 2.3562296 | 2.3490106 | 1.539731 | 2.8645082 | 4.1356059 | 4.0919962 |
| ENSG00000177854.7_3 | TMEM187 | chrX:15323+ | protein_codi-0.278034 | 0.8247139 | 0.0417466 | 0.3140442 | 0.9635381 | 1.2415724 | 0.8978576 | 0.9484126 | 1.0054742 | 1.6466079 | 1.3489575 | 1.1266052 |
| ENSG00000178537.9_2 | SLC25A20 | chr3:488943- | protein_codi0.3510889 | 1.275523 | 0.0030728 | 0.1668001 | 3.491128 | 3.140039 | 10.685769 | 10.171521 | 9.8901306 | 8.3839345 | 7.4426951 | 7.6471246 |
| ENSG00000179195.15_3 | ZNF664 | chr12:12445+ | protein_codi-0.274345 | 0.8268255 | 0.0381731 | 0.3085154 | 4.3015809 | 4.5759261 | 20.008049 | 19.651082 | 16.676086 | 22.54752 | 24.23785 | 21.828401 |
| ENSG00000180061.9_2 | TMEM150B | chr19:55824- | protein_codi-0.311554 | 0.8057734 | 0.0470558 | 0.3240376 | 0.6660538 | 0.9776077 | 0.7053884 | 0.5887974 | 0.4743979 | 1.1069835 | 0.7344898 | 1.0894637 |
| ENSG00000180626.9_3 | ZNF594 | chr17:50828- | protein_codi-0.272314 | 0.8279902 | 0.0313975 | 0.2934744 | 1.3620692 | 1.6343837 | 1.6307393 | 1.6236067 | 1.460903 | 1.8512376 | 2.4299811 | 2.0596404 |
| ENSG00000180644.7_2 | PRF1 | chr10:72357- | protein_codi-0.598618 | 0.660386 | 0.0085444 | 0.2112786 | 1.1543845 | 1.7530029 | 1.042844 | 1.069451 | 1.6086915 | 2.5842941 | 2.1962359 | 2.3425426 |
| ENSG00000180745.4_2 | CLRN3 | chr10:12967- | protein_codi-0.359932 | 0.7792012 | 0.0117411 | 0.23143 | 1.1953503 | 1.5552826 | 1.337178 | 1.47899 | 1.072728 | 1.819998 | 2.025241 | 1.975441 |
| ENSG00000181026.14_2 | AEN | chr15:89164+ | protein_codi0.2972144 | 1.2287696 | 0.0007687 | 0.1159361 | 4.7659961 | 4.4687817 | 25.137316 | 26.824807 | 26.69684 | 20.768821 | 21.511853 | 21.154701 |
| ENSG00000181192.11_3 | DHTKD1 | chr10:12110+ | protein_codi-0.289481 | 0.8181961 | 0.0070964 | 0.2060586 | 3.3073014 | 3.5967828 | 9.1240545 | 9.3866179 | 8.2249096 | 10.721002 | 11.218565 | 11.366124 |
| ENSG00000181333.11_2 | HEPHL1 | chr11:93754+ | protein_codi-0.455715 | 0.7291485 | 0.0075872 | 0.2074702 | 1.7422542 | 2.1979696 | 2.601497 | 2.486074 | 1.982584 | 3.420415 | 3.539548 | 3.813807 |
| ENSG00000181634.7_2 | TNFSF15 | chr9:117546- | protein_codi-0.372107 | 0.7726533 | 0.0224099 | 0.2659269 | 2.4946268 | 2.8667336 | 5.3672559 | 4.6400323 | 3.9846954 | 6.3157339 | 6.4025792 | 6.1660113 |
| ENSG00000181852.17_3 | RNF41 | chr12:56595- | protein_codi-0.317401 | 0.8025144 | 0.0478067 | 0.3253462 | 3.0110972 | 3.328498 | 7.3956752 | 7.9903964 | 5.9415623 | 9.2275762 | 8.882603 | 9.029728 |
| ENSG00000182149.20_3 | IST1 | chr16:71879+ | protein_codi-0.671917 | 0.6276723 | 0.0315724 | 0.2945658 | 3.123682 | 3.7955986 | 5.5433519 | 9.0115143 | 9.108015 | 12.690042 | 13.058421 | 12.913161 |
| ENSG00000182220.14_4 | ATP6AP2 | chrX:40438+ | protein_codi0.2990005 | 1.2302917 | 0.009482 | 0.2138428 | 1.6608174 | 1.361817 | 2.2852322 | 2.1559812 | 2.049068 | 1.7689608 | 1.4311472 | 1.5218265 |
| ENSG00000182687.3_3 | GALR2 | chr17:74070+ | protein_codi0.5650736 | 1.479463 | 0.0024477 | 0.1571562 | 1.0407196 | 0.475646 | 0.841364 | 1.167888 | 1.181157 | 0.405917 | 0.348246 | 0.418477 |
| ENSG00000182700.4_2 | IGIP | chr5:139505+ | protein_codi-0.539743 | 0.6878932 | 0.0025716 | 0.1603768 | 0.5775419 | 1.1172853 | 0.545013 | 0.574822 | 0.365868 | 1.104378 | 1.31944 | 1.091714 |
| ENSG00000182866.16_2 | LCK | chr1:327168+ | protein_codi-0.41092 | 0.7521434 | 0.0043454 | 0.1849138 | 0.4321111 | 0.8430315 | 0.419612 | 0.2864624 | 0.3448333 | 0.7434595 | 0.7060247 | 0.9406005 |
| ENSG00000183011.13_3 | NAA38 | chr17:77600- | protein_codi0.3066987 | 1.2368741 | 0.0323124 | 0.2958398 | 3.3722383 | 3.0655396 | 9.6111221 | 8.2023345 | 10.370389 | 7.3862646 | 7.0525542 | 7.6887225 |
| ENSG00000183248.11_2 | PRR36 | chr19:79336- | protein_codi-0.353238 | 0.7828254 | 0.0038805 | 0.182744 | 1.3571054 | 1.710343 | 1.567521 | 1.390639 | 1.7388 | 2.329953 | 2.282756 | 2.205656 |
| ENSG00000183255.11_2 | PTTG1IP | chr21:46269- | protein_codi-1.449905 | 0.3660454 | 1.09E-06 | 0.0082969 | 4.5630041 | 6.0129095 | 21.975725 | 22.827175 | 23.124679 | 65.098798 | 63.828519 | 61.840362 |
| ENSG00000183336.8_3 | BOLA2 | chr16:29454- | protein_codi0.3902101 | 1.3105842 | 0.0056134 | 0.1931245 | 3.7338699 | 3.3436598 | 11.630169 | 11.864196 | 13.495349 | 9.5084233 | 8.6704037 | 9.2954069 |
| ENSG00000183486.12_3 | MX2 | chr21:42733+ | protein_codi0.2680285 | 1.2041612 | 0.0064869 | 0.2040161 | 0.6919752 | 0.4239466 | 0.6691455 | 0.5122666 | 0.6702954 | 0.3074539 | 0.368759 | 0.3493002 |
| ENSG00000183615.5_2 | FAM167B | chr1:327128+ | protein_codi-0.39012 | 0.7630662 | 0.0021726 | 0.1530585 | 0.6294332 | 1.0195532 | 0.564563 | 0.54607 | 0.530427 | 1.141204 | 1.064952 | 0.884431 |
| ENSG00000183684.7_2 | ALYREF | chr17:79845- | protein_codi0.3099838 | 1.2396938 | 0.0080179 | 0.2096161 | 6.8335106 | 6.5235268 | 109.69299 | 110.09202 | 119.63463 | 96.485285 | 85.768357 | 91.051589 |
| ENSG00000183718.5_2 | TRIM52 | chr5:180681- | protein_codi-0.425056 | 0.74481 | 0.0217161 | 0.2637296 | 0.6808641 | 1.1059196 | 0.6454845 | 0.8057288 | 0.386549 | 1.2506607 | 1.1220181 | 1.0877852 |
| ENSG00000183762.12_2 | KREMEN1 | chr22:29469+ | protein_codi-0.302541 | 0.8108229 | 0.0051674 | 0.1896292 | 2.0657483 | 2.3682896 | 2.9760975 | 3.1485873 | 3.4483543 | 4.3227564 | 4.1858298 | 3.986821 |
| ENSG00000184009.9_3 | ACTG1 | chr17:79476- | protein_codi0.3329568 | 1.2595923 | 0.0378845 | 0.3079184 | 6.8209669 | 6.4880102 | 112.62226 | 110.36968 | 113.21328 | 97.473232 | 76.343818 | 93.953989 |
| ENSG00000184012.11_3 | TMPRSS2 | chr21:42836- | protein_codi-0.280544 | 0.8232804 | 0.0176837 | 0.2506617 | 1.2965232 | 1.5770675 | 1.6543734 | 1.4024228 | 1.3241583 | 2.0481811 | 2.0991037 | 1.8116224 |
| ENSG00000184047.16_3 | DIABLO | chr12:12269- | protein_codi0.3119566 | 1.2413902 | 0.032142 | 0.2958398 | 3.1741259 | 2.8621693 | 7.9676639 | 7.8337546 | 8.2831786 | 5.9724273 | 7.2666068 | 5.6693755 |
| ENSG00000184207.8_2 | PGP | chr16:22619- | protein_codi0.2905148 | 1.2230766 | 0.0217916 | 0.2638205 | 5.1090447 | 4.8185299 | 32.131023 | 32.08279 | 36.505083 | 29.172462 | 25.636334 | 26.956377 |
| ENSG00000184281.14_4 | TSSC4 | chr11:24217+ | protein_codi0.2898216 | 1.2224891 | 0.0021705 | 0.1530585 | 3.340273 | 3.0504514 | 9.6387983 | 9.0894679 | 8.6784599 | 7.407008 | 7.1672643 | 7.2815961 |
| ENSG00000184384.13_3 | MAML2 | chr11:95709- | protein_codi-0.320431 | 0.8008307 | 0.023569 | 0.2689009 | 1.3127694 | 1.6332002 | 1.5311445 | 1.7128945 | 1.232539 | 1.9555724 | 2.1378567 | 2.218489 |
| ENSG00000184445.11_3 | KNTC1 | chr12:12301+ | protein_codi-0.306049 | 0.8088539 | 0.0436486 | 0.3171923 | 2.3212085 | 2.6272575 | 4.2064855 | 4.3302417 | 3.4974734 | 4.6515483 | 5.7153818 | 5.2145802 |
| ENSG00000184465.15_3 | WDR27 | chr6:169857- | protein_codi-0.29673 | 0.8140958 | 0.0482816 | 0.3263809 | 1.5494258 | 1.8461553 | 2.1435146 | 1.6048659 | 2.0624574 | 2.3729592 | 2.9077077 | 2.5262349 |
| ENSG00000184557.4_3 | SOCS3 | chr17:76352- | protein_codi0.2650453 | 1.2016738 | 0.0063973 | 0.2040161 | 3.3711972 | 3.1061519 | 9.050489 | 9.171034 | 9.837825 | 7.658617 | 7.207864 | 7.983716 |
| ENSG00000184575.11_2 | XPOT | chr12:64798+ | protein_codi-0.448444 | 0.7328327 | 0.0304077 | 0.2906274 | 4.3112525 | 4.7596967 | 21.151761 | 20.146139 | 15.703583 | 24.335849 | 27.716687 | 26.32528 |
| ENSG00000184979.9_2 | USP18 | chr22:18632+ | protein_codi0.3936929 | 1.313752 | 0.0041441 | 0.183315 | 3.6786618 | 3.2849688 | 12.166682 | 10.929336 | 12.368109 | 8.716035 | 9.273362 | 8.277283 |
| ENSG00000185133.13_3 | INPP5J | chr22:31518+ | protein_codi-0.30757 | 0.8080018 | 0.0085667 | 0.2112786 | 0.7994552 | 1.1070247 | 0.7411721 | 0.6481242 | 0.8371659 | 1.0650337 | 1.1135698 | 1.2898088 |
| ENSG00000185201.16_3 | IFITM2 | chr11:30763+ | protein_codi-0.382554 | 0.7670785 | 0.038223 | 0.3085154 | 0.6519129 | 1.0344667 | 0.3793677 | 0.7609054 | 0.5970552 | 1.0954253 | 0.8598232 | 1.2053252 |
| ENSG00000185432.11_2 | METTL7A | chr12:51317+ | protein_codi-0.361242 | 0.7784939 | 0.0268529 | 0.2816132 | 0.8429882 | 1.2042306 | 0.7350631 | 0.834912 | 0.8128575 | 0.9989915 | 1.4485386 | 1.4992557 |
| ENSG00000185453.12_3 | C19orf68 | chr19:48673+ | protein_codi-0.271443 | 0.8284904 | 0.0098685 | 0.2166029 | 2.0465759 | 2.3180191 | 2.8651979 | 3.1247563 | 3.422544 | 4.0997021 | 3.8941047 | 3.9677733 |
| ENSG00000185499.16_3 | MUC1 | chr1:155158- | protein_codi-0.358682 | 0.7798766 | 0.0105301 | 0.2222478 | 2.9087382 | 3.2674204 | 6.0772149 | 6.1556883 | 7.3625469 | 8.8243393 | 8.5245587 | 8.5417158 |
| ENSG00000185561.9_2 | TLCD2 | chr17:16060- | protein_codi-0.314606 | 0.8040704 | 0.0050931 | 0.1896292 | 2.015357 | 2.3299633 | 3.03329 | 3.196692 | 2.903752 | 3.908168 | 3.82431 | 4.367992 |
| ENSG00000185745.9_2 | IFIT1 | chr10:91152+ | protein_codi0.3406232 | 1.2663034 | 0.0297605 | 0.2901126 | 4.6373212 | 4.296698 | 25.419943 | 22.628996 | 23.691143 | 16.383016 | 20.5101 | 19.301935 |
| ENSG00000185946.15_3 | RNPC3 | chr1:104068+ | protein_codi-0.342096 | 0.7888946 | 0.0124357 | 0.2343664 | 0.8539662 | 1.1960618 | 0.6730583 | 0.8454586 | 0.9124625 | 1.1307444 | 1.4163991 | 1.3358848 |
| ENSG00000186010.18_3 | NDUFA13 | chr19:19626+ | protein_codi0.3023918 | 1.2331872 | 0.0487418 | 0.3276076 | 4.6166656 | 4.3142738 | 21.984808 | 22.700521 | 26.106014 | 18.530626 | 17.276211 | 21.058455 |
| ENSG00000186193.8_2 | SAPCD2 | chr9:139956- | protein_codi0.2849356 | 1.2183558 | 0.0260875 | 0.2797561 | 5.1963277 | 4.9113922 | 34.62265 | 34.212173 | 38.294567 | 31.721151 | 26.983 | 28.765026 |
| ENSG00000186326.3_2 | RGS9BP | chr19:33166+ | protein_codi-0.275973 | 0.8258929 | 0.0287297 | 0.2885442 | 1.4087617 | 1.6847351 | 1.371036 | 1.79073 | 1.828672 | 2.260367 | 2.177855 | 2.206763 |
| ENSG00000186654.20_3 | PRR5 | chr22:45064+ | protein_codi0.5819136 | 1.4968334 | 0.0008045 | 0.1165864 | 2.4742219 | 1.8923083 | 4.6216269 | 4.4231852 | 4.6276694 | 3.0409375 | 2.5799031 | 2.5364804 |
| ENSG00000186814.13_3 | ZSCAN30 | chr18:32831- | protein_codi-0.276418 | 0.8256387 | 0.0285802 | 0.2885442 | 0.7414165 | 1.0178341 | 0.5747193 | 0.7320071 | 0.7132197 | 1.0548552 | 1.1846619 | 0.8493977 |
| ENSG00000186847.5_2 | KRT14 | chr17:39738- | protein_codi0.2837455 | 1.2173512 | 0.0041047 | 0.182744 | 0.7071314 | 0.4233859 | 0.5751159 | 0.6098244 | 0.7159801 | 0.3570325 | 0.3808712 | 0.2870998 |
| ENSG00000187094.11_2 | CCK | chr3:422993- | protein_codi0.305324 | 1.2356961 | 0.0026725 | 0.1614437 | 1.039459 | 0.734135 | 1.1140234 | 1.0218464 | 1.0317349 | 0.6416614 | 0.5956166 | 0.7570247 |
| ENSG00000187231.13_4 | SESTD1 | chr2:179966- | protein_codi-0.350936 | 0.784075 | 0.0109478 | 0.2246724 | 1.5658493 | 1.9167858 | 2.023409 | 2.1367106 | 1.7360971 | 2.5436056 | 3.0096735 | 2.7885728 |
| ENSG00000187240.13_2 | DYNC2H1 | chr11:10298+ | protein_codi-0.291356 | 0.8171338 | 0.0468411 | 0.3240376 | 0.5547405 | 0.8460962 | 0.4738884 | 0.5309737 | 0.4045858 | 0.6572481 | 1.0512565 | 0.708809 |
| ENSG00000187266.13_3 | EPOR | chr19:11487- | protein_codi-0.415116 | 0.749959 | 0.010662 | 0.2227482 | 0.9949235 | 1.4100399 | 1.1860573 | 0.8596338 | 0.9472264 | 1.4987543 | 1.8773511 | 1.6102164 |
| ENSG00000187288.10_2 | CIDEC | chr3:990839- | protein_codi-0.41213 | 0.7515128 | 0.0284034 | 0.2884935 | 0.8977872 | 1.3099176 | 0.8303998 | 0.9597482 | 0.8031688 | 1.7833666 | 1.1201901 | 1.5824284 |
| ENSG00000187608.8_4 | ISG15 | chr1:936518+ | protein_codi0.4147822 | 1.3330974 | 0.0022109 | 0.1540141 | 7.6879656 | 7.2731835 | 206.02176 | 191.20985 | 219.3602 | 151.20953 | 152.70856 | 157.19737 |
| ENSG00000187908.16_2 | DMBT1 | chr10:12432+ | protein_codi-0.391247 | 0.7624704 | 0.0019087 | 0.1517515 | 2.2901855 | 2.6814322 | 3.6179878 | 4.1216109 | 3.9474816 | 5.5360117 | 5.1438911 | 5.5738265 |
| ENSG00000188229.5_2 | TUBB4B | chr9:140135+ | protein_codi0.3655651 | 1.2883862 | 0.0026998 | 0.1614437 | 8.6661507 | 8.3005856 | 405.63748 | 389.1447 | 421.55265 | 333.01175 | 299.99707 | 310.7825 |
| ENSG00000188368.9_4 | PRR19 | chr19:42806+ | protein_codi0.3996834 | 1.3192184 | 0.0167271 | 0.2498875 | 1.744284 | 1.3446006 | 2.1994058 | 2.3726506 | 2.4850157 | 1.2556689 | 1.8272516 | 1.5683578 |
| ENSG00000188613.6_2 | NANOS1 | chr10:12078+ | protein_codi-0.355721 | 0.7814787 | 0.0003548 | 0.0943724 | 1.9806405 | 2.3363619 | 2.8443526 | 2.9779662 | 3.0198727 | 3.9638678 | 3.9637965 | 4.227703 |
| ENSG00000188643.10_2 | S100A16 | chr1:153579- | protein_codi0.3575363 | 1.2812361 | 0.0099868 | 0.2170995 | 6.4692368 | 6.1117004 | 93.483825 | 85.574591 | 84.026644 | 72.340738 | 69.869983 | 62.62208 |
| ENSG00000188784.4_2 | PLA2G2E | chr1:202465- | protein_codi-0.61741 | 0.65184 | 0.0355889 | 0.3030195 | 1.8838261 | 2.5012364 | 1.979643 | 3.395185 | 2.83816 | 5.306264 | 3.88257 | 4.894146 |
| ENSG00000188910.7_2 | GJB3 | chr1:352467+ | protein_codi0.367827 | 1.2904077 | 0.0109323 | 0.2246724 | 4.456319 | 4.088492 | 19.985655 | 20.890296 | 22.029415 | 17.554877 | 14.600123 | 16.009443 |
| ENSG00000188959.9_2 | C9orf152 | chr9:112952- | protein_codi-0.377682 | 0.7696731 | 0.0007316 | 0.114143 | 3.1826609 | 3.5603432 | 7.9971756 | 7.7417622 | 8.5175241 | 10.537076 | 11.057593 | 10.801955 |
| ENSG00000188976.10_2 | NOC2L | chr1:879584- | protein_codi0.2961597 | 1.2278716 | 0.0025714 | 0.1603768 | 5.2094236 | 4.9132639 | 34.77186 | 36.559881 | 36.697504 | 30.508807 | 27.843743 | 29.104811 |
| ENSG00000189056.13_3 | RELN | chr7:103112- | protein_codi-0.790317 | 0.5782172 | 3.847E-05 | 0.061109 | 1.5277896 | 2.3181062 | 1.9132888 | 1.9918223 | 1.7505034 | 3.9742785 | 3.8886261 | 4.0996644 |
| ENSG00000189159.15_4 | JPT1 | chr17:73131- | protein_codi0.3684083 | 1.2909277 | 0.0027741 | 0.162234 | 5.1784873 | 4.810079 | 35.519443 | 34.457847 | 35.677817 | 28.393446 | 25.054605 | 27.826982 |
| ENSG00000189221.9_2 | MAOA | chrX:43514+ | protein_codi-0.36772 | 0.7750063 | 0.0008705 | 0.1226544 | 1.128554 | 1.496274 | 1.2993013 | 1.1047311 | 1.1596993 | 1.8408996 | 1.7598402 | 1.8637179 |
| ENSG00000196233.13_4 | LCOR | chr10:98592+ | protein_codi-0.325325 | 0.7981183 | 0.0279876 | 0.2865151 | 2.4243894 | 2.7497149 | 4.4516159 | 4.8966339 | 3.8118536 | 5.3546283 | 6.0737119 | 5.7686558 |
| ENSG00000196247.11_3 | ZNF107 | chr7:641264+ | protein_codi-0.314469 | 0.8041471 | 0.0423945 | 0.3149427 | 1.0303883 | 1.344857 | 1.1115252 | 1.1146044 | 0.9085694 | 1.4054797 | 1.3523222 | 1.8961986 |
| ENSG00000196323.12_3 | ZBTB44 | chr11:13009- | protein_codi-0.383762 | 0.7664363 | 0.031488 | 0.2941392 | 1.8087234 | 2.1924857 | 2.931685 | 2.4208646 | 2.1968728 | 3.1011405 | 3.8936244 | 3.7585711 |
| ENSG00000196449.3_2 | YRDC | chr1:382686- | protein_codi0.3877606 | 1.3083609 | 0.0198531 | 0.2575012 | 3.8550546 | 3.4672941 | 12.265384 | 13.045941 | 15.262622 | 10.32734 | 9.267317 | 10.633051 |
| ENSG00000196497.16_3 | IPO4 | chr14:24649- | protein_codi0.2988974 | 1.2302038 | 0.0027268 | 0.1614437 | 5.4662033 | 5.167306 | 43.422904 | 40.921198 | 45.390907 | 35.429255 | 35.235304 | 34.152813 |
| ENSG00000196517.11_3 | SLC6A9 | chr1:444571- | protein_codi-0.818256 | 0.5671273 | 0.0014022 | 0.1390335 | 2.9067665 | 3.725022 | 5.9209134 | 6.0800973 | 7.6073423 | 12.135985 | 11.827199 | 12.722575 |
| ENSG00000196597.11_2 | ZNF782 | chr9:995787- | protein_codi-0.296589 | 0.8141753 | 0.0166558 | 0.2498875 | 0.9997878 | 1.2963763 | 0.9462389 | 0.8953267 | 1.1677944 | 1.3319108 | 1.6015739 | 1.4422853 |
| ENSG00000196605.7_3 | ZNF846 | chr19:98626- | protein_codi-0.295942 | 0.8145401 | 0.0309395 | 0.2922448 | 0.4761485 | 0.7720909 | 0.2750022 | 0.3792232 | 0.530592 | 0.6072113 | 0.7149537 | 0.8069335 |
| ENSG00000196793.13_2 | ZNF239 | chr10:44051- | protein_codi-0.53036 | 0.6923817 | 0.0367848 | 0.3055634 | 1.5142368 | 2.0445972 | 1.5357014 | 1.5436423 | 2.613582 | 2.9664606 | 3.2159361 | 3.1991179 |
| ENSG00000196981.3_3 | WDR5B | chr3:122131- | protein_codi-0.277343 | 0.825109 | 0.043291 | 0.3166579 | 1.2179387 | 1.4952821 | 1.381128 | 1.380496 | 1.220537 | 1.993002 | 2.003749 | 1.492319 |
| ENSG00000197019.4_2 | SERTAD1 | chr19:40927- | protein_codi0.4617919 | 1.3772514 | 0.0032685 | 0.1710595 | 4.2002936 | 3.7385017 | 17.391205 | 16.757317 | 18.021973 | 13.657864 | 11.687951 | 11.786193 |
| ENSG00000197062.11_3 | ZSCAN26 | chr6:282347+ | protein_codi-0.354803 | 0.7819766 | 0.0215891 | 0.2634495 | 1.7285048 | 2.0833075 | 2.5146783 | 2.1564151 | 2.2803162 | 2.9979659 | 3.7722159 | 2.9889171 |
| ENSG00000197110.8_2 | IFNL3 | chr19:39734- | protein_codi0.2910171 | 1.2235025 | 0.0047507 | 0.1853643 | 1.0037593 | 0.7127422 | 1.0609257 | 0.881788 | 1.0789878 | 0.6451432 | 0.5921876 | 0.6806313 |
| ENSG00000197142.10_2 | ACSL5 | chr10:11413+ | protein_codi-0.371223 | 0.773127 | 0.0003036 | 0.0943724 | 1.8283733 | 2.199596 | 2.6328026 | 2.4604244 | 2.5629889 | 3.7495138 | 3.5330072 | 3.5019179 |
| ENSG00000197183.14_4 | NOL4L | chr20:31030- | protein_codi-0.276184 | 0.8257726 | 0.012555 | 0.2343664 | 1.629267 | 1.9054505 | 1.8419705 | 2.282923 | 2.1731781 | 2.7920695 | 2.6821889 | 2.7653984 |
| ENSG00000197329.11_2 | PELI1 | chr2:643197- | protein_codi-0.285821 | 0.8202747 | 0.0424167 | 0.3149427 | 3.3427499 | 3.6285708 | 9.5707453 | 9.9671147 | 8.0075347 | 10.739289 | 12.019018 | 11.379582 |
| ENSG00000197353.3_2 | LYPD2 | chr8:143831- | protein_codi-0.45283 | 0.7306083 | 0.0105779 | 0.2222535 | 0.8565231 | 1.3093531 | 1.038579 | 0.659402 | 0.754843 | 1.331036 | 1.597327 | 1.514132 |
| ENSG00000197385.5_2 | ZNF860 | chr3:320232+ | protein_codi-0.436801 | 0.738771 | 0.0091758 | 0.2129568 | 0.9225568 | 1.3593576 | 0.9854211 | 0.9088071 | 0.7969546 | 1.3155818 | 1.8221 | 1.5845786 |
| ENSG00000197442.9_2 | MAP3K5 | chr6:136878- | protein_codi-0.362443 | 0.7778461 | 0.0335532 | 0.2981619 | 2.3773051 | 2.7397485 | 4.3895589 | 4.4286437 | 3.7937551 | 5.0194396 | 6.5898474 | 5.5230439 |
| ENSG00000197555.9_3 | SIPA1L1 | chr14:71787+ | protein_codi-0.316271 | 0.8031431 | 0.0002076 | 0.0943724 | 2.5391544 | 2.8554254 | 4.9444936 | 4.777057 | 4.7182531 | 6.1437041 | 6.4186769 | 6.1524723 |
| ENSG00000197785.13_2 | ATAD3A | chr1:144753+ | protein_codi0.3379193 | 1.2639324 | 0.008932 | 0.2129568 | 5.2044554 | 4.8665361 | 35.231884 | 35.422094 | 36.987062 | 30.493664 | 25.788062 | 28.427649 |
| ENSG00000197822.10_2 | OCLN | chr5:687881+ | protein_codi-0.293117 | 0.8161366 | 0.0362987 | 0.3048333 | 3.9221655 | 4.2152829 | 15.243573 | 14.658199 | 12.697616 | 16.289423 | 18.870561 | 17.654738 |
| ENSG00000197841.14_3 | ZNF181 | chr19:35225+ | protein_codi-0.358583 | 0.7799305 | 0.0253529 | 0.2765412 | 0.8547996 | 1.2133821 | 0.738875 | 1.0064137 | 0.6953996 | 1.2267171 | 1.192408 | 1.5539173 |
| ENSG00000197978.8 | GOLGA6L9 | chr15:82722+ | protein_codi-0.268934 | 0.8299323 | 0.0148557 | 0.2432408 | 0.548591 | 0.8175255 | 0.5547706 | 0.4377666 | 0.3998205 | 0.6997965 | 0.8816124 | 0.7114807 |
| ENSG00000198000.11_3 | NOL8 | chr9:950596- | protein_codi-0.316873 | 0.8028079 | 0.0480041 | 0.3253803 | 1.9587518 | 2.2756251 | 3.2804986 | 2.9269889 | 2.4944202 | 3.5876059 | 4.3643094 | 3.6131159 |
| ENSG00000198075.9_2 | SULT1C4 | chr2:108994+ | protein_codi-0.398775 | 0.7585023 | 0.0072711 | 0.2074702 | 5.0127022 | 5.4114768 | 32.134344 | 32.597112 | 29.22323 | 38.007369 | 43.55455 | 43.362072 |
| ENSG00000198150.2 | AC135178.1 | chr17:82617- | protein_codi-0.300383 | 0.8120368 | 0.027653 | 0.2860611 | 0.3465193 | 0.6469023 | 0.300278 | 0.202022 | 0.315193 | 0.446062 | 0.527068 | 0.738464 |
| ENSG00000198315.10_2 | ZKSCAN8 | chr6:281096+ | protein_codi-0.424453 | 0.7451213 | 0.0169962 | 0.2499474 | 2.1282307 | 2.5526835 | 3.1048039 | 3.7978482 | 3.242725 | 4.3670175 | 5.5369113 | 4.7570138 |
| ENSG00000198535.5_2 | C2CD4A | chr15:62359+ | protein_codi-0.41303 | 0.7510442 | 0.0130922 | 0.2354662 | 2.526184 | 2.9392144 | 5.493797 | 4.713838 | 4.151632 | 6.721369 | 6.573021 | 6.716344 |
| ENSG00000198677.10_2 | TTC37 | chr5:947995- | protein_codi-0.397519 | 0.7591627 | 0.0479739 | 0.3253803 | 3.0485668 | 3.4460857 | 7.2638802 | 7.9536683 | 6.6549987 | 8.6822925 | 11.905122 | 9.3606074 |
| ENSG00000198718.12_3 | TOGARAM1 | chr14:45431+ | protein_codi-0.305931 | 0.8089199 | 0.0297393 | 0.2901068 | 1.9202209 | 2.2261522 | 2.9572178 | 2.9765111 | 2.4454037 | 3.3707717 | 4.0750079 | 3.6176491 |
| ENSG00000198768.10_2 | APCDD1L | chr20:57034- | protein_codi-0.384164 | 0.7662229 | 0.0124711 | 0.2343664 | 1.9612785 | 2.3454425 | 2.5553278 | 2.908706 | 3.2491138 | 4.4072672 | 4.0463055 | 3.8105447 |
| ENSG00000198825.12_3 | INPP5F | chr10:12148+ | protein_codi-0.284335 | 0.82112 | 0.029945 | 0.2902373 | 1.2746808 | 1.5590158 | 1.6041982 | 1.4265391 | 1.2412491 | 1.8963273 | 2.1861528 | 1.7721535 |
| ENSG00000198959.11_2 | TGM2 | chr20:36755- | protein_codi-0.476203 | 0.7188673 | 0.0007924 | 0.1165864 | 3.4220383 | 3.8982409 | 9.2963168 | 9.6640853 | 10.215097 | 14.623991 | 13.270372 | 13.867403 |
| ENSG00000203724.10_2 | C1orf53 | chr1:197871+ | protein_codi0.3114607 | 1.2409635 | 0.049402 | 0.3291951 | 3.153082 | 2.8416214 | 6.7717077 | 9.1336581 | 7.9378717 | 6.0280384 | 6.1789072 | 6.3004115 |
| ENSG00000203727.3_3 | SAMD5 | chr6:147830+ | protein_codi-0.458787 | 0.7275978 | 0.0394536 | 0.3123648 | 3.8610607 | 4.3198477 | 14.701205 | 15.090768 | 11.144385 | 17.352304 | 21.154123 | 18.591426 |
| ENSG00000203827.7 | NBPF16 | chr1:148739+ | protein_codi-0.295297 | 0.8149044 | 0.0286093 | 0.2885442 | 2.178744 | 2.4740414 | 3.677442 | 3.667234 | 3.251419 | 4.248225 | 5.169019 | 4.297286 |
| ENSG00000203857.9_2 | HSD3B1 | chr1:120049+ | protein_codi-0.413722 | 0.750684 | 0.0003664 | 0.0943724 | 3.0617992 | 3.4755216 | 7.7721208 | 7.1705216 | 7.1231757 | 10.270688 | 10.003493 | 10.097568 |
| ENSG00000204388.6_3 | HSPA1B | chr6:317955+ | protein_codi0.2876931 | 1.2206868 | 0.0126087 | 0.2343664 | 7.2239013 | 6.9362082 | 154.03835 | 144.05458 | 147.54646 | 132.14427 | 115.55032 | 117.35434 |
| ENSG00000204389.9_3 | HSPA1A | chr6:317832+ | protein_codi0.2681205 | 1.2042379 | 0.0076365 | 0.2074702 | 6.3262068 | 6.0580864 | 82.731467 | 77.922649 | 77.170645 | 69.351566 | 62.288587 | 65.435157 |
| ENSG00000204472.12_3 | AIF1 | chr6:315829+ | protein_codi-0.281247 | 0.8228792 | 0.0171647 | 0.2499474 | 2.1243256 | 2.405573 | 3.4424668 | 3.1862601 | 3.4566324 | 4.1009799 | 4.028799 | 4.7987047 |
| ENSG00000205138.3_2 | SDHAF1 | chr19:36486+ | protein_codi0.287302 | 1.2203559 | 0.0162052 | 0.2484394 | 5.1513533 | 4.8640513 | 35.57579 | 31.609884 | 36.635059 | 29.477341 | 27.120298 | 27.819008 |
| ENSG00000205426.10_2 | KRT81 | chr12:52679- | protein_codi0.5407705 | 1.4547493 | 0.0183991 | 0.2558547 | 1.0358787 | 0.4951082 | 0.890842 | 1.399706 | 0.899671 | 0.500398 | 0.485448 | 0.256213 |
| ENSG00000213015.8_3 | ZNF580 | chr19:56146+ | protein_codi-0.510327 | 0.7020632 | 0.0488334 | 0.327958 | 3.050385 | 3.5607122 | 6.8231653 | 9.3690949 | 6.0088829 | 11.254547 | 9.7149532 | 11.512844 |
| ENSG00000213221.4_3 | DNLZ | chr9:139253- | protein_codi0.2913108 | 1.2237516 | 0.0245741 | 0.274152 | 3.2597125 | 2.9684017 | 8.5519617 | 7.8419652 | 9.40333 | 7.3622198 | 6.5812639 | 6.5625922 |
| ENSG00000213398.7_3 | LCAT | chr16:67973- | protein_codi0.2863763 | 1.2195732 | 0.0018541 | 0.1515851 | 1.1421247 | 0.8557484 | 1.1993416 | 1.2151477 | 1.2067139 | 0.7153756 | 0.8463691 | 0.8712857 |
| ENSG00000213463.4_3 | SYNJ2BP | chr14:70833- | protein_codi-0.393422 | 0.7613215 | 0.009373 | 0.2136778 | 3.2915864 | 3.6850086 | 9.8262729 | 8.1641961 | 8.4629241 | 11.316854 | 12.546425 | 11.751754 |
| ENSG00000213523.10_3 | SRA1 | chr5:139916- | protein_codi0.3731809 | 1.2952054 | 0.0074478 | 0.2074702 | 3.2127888 | 2.8396078 | 7.8845838 | 8.3913993 | 8.5514647 | 6.4588929 | 5.5201196 | 6.5420858 |
| ENSG00000213676.10_3 | ATF6B | chr6:320659- | protein_codi0.3872811 | 1.3079262 | 0.0163662 | 0.2492933 | 3.6405772 | 3.2532961 | 10.67244 | 11.214786 | 12.605713 | 9.3239768 | 7.7001408 | 8.6526333 |
| ENSG00000213977.7_3 | TAX1BP3 | chr17:35661- | protein_codi0.2722486 | 1.2076887 | 0.0031047 | 0.1669411 | 6.5711769 | 6.2989283 | 90.891413 | 94.741032 | 96.721702 | 80.908362 | 74.528686 | 77.896713 |
| ENSG00000215018.9_2 | COL28A1 | chr7:739583- | protein_codi-0.310194 | 0.8065331 | 0.0033772 | 0.1730307 | 0.9416029 | 1.2517972 | 0.9065062 | 0.9429826 | 0.9126918 | 1.2830878 | 1.5471345 | 1.3222612 |
| ENSG00000217930.7_3 | PAM16 | chr16:43815- | protein_codi0.3151293 | 1.2441232 | 0.0415158 | 0.3140442 | 2.9789375 | 2.6638082 | 7.5632555 | 5.9520978 | 7.2317814 | 5.7405976 | 4.9344173 | 5.3618291 |
| ENSG00000221983.7_3 | UBA52 | chr19:18682+ | protein_codi0.272059 | 1.20753 | 0.0470285 | 0.3240376 | 5.9584764 | 5.6864174 | 63.135731 | 59.260576 | 61.21671 | 56.580334 | 45.131819 | 50.412873 |
| ENSG00000224259.6_2 | LINC01133 | chr1:159931+ | lincRNA -0.492847 | 0.7106213 | 4.237E-05 | 0.061109 | 1.5864971 | 2.0793443 | 2.092795 | 1.9747831 | 1.9440367 | 3.1630189 | 3.2170937 | 3.2994544 |
| ENSG00000224877.3_2 | NDUFAF8 | chr17:79213+ | protein_codi0.4515711 | 1.3675287 | 0.0131374 | 0.2354662 | 4.281733 | 3.8301619 | 18.440461 | 16.386967 | 20.770069 | 13.292728 | 14.06619 | 12.361718 |
| ENSG00000225163.4_3 | LINC00618 | chr14:97397+ | sense_overla-0.317698 | 0.802349 | 0.0199387 | 0.2575012 | 0.5291811 | 0.8468793 | 0.396672 | 0.4246904 | 0.5103694 | 0.9313923 | 0.6185387 | 0.8612924 |
| ENSG00000225255.2 | LA16c-83F12.6 | chr22:16199- | lincRNA 0.5354431 | 1.4493872 | 0.003738 | 0.1783088 | 0.5984979 | 0.0630548 | 0.5617073 | 0.5978033 | 0.3911517 | 0.1401031 | 0 | 0 |
| ENSG00000229152.2_3 | ANKRD10-IT1 | chr13:11154- | sense_intron-0.533512 | 0.6908708 | 0.0444264 | 0.3192892 | 1.6063715 | 2.1398837 | 2.316618 | 2.39168 | 1.509503 | 3.081536 | 4.20601 | 3.02883 |
| ENSG00000229955.1_4 | Z98749.1 | chr22:38783- | sense_intron-0.341933 | 0.7889837 | 0.0279174 | 0.2862563 | 0.7852287 | 1.1271613 | 0.654978 | 0.830566 | 0.689486 | 0.946265 | 1.419133 | 1.213433 |
| ENSG00000231196.3 | RP11-495P10.8 | chr1:147762- | lincRNA 0.3036762 | 1.2342856 | 0.0432501 | 0.3166579 | 1.8046343 | 1.5009581 | 2.755129 | 2.615949 | 2.139786 | 1.767267 | 2.099041 | 1.643759 |
| ENSG00000231290.5_3 | APCDD1L-AS1 | chr20:57090+ | processed_tr-0.55444 | 0.6809214 | 0.0005965 | 0.1106916 | 1.7544325 | 2.3088724 | 2.2436112 | 2.5026251 | 2.3805549 | 4.2084907 | 4.0048726 | 3.6667506 |
| ENSG00000231924.9_3 | PSG1 | chr19:43370- | protein_codi-0.531113 | 0.6920208 | 0.0069235 | 0.2047074 | 3.1985389 | 3.7296517 | 8.7717951 | 8.4239238 | 7.401607 | 11.86166 | 11.278347 | 13.783396 |
| ENSG00000232931.5_2 | LINC00342 | chr2:964728- | lincRNA -0.383941 | 0.7663411 | 0.0452176 | 0.3212307 | 1.0094665 | 1.3934079 | 0.8325245 | 1.1552581 | 1.0658082 | 2.0663515 | 1.4957732 | 1.3688942 |
| ENSG00000233895.1_3 | AL121761.1 | chr20:19738+ | lincRNA -0.441794 | 0.7362183 | 0.0034104 | 0.1733337 | 0.3828598 | 0.8246542 | 0.2421627 | 0.4292414 | 0.2487421 | 0.7919261 | 0.81311 | 0.7099864 |
| ENSG00000233967.6_3 | AL359715.2 | chr6:811530+ | lincRNA -0.460938 | 0.726514 | 0.0199495 | 0.2575012 | 0.1734796 | 0.6344172 | 0.1375325 | 0 | 0.2609675 | 0.4575333 | 0.4894615 | 0.723011 |
| ENSG00000234745.10_2 | HLA-B | chr6:312372- | protein_codi0.775915 | 1.7122757 | 0.003506 | 0.1743497 | 4.1260036 | 3.3500886 | 15.025922 | 16.455843 | 18.027805 | 10.697431 | 8.8704546 | 8.1834064 |
| ENSG00000235173.6_2 | HGH1 | chr8:145192+ | protein_codi-0.337006 | 0.7916826 | 0.0287658 | 0.2885442 | 2.055266 | 2.392272 | 2.9163559 | 3.015685 | 3.5650795 | 4.7914731 | 4.1283784 | 3.871557 |
| ENSG00000237903.1_4 | AC004000.1 | chrX:11855+ | sense_intron-0.35023 | 0.7844591 | 0.0093941 | 0.2136778 | 0.6841763 | 1.0344062 | 0.585506 | 0.620474 | 0.614592 | 0.881249 | 1.246455 | 1.033377 |
| ENSG00000239407.5_4 | Z68871.1 | chrX:10213+ | lincRNA -0.270662 | 0.8289392 | 0.0311526 | 0.2933365 | 2.4205228 | 2.6911847 | 4.2512118 | 4.6269711 | 4.1929685 | 4.9933783 | 6.1347137 | 5.2999006 |
| ENSG00000239672.7_3 | NME1 | chr17:49230+ | protein_codi0.3215379 | 1.249662 | 0.023019 | 0.2666812 | 5.4776042 | 5.1560663 | 41.413091 | 45.195428 | 44.151339 | 38.781599 | 31.917331 | 33.61665 |
| ENSG00000240065.7_3 | PSMB9 | chr6:328119+ | protein_codi0.3578422 | 1.2815078 | 0.0093909 | 0.2136778 | 1.2951398 | 0.9372976 | 1.3806445 | 1.3360832 | 1.6573265 | 0.9436081 | 0.7932391 | 1.0147315 |
| ENSG00000241058.3_3 | NSUN6 | chr10:18834- | protein_codi-0.328971 | 0.7961042 | 0.0445196 | 0.3194635 | 1.3362303 | 1.6652011 | 1.7382778 | 1.58181 | 1.2768499 | 2.0198656 | 2.5539006 | 1.9725799 |
| ENSG00000242247.10_3 | ARFGAP3 | chr22:43192- | protein_codi-0.345857 | 0.7868405 | 0.014899 | 0.2432408 | 3.9868458 | 4.3327026 | 15.990106 | 15.088513 | 13.58037 | 17.777318 | 20.36085 | 19.397091 |
| ENSG00000242375.1_4 | AL590705.3 | chr9:999576- | lincRNA -0.321879 | 0.8000273 | 0.0177881 | 0.2515626 | 1.1050953 | 1.4269742 | 1.086736 | 1.318306 | 1.057606 | 1.518521 | 1.644338 | 1.918929 |
| ENSG00000243130.7_3 | PSG11 | chr19:43511- | protein_codi-0.528728 | 0.6931659 | 0.0029098 | 0.162509 | 2.3338618 | 2.8625893 | 3.879565 | 3.9715297 | 4.2822149 | 5.82792 | 7.048612 | 6.0011004 |
| ENSG00000243649.8_3 | CFB | chr6:319134+ | protein_codi-0.366392 | 0.7757199 | 0.0148019 | 0.2432408 | 1.3281331 | 1.6945254 | 1.3707206 | 1.4635621 | 1.7100658 | 2.3772906 | 2.4099235 | 1.9443936 |
| ENSG00000244754.8_3 | N4BP2L2 | chr13:33006- | protein_codi-0.301169 | 0.8115943 | 0.0313635 | 0.2933365 | 1.5428728 | 1.8440421 | 1.8155495 | 2.0251458 | 1.9043137 | 2.4428127 | 2.3212771 | 3.0468536 |
| ENSG00000245532.7_3 | NEAT1 | chr11:65190+ | lincRNA -0.53689 | 0.6892551 | 0.0237786 | 0.2694256 | 2.5019284 | 3.0388185 | 4.4111461 | 4.7453306 | 4.8460452 | 6.0554292 | 8.9773341 | 6.8847617 |
| ENSG00000248751.6_4 | AC004997.1 | chr22:30681- | protein_codi-0.675668 | 0.6260421 | 0.0399021 | 0.3123853 | 0.3571792 | 1.0328477 | 0.089347 | 0.2157735 | 0.5868874 | 0.9408955 | 0.749146 | 1.5230518 |
| ENSG00000251562.7_2 | MALAT1 | chr11:65265+ | lincRNA -0.599214 | 0.6601137 | 0.0250368 | 0.2761127 | 6.889879 | 7.4890927 | 127.96317 | 119.45022 | 106.37606 | 150.93463 | 217.72327 | 173.49092 |
| ENSG00000253161.5_3 | LINC01605 | chr8:372788- | lincRNA -0.378066 | 0.7694684 | 0.0132364 | 0.2354662 | 1.8259653 | 2.2040313 | 2.2980175 | 2.409803 | 2.9630431 | 3.5525005 | 3.8341035 | 3.4450094 |
| ENSG00000253598.1_2 | SLC10A5 | chr8:826058- | protein_codi-0.326328 | 0.797564 | 0.0065227 | 0.2040161 | 0.6919856 | 1.0183134 | 0.626423 | 0.612212 | 0.607939 | 0.895841 | 1.197898 | 0.994432 |
| ENSG00000253746.1_3 | AC091182.2 | chr8:372629- | lincRNA -0.430154 | 0.7421825 | 0.0400502 | 0.3124294 | 0.9233757 | 1.3535297 | 0.674949 | 1.308708 | 0.76409 | 1.561571 | 1.558827 | 1.545727 |
| ENSG00000254577.1_4 | AC087276.2 | chr11:43411+ | sense_overla-0.49869 | 0.7077491 | 0.0102632 | 0.2196553 | 0.1268503 | 0.6255404 | 0.037281 | 0.111459 | 0.129191 | 0.354578 | 0.729728 | 0.567247 |
| ENSG00000254986.7_3 | DPP3 | chr11:66247+ | protein_codi0.2730291 | 1.2083423 | 0.0064195 | 0.2040161 | 4.4510997 | 4.1780706 | 21.231647 | 19.827425 | 21.601454 | 17.979802 | 16.332681 | 17.030854 |
| ENSG00000255198.4_2 | SNHG9 | chr16:20149+ | lincRNA 0.4550059 | 1.3707884 | 0.0230962 | 0.2666812 | 3.0031612 | 2.5481553 | 7.3068588 | 6.2012882 | 7.6154472 | 4.1015478 | 5.3610232 | 5.1657316 |
| ENSG00000256087.6_3 | ZNF432 | chr19:52534- | protein_codi-0.285679 | 0.8203556 | 0.0494207 | 0.3291951 | 1.3537595 | 1.6394381 | 1.7258058 | 1.6288607 | 1.3297225 | 1.8033632 | 2.2988765 | 2.2697514 |
| ENSG00000256806.5_3 | C17orf100 | chr17:65550+ | protein_codi-0.333806 | 0.7934403 | 0.0377844 | 0.3077276 | 1.0009175 | 1.3347239 | 0.8000345 | 1.0014124 | 1.2248523 | 1.4433211 | 1.3842699 | 1.7544812 |
| ENSG00000257921.5_4 | AC025165.3 | chr12:58166+ | protein_codi0.3498693 | 1.2744451 | 0.0024792 | 0.1575693 | 1.1028206 | 0.7529514 | 1.2255142 | 1.04967 | 1.1718607 | 0.6604767 | 0.7732754 | 0.625451 |
| ENSG00000258790.1_4 | AL121594.3 | chr14:35591+ | protein_codi-0.560197 | 0.6782097 | 0.0193316 | 0.2575012 | 1.778133 | 2.3383297 | 3.065934 | 2.317469 | 1.99121 | 3.643054 | 4.065319 | 4.499353 |
| ENSG00000258904.1_4 | AL157871.5 | chr14:10073+ | sense_intron-0.373548 | 0.7718821 | 0.0324798 | 0.2958398 | 0.7295444 | 1.1030921 | 0.641824 | 0.757127 | 0.580209 | 1.078934 | 1.475916 | 0.925816 |
| ENSG00000258947.6_3 | TUBB3 | chr16:89987+ | protein_codi0.394794 | 1.314755 | 0.0071146 | 0.2060586 | 3.9935819 | 3.5987879 | 13.975952 | 14.724131 | 16.163377 | 12.027264 | 10.650066 | 10.717845 |
| ENSG00000259129.5_2 | LINC00648 | chr14:48234- | lincRNA -0.439634 | 0.7373214 | 0.0139448 | 0.2388191 | 0.6979465 | 1.137581 | 0.5076137 | 0.7120622 | 0.6538606 | 1.0830584 | 1.4912524 | 1.0521958 |
| ENSG00000259820.1_3 | AC083843.2 | chr8:135804- | lincRNA -0.458406 | 0.7277898 | 0.0267923 | 0.2812065 | 1.0392764 | 1.4976826 | 1.025031 | 0.985744 | 1.158764 | 1.475803 | 2.349602 | 1.715394 |
| ENSG00000260260.1_2 | SNHG19 | chr16:22047- | lincRNA 0.4142064 | 1.3325655 | 0.0047428 | 0.1853643 | 4.3055345 | 3.8913281 | 17.320324 | 18.532499 | 20.606985 | 13.547011 | 13.672894 | 14.308404 |
| ENSG00000260269.5_4 | AC105036.3 | chr15:75819- | processed_tr-0.296839 | 0.8140343 | 0.0050385 | 0.1896292 | 0.6355477 | 0.9323862 | 0.6180303 | 0.4977647 | 0.5471289 | 1.019555 | 0.8315706 | 0.8790975 |
| ENSG00000260604.2_3 | AL590004.4 | chr6:390515- | lincRNA -0.275227 | 0.8263206 | 0.0110333 | 0.225664 | 2.3792264 | 2.654453 | 4.240451 | 4.204537 | 4.163032 | 5.108882 | 5.846069 | 4.967699 |
| ENSG00000260953.1_3 | AC009093.4 | chr16:29273- | lincRNA -0.847725 | 0.5556603 | 0.0008075 | 0.1165864 | 0.0903192 | 0.938044 | 0.10982 | 0 | 0.087211 | 0.966763 | 0.720414 | 1.078515 |
| ENSG00000261474.1_3 | AC026471.4 | chr16:31460- | lincRNA -0.326346 | 0.7975538 | 0.0173482 | 0.2504633 | 0.8596677 | 1.1860139 | 0.813612 | 0.669871 | 0.973013 | 1.225505 | 1.18399 | 1.423249 |
| ENSG00000261716.1_3 | AC239868.2 | chr1:149816- | sense_overla-0.417719 | 0.7486071 | 0.0380875 | 0.3083391 | 1.0554339 | 1.4731533 | 1.147878 | 1.288324 | 0.826521 | 1.664798 | 2.165987 | 1.536393 |
| ENSG00000262172.1_4 | AC116025.2 | chr17:77965- | sense_intron-0.894197 | 0.5380465 | 0.0187841 | 0.2558979 | 0.9972415 | 1.8914387 | 1.478457 | 0.642473 | 0.953976 | 3.400458 | 2.020277 | 2.84233 |
| ENSG00000262979.1_4 | AC124319.1 | chr17:78289+ | sense_intron-0.323064 | 0.7993705 | 0.0488414 | 0.327958 | 0.4105385 | 0.7336023 | 0.160201 | 0.419322 | 0.426064 | 0.530337 | 0.71165 | 0.75512 |
| ENSG00000266338.6_2 | NBPF15 | chr1:148555+ | protein_codi-0.291479 | 0.817064 | 0.0172342 | 0.2500978 | 2.4266351 | 2.7181141 | 4.6041886 | 4.1955109 | 4.3373887 | 5.5610402 | 6.1423441 | 5.0797847 |
| ENSG00000267221.2_4 | C17orf113 | chr17:40190- | protein_codi-0.385996 | 0.7652506 | 0.0269008 | 0.2817275 | 0.8373597 | 1.2233555 | 0.731431 | 1.059766 | 0.599514 | 1.397686 | 1.227164 | 1.383722 |
| ENSG00000267645.5_3 | POLR2J2 | chr7:102277- | protein_codi-0.288875 | 0.8185403 | 0.0278478 | 0.2862563 | 3.7467371 | 4.0356117 | 11.96188 | 11.510707 | 13.917325 | 15.834345 | 14.59828 | 15.797614 |
| ENSG00000267868.1_3 | AL356740.1 | chr13:11361- | lincRNA -0.280556 | 0.8232739 | 0.0402009 | 0.3124294 | 0.4683109 | 0.7488664 | 0.382081 | 0.25915 | 0.521654 | 0.569584 | 0.763633 | 0.714356 |
| ENSG00000268201.1_4 | AC020915.3 | chr19:58778+ | sense_intron-0.380562 | 0.7681383 | 0.0251292 | 0.2762939 | 1.0625013 | 1.4430633 | 1.408844 | 1.010689 | 0.880965 | 1.783777 | 1.637651 | 1.737564 |
| ENSG00000268702.1 | AL049829.1 | chr14:23790- | protein_codi0.614611 | 1.5311451 | 0.0375406 | 0.3077276 | 3.3200016 | 2.7053905 | 8.234838 | 7.79789 | 11.258924 | 6.508056 | 5.753335 | 4.472242 |
| ENSG00000268798.1_4 | AC027307.3 | chr19:14408+ | lincRNA 0.2679086 | 1.2040611 | 0.0301714 | 0.2902479 | 0.9010874 | 0.6331788 | 0.737691 | 0.851844 | 1.023886 | 0.632971 | 0.450587 | 0.575053 |
| ENSG00000269113.3_3 | TRABD2B | chr1:482262- | protein_codi0.2742603 | 1.2093739 | 0.0003168 | 0.0943724 | 1.1007688 | 0.8265084 | 1.174857 | 1.128217 | 1.1313101 | 0.825763 | 0.739031 | 0.756547 |
| ENSG00000269242.1_4 | AC010422.3 | chr19:12754- | protein_codi0.3101544 | 1.2398404 | 0.0399673 | 0.3123853 | 0.7574867 | 0.4473322 | 0.69054 | 0.644695 | 0.737672 | 0.559658 | 0.318071 | 0.233142 |
| ENSG00000269974.1_4 | AC091057.3 | chr15:30941+ | lincRNA -0.543748 | 0.6859865 | 0.0069614 | 0.2052944 | 0.5353075 | 1.0790554 | 0.527994 | 0.334899 | 0.492316 | 0.876728 | 1.185792 | 1.298656 |
| ENSG00000270072.1_4 | AC090559.2 | chr11:47535- | sense_intron-0.301686 | 0.8113039 | 0.0149625 | 0.2432408 | 0.3456842 | 0.6473699 | 0.152931 | 0.350005 | 0.318394 | 0.611123 | 0.543672 | 0.545075 |
| ENSG00000270190.1_4 | AC068491.3 | chr2:112024- | lincRNA -0.680943 | 0.6237575 | 0.0047057 | 0.1853643 | 0.3403926 | 1.0213354 | 0.20333 | 0.396138 | 0.20807 | 0.848573 | 1.311133 | 0.957475 |
| ENSG00000271992.1_3 | AL354872.2 | chr1:709107+ | lincRNA -0.595062 | 0.6620159 | 0.0288553 | 0.2885442 | 0.4090951 | 1.0041574 | 0.155278 | 0.322832 | 0.531999 | 1.414927 | 0.827791 | 0.828159 |
| ENSG00000272732.1_3 | AC004982.1 | chr7:758973- | lincRNA -0.452071 | 0.7309926 | 0.0385051 | 0.3090535 | 1.2059952 | 1.6580665 | 1.359995 | 1.386123 | 1.18031 | 1.651576 | 2.738271 | 2.171089 |
| ENSG00000272791.1_3 | AC073389.3 | chr10:75390+ | lincRNA -0.370981 | 0.7732565 | 0.0469802 | 0.3240376 | 0.2098696 | 0.5808506 | 0.254944 | 0.135169 | 0.086041 | 0.277459 | 0.654627 | 0.583118 |
| ENSG00000273035.1_4 | AC007684.1 | chr2:395504- | sense_intron-0.463847 | 0.7250503 | 0.0072836 | 0.2074702 | 0.3991262 | 0.8629732 | 0.437754 | 0.248616 | 0.277419 | 0.657385 | 0.90713 | 0.903438 |
| ENSG00000273084.1_3 | AC092171.5 | chr7:546836+ | lincRNA -0.33554 | 0.7924876 | 0.0135266 | 0.2376832 | 3.0639175 | 3.3994571 | 7.48219 | 6.73951 | 7.907826 | 10.342749 | 9.291288 | 9.065321 |
| ENSG00000273243.1_4 | Z82243.1 | chr22:45551- | lincRNA -0.296196 | 0.814397 | 0.018755 | 0.2558547 | 1.9035681 | 2.199764 | 2.716484 | 3.034766 | 2.49255 | 3.54184 | 3.894451 | 3.361632 |
| ENSG00000273331.1_3 | TM4SF19-TCTEXchr3:196042- | | protein_codi-0.468396 | 0.7227678 | 0.0362284 | 0.304814 | 0.2325374 | 0.7009332 | 0.069414 | 0.118327 | 0.356087 | 0.417612 | 0.837554 | 0.648953 |
| ENSG00000273344.1_2 | PAXIP1-AS1 | chr7:154795+ | lincRNA -0.306466 | 0.8086198 | 0.0469657 | 0.3240376 | 1.782077 | 2.0885434 | 2.104177 | 2.425316 | 2.825857 | 3.642503 | 3.115621 | 3.026754 |
| ENSG00000273437.1_3 | AC108673.3 | chr3:128882- | lincRNA 0.3425641 | 1.2680082 | 0.0229797 | 0.2666812 | 0.8956579 | 0.5530939 | 0.939284 | 1.014461 | 0.648388 | 0.511417 | 0.494891 | 0.397976 |
| ENSG00000273723.1_3 | AL139089.1 | chr13:53225- | lincRNA 0.28044 | 1.2145652 | 0.0452694 | 0.3212988 | 0.6231883 | 0.3427483 | 0.693591 | 0.433352 | 0.505337 | 0.184646 | 0.384529 | 0.243489 |
| ENSG00000274373.1_3 | AC148476.1 | chr12:13300- | lincRNA 0.4379495 | 1.3546775 | 0.0053549 | 0.1925603 | 0.8516511 | 0.4137017 | 0.798813 | 0.682221 | 0.941997 | 0.238568 | 0.388601 | 0.374398 |
| ENSG00000274979.1_3 | AC020656.2 | chr12:69720- | lincRNA -0.272707 | 0.8277652 | 0.0200016 | 0.2575012 | 0.5239407 | 0.7966473 | 0.364533 | 0.554289 | 0.401683 | 0.748258 | 0.826922 | 0.641038 |
| ENSG00000275126.1_2 | HIST1H4L | chr6:278409- | protein_codi0.2719177 | 1.2074117 | 0.0370136 | 0.3060351 | 8.8906619 | 8.6187442 | 454.6731 | 446.60123 | 523.23151 | 408.65326 | 365.34723 | 403.75504 |
| ENSG00000275784.1_4 | AL034549.1 | chr20:32577- | lincRNA 0.2848803 | 1.2183092 | 0.0153902 | 0.2452764 | 1.2163981 | 0.9315178 | 1.264055 | 1.422432 | 1.287591 | 1.065146 | 0.774802 | 0.89297 |
| ENSG00000276023.4_2 | DUSP14 | chr17:35849+ | protein_codi0.27346 | 1.2087032 | 0.0011643 | 0.1332171 | 4.1969525 | 3.9234925 | 17.812966 | 17.049762 | 17.167577 | 14.71487 | 14.098004 | 13.724362 |
| ENSG00000276334.1_3 | AL133243.2 | chr2:327469+ | sense_intron-0.4493 | 0.7323983 | 0.0277676 | 0.2860796 | 0.6528121 | 1.1021117 | 0.782964 | 0.546702 | 0.409285 | 1.005028 | 1.428143 | 1.031942 |
| ENSG00000276418.5_4 | AC036214.3 | chr8:808310- | protein_codi-0.802693 | 0.5732779 | 0.0053306 | 0.1922172 | 0.1140016 | 0.916695 | 0.097949 | 0.046971 | 0.102648 | 1.102916 | 1.068578 | 0.546551 |
| ENSG00000276529.1_3 | AP001505.2 | chr21:46398+ | lincRNA 0.3047123 | 1.2351723 | 0.0082254 | 0.210149 | 2.1795777 | 1.8748653 | 3.270992 | 3.607791 | 3.724254 | 2.620696 | 2.502441 | 2.890534 |
| ENSG00000276564.1_4 | AC130650.2 | chr16:14244+ | lincRNA -0.28131 | 0.8228436 | 0.0291277 | 0.2885442 | 0.3167829 | 0.5980928 | 0.282624 | 0.263392 | 0.192466 | 0.404736 | 0.681907 | 0.46803 |
| ENSG00000276931.1_4 | AC009041.4 | chr16:10183- | sense_intron-0.347301 | 0.7860532 | 0.0410522 | 0.3138435 | 1.702463 | 2.049764 | 1.969551 | 2.512266 | 2.30521 | 3.060577 | 2.744128 | 3.66856 |
| ENSG00000277363.4_4 | SRCIN1 | chr17:36686- | protein_codi-0.287462 | 0.8193424 | 0.004774 | 0.1857934 | 1.3815707 | 1.6690323 | 1.5379834 | 1.5210974 | 1.7644215 | 2.3009836 | 2.1353547 | 2.1071077 |
| ENSG00000278107.1_4 | AC027575.4 | chr18:74517- | lincRNA -0.344498 | 0.7875822 | 0.0464602 | 0.3240376 | 0.2615842 | 0.6060817 | 0.27836 | 0.086731 | 0.240106 | 0.737146 | 0.392384 | 0.457978 |
| ENSG00000278978.1_4 | AC092611.3 | chr4:152590+ | TEC -0.339043 | 0.7905655 | 0.0365438 | 0.3055634 | 1.9745789 | 2.3136221 | 2.691092 | 3.14336 | 2.969309 | 3.871895 | 4.643465 | 3.468535 |
| ENSG00000279089.1_4 | AC005839.1 | chr17:49260- | TEC -0.31992 | 0.8011143 | 0.0187345 | 0.2558547 | 0.6646734 | 0.9845935 | 0.682639 | 0.619128 | 0.462136 | 0.974162 | 1.123515 | 0.848155 |
| ENSG00000279144.1_4 | AC099667.1 | chr3:528821- | TEC -0.514945 | 0.6998194 | 0.0037098 | 0.1780801 | 0.2651842 | 0.7801297 | 0.134007 | 0.338106 | 0.143876 | 0.658947 | 0.796827 | 0.698981 |
| ENSG00000279313.1_3 | CU634019.4 | GL000195.1+ | lincRNA 0.771559 | 1.7071135 | 0.0438218 | 0.3175232 | 0.8700193 | 0.0984603 | 1.143724 | 1.192489 | 0.298971 | 0 | 0.227209 | 0 |
| ENSG00000279400.1_3 | AC008957.3 | chr5:366678+ | TEC -0.49842 | 0.7078816 | 0.0223721 | 0.2659269 | 1.2880151 | 1.7864352 | 1.516348 | 1.31753 | 1.496879 | 1.975322 | 3.081061 | 2.380685 |
| ENSG00000279500.1_4 | AC108704.2 | chr12:12929- | TEC -0.351351 | 0.7838499 | 0.0411499 | 0.3138435 | 1.1168367 | 1.4681874 | 0.902 | 1.160025 | 1.48276 | 1.909874 | 1.638038 | 1.75899 |
| ENSG00000279591.1_4 | AC002044.2 | chr16:73048- | TEC -0.428056 | 0.7432625 | 0.0460912 | 0.3227656 | 0.7639827 | 1.1920391 | 0.51664 | 0.54946 | 1.083926 | 1.212132 | 1.337187 | 1.306822 |
| ENSG00000279759.1_4 | AC118344.2 | chr19:40861+ | TEC -0.286037 | 0.8201516 | 0.0447338 | 0.3195842 | 1.1386325 | 1.4246699 | 1.021601 | 1.296186 | 1.299244 | 1.434502 | 1.711063 | 1.931267 |
| ENSG00000280042.1_4 | AC022336.3 | chr3:124509- | TEC -0.329092 | 0.7960372 | 0.0003519 | 0.0943724 | 0.3576347 | 0.6867269 | 0.286377 | 0.271779 | 0.285868 | 0.618046 | 0.661161 | 0.551578 |
| ENSG00000280099.1_3 | AL603750.1 | chr1:856181- | TEC -0.309492 | 0.8069259 | 0.0023585 | 0.1571562 | 0.9802326 | 1.2897245 | 0.990239 | 0.861673 | 1.07219 | 1.446424 | 1.447554 | 1.440469 |
| ENSG00000283000.1_3 | AL392046.2 | chr10:35258+ | lincRNA -0.274654 | 0.8266488 | 0.0134406 | 0.2374656 | 0.5735535 | 0.848207 | 0.4465153 | 0.4510408 | 0.5702462 | 0.9218401 | 0.6959653 | 0.7900824 |

**Supplementary Table 2.** Correlation between PAXIP1-AS1 expression and

clinicopathological parameters of GC

| **Parameters** | **Case** | **PAXIP1-AS1 expression** | **p-value** |
| --- | --- | --- | --- |
| **Gender** |  |  |  |
| Male | 54 | 0.587±0.996 | 0.921 |
| Female | 21 | 0.564±0.348 |  |
| **Age(yrs)** |  |  |  |
| <60 | 41 | 0.614±0.886 | 0.718 |
| ≥60 | 34 | 0.541±0.844 |  |
| **Tumor size(cm)** |  |  |  |
| <5 | 43 | 0.527±0.447 | 0.537 |
| ≥5 | 32 | 0.652±1.222 |  |
| **Differentiation** |  |  |  |
| Well/Moderate | 24 | 0.391±0.395 | 0.192 |
| Poor | 51 | 0.669±1.001 |  |
| **Tumor invasion(T)** |  |  |  |
| T1-T2 | 12 | 1.074±1.239 | **0.029** |
| T3-T4 | 63 | 0.486±0.747 |  |
| **Lymph node(N)** |  |  |  |
| Absent | 17 | 0.980±1.254 | **0.029** |
| Present | 58 | 0.463±0.679 |  |
| **AJCC stage** |  |  |  |
| I-II | 25 | 0.960±1.328 | **0.046** |
| III-IV | 50 | 0.391±0.384 |  |

**Supplementary Table 3.** Mass spectra obtained for PAXIP1-AS1 pulldown proteins

| **Gene Name** | **UniProtKB** | **Score** | **Mass** | **Matches** | **emPAI** | **Description** |
| --- | --- | --- | --- | --- | --- | --- |
| HNRNPH2 | P55795 | 487 | 49517 | 14 | 0.91 | Heterogeneous nuclear ribonucleoprotein H2 |
| RPS4X | P62701 | 238 | 29807 | 9 | 1.09 | 40S ribosomal protein S4, X isoform |
| PABPC1 | P11940 | 153 | 70854 | 9 | 0.44 | Polyadenylate-binding protein 1 |
| DHX30 | Q7L2E3 | 225 | 134938 | 8 | 0.21 | ATP-dependent RNA helicase DHX30 |
| HARS2 | P49590 | 127 | 57593 | 6 | 0.4 | Probable histidine--tRNA ligase, mitochondrial |
| PCBP1 | Q15365 | 240 | 37987 | 5 | 0.4 | Poly(rC)-binding protein 1 |
| ABCF1 | Q8NE71 | 213 | 96323 | 5 | 0.18 | ATP-binding cassette sub-family F member 1 |
| EPRS | P07814 | 130 | 172080 | 5 | 0.1 | Bifunctional glutamate/proline--tRNA ligase |
| EEF2 | P13639 | 123 | 96246 | 5 | 0.18 | Elongation factor 2 |
| PLS3 | P13797 | 112 | 71279 | 5 | 0.25 | Plastin-3 |
| PARP1 | P09874 | 112 | 113811 | 5 | 0.15 | Poly [ADP-ribose] polymerase 1 |
| PABPC4 | Q13310 | 83 | 71080 | 5 | 0.25 | Polyadenylate-binding protein 4 |
| RPL26 | P61254 | 79 | 17248 | 5 | 1.45 | 60S ribosomal protein L26 |
| FXR2 | P51116 | 200 | 74520 | 4 | 0.19 | Fragile X mental retardation syndrome-related protein 2 |
| EIF3C | Q99613 | 179 | 105962 | 4 | 0.13 | Eukaryotic translation initiation factor 3 subunit C |
| RFC1 | P35251 | 142 | 128688 | 4 | 0.11 | Replication factor C subunit 1 |
| HNRNPM | P52272 | 134 | 77749 | 4 | 0.13 | Heterogeneous nuclear ribonucleoprotein M |
| ADAR | P55265 | 99 | 137178 | 4 | 0.1 | Double-stranded RNA-specific adenosine deaminase |
| RPL13 | P26373 | 93 | 24304 | 4 | 0.67 | 60S ribosomal protein L13 |
| RPS7 | P62081 | 90 | 22113 | 4 | 0.33 | 40S ribosomal protein S7 |
| ACLY | P53396 | 84 | 121674 | 4 | 0.11 | ATP-citrate synthase |
| VCP | P55072 | 74 | 89950 | 4 | 0.15 | Transitional endoplasmic reticulum ATPase |
| CTNNB1 | P35222 | 74 | 86069 | 4 | 0.16 | Catenin beta-1 |
| TOP2A | P11388 | 68 | 175017 | 4 | 0.08 | DNA topoisomerase 2-alpha |
| MAP7 | Q14244 | 152 | 84116 | 3 | 0.08 | Ensconsin |
| ESRP1 | Q6NXG1 | 129 | 76449 | 3 | 0.13 | Epithelial splicing regulatory protein 1 |
| NSFL1C | Q9UNZ2 | 121 | 40548 | 3 | 0.27 | NSFL1 cofactor p47 |
| HARS | P12081 | 103 | 57944 | 3 | 0.18 | Histidine--tRNA ligase, cytoplasmic |
| DRG1 | Q9Y295 | 89 | 40802 | 3 | 0.26 | Developmentally-regulated GTP-binding protein 1 |
| RFC5 | P40937 | 85 | 38757 | 3 | 0.28 | Replication factor C subunit 5 |
| PAWR | Q96IZ0 | 80 | 36659 | 3 | 0.3 | PRKC apoptosis WT1 regulator protein |
| RAN | P62826 | 79 | 24579 | 3 | 0.47 | GTP-binding nuclear protein Ran |
| RPS24 | P62847 | 77 | 15413 | 3 | 0.82 | 40S ribosomal protein S24 |
| YWHAH | Q04917 | 76 | 28372 | 3 | 0.39 | 14-3-3 protein eta |
| SF3B3 | Q15393 | 72 | 136575 | 3 | 0.07 | Splicing factor 3B subunit 3 |
| RPS27 | P42677 | 69 | 9797 | 3 | 0.84 | 40S ribosomal protein S27 |
| TOP1 | P11387 | 62 | 91125 | 3 | 0.07 | DNA topoisomerase 1 |
| RPS23 | P62266 | 59 | 15969 | 3 | 0.47 | 40S ribosomal protein S23 |
| U2SURP | O15042 | 57 | 118675 | 3 | 0.08 | U2 snRNP-associated SURP motif-containing protein |
| ASCC3 | Q8N3C0 | 52 | 252898 | 3 | 0.04 | Activating signal cointegrator 1 complex subunit 3 |
| RPL24 | P83731 | 117 | 17882 | 2 | 0.41 | 60S ribosomal protein L24 |
| EIF4G1 | Q04637 | 109 | 176124 | 2 | 0.04 | Eukaryotic translation initiation factor 4 gamma 1 |
| SMARCA5 | O60264 | 107 | 122513 | 2 | 0.05 | SWI/SNF-related matrix-associated actin-dependent regulator of chromatin subfamily A member 5 |
| SARNP | P82979 | 104 | 23713 | 2 | 0.3 | SAP domain-containing ribonucleoprotein |
| PNKP | Q96T60 | 97 | 57554 | 2 | 0.12 | Bifunctional polynucleotide phosphatase/kinase |
| YWHAQ | P27348 | 97 | 28032 | 2 | 0.25 | 14-3-3 protein theta |
| PHAX | Q9H814 | 95 | 44546 | 2 | 0.15 | Phosphorylated adapter RNA export protein |
| DLST | P36957 | 95 | 49067 | 2 | 0.14 | Dihydrolipoyllysine-residue succinyltransferase component of 2-oxoglutarate dehydrogenase complex, mitochondrial |
| PGAM5 | Q96HS1 | 93 | 32213 | 2 | 0.22 | Serine/threonine-protein phosphatase PGAM5, mitochondrial |
| FMR1 | Q06787 | 92 | 71473 | 2 | 0.09 | Synaptic functional regulator FMR1 |
| CHTOP | Q9Y3Y2 | 87 | 26380 | 2 | 0.27 | Chromatin target of PRMT1 protein |
| L1RE1 | Q9UN81 | 86 | 40259 | 2 | 0.17 | LINE-1 retrotransposable element ORF1 protein |
| RPL27A | P46776 | 86 | 16665 | 2 | 0.45 | 60S ribosomal protein L27a |
| FARSA | Q9Y285 | 82 | 57585 | 2 | 0.12 | Phenylalanine--tRNA ligase alpha subunit |
| THRAP3 | Q9Y2W1 | 78 | 108658 | 2 | 0.06 | Thyroid hormone receptor-associated protein 3 |
| ESRP2 | Q9H6T0 | 72 | 79206 | 2 | 0.08 | Epithelial splicing regulatory protein 2 |
| SLC3A2 | P08195 | 72 | 68180 | 2 | 0.1 | 4F2 cell-surface antigen heavy chain |
| IFIT1 | P09914 | 70 | 55781 | 2 | 0.12 | Interferon-induced protein with tetratricopeptide repeats 1 |
| FKBP3 | Q00688 | 69 | 25218 | 2 | 0.28 | Peptidyl-prolyl cis-trans isomerase FKBP3 |
| PNO1 | Q9NRX1 | 68 | 28078 | 2 | 0.25 | RNA-binding protein PNO1 |
| CPSF6 | Q16630 | 68 | 59344 | 2 | 0.11 | Cleavage and polyadenylation specificity factor subunit 6 |
| ECH1 | Q13011 | 67 | 36136 | 2 | 0.19 | Delta(3,5)-Delta(2,4)-dienoyl-CoA isomerase, mitochondrial |
| CDC37 | Q16543 | 64 | 44953 | 2 | 0.15 | Hsp90 co-chaperone Cdc37 |
| EIF4B | P23588 | 64 | 69167 | 2 | 0.1 | Eukaryotic translation initiation factor 4B |
| SNRPD2 | P62316 | 63 | 13632 | 2 | 0.56 | Small nuclear ribonucleoprotein Sm D2 |
| DDX54 | Q8TDD1 | 61 | 98819 | 2 | 0.07 | ATP-dependent RNA helicase DDX54 |
| SARS | P49591 | 60 | 59253 | 2 | 0.11 | Serine--tRNA ligase, cytoplasmic |
| RPL5 | P46777 | 60 | 34569 | 2 | 0.2 | 60S ribosomal protein L5 |
| FBL | P22087 | 59 | 33877 | 2 | 0.21 | rRNA 2~-O-methyltransferase fibrillarin |
| RPL4 | P36578 | 59 | 47953 | 2 | 0.14 | 60S ribosomal protein L4 |
| EIF3G | O75821 | 56 | 35874 | 2 | 0.19 | Eukaryotic translation initiation factor 3 subunit G |
| ETF1 | P62495 | 56 | 49228 | 2 | 0.14 | Eukaryotic peptide chain release factor subunit 1 |
| G3BP1 | Q13283 | 55 | 52189 | 2 | 0.13 | Ras GTPase-activating protein-binding protein 1 |
| RPL10L | Q96L21 | 53 | 24959 | 2 | 0.29 | 60S ribosomal protein L10-like |
| SMC1A | Q14683 | 53 | 143771 | 2 | 0.05 | Structural maintenance of chromosomes protein 1A |
| HP1BP3 | Q5SSJ5 | 52 | 61454 | 2 | 0.11 | Heterochromatin protein 1-binding protein 3 |
| DNMT1 | P26358 | 51 | 185388 | 2 | 0.04 | DNA (cytosine-5)-methyltransferase 1 |
| PSMA7 | O14818 | 51 | 28041 | 2 | 0.25 | Proteasome subunit alpha type-7 |
| PACSIN2 | Q9UNF0 | 51 | 56046 | 2 | 0.12 | Protein kinase C and casein kinase substrate in neurons protein 2 |
| PSMB6 | P28072 | 49 | 25570 | 2 | 0.28 | Proteasome subunit beta type-6 |
| CCDC124 | Q96CT7 | 49 | 25820 | 2 | 0.27 | Coiled-coil domain-containing protein 124 |
| NOP16 | Q9Y3C1 | 49 | 21232 | 2 | 0.34 | Nucleolar protein 16 |
| PRPF19 | Q9UMS4 | 48 | 55603 | 2 | 0.12 | Pre-mRNA-processing factor 19 |
| BCLAF1 | Q9NYF8 | 46 | 106173 | 2 | 0.06 | Bcl-2-associated transcription factor 1 |
| PRDX2 | P32119 | 46 | 22049 | 2 | 0.33 | Peroxiredoxin-2 |
| DYNC1H1 | Q14204 | 46 | 534809 | 2 | 0.01 | Cytoplasmic dynein 1 heavy chain 1 |
| NEMF | O60524 | 45 | 123334 | 2 | 0.05 | Nuclear export mediator factor NEMF |
| RPS26P11 | Q5JNZ5 | 45 | 13336 | 2 | 0.58 | Putative 40S ribosomal protein S26-like 1 |
| TPR | P12270 | 41 | 267530 | 2 | 0.02 | Nucleoprotein TPR |
| GOT2 | P00505 | 41 | 47886 | 2 | 0.14 | Aspartate aminotransferase, mitochondrial |
| BTF3 | P20290 | 40 | 22211 | 2 | 0.32 | Transcription factor BTF3 |
| LASP1 | Q14847 | 35 | 30097 | 2 | 0.23 | LIM and SH3 domain protein 1 |
| SRBD1 | Q8N5C6 | 34 | 112960 | 2 | 0.06 | S1 RNA-binding domain-containing protein 1 |
| CACYBP | Q9HB71 | 34 | 26308 | 2 | 0.27 | Calcyclin-binding protein |
| RPS17 | P08708 | 31 | 15597 | 2 | 0.48 | 40S ribosomal protein S17 |
| QARS | P47897 | 29 | 88655 | 2 | 0.08 | Glutamine--tRNA ligase |
| ERH | P84090 | 26 | 12422 | 2 | 0.63 | Enhancer of rudimentary homolog |
| CTNNA2 | P26232 | 102 | 106045 | 1 | 0.03 | Catenin alpha-2 |
| GLYR1 | Q49A26 | 96 | 61079 | 1 | 0.05 | Putative oxidoreductase GLYR1 |
| SARS2 | Q9NP81 | 95 | 58702 | 1 | 0.06 | Serine--tRNA ligase, mitochondrial |
| GEMIN5 | Q8TEQ6 | 84 | 170821 | 1 | 0.02 | Gem-associated protein 5 |
| CALD1 | Q05682 | 80 | 93232 | 1 | 0.04 | Caldesmon |
| RPLP2 | P05387 | 76 | 11658 | 1 | 0.3 | 60S acidic ribosomal protein P2 |
| BOD1L1 | Q8NFC6 | 74 | 332433 | 1 | 0.01 | Biorientation of chromosomes in cell division protein 1-like 1 |
| RBM4 | Q9BWF3 | 72 | 40688 | 1 | 0.08 | RNA-binding protein 4 |
| EIF1AY | O14602 | 68 | 16546 | 1 | 0.2 | Eukaryotic translation initiation factor 1A, Y-chromosomal |
| BAX | Q07812 | 66 | 21285 | 1 | 0.16 | Apoptosis regulator BAX |
| PRMT1 | Q99873 | 64 | 43061 | 1 | 0.08 | Protein arginine N-methyltransferase 1 |
| RBM17 | Q96I25 | 64 | 45162 | 1 | 0.07 | Splicing factor 45 |
| AHNAK2 | Q8IVF2 | 63 | 617383 | 1 | 0.01 | Protein AHNAK2 |
| NOL6 | Q9H6R4 | 63 | 128368 | 1 | 0.03 | Nucleolar protein 6 |
| RFC2 | P35250 | 63 | 39588 | 1 | 0.08 | Replication factor C subunit 2 |
| EIF3L | Q9Y262 | 60 | 66912 | 1 | 0.05 | Eukaryotic translation initiation factor 3 subunit L |
| ESF1 | Q9H501 | 60 | 99134 | 1 | 0.03 | ESF1 homolog |
| EIF3I | Q13347 | 60 | 36878 | 1 | 0.09 | Eukaryotic translation initiation factor 3 subunit I |
| USP5 | P45974 | 59 | 96638 | 1 | 0.03 | Ubiquitin carboxyl-terminal hydrolase 5 |
| SMARCE1 | Q969G3 | 59 | 46678 | 1 | 0.07 | SWI/SNF-related matrix-associated actin-dependent regulator of chromatin subfamily E member 1 |
| MANBA | O00462 | 58 | 101800 | 1 | 0.03 | Beta-mannosidase |
| WARS | P23381 | 58 | 53474 | 1 | 0.06 | Tryptophan--tRNA ligase, cytoplasmic |
| DARS | P14868 | 57 | 57499 | 1 | 0.06 | Aspartate--tRNA ligase, cytoplasmic |
| DNAJC9 | Q8WXX5 | 55 | 30062 | 1 | 0.11 | DnaJ homolog subfamily C member 9 |
| SPIN1 | Q9Y657 | 55 | 29696 | 1 | 0.11 | Spindlin-1 |
| ZC3HAV1 | Q7Z2W4 | 54 | 103135 | 1 | 0.03 | Zinc finger CCCH-type antiviral protein 1 |
| APEH | P13798 | 54 | 82142 | 1 | 0.04 | Acylamino-acid-releasing enzyme |
| RBM25 | P49756 | 52 | 100467 | 1 | 0.03 | RNA-binding protein 25 |
| HSPH1 | Q92598 | 52 | 97716 | 1 | 0.03 | Heat shock protein 105 kDa |
| C1QBP | Q07021 | 50 | 31742 | 1 | 0.1 | Complement component 1 Q subcomponent-binding protein, mitochondrial |
| FASN | P49327 | 50 | 275877 | 1 | 0.01 | Fatty acid synthase |
| NMT1 | P30419 | 50 | 57112 | 1 | 0.06 | Glycylpeptide N-tetradecanoyltransferase 1 |
| DDX6 | P26196 | 50 | 54781 | 1 | 0.06 | Probable ATP-dependent RNA helicase DDX6 |
| DBNL | Q9UJU6 | 50 | 48463 | 1 | 0.07 | Drebrin-like protein |
| LIG3 | P49916 | 49 | 114317 | 1 | 0.03 | DNA ligase 3 |
| PRSS3 | P35030 | 49 | 33306 | 1 | 0.1 | Trypsin-3 |
| LAMP1 | P11279 | 49 | 45367 | 1 | 0.07 | Lysosome-associated membrane glycoprotein 1 |
| LYAR | Q9NX58 | 49 | 44044 | 1 | 0.07 | Cell growth-regulating nucleolar protein |
| HNRNPUL2 | Q1KMD3 | 48 | 85622 | 1 | 0.04 | Heterogeneous nuclear ribonucleoprotein U-like protein 2 |
| GSS | P48637 | 48 | 52523 | 1 | 0.06 | Glutathione synthetase |
| PSME3 | P61289 | 48 | 29602 | 1 | 0.11 | Proteasome activator complex subunit 3 |
| IARS2 | Q9NSE4 | 47 | 114688 | 1 | 0.03 | Isoleucine--tRNA ligase, mitochondrial |
| S100A13 | Q99584 | 47 | 11464 | 1 | 0.3 | Protein S100-A13 |
| PPP1R13L | Q8WUF5 | 47 | 89378 | 1 | 0.04 | RelA-associated inhibitor |
| PFDN4 | Q9NQP4 | 47 | 15419 | 1 | 0.22 | Prefoldin subunit 4 |
| RPSA | P08865 | 46 | 32947 | 1 | 0.1 | 40S ribosomal protein SA |
| RBM8A | Q9Y5S9 | 46 | 19934 | 1 | 0.17 | RNA-binding protein 8A |
| RPL15 | P61313 | 46 | 24245 | 1 | 0.14 | 60S ribosomal protein L15 |
| PARVA | Q9NVD7 | 46 | 42274 | 1 | 0.08 | Alpha-parvin |
| IFRD1 | O00458 | 46 | 51035 | 1 | 0.06 | Interferon-related developmental regulator 1 |
| TARS1 | P26639 | 46 | 84294 | 1 | 0.04 | Threonine--tRNA ligase 1, cytoplasmic |
| TATDN1 | Q6P1N9 | 46 | 34150 | 1 | 0.1 | Putative deoxyribonuclease TATDN1 |
| MATR3 | P43243 | 46 | 95078 | 1 | 0.03 | Matrin-3 |
| CORO1B | Q9BR76 | 45 | 54885 | 1 | 0.06 | Coronin-1B |
| SRPK2 | P78362 | 45 | 78219 | 1 | 0.04 | SRSF protein kinase 2 |
| NIPSNAP2 | O75323 | 44 | 33949 | 1 | 0.1 | Protein NipSnap homolog 2 |
| EIF2S2 | P20042 | 44 | 38706 | 1 | 0.09 | Eukaryotic translation initiation factor 2 subunit 2 |
| EIF6 | P56537 | 44 | 27095 | 1 | 0.12 | Eukaryotic translation initiation factor 6 |
| BAG3 | O95817 | 44 | 61728 | 1 | 0.05 | BAG family molecular chaperone regulator 3 |
| RANBP1 | P43487 | 44 | 23467 | 1 | 0.14 | Ran-specific GTPase-activating protein |
| SBNO1 | A3KN83 | 44 | 154900 | 1 | 0.02 | Protein strawberry notch homolog 1 |
| SNRPA1 | P09661 | 43 | 28512 | 1 | 0.12 | U2 small nuclear ribonucleoprotein A~ |
| PKP2 | Q99959 | 43 | 97868 | 1 | 0.03 | Plakophilin-2 |
| LRRC47 | Q8N1G4 | 43 | 64004 | 1 | 0.05 | Leucine-rich repeat-containing protein 47 |
| RAB2A | P61019 | 43 | 23702 | 1 | 0.14 | Ras-related protein Rab-2A |
| ZNFX1 | Q9P2E3 | 43 | 225102 | 1 | 0.01 | NFX1-type zinc finger-containing protein 1 |
| MTREX | P42285 | 43 | 118756 | 1 | 0.03 | Exosome RNA helicase MTR4 |
| ZC3H4 | Q9UPT8 | 42 | 140797 | 1 | 0.02 | Zinc finger CCCH domain-containing protein 4 |
| FAM111A | Q96PZ2 | 42 | 71007 | 1 | 0.05 | Protein FAM111A |
| MTDH | Q86UE4 | 42 | 63856 | 1 | 0.05 | Protein LYRIC |
| RBM10 | P98175 | 42 | 103811 | 1 | 0.03 | RNA-binding protein 10 |
| IBA57 | Q5T440 | 42 | 38473 | 1 | 0.09 | Putative transferase CAF17, mitochondrial |
| SARG | Q9BW04 | 41 | 64211 | 1 | 0.05 | Specifically androgen-regulated gene protein |
| SF3A1 | Q15459 | 41 | 88888 | 1 | 0.04 | Splicing factor 3A subunit 1 |
| LSM8 | O95777 | 41 | 10396 | 1 | 0.33 | U6 snRNA-associated Sm-like protein LSm8 |
| VARS | P26640 | 40 | 141642 | 1 | 0.02 | Valine--tRNA ligase |
| RFC3 | P40938 | 40 | 41328 | 1 | 0.08 | Replication factor C subunit 3 |
| UCHL3 | P15374 | 40 | 26337 | 1 | 0.13 | Ubiquitin carboxyl-terminal hydrolase isozyme L3 |
| YARS | P54577 | 40 | 59448 | 1 | 0.06 | Tyrosine--tRNA ligase, cytoplasmic |
| SPTBN1 | Q01082 | 40 | 275237 | 1 | 0.01 | Spectrin beta chain, non-erythrocytic 1 |
| BCAP31 | P51572 | 40 | 28031 | 1 | 0.12 | B-cell receptor-associated protein 31 |
| SMC2 | O95347 | 40 | 136085 | 1 | 0.02 | Structural maintenance of chromosomes protein 2 |
| BUB3 | O43684 | 39 | 37587 | 1 | 0.09 | Mitotic checkpoint protein BUB3 |
| SRSF7 | Q16629 | 39 | 27578 | 1 | 0.12 | Serine/arginine-rich splicing factor 7 |
| ZFP91 | Q96JP5 | 39 | 64261 | 1 | 0.05 | E3 ubiquitin-protein ligase ZFP91 |
| ARL6IP4 | Q66PJ3 | 39 | 45287 | 1 | 0.07 | ADP-ribosylation factor-like protein 6-interacting protein 4 |
| ZNF787 | Q6DD87 | 39 | 41429 | 1 | 0.08 | Zinc finger protein 787 |
| COPA | P53621 | 38 | 139797 | 1 | 0.02 | Coatomer subunit alpha |
| RIOK1 | Q9BRS2 | 38 | 65884 | 1 | 0.05 | Serine/threonine-protein kinase RIO1 |
| DDX1 | Q92499 | 38 | 83349 | 1 | 0.04 | ATP-dependent RNA helicase DDX1 |
| GSR | P00390 | 38 | 56791 | 1 | 0.06 | Glutathione reductase, mitochondrial |
| PRKRA | O75569 | 38 | 34839 | 1 | 0.1 | Interferon-inducible double-stranded RNA-dependent protein kinase activator A |
| ADSS2 | P30520 | 38 | 50465 | 1 | 0.07 | Adenylosuccinate synthetase isozyme 2 |
| ECI1 | P42126 | 38 | 33080 | 1 | 0.1 | Enoyl-CoA delta isomerase 1, mitochondrial |
| DDX41 | Q9UJV9 | 37 | 70477 | 1 | 0.05 | Probable ATP-dependent RNA helicase DDX41 |
| NOLC1 | Q14978 | 37 | 73560 | 1 | 0.04 | Nucleolar and coiled-body phosphoprotein 1 |
| GATAD2B | Q8WXI9 | 36 | 65562 | 1 | 0.05 | Transcriptional repressor p66-beta |
| CCDC12 | Q8WUD4 | 36 | 19226 | 1 | 0.18 | Coiled-coil domain-containing protein 12 |
| INF2 | Q27J81 | 36 | 136851 | 1 | 0.02 | Inverted formin-2 |
| PLP2 | Q04941 | 36 | 17022 | 1 | 0.2 | Proteolipid protein 2 |
| SLC7A5 | Q01650 | 36 | 55659 | 1 | 0.06 | Large neutral amino acids transporter small subunit 1 |
| CDV3 | Q9UKY7 | 36 | 27318 | 1 | 0.12 | Protein CDV3 homolog |
| DKC1 | O60832 | 36 | 58094 | 1 | 0.06 | H/ACA ribonucleoprotein complex subunit DKC1 |
| SEPTIN11 | Q9NVA2 | 36 | 49652 | 1 | 0.07 | Septin-11 |
| TOMM34 | Q15785 | 36 | 34937 | 1 | 0.09 | Mitochondrial import receptor subunit TOM34 |
| NCBP2 | P52298 | 36 | 18161 | 1 | 0.19 | Nuclear cap-binding protein subunit 2 |
| S100A7A | Q86SG5 | 36 | 11412 | 1 | 0.3 | Protein S100-A7A |
| PGLS | O95336 | 36 | 27815 | 1 | 0.12 | 6-phosphogluconolactonase |
| AIFM1 | O95831 | 35 | 67144 | 1 | 0.05 | Apoptosis-inducing factor 1, mitochondrial |
| NAP1L1 | P55209 | 35 | 45631 | 1 | 0.07 | Nucleosome assembly protein 1-like 1 |
| HEXIM1 | O94992 | 35 | 40884 | 1 | 0.08 | Protein HEXIM1 |
| RPS27L | Q71UM5 | 35 | 9813 | 1 | 0.36 | 40S ribosomal protein S27-like |
| ALDH3A1 | P30838 | 35 | 50762 | 1 | 0.06 | Aldehyde dehydrogenase, dimeric NADP-preferring |
| EIF4H | Q15056 | 35 | 27425 | 1 | 0.12 | Eukaryotic translation initiation factor 4H |
| KIF2A | O00139 | 35 | 80589 | 1 | 0.04 | Kinesin-like protein KIF2A |
| BTF3L4 | Q96K17 | 34 | 17260 | 1 | 0.2 | Transcription factor BTF3 homolog 4 |
| RBBP7 | Q16576 | 34 | 48132 | 1 | 0.07 | Histone-binding protein RBBP7 |
| PRPF6 | O94906 | 34 | 107656 | 1 | 0.03 | Pre-mRNA-processing factor 6 |
| PSMA4 | P25789 | 34 | 29750 | 1 | 0.11 | Proteasome subunit alpha type-4 |
| PEBP4 | Q96S96 | 34 | 26002 | 1 | 0.13 | Phosphatidylethanolamine-binding protein 4 |
| NUDT21 | O43809 | 34 | 26268 | 1 | 0.13 | Cleavage and polyadenylation specificity factor subunit 5 |
| XRCC1 | P18887 | 34 | 69776 | 1 | 0.05 | DNA repair protein XRCC1 |
| RBM42 | Q9BTD8 | 33 | 50496 | 1 | 0.07 | RNA-binding protein 42 |
| DYNC1LI2 | O43237 | 33 | 54351 | 1 | 0.06 | Cytoplasmic dynein 1 light intermediate chain 2 |
| LSM3 | P62310 | 33 | 11838 | 1 | 0.29 | U6 snRNA-associated Sm-like protein LSm3 |
| UBR5 | O95071 | 33 | 312352 | 1 | 0.01 | E3 ubiquitin-protein ligase UBR5 |
| CARS | P49589 | 33 | 86103 | 1 | 0.04 | Cysteine--tRNA ligase, cytoplasmic |
| CHAF1B | Q13112 | 32 | 61910 | 1 | 0.05 | Chromatin assembly factor 1 subunit B |
| ZC3H15 | Q8WU90 | 32 | 48972 | 1 | 0.07 | Zinc finger CCCH domain-containing protein 15 |
| EIF3K | Q9UBQ5 | 32 | 25329 | 1 | 0.13 | Eukaryotic translation initiation factor 3 subunit K |
| MAPRE1 | Q15691 | 32 | 30151 | 1 | 0.11 | Microtubule-associated protein RP/EB family member 1 |
| NAGK | Q9UJ70 | 32 | 37694 | 1 | 0.09 | N-acetyl-D-glucosamine kinase |
| SRSF3 | P84103 | 32 | 19546 | 1 | 0.17 | Serine/arginine-rich splicing factor 3 |
| RPL34 | P49207 | 32 | 13513 | 1 | 0.25 | 60S ribosomal protein L34 |
| PDLIM1 | O00151 | 31 | 36505 | 1 | 0.09 | PDZ and LIM domain protein 1 |
| PLOD3 | O60568 | 31 | 85302 | 1 | 0.04 | Multifunctional procollagen lysine hydroxylase and glycosyltransferase LH3 |
| KNOP1 | Q1ED39 | 31 | 51728 | 1 | 0.06 | Lysine-rich nucleolar protein 1 |
| NOP56 | O00567 | 31 | 66408 | 1 | 0.05 | Nucleolar protein 56 |
| KLF16 | Q9BXK1 | 31 | 25871 | 1 | 0.13 | Krueppel-like factor 16 |
| RFC4 | P35249 | 31 | 40170 | 1 | 0.08 | Replication factor C subunit 4 |
| SDAD1 | Q9NVU7 | 31 | 80277 | 1 | 0.04 | Protein SDA1 homolog |
| XRCC6 | P12956 | 30 | 70084 | 1 | 0.05 | X-ray repair cross-complementing protein 6 |
| DDB1 | Q16531 | 30 | 128142 | 1 | 0.03 | DNA damage-binding protein 1 |
| HNRNPLL | Q8WVV9 | 30 | 60900 | 1 | 0.05 | Heterogeneous nuclear ribonucleoprotein L-like |
| HTATSF1 | O43719 | 30 | 86371 | 1 | 0.04 | HIV Tat-specific factor 1 |
| FUT5 | Q11128 | 30 | 43437 | 1 | 0.08 | Alpha-(1,3)-fucosyltransferase 5 |
| TRA2B | P62995 | 30 | 33760 | 1 | 0.1 | Transformer-2 protein homolog beta |
| EFHD1 | Q9BUP0 | 30 | 27025 | 1 | 0.12 | EF-hand domain-containing protein D1 |
| EIF3J | O75822 | 30 | 29159 | 1 | 0.11 | Eukaryotic translation initiation factor 3 subunit J |
| CD2BP2 | O95400 | 30 | 37737 | 1 | 0.09 | CD2 antigen cytoplasmic tail-binding protein 2 |
| NOP53 | Q9NZM5 | 29 | 54470 | 1 | 0.06 | Ribosome biogenesis protein NOP53 |
| GLO1 | Q04760 | 29 | 20992 | 1 | 0.16 | Lactoylglutathione lyase |
| WDR73 | Q6P4I2 | 29 | 42229 | 1 | 0.08 | WD repeat-containing protein 73 |
| SNRPC | P09234 | 29 | 17552 | 1 | 0.19 | U1 small nuclear ribonucleoprotein C |
| CHD1L | Q86WJ1 | 29 | 101507 | 1 | 0.03 | Chromodomain-helicase-DNA-binding protein 1-like |
| CHMP4B | Q9H444 | 29 | 24935 | 1 | 0.13 | Charged multivesicular body protein 4b |
| EIF4A2 | Q14240 | 29 | 46601 | 1 | 0.07 | Eukaryotic initiation factor 4A-II |
| KPNA2 | P52292 | 28 | 58168 | 1 | 0.06 | Importin subunit alpha-1 |
| PRKDC | P78527 | 28 | 473749 | 1 | 0.01 | DNA-dependent protein kinase catalytic subunit |
| XRCC5 | P13010 | 28 | 83222 | 1 | 0.04 | X-ray repair cross-complementing protein 5 |
| SMNDC1 | O75940 | 28 | 26866 | 1 | 0.12 | Survival of motor neuron-related-splicing factor 30 |
| NARS1 | O43776 | 28 | 63758 | 1 | 0.05 | Asparagine--tRNA ligase, cytoplasmic |
| PAQR4 | Q8N4S7 | 28 | 29677 | 1 | 0.11 | Progestin and adipoQ receptor family member 4 |
| RPL10A | P62906 | 28 | 24987 | 1 | 0.13 | 60S ribosomal protein L10a |
| ANKRD18B | A2A2Z9 | 28 | 119126 | 1 | 0.03 | Ankyrin repeat domain-containing protein 18B |
| PTK2B | Q14289 | 27 | 117112 | 1 | 0.03 | Protein-tyrosine kinase 2-beta |
| CBLN4 | Q9NTU7 | 27 | 21908 | 1 | 0.15 | Cerebellin-4 |
| IGLV8-61 | A0A075B6I0 | 27 | 12920 | 1 | 0.27 | Immunoglobulin lambda variable 8-61 |
| MORF4L2 | Q15014 | 26 | 32345 | 1 | 0.1 | Mortality factor 4-like protein 2 |
| GALE | Q14376 | 26 | 38656 | 1 | 0.09 | UDP-glucose 4-epimerase |
| CHMP4A | Q9BY43 | 26 | 25083 | 1 | 0.13 | Charged multivesicular body protein 4a |
| ELOB | Q15370 | 26 | 13239 | 1 | 0.26 | Elongin-B |
| HDGF | P51858 | 26 | 26886 | 1 | 0.12 | Hepatoma-derived growth factor |
| RPL6 | Q02878 | 26 | 32765 | 1 | 0.1 | 60S ribosomal protein L6 |
| AP3B1 | O00203 | 26 | 121815 | 1 | 0.03 | AP-3 complex subunit beta-1 |
| SIN3A | Q96ST3 | 26 | 145883 | 1 | 0.02 | Paired amphipathic helix protein Sin3a |
| ELANE | P08246 | 26 | 29127 | 1 | 0.11 | Neutrophil elastase |
| RPL9 | P32969 | 26 | 21964 | 1 | 0.15 | 60S ribosomal protein L9 |
| CTNND1 | O60716 | 26 | 108674 | 1 | 0.03 | Catenin delta-1 |
| POP7 | O75817 | 26 | 15755 | 1 | 0.22 | Ribonuclease P protein subunit p20 |
| POLR2A | P24928 | 26 | 218408 | 1 | 0.01 | DNA-directed RNA polymerase II subunit RPB1 |
| EXTL1 | Q92935 | 26 | 75391 | 1 | 0.04 | Exostosin-like 1 |
| SERPINH1 | P50454 | 25 | 46525 | 1 | 0.07 | Serpin H1 |
| GTF2I | P78347 | 25 | 112859 | 1 | 0.03 | General transcription factor II-I |
| SRRM2 | Q9UQ35 | 25 | 300179 | 1 | 0.01 | Serine/arginine repetitive matrix protein 2 |
| CDH5 | P33151 | 25 | 87816 | 1 | 0.04 | Cadherin-5 |
| PARD6B | Q9BYG5 | 25 | 41214 | 1 | 0.08 | Partitioning defective 6 homolog beta |
| DCSTAMP | Q9H295 | 25 | 54099 | 1 | 0.06 | Dendritic cell-specific transmembrane protein |
| RPL27 | P61353 | 25 | 15788 | 1 | 0.22 | 60S ribosomal protein L27 |
| RBMS2 | Q15434 | 25 | 44159 | 1 | 0.07 | RNA-binding motif, single-stranded-interacting protein 2 |
| RRP1B | Q14684 | 25 | 84774 | 1 | 0.04 | Ribosomal RNA processing protein 1 homolog B |
| KYAT3 | Q6YP21 | 25 | 51824 | 1 | 0.06 | Kynurenine--oxoglutarate transaminase 3 |
| HSD17B6 | O14756 | 24 | 36285 | 1 | 0.09 | 17-beta-hydroxysteroid dehydrogenase type 6 |
| TRIM28 | Q13263 | 24 | 90261 | 1 | 0.04 | Transcription intermediary factor 1-beta |
| BTBD10 | Q9BSF8 | 24 | 54202 | 1 | 0.06 | BTB/POZ domain-containing protein 10 |
| SRCAP | Q6ZRS2 | 24 | 344996 | 1 | 0.01 | Helicase SRCAP |
| ATIC | P31939 | 24 | 65089 | 1 | 0.05 | Bifunctional purine biosynthesis protein PURH |
| USP9Y | O00507 | 23 | 294370 | 1 | 0.01 | Probable ubiquitin carboxyl-terminal hydrolase FAF-Y |
| SRRT | Q9BXP5 | 22 | 101060 | 1 | 0.03 | Serrate RNA effector molecule homolog |
| VRK1 | Q99986 | 22 | 45790 | 1 | 0.07 | Serine/threonine-protein kinase VRK1 |
| TFEC | O14948 | 21 | 38935 | 1 | 0.08 | Transcription factor EC |
| ZP2 | Q05996 | 21 | 83501 | 1 | 0.04 | Zona pellucida sperm-binding protein 2 |
| TEX13C | A0A0J9YWL9 | 21 | 110419 | 1 | 0.03 | Putative testis-expressed protein 13C |
| TERF2 | Q15554 | 20 | 59728 | 1 | 0.06 | Telomeric repeat-binding factor 2 |
| PRSS3P2 | Q8NHM4 | 20 | 27090 | 1 | 0.12 | Putative trypsin-6 |
| PRH1 | P02810 | 19 | 17006 | 1 | 0.2 | Salivary acidic proline-rich phosphoprotein 1/2 |
| HMGN3 | Q15651 | 19 | 10660 | 1 | 0.32 | High mobility group nucleosome-binding domain-containing protein 3 |
| ARPC4 | P59998 | 19 | 19768 | 1 | 0.17 | Actin-related protein 2/3 complex subunit 4 |
| DNAJB1 | P25685 | 18 | 38191 | 1 | 0.09 | DnaJ homolog subfamily B member 1 |
| CARMIL1 | Q5VZK9 | 18 | 152717 | 1 | 0.02 | F-actin-uncapping protein LRRC16A |
| PSIP1 | O75475 | 17 | 60181 | 1 | 0.05 | PC4 and SFRS1-interacting protein |
| CCAR1 | Q8IX12 | 16 | 133423 | 1 | 0.02 | Cell division cycle and apoptosis regulator protein 1 |
| RPL32 | P62910 | 15 | 15964 | 1 | 0.21 | 60S ribosomal protein L32 |
| USP46 | P62068 | 15 | 43156 | 1 | 0.08 | Ubiquitin carboxyl-terminal hydrolase 46 |
| RPLP0 | P05388 | 13 | 34423 | 1 | 0.1 | 60S acidic ribosomal protein P0 |

**Supplementary Table 4.** Predicted mRNAs capable of binding to PABPC1 from AURA

| COQ7 | HES7 | SH3BGRL3 | FBXO25 | SLA | SPOP |
| --- | --- | --- | --- | --- | --- |
| CFDP1 | CSF2 | TMLHE | UBE2V2 | IKBKE | RNPS1 |
| GPX4 | VPS33A | EGLN2 | UBE2J2 | STK39 | YBX1 |
| GNPAT | AQP3 | NDUFB6 | QARS | ARNTL | PKNOX2 |
| ENDOG | BGLAP | UQCRFS1 | NOSIP | PDE6D | ZFP161 |
| VAMP5 | FUT10 | NDUFA3 | LAT | MYCBP | IRX2 |
| NDUFV2 | PDCD1 | NDUFV1 | KCNK5 | CD7 | ZNF79 |
| TLX2 | DEAF1 | TXN2 | NAPG | ACTG2 | ZNF345 |
| COPS3 | PLEKHA1 | F12 | STX11 | TESK1 | ELK1 |
| GBX2 | IRX5 | LGALS8 | NUDT5 | EIF5A2 | IRF2 |
| GDF11 | PPP3CC | SFTPC | CRADD | PTGER4 | CARHSP1 |
| CASP6 | DFFB | CCL1 | PSMB8 | NR4A1 | ZNF32 |
| FOXE1 | TRIM39 | C1QTNF7 | PGGT1B | AIP | SCAND1 |
| SIX6 | ELMO1 | RNPEP | APOA1BP | YME1L1 | PREB |
| MSX1 | VAV1 | EFNA3 | C2orf7 | MRPL39 | SSBP3 |
| CD1D | LGALS7 | USF1 | SIGLEC10 | NUDT2 | TFDP2 |
| SYK | NUP62 | RAB3IP | LGALS9 | CDH12 | NFYB |
| COL8A1 | BRE | RGS14 | MAP2K2 | DGUOK | PAK6 |
| FOXD4 | CLN3 | PPP1R8 | AP3S1 | RPS6KB2 | NEK7 |
| PRDX2 | GZMA | GMFG | TIMM17B | GUK1 | CDK8 |
| HDAC3 | BCL2L12 | GIT2 | AP4B1 | XPNPEP2 | MVK |
| HIC1 | PTPN6 | PPP1CA | CDY1 | PRKACB | PSMD9 |
| NAB2 | TNFRSF25 | CD83 | C21orf45 | CLK3 | SAP18 |
| IRX3 | PAK1 | CD69 | PIN1 | CLK4 | MCRS1 |
| DTX1 | ITGB2 | HLA-DQB1 | RPIA | DBR1 | PFDN2 |
| ADM | RAN | CD48 | PPID | HPCAL1 | CD1A |
| VAV3 | GCH1 | TNFRSF4 | PMM2 | PTER | CD1C |
| FABP5 | IL16 | ABCG1 | CA8 | DPEP1 | CD1E |
| KRT1 | ISG20 | ETS1 | SHMT1 | ASRGL1 | CD1B |
| OSTF1 | IFNAR2 | RPL13 | MVD | PSMA5 | LZIC |
| CASP3 | THOC4 | MRPL1 | KCNK1 | PTPN7 | MAPKAP1 |
| ADRBK1 | IL23A | MRPL33 | SLC31A2 | TAF9 | LTA |
| CHUK | KIN | MRPL11 | TTYH1 | COPS5 | STX8 |
| CD3D | HSD11B2 | RPL39L | GTF2F2 | PTPN2 | PEX16 |
| EVL | TCEB1 | RPL27A | DDX31 | NGLY1 | STX10 |
| TAL1 | HBXIP | RPS23 | MOCS2 | CDC25A | SLC38A6 |
| GABPA | CUL4A | MRPL17 | B3GAT3 | RAB33B | MTX2 |
| RNF2 | DEFA3 | MRPL51 | C7orf10 | CLPP |  |
| PTPRC | ZYX | MRPL24 | TAF10 | VPS4A |  |
| HHIP | CCL4 | MRP63 | ZDHHC12 | RAB11B |  |
| HCLS1 | NOS2 | MRPS22 | MGAT1 | PXMP2 |  |
| TNF | UQCRB | MRPL23 | TPST2 | CTSW |  |
| STMN1 | NDUFA7 | MRPL16 | POLR2K | SNRPA1 |  |
| CD28 | ACAD8 | UCP3 | SETMAR | TARBP2 |  |
| PDCD10 | ALOX5AP | NUPL1 | PEMT | SNRPN |  |
| SCG2 | UROS | SLC27A2 | B4GALT3 | RBMS1 |  |
| FLI1 | NDUFB9 | SLC2A5 | AGPAT2 | CHAF1A |  |
| ARIH2 | ALDH6A1 | TAPBP | LCMT2 | TAF1A |  |
| DRD2 | NDUFS3 | SLC29A2 | RGS10 | TCEA2 |  |
| ODF3 | AKR1A1 | SLC19A1 | STAM | MAZ |  |
| PKHD1 | NDUFS7 | UBE2E1 | GNG4 | GTF3C5 |  |

**Supplementary Table 5**. Kyoto Encyclopedia of Genes and Genomes (KEGG) enrichment analysis from STRING database

| **Term ID** | **Term description** | **Observed gene count** | | **Background gene count** | **Strength** | **False**  **discovery**  **rate** | **Matching proteins in your network (IDs)** | **Matching proteins in your network (labels)** |
| --- | --- | --- | --- | --- | --- | --- | --- | --- |
| hsa04660 | T cell receptor signaling pathway | 14 | | 101 | 0.97 | 4.91E-07 | ENSP00000262948,ENSP00000278568,ENSP00000296871,ENSP00000300692,ENSP00000324890,ENSP00000335062,ENSP00000359073,ENSP00000359424,ENSP00000380878,ENSP00000391592,ENSP00000398698,ENSP00000406873,ENSP00000411355,ENSP00000472929 | MAP2K2,PAK1,CSF2,CD3D,CD28,PDCD1,VAV3,CHUK,PPP3CC,PTPN6,TNF,PAK6,PTPRC,VAV1 |
| hsa04650 | Natural killer cell mediated cytotoxicity | 13 | | 121 | 0.86 | 1.50E-05 | ENSP00000262948,ENSP00000278568,ENSP00000296871,ENSP00000311032,ENSP00000343957,ENSP00000359073,ENSP00000364907,ENSP00000380878,ENSP00000380948,ENSP00000391592,ENSP00000398698,ENSP00000472929,ENSP00000484431 | MAP2K2,PAK1,CSF2,CASP3,IFNAR2,VAV3,SYK,PPP3CC,ITGB2,PTPN6,TNF,VAV1,CD48 |
| hsa05146 | Amoebiasis | 11 | | 100 | 0.87 | 8.02E-05 | ENSP00000289429,ENSP00000296871,ENSP00000311032,ENSP00000327251,ENSP00000357149,ENSP00000357150,ENSP00000357152,ENSP00000357153,ENSP00000359719,ENSP00000380948,ENSP00000398698 | CD1A,CSF2,CASP3,NOS2,CD1E,CD1B,CD1C,CD1D,PRKACB,ITGB2,TNF |
| hsa05012 | Parkinson disease | 16 | | 240 | 0.65 | 0.00011 | ENSP00000216185,ENSP00000233627,ENSP00000263774,ENSP00000271308,ENSP00000276689,ENSP00000301457,ENSP00000306397,ENSP00000311032,ENSP00000322450,ENSP00000327268,ENSP00000354859,ENSP00000359719,ENSP00000369176,ENSP00000418438,ENSP00000430494,ENSP00000440485 | TXN2,NDUFS7,NDUFS3,PSMA5,NDUFB9,NDUFA7,UQCRFS1,CASP3,NDUFV1,NDUFV2,DRD2,PRKACB,NDUFB6,NDUFA3,UQCRB,PSMD9 |
| hsa05010 | Alzheimer disease | 19 | | 355 | 0.56 | 0.00018 | ENSP00000233627,ENSP00000262948,ENSP00000263774,ENSP00000271308,ENSP00000276689,ENSP00000301457,ENSP00000303754,ENSP00000306397,ENSP00000311032,ENSP00000322450,ENSP00000327251,ENSP00000327268,ENSP00000359424,ENSP00000369176,ENSP00000380878,ENSP00000398698,ENSP00000418438,ENSP00000430494,ENSP00000440485 | NDUFS7,MAP2K2,NDUFS3,PSMA5,NDUFB9,NDUFA7,PPID,UQCRFS1,CASP3,NDUFV1,NOS2,NDUFV2,CHUK,NDUFB6,PPP3CC,TNF,NDUFA3,UQCRB,PSMD9 |
| hsa05014 | Amyotrophic lateral sclerosis | 19 | | 352 | 0.56 | 0.00018 | ENSP00000233627,ENSP00000263774,ENSP00000271308,ENSP00000276689,ENSP00000301457,ENSP00000306397,ENSP00000311032,ENSP00000322450,ENSP00000327251,ENSP00000327268,ENSP00000369176,ENSP00000371155,ENSP00000380878,ENSP00000398698,ENSP00000418438,ENSP00000421592,ENSP00000430494,ENSP00000440485,ENSP00000471191 | NDUFS7,NDUFS3,PSMA5,NDUFB9,NDUFA7,UQCRFS1,CASP3,NDUFV1,NOS2,NDUFV2,NDUFB6,NUPL1,PPP3CC,TNF,NDUFA3,ALYREF,UQCRB,PSMD9,NUP62 |
| hsa04932 | Non-alcoholic fatty liver disease | 12 | | 148 | 0.74 | 0.00021 | ENSP00000233627,ENSP00000263774,ENSP00000276689,ENSP00000301457,ENSP00000306397,ENSP00000311032,ENSP00000322450,ENSP00000327268,ENSP00000369176,ENSP00000398698,ENSP00000418438,ENSP00000430494 | NDUFS7,NDUFS3,NDUFB9,NDUFA7,UQCRFS1,CASP3,NDUFV1,NDUFV2,NDUFB6,TNF,NDUFA3,UQCRB |
| hsa05020 | Prion disease | 16 | | 265 | 0.61 | 0.00021 | ENSP00000233627,ENSP00000263774,ENSP00000271308,ENSP00000276689,ENSP00000301457,ENSP00000306397,ENSP00000311032,ENSP00000322450,ENSP00000327268,ENSP00000359719,ENSP00000369176,ENSP00000380878,ENSP00000398698,ENSP00000418438,ENSP00000430494,ENSP00000440485 | NDUFS7,NDUFS3,PSMA5,NDUFB9,NDUFA7,UQCRFS1,CASP3,NDUFV1,NDUFV2,PRKACB,NDUFB6,PPP3CC,TNF,NDUFA3,UQCRB,PSMD9 |
| hsa05166 | Human T-cell leukemia virus 1 infection | 14 | | 211 | 0.65 | 0.00024 | ENSP00000240055,ENSP00000262948,ENSP00000296871,ENSP00000300692,ENSP00000359424,ENSP00000359719,ENSP00000372170,ENSP00000376436,ENSP00000380878,ENSP00000380948,ENSP00000398698,ENSP00000403495,ENSP00000446215,ENSP00000483056 | NFYB,MAP2K2,CSF2,CD3D,CHUK,PRKACB,MSX1,ETS1,PPP3CC,ITGB2,TNF,LTA,RAN,ELK1 |
| hsa04640 | Hematopoietic cell lineage | 9 | | 91 | 0.82 | 0.00055 | ENSP00000289429,ENSP00000296871,ENSP00000300692,ENSP00000312027,ENSP00000357149,ENSP00000357150,ENSP00000357152,ENSP00000357153,ENSP00000398698 | CD1A,CSF2,CD3D,CD7,CD1E,CD1B,CD1C,CD1D,TNF |
| hsa05170 | Human immunodeficiency virus 1 infection | | 13 | 204 | 0.63 | 0.00062 | ENSP00000262948,ENSP00000278568,ENSP00000300692,ENSP00000308413,ENSP00000311032,ENSP00000359424,ENSP00000364589,ENSP00000375727,ENSP00000380878,ENSP00000398698,ENSP00000404833,ENSP00000406873,ENSP00000478121 | MAP2K2,PAK1,CD3D,RPS6KB2,CASP3,CHUK,CUL4A,GNG4,PPP3CC,TNF,TAPBP,PAK6,TCEB1 |
| hsa00190 | Oxidative phosphorylation | 10 | | 130 | 0.71 | 0.0012 | ENSP00000233627,ENSP00000263774,ENSP00000276689,ENSP00000301457,ENSP00000306397,ENSP00000322450,ENSP00000327268,ENSP00000369176,ENSP00000418438,ENSP00000430494 | NDUFS7,NDUFS3,NDUFB9,NDUFA7,UQCRFS1,NDUFV1,NDUFV2,NDUFB6,NDUFA3,UQCRB |
| hsa03010 | Ribosome | 10 | | 130 | 0.71 | 0.0012 | ENSP00000288937,ENSP00000296102,ENSP00000296674,ENSP00000300151,ENSP00000307889,ENSP00000308897,ENSP00000315017,ENSP00000346015,ENSP00000354525,ENSP00000380466 | MRPL17,MRPL33,RPS23,MRPL16,RPL13,MRPL11,MRPL1,RPL27A,MRPL24,RPL23L |
| hsa05205 | Proteoglycans in cancer | 12 | | 196 | 0.61 | 0.0015 | ENSP00000262948,ENSP00000278568,ENSP00000308413,ENSP00000311032,ENSP00000320176,ENSP00000326031,ENSP00000359073,ENSP00000359719,ENSP00000391592,ENSP00000398698,ENSP00000472929,ENSP00000483056 | MAP2K2,PAK1,RPS6KB2,CASP3,HCLS1,PPP1CA,VAV3,PRKACB,PTPN6,TNF,VAV1,ELK1 |
| hsa05235 | PD-L1 expression and PD-1 checkpoint pathway in cancer | 8 | | 88 | 0.79 | 0.0019 | ENSP00000262948,ENSP00000300692,ENSP00000308413,ENSP00000324890,ENSP00000335062,ENSP00000359424,ENSP00000380878,ENSP00000391592 | MAP2K2,CD3D,RPS6KB2,CD28,PDCD1,CHUK,PPP3CC,PTPN6 |
| hsa04664 | Fc epsilon RI signaling pathway | 7 | | 66 | 0.85 | 0.002 | ENSP00000262948,ENSP00000296871,ENSP00000359073,ENSP00000364907,ENSP00000398698,ENSP00000472929,ENSP00000479870 | MAP2K2,CSF2,VAV3,SYK,TNF,VAV1,ALOX5AP |
| hsa04723 | Retrograde endocannabinoid signaling | 10 | | 145 | 0.67 | 0.002 | ENSP00000233627,ENSP00000263774,ENSP00000276689,ENSP00000301457,ENSP00000322450,ENSP00000327268,ENSP00000359719,ENSP00000369176,ENSP00000375727,ENSP00000418438 | NDUFS7,NDUFS3,NDUFB9,NDUFA7,NDUFV1,NDUFV2,PRKACB,NDUFB6,GNG4,NDUFA3 |
| hsa01100 | Metabolic pathways | 40 | | 1447 | 0.27 | 0.0026 | ENSP00000233627,ENSP00000255389,ENSP00000263774,ENSP00000264093,ENSP00000265471,ENSP00000268261,ENSP00000276689,ENSP00000281182,ENSP00000283646,ENSP00000287600,ENSP00000301012,ENSP00000301457,ENSP00000306397,ENSP00000307567,ENSP00000314407,ENSP00000316786,ENSP00000318868,ENSP00000322316,ENSP00000322450,ENSP00000327251,ENSP00000327268,ENSP00000335261,ENSP00000346103,ENSP00000355689,ENSP00000357787,ENSP00000360761,ENSP00000361140,ENSP00000368455,ENSP00000370201,ENSP00000373354,ENSP00000380157,ENSP00000400057,ENSP00000404718,ENSP00000418438,ENSP00000419045,ENSP00000419628,ENSP00000430494,ENSP00000443551,ENSP00000450436,ENSP00000480428 | NDUFS7,PEMT,NDUFS3,DGUOK,B3GAT3,PMM2,NDUFB9,ACAD8,RPIA,PDE6D,MVD,NDUFA7,UQCRFS1,QARS,CA8,HSD11B2,SHMT1,COQ7,NDUFV1,NOS2,NDUFV2,TMLHE,GPX4,GUK1,UROS,AGPAT2,AKR1A1,NUDT2,TAF9,SETMAR,MOCS2,ASRGL1,MGAT1,NDUFA3,GCH1,NUDT5,UQCRB,MVK,ALDH6A1,B4GALT3 |
| hsa05163 | Human cytomegalovirus infection | 12 | | 218 | 0.57 | 0.0028 | ENSP00000262948,ENSP00000302846,ENSP00000308413,ENSP00000311032,ENSP00000359424,ENSP00000359719,ENSP00000375727,ENSP00000380878,ENSP00000398698,ENSP00000404833,ENSP00000482259,ENSP00000483056 | MAP2K2,PTGER4,RPS6KB2,CASP3,CHUK,PRKACB,GNG4,PPP3CC,TNF,TAPBP,CCL4,ELK1 |
| hsa05016 | Huntington disease | 14 | | 298 | 0.5 | 0.0037 | ENSP00000233627,ENSP00000263774,ENSP00000271308,ENSP00000276689,ENSP00000301457,ENSP00000306397,ENSP00000311032,ENSP00000322450,ENSP00000327268,ENSP00000342889,ENSP00000369176,ENSP00000418438,ENSP00000430494,ENSP00000440485 | NDUFS7,NDUFS3,PSMA5,NDUFB9,NDUFA7,UQCRFS1,CASP3,NDUFV1,NDUFV2,POLR2K,NDUFB6,NDUFA3,UQCRB,PSMD9 |
| hsa04662 | B cell receptor signaling pathway | 7 | | 78 | 0.78 | 0.0039 | ENSP00000262948,ENSP00000359073,ENSP00000359424,ENSP00000364907,ENSP00000380878,ENSP00000391592,ENSP00000472929 | MAP2K2,VAV3,CHUK,SYK,PPP3CC,PTPN6,VAV1 |
| hsa04714 | Thermogenesis | 12 | | 229 | 0.55 | 0.0039 | ENSP00000233627,ENSP00000263774,ENSP00000276689,ENSP00000301457,ENSP00000306397,ENSP00000308413,ENSP00000322450,ENSP00000327268,ENSP00000359719,ENSP00000369176,ENSP00000418438,ENSP00000430494 | NDUFS7,NDUFS3,NDUFB9,NDUFA7,UQCRFS1,RPS6KB2,NDUFV1,NDUFV2,PRKACB,NDUFB6,NDUFA3,UQCRB |
| hsa04062 | Chemokine signaling pathway | 10 | | 186 | 0.56 | 0.0095 | ENSP00000225842,ENSP00000278568,ENSP00000312185,ENSP00000312262,ENSP00000359073,ENSP00000359424,ENSP00000359719,ENSP00000375727,ENSP00000472929,ENSP00000482259 | CCL1,PAK1,ELMO1,ADRBK1,VAV3,CHUK,PRKACB,GNG4,VAV1,CCL4 |
| hsa05211 | Renal cell carcinoma | 6 | | 66 | 0.79 | 0.0095 | ENSP00000262948,ENSP00000278568,ENSP00000376436,ENSP00000406873,ENSP00000469686,ENSP00000478121 | MAP2K2,PAK1,ETS1,PAK6,EGLN2,TCEB1 |
| hsa04625 | C-type lectin receptor signaling pathway | 7 | | 102 | 0.66 | 0.0151 | ENSP00000228534,ENSP00000278568,ENSP00000359424,ENSP00000364907,ENSP00000380878,ENSP00000398698,ENSP00000464030 | IL23A,PAK1,CHUK,SYK,PPP3CC,TNF,IKBKE |
| hsa04146 | Peroxisome | 6 | | 79 | 0.71 | 0.0196 | ENSP00000241041,ENSP00000267842,ENSP00000321271,ENSP00000327251,ENSP00000355607,ENSP00000443551 | PEX16,SLC27A2,PXMP2,NOS2,GNPAT,MVK |
| hsa05132 | Salmonella infection | 10 | | 209 | 0.51 | 0.0196 | ENSP00000216185,ENSP00000262948,ENSP00000267199,ENSP00000278568,ENSP00000311032,ENSP00000312185,ENSP00000359424,ENSP00000398698,ENSP00000411355,ENSP00000466298 | TXN2,MAP2K2,VPS33A,PAK1,CASP3,ELMO1,CHUK,TNF,PTPRC,STX10 |
| hsa01523 | Antifolate resistance | 4 | | 31 | 0.94 | 0.0198 | ENSP00000308895,ENSP00000318868,ENSP00000359424,ENSP00000398698 | SLC19A1,SHMT1,CHUK,TNF |
| **hsa04010** | **MAPK signaling pathway** | **12** | | **288** | **0.45** | **0.0198** | **ENSP00000262948,ENSP00000278568,ENSP00000309116,ENSP00000311032,ENSP00000357393,ENSP00000359424,ENSP00000359719,ENSP00000380878,ENSP00000398698,ENSP00000410452,ENSP00000440864,ENSP00000483056** | **MAP2K2,PAK1,PTPN7,CASP3,EFNA3,CHUK,PRKACB,PPP3CC,TNF,STMN1,NR4A1,ELK1** |
| hsa05167 | Kaposi sarcoma-associated herpesvirus infection | 9 | | 187 | 0.51 | 0.0278 | ENSP00000262948,ENSP00000296871,ENSP00000311032,ENSP00000343957,ENSP00000359424,ENSP00000364907,ENSP00000375727,ENSP00000380878,ENSP00000464030 | MAP2K2,CSF2,CASP3,IFNAR2,CHUK,SYK,GNG4,PPP3CC,IKBKE |
| hsa04666 | Fc gamma R-mediated phagocytosis | 6 | | 90 | 0.65 | 0.0314 | ENSP00000278568,ENSP00000308413,ENSP00000359073,ENSP00000364907,ENSP00000411355,ENSP00000472929 | PAK1,RPS6KB2,VAV3,SYK,PTPRC,VAV1 |
| hsa05135 | Yersinia infection | 7 | | 125 | 0.58 | 0.0352 | ENSP00000262948,ENSP00000312185,ENSP00000347464,ENSP00000359073,ENSP00000359424,ENSP00000398698,ENSP00000472929 | MAP2K2,ELMO1,GIT2,VAV3,CHUK,TNF,VAV1 |
| hsa04210 | Apoptosis | 7 | | 132 | 0.55 | 0.0454 | ENSP00000262948,ENSP00000265164,ENSP00000311032,ENSP00000311300,ENSP00000359424,ENSP00000361725,ENSP00000398698 | MAP2K2,CASP6,CASP3,CTSW,CHUK,ENDOG,TNF |
| hsa05152 | Tuberculosis | 8 | | 168 | 0.51 | 0.0454 | ENSP00000228534,ENSP00000240055,ENSP00000311032,ENSP00000327251,ENSP00000364907,ENSP00000380878,ENSP00000380948,ENSP00000398698 | IL23A,NFYB,CASP3,NOS2,SYK,PPP3CC,ITGB2,TNF |
| hsa04620 | Toll-like receptor signaling pathway | 6 | | 101 | 0.6 | 0.0469 | ENSP00000262948,ENSP00000343957,ENSP00000359424,ENSP00000398698,ENSP00000464030,ENSP00000482259 | MAP2K2,IFNAR2,CHUK,TNF,IKBKE,CCL4 |
| hsa05140 | Leishmaniasis | 5 | | 70 | 0.68 | 0.0469 | ENSP00000327251,ENSP00000380948,ENSP00000391592,ENSP00000398698,ENSP00000483056 | NOS2,ITGB2,PTPN6,TNF,ELK1 |

**Supplementary Table 6.** List of antibodies used in western blotting, co-immunoprecipitation, ChIP, RIP, and IHC.

| **Antibody** | **Vendor** | **Catalog No.** | **Application** |
| --- | --- | --- | --- |
| HOXD9 | Proteintech | 20560-1-AP | WB: 1:800  IHC: 1:50 |
| HOXD9 | Cell signaling | #55962 | ChIP: 2μg |
| PABPC1 | Proteintech | 10970-1-AP | IP: 2μg  RIP:5μg |
| PAPBC1 | ABclonal | A14872 | WB: 1:1000  IHC: 1:300 |
| Ubiquitin | Proteintech | 10201-2-AP | WB: 1:1000 |
| GAPDH | Proteintech | 60004-1-Ig | WB: 1:30000 |
| β-tubulin | Ray antibody | RM2003 | WB: 1:1000 |
| Vimentin | Proteintech | 10366-1-AP | WB: 1:2000 |
| E-cadherin | Proteintech | 20874-1-AP | WB: 1:5000  IHC: 1:2000 |
| MMP2 | Proteintech | 10373-2-AP | WB: 1:1000 |
|  |  |  | IHC: 1:200 |
| Ki-67 | Proteintech | 27309-1-AP | IHC: 1:5000 |
| 6*His tag | Proteintech | 66005-1-Ig | WB: 1:10000  IP: 2μg  RIP:5μg |
| PAK1 | Proteintech | 21401-1-AP | WB: 1:1000  IHC: 1:50 |

**Supplementary Table 7.** List of primers used for amplification

| **Experiment** | **Name** | **Position or orientation** | **Sequence (5’-3’)** |
| --- | --- | --- | --- |
| qPCR | HOXD9 | F | GCAGCAACTTGACCCAAACAACC |
|  |  | R | AATTCTTTCTCCAGCTCAAGCG |
|  | PAXIP1-AS1 | F | GTTAATACTACACAGCGGCGAT |
|  |  | R | TTCTTCTCCCAACTTCCGGTC |
|  | PABPC1 | F | CATCCTCTCCATCCGGGTC |
|  |  | R | CTGCTGGAAGTTCACATACGC |
|  | GAPDH | F | AAATCCCATCACCATCTTCC |
|  |  | R | TCACACCCATGACGAACA |
|  | B2M | F | AAATCCCATCACCATCTTCC |
|  |  | R | TCACACCCATGACGAACA |
|  | NEAT1 | F | TGTGGTGGTGGGTGCCTGTAG |
|  |  | R | TGACTGTAACCTCCGCCTCCTG |
|  | PAK1 | F | CAGCCCCTCCGATGAGAAATA |
|  |  | R | CAAAACCGACATGAATTGTGTGT |
|  | TNF | F | CCCTCTATTTATGTTTGCACT |
|  |  | R | TCACGGAAAACATGTCTGAG |
|  | NR4A1 | F | TTGACTTGGCTCATTCCCC |
|  |  | R | CTGTAGTCCCAGCTACTCT |
|  | MAP2K2 | F | TCACCCCTGCGGAGAGCACC |
|  |  | R | CCCGTCCCCAGAGGCACCC |
|  | EFNA3 | F | GTGCCCTCTCCCTTTGTCC |
|  |  | R | CCCTTGCCCATCTAGTCCC |
|  | PRKACB | F | AAATACTCATTGTAAGCCTG |
|  |  | R | GTAGTGCATAGGAAATTCCA |
|  | PTPN7 | F | TGCTCCTGCCTCCATGGTT |
|  |  | R | AACAGCCAAGCTCTCTAGGTG |
|  | STMN1 | F | AGGCAATAGAAGAGAACAACA |
|  |  | R | TGCTTATCCTTCTCTCGCAAA |
|  | ELK1 | F | CTGCCTCCTAGCATTCACTTC |
|  | ELK1 | R | GCTGCCACTGGATGGAAACT |
|  | CASP3 | F | CATGGAAGCGAATCAATGGACT |
|  |  | R | CTGTACCAGACCGAGATGTCA |
|  | CHUK | F | GGCTTCGGGAACGTCTGTC |
|  |  | R | TTTGGTACTTAGCTCTAGGCGA |
|  | PPP3CC | F | ACCGCGTCATCAAAGCTGT |
|  |  | R | CTTCCAGTCGTCCTTCCTTTAC |
| ChIP (PAXIP1-AS1 promoter) | Site 1 | F: -1361~-1337 | ATTCAACTAAGATGCATTCCTACCA |
|  |  | R: -1192~-1172 | CCTTGATTTTCCCACTTGGAC |
|  | Site 2 | F: -1614 ~ -1595 | GGCAAAAAGGGTCCAAAAAG |
|  |  | R: -1444 ~ -1425 | CCTTGGGTGTGACATCAGTG |

**Supplementary Table 8.** List of siRNAs for transient transfection

| **Name** | **siRNA** | **Sense** |
| --- | --- | --- |
| HOXD9 | siRNA1 | 5'- CAGCAACUUGACCCAAACATT -3' |
|  | siRNA2 | 5' CACCAAAUACCAGACGCUUTT-3' |
|  | siRNA3 | 5'- AAAUAUAGCAUUUCUUAAGGA-3' |
| PAXIP1-AS1 | siRNA1 | 5'- CAUAUAUCAGUGUAAUAAATT-3' |
|  | siRNA2 | 5'- CAUAUAUCAGUGUAAUAAATT-3' |
|  | siRNA3 | 5'- GCCAAAUAUACGGGUUAUCUATT-3' |
| PABPC1 | siRNA1 | 5'- GGCUAUGGAUUUGUACACUTT-3' |
|  | siRNA2 | 5'- GCCUGCCUUAAGUGUGAAATT-3' |
|  | siRNA3 | 5'- GACCACCAUUUAGUACUAUTT-3' |
| PAK1 | siRNA1 | 5'- GGCGAUCCUAAGAAGAAAUTT-3' |
|  | siRNA2 | 5'- GAUGCUUUGACCCGGAAUATT-3' |
|  | siRNA3 | 5'- CCCUAAACCAUGGUUCUAATT-3' |
